# Genomic expression differences between cutaneous cells from red hair color individuals and black hair color individuals based on bioinformatic analysis

**DOI:** 10.18632/oncotarget.14140

**Published:** 2016-12-24

**Authors:** Joan Anton Puig-Butille, Pol Gimenez-Xavier, Alessia Visconti, Jérémie Nsengimana, Francisco Garcia-García, Gemma Tell-Marti, Maria José Escamez, Julia Newton-Bishop, Veronique Bataille, Marcela del Río, Joaquín Dopazo, Mario Falchi, Susana Puig

**Affiliations:** ^1^ Biochemistry and Molecular Genetics Department, Melanoma Unit, Hospital Clinic & IDIBAPS, CIBER de Enfermedades Raras (CIBERER), Barcelona, Spain; ^2^ Dermatology Department, Melanoma Unit, Hospital Clinic & IDIBAPS, CIBER de Enfermedades Raras (CIBERER), Barcelona, Spain; ^3^ Department of Twin Research and Genetic Epidemiology, King's College London, London, UK; ^4^ Section of Epidemiology and Biostatistics, Leeds Institute of Cancer and Pathology, University of Leeds, Leeds, UK; ^5^ Computational Genomics Department, Centro de Investigación Príncipe Felipe (CIPF), Valencia, Spain; ^6^ Departamento de Bioingeniería, Universidad Carlos III de Madrid, CIEMAT, IIS-Fundación Jiménez Díaz, CIBER de Enfermedades Raras (CIBERER), Madrid, Spain; ^7^ Functional Genomics Node, (INB) at CIPF, Valencia, Spain; ^8^ CIBER de Enfermedades Raras (CIBERER), Valencia, Spain

**Keywords:** MC1R, red hair phenotype, melanocyte, melanoma, autophagy

## Abstract

The *MC1R* gene plays a crucial role in pigmentation synthesis. Loss-of-function *MC1R* variants, which impair protein function, are associated with red hair color (RHC) phenotype and increased skin cancer risk. Cultured cutaneous cells bearing loss-of-function *MC1R* variants show a distinct gene expression profile compared to wild-type *MC1R* cultured cutaneous cells. We analysed the gene signature associated with RHC co-cultured melanocytes and keratinocytes by Protein-Protein interaction (PPI) network analysis to identify genes related with non-functional *MC1R* variants. From two detected networks, we selected 23 nodes as hub genes based on topological parameters. Differential expression of hub genes was then evaluated in healthy skin biopsies from RHC and black hair color (BHC) individuals. We also compared gene expression in melanoma tumors from individuals with RHC versus BHC. Gene expression in normal skin from RHC cutaneous cells showed dysregulation in 8 out of 23 hub genes (*CLN3*, *ATG10*, *WIPI2*, *SNX2*, *GABARAPL2*, *YWHA, PCNA* and *GBAS*). Hub genes did not differ between melanoma tumors in RHC versus BHC individuals. The study suggests that healthy skin cells from RHC individuals present a constitutive genomic deregulation associated with the red hair phenotype and identify novel genes involved in melanocyte biology.

## INTRODUCTION

Human cutaneous pigmentation is dependent on melanin pigment production (eumelanin and pheomelanin) by epidermal and follicular melanocytes. Melanin synthesis is controlled by the melanocortin receptor type 1 (*MC1R*) which encodes a 7-pass transmembrane G-protein-coupled receptor. In wild-type *MC1R* melanocytes, activation of the receptor by the α-melanocyte stimulating hormone (α-MSH) promotes the synthesis of eumelanin pigment (dark pigment), reduces UV-induced oxidative stress and enhances DNA repair through base-excision repair and NER mechanisms which repairs UV-photoproducts [1]. *MC1R* is a highly polymorphic gene and loss-of-function variants (p.R151C, p.R142H, p.R161W, p.D294H p.D84E and p.I155T) result in a minimal receptor activity and mainly produces pheomelanin (red/yellow) pigment [2].

Epidemiological studies have found that loss-of-function variants in *MC1R* can, in part, predict the red-hair color (RHC) phenotype (red hair, fair skin, low tan capacity and high UV sensitivity) as well as melanoma [3] and non-melanoma skin cancer risk [4]. The decreased eumelanin production compared to pheomelanin in RHC individuals increases skin cancer risk due to the weak UV shielding capacity of pheomelanin, increase in UVA-induced reactive oxygen species [5, 6] and altered NER pathway. However, recent data indicate that RHC *MC1R* variants also contribute to carcinogenesis by UV-independent mechanisms. Mitra D et al. observed that the absence of pheomelanin is protective against melanoma development in mice models [7]. They detected high levels of oxidative DNA and lipid damage in RHC mice in a UV-independent model which still leads to oxidative damage. In addition, we h[2]ave previously reported that co-cultured melanocytes and keratinocytes harbouring loss-of-function *MC1R* variants show a constitutive overexpression of genes involved in oxidative phosphorylation pathway and DNA repair mechanisms [8].

The aim of this study was to further identify genes related to the RHC phenotype by analysing the previously reported expression signature pattern associated with RHC MC1R variants [8] using protein-protein interaction (PPI) network analyses. Gene co-expression networks have the potential to highlight specific molecular mechanisms and genes related to a specific trait or disease [9–11]. Expression of candidate genes from the PPI network analyses were further analysed in independent gene expression data generated from normal skin biopsy from RHC individuals and Black hair color (BHC) using the UK MuTHER dataset and in a subset of melanomas from RHC and BHC patients.

## RESULTS

**Figure 1:**
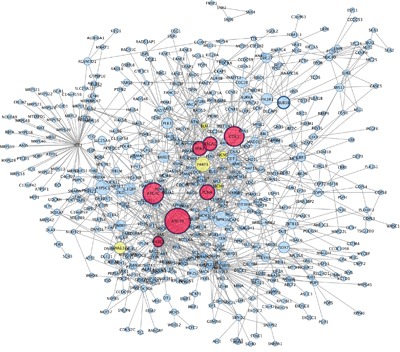
Protein Protein interaction network among up–regulated genes detected in co-cultured keratinocytes and melanocytes from individuals harbouring Red hair *color MC1R* variants (GSE44805 dataset) Nodes represent genes and edges indicate connections between proteins. Nodes are coloured based on number of degree in: low connected node (blue), medium connected node (yellow) and high connected node (red). Node size is proportional to the betweenness centrality value: the higher the value, the larger the node. Hub genes are represented with a thicker black border. The *GBAS*, *PRKAA1*, *ICT1* and *SNX2* hub genes do not follow the node size and border criteria to improve graphical representation of the network.

**Figure 2:**
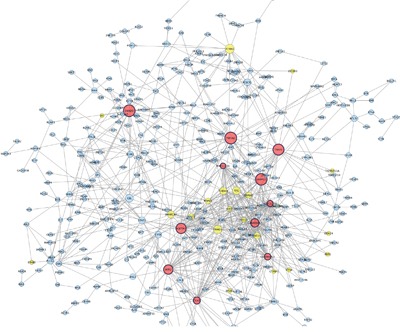
Protein Protein interaction network among down–regulated genes detected in co-cultured keratinocytes and melanocytes from individuals harbouring Red hair color *MC1R variants* (GSE44805 dataset) Nodes represent genes and edges indicate interaction between proteins. Nodes are coloured based on number of degree in: low connected node (blue), medium connected node (yellow) and high connected node (red). Node size indicate Betweenness centrality values. Hub genes are represented with a thicker black border.

We identified 3,570 differentially expressed genes (DEGs) in co-cultured melanocytes and keratinocytes from a pair of RHC siblings carrying non-functional *MC1R* variants versus a pair of siblings with wild-type *MC1R* alleles as previously described [8]. The expression signature pattern associated with RHC *MC1R* variants was evaluated by PPI network analysis. Networks from up-regulated and down-regulated DEGs were constructed separately as, from a systems biology perspective, functionally-related genes are frequently co-expressed across a set of samples [12] and up-regulated and down-regulated transcripts tend to sub-connect [13, 14]. A statistically significant network composed of 557 nodes was detected among the set of 1954 up-regulated genes (p< 0.001; Figure 1). Also, we found a statistically significant network composed of 450 nodes among the set of 1616 down-regulated genes (p<0.001, Figure 2). Topological information of networks and name of nodes (genes) are indicated in Supplementary Data Table S1 and Table S2.

**Table 1:** Topological information of networks detected among deregulated genes detected co-cultured keratinocytes and melanocytes from individuals harbouring Red hair color MC1R variants (GSE44805 dataset)

**Degree Centrality**						
Network	N. of nodes	N. of edges	HighestValue	Lowest Value	Mean ± SD	Hub genes*
Upregulated genes	514	1144	77	1	4.45±6.78	*GBAS (77), PRKAA1 (54), ICT1* (54), *ATG4C (42), ATG10 (50) PIK3C3 (30), PCNA (28), CDK1 (27), BRCA1(27) RPA1 (25)*, *BUB1B (12)^a^, SNX2(3)^a^*
Downregulated genes	411	818	42	1	3.98±6.07	*GABARAPL2 (42) SQSTM1 (39)GABARAPL1 (38), MAP1LC3B (38), WIPI2 (34), MAP1LC3A(31*) *CLN3(30), YWHAG(29*), *SMAD3(27), TRAF2(27), PABPC1(24*)
**Betweenness Centrality**						
Network	N. of nodes	N. of edges	HighestValue	Lowest Value	Mean ± SD	Hub genes*
Upregulated genes	514	1144	0.66	0.00	0.01±0.03	*SNX2(0.66), GBAS (0.29), ICT1* (0.18), *PRKAA1 (0.16), ATG10 (0.11),ATG4C (0.09), CDK1 (0.08), PCNA (0.06), RPA1 (0.05)*, *BRCA1(0.05) BUB1B (0.04),PIK3C3 (0.03)*
Down regulated genes	411	818	0.15	0.00	0.01±0.02	*SMAD3(0.15), YWHAG (0.15*), *PABPC1(0.14*) *TRAF2(0.13), SQSTM1 (0.12), WIPI2 (0.10) CLN3(0.06), GABARAPL1 (0.05), MAP1LC3B (0.05) GABARAPL2(0.04), MAP1LC3A(0.04*)

Numberof nodes, number of edges, Betweenness Centrality and Degree Centrality from both detected networks, *Name of hub genes and its Betweenness centrality and degree values. ^a^: *BUB1B* and *SNX2* were selected based on its Betweenness Centrality values.

**Table 2:** Differential Expression of hub genes in healthy skin from Red hair color individuals vs Black hair color individuals

ID	Results for whole list of genes^(A)^(N=1952)			Results from list of hub genes^(B)^(N=18)		
	logFC	p.value	adj. p.value	logFC	p.value	adj. p.value
*ATG10*	-0.431	0.001	0.032	-0.432	> 0.001	0.007
*CLN3*	-0.339	> 0.001	0.024	-0.339	> 0.001	0.007
*GABARAPL2*	ns	ns	ns	0.254	0.0188	0.056
*GBAS*	0.374	0.005	0.067	0.373	0.005	0.017
*PCNA*	0.209	> 0.001	0.026	0.209	0.001	0.007
*SNX2*	0.506	0.002	0.041	0.508	0.001	0.007
*WIPI2*	ns	ns	ns	-0.156	0.034	0.076
*YWHAG*	ns	ns	ns	0.273	0.023	0.060

Differential expression of hub genes was assessed (A) by analyzing the whole gene expression dataset and (B) by analyzing the differential expression of only hub genes. Table shows hub genes with statistically significant p-values and adjusted P-values (adj.p.value). logFC= logarithm of Fold Change; ns=not significant P-value

We evaluated degree and betweenness centrality in both networks to investigate relationship between nodes and select the hub genes in each network. Twenty-three nodes were selected as hub genes (Table 1). In both networks, highly connected nodes tend to also show high betweenness centrality values. However, two nodes with a low degree centrality value (*BUB1B* and *SNX2* gene) were selected based on their high Betweenness centrality value.

**Figure 3:**
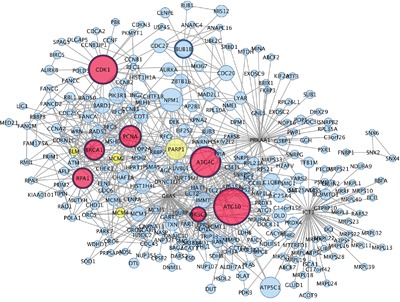
Hub genes and their first connected genes from network detected among up-regulated genes in co-cultured keratinocytes and melanocytes from individuals harbouring Red hair color *MC1R* variants (GSE44805 dataset) Only the first connection of hub genes is indicated. Nodes represent proteins and edges indicate connections between proteins. Nodes are coloured based on number of degree: Red (degrees >μ+ 3σ; x>24.8), yellow (μ+ 3σ < degree>μ+ 2σ; 24.8<degree>18.6), blue (degrees<μ+2σ, degree<18.6). Node size indicate betweenness centrality values. Hub genes are represented with a thicker black border. The hub genes (N=13 nodes) are directly connected with 270 out of 557 nodes within network. The *GBAS*, *PRKAA1*, *ICT1* and *SNX2* hub genes do not follow the node size and border criteria to improve graphical representation of the network.

In the network of up-regulated genes, 13 nodes were classified as hub genes (Figure 3). These genes play a role in DNA repair and cell-cycle homeostasis (*PRKAA1, CDK1, BUB1B, PCNA, RPA1* and *BRCA1*), oxidative phosphorylation (*GBAS, ICT1*) and autophagy (*PIK3C3, ATG4C, ATG10* and *SNX2*). The neighbouring genes of each hub gene are listed in Supplementary Tables S3-S14.

**Figure 4:**
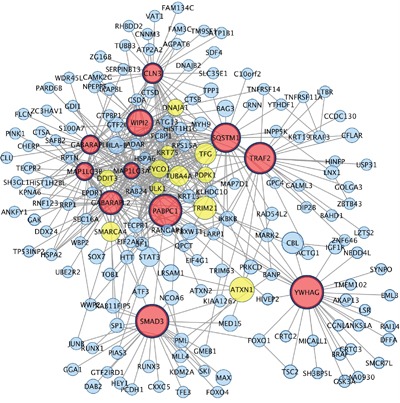
Hub genes and their first connected genes from network detected among down-regulated genes in co-cultured keratinocytes and melanocytes from individuals harbouring Red hair color *MC1R* variants (GSE44805 dataset) Only the first connection of hub genes is indicated. Nodes represent genes and edges indicate direct interaction between proteins. Nodes are coloured based on number of degree in Red (degrees >μ+ 3σ; x>22.2), yellow ( μ+ 3σ < degree>μ+ 2σ; 22.2<degree>16.3), blue (degrees< μ+2σ, degree<16.3 Node size indicate Betweenness centrality values. Hub genes are those nodes with the widest border. The hub genes (N=11 nodes) connects 177 out of 411 nodes.

In the network of down-regulated genes, 11 nodes were classified as hub genes (Figure 4). These genes are involved in apoptosis (*SMAD3, YWHAG*), mRNA metabolism (*PABPC1*), and autophagy (*TRAF2, SQSTM1, CLN3, WIPI2, GABARAPL1, GABARAPL2, MAP1LC3B, MAP1LC3A*). The neighbouring genes of each hub gene are listed in Supplementary Tables S15-S25.

Next, we assessed the differential expression of those 23 genes in an additional whole genome expression dataset of healthy skin biopsies from the TwinsUK MuTHER dataset in 14 RHC individuals and 7 BHC individuals. At 10% FDR, genome wide differential expression analyses identified 1,952 DEGs between RHC and BHC individuals, consisting of 1,074 up-regulated and 878 down-regulated genes in RHC (Supplementary Table S26). Overall, 378 DEGs were common between the healthy skin and the co-cultured melanocyte-keratinocyte datasets. No statistically significant PPI networks were detected among DEGs sets (data not shown). Expression data from 18 out of 23 hub genes identified in the melanocyte-keratinocyte network analysis was available in the MuTHER dataset (*MAPL1C3B* was not included in the array and *PABPC1, ATG4C, CDK1* and *PIK3C3* genes failed the quality control). Five out of 18 genes classified as hub genes in the melanocyte-keratinocyte network analysis were differentially expressed in the MuTHER dataset with RHC skin showing down-regulation of *CLN3* and *ATG10* genes and up-regulation of *SNX2*, *PCNA* and *GBAS* genes. When the differential expression analysis was restricted to these 18 hub genes, up-regulation of *GABARAPL2* and *YWHA* genes and down-regulation of *WIPI2* gene also reached statistically significant values (Table 2).

Finally, expression of hub genes was evaluated in a whole genome expression dataset of 26 melanomas from 6 BHC and 20 RHC patients. None of these genes were found to be differentially expressed between tumors from RHC patients compared with BHC patients. Interestingly, the top two upregulated genes in melanomas from RHC were *PRKAA1* (fold change=1.37, p=0.08) and *PIK3C3* (fold change=1.23, p=0.09) while the top two downregulated were *YWHAG* (fold change=0.62, p=0.29) and *CLN3* (fold change=0.77, p=0.3). These p-values were unadjusted for multiple testing.

## DISCUSSION

In this study, we aimed to uncover genes associated with the RHC phenotype by analysing a previously reported gene expression pattern [8]. By PPI network analysis, we identified two gene co-expression networks that reached statistically significant values in loss-of-function *MC1R* variants cutaneous cells. Since highly connected nodes are central to the network's architecture [15], the study was focused on those nodes with higher degree and betweenness centrality values. Overall, 23 nodes were selected as hub genes and their expression was analysed in an additional dataset from non-lesional skin tissue from RHC individuals or BHC individuals. We detected a gene expression pattern composed of 1,952 transcripts in cutaneous cells from RHC individuals indicating that skin from those individuals presents a distinct gene expression pattern compared to BCH individuals independent of the UV radiation effect. Altogether, these results confirm that loss-of-function *MC1R* variants promote a constitutive genomic deregulation associated with pheomelanin synthesis as observed *in vitro* [8] and in mice models [7]. Three hundred seventy-eight genes were in common in the set of deregulated transcripts both *in vitro* and *in vivo* cutaneous cells from RHC individuals, including a set of the network's hub genes such as *CLN3*, *ATG10*, *WIPI2, SNX2*, *PCNA*, *GBAS, GABARAPL2* and *YWHAG*.

Notably, *CLN3, ATG10*, *WIPI2*, *SNX2* and *GABARAPL2* are members of the autophagy interaction network [16]. Autophagy is a highly conserved lysosomal pathway involved in tissue homeostasis, adaptation to starvation and removal of dysfunctional organelles or pathogens [17]. Additional autophagy genes (*ATG12, ATG2A*, *ATG9A, ATG7)* are also down-deregulated in healthy skin from RHC individuals (Supplementary Table S26). Previous data suggest that regulators of autophagosome formation play a role in melanosome formation and destruction of abnormal melanosomes [18]. Autophagosome formation is a multistep process in which the expansion and closure of the vesicle membrane is controlled by the UBL complex [19]. Notably, most of deregulated genes (*ATG7, ATG12, ATG10*, *ATG5* and *WIP*I) are related to the UBL complex [20, 21]. Moreover, cutaneous cells from RHC individuals show over-expression of *GABARAPL2* which is essential for autophagosome maturation [19] and down-regulation of *CLN3* which play a role in autophagy, endocytosis and vesicular trafficking [16]. Previous comparison of black and red human hair melanosomes reveals differences between eumelanosomes and pheomelanosomes in physical terms such as shape or structural integrity [22]. Our findings support that autophagy genes are closely related to melanosome biogenesis and further suggest that these genes could underlie part of the physical differences between types of melanosomes.

Findings from healthy skins could not be replicated in melanomas. This could be because if the network's hub genes play a role in melanoma development, it may be in early tumor initiation as opposed to progression. In addition, gene expression is under a tighter regulatory control in tumors than in healthy tissues and differences are often subtler, requiring a larger sample to be detected. The largest fold change between RHC and BHC hub gene expression was 1.37 and the power to detect a difference of this magnitude at α=0.05 with 20 RHC and 6 BHC tumors was only 37% without multiple testing adjustments, reducing to 5% with adjustment. This is in contrast with the gene showing the largest difference in healthy skins (fold change=2.7, power=0.97 after multiple-tests correction). Nonetheless, the tumor data reinforce the possible involvement of cell cycle homeostasis and autophagy as *PRKAA1* and *PIK3C3* were the top 2 ranked genes. Additional studies of genes identified in this study should be conducted in skin cancer tumors to elucidate their role in malignant transformation.

A previous PPI network analyses conducted in a whole gene expression dataset of 31 primary melanomas and 52 metastases reported a PPI network in which *PCNA, CDK1, MAD2L1, RFC4* and *BRCA1* genes showed highest degree centrality values among upregulated genes in metastases [14]. We observed a similar behaviour in *in vitro* RHC cutaneous cells since *PCNA*, *CDK1* and *BRCA1* were hub genes in the network of up-regulated genes. However, only *PCNA* reaches statistically significant values in *in vivo* RHC cutaneous cells. Since these genes are essential for cell cycle progression and DNA repair [23–24], these findings suggest that up-regulation of these genes may be caused by an increased and continuous DNA damage. The intrinsic pheomelanin pathway represents an additional contributor to DNA damage by increasing oxidation [7, 8]. However, the molecular process underlying the increased DNA damage is poorly understood. Both *in vitro* and *in vivo* RHC cutaneous cells show an overexpression of *GBAS* which encodes a mitochondrial protein involved in oxidative phosphorylation [25, 26]. Further studies are required to clarify the biological role of mitochondrial related gene in melanocyte and melanogenesis.

The main limitations of present study are the disparity between platforms used for gene expression capture (resulting in presence/absence of gene probes and probe design), the low frequency of phenotypically black hair individuals included in the datasets and the differences in type of samples analyzed (cultured skin cells vs non-cultured skin cells) Establishment of cultured cutaneous cells might influence the expression of certain genes resulting in distinct expression such as *ATG10* gene expression which is found up-regulated in cultured cells and down-regulated in non-cultured cells.

In summary, the genes identified may help to reveal underlying molecular mechanisms associated with red hair color phenotype and future studies of these genes may provide insight to better understand the increased skin cancer risk observed in individuals harbouring loss-of-function MC1R variants.

## MATERIALS AND METHODS

Whole genome expression data from three different datasets were included in the study. An initial dataset included whole genome expression from four co-cultured keratinocytes and melanocytes from two pair of siblings. Phenotypical data and genetic variants in melanoma susceptibility genes *MC1R* and *CDKN2A* were obtained in all individuals. A pair of siblings, phenotypically classified as RHC individuals were double heterozygous for the p.R160W, p.R151C variants in *MC1R* (individual 1 and individual 2 were 38 and 33 years old, respectively). In contrast, a pair of siblings with brown hair color carried wild-type *MC1R* alleles (individual 1 and individual 2 were 50 and 51 years old, respectively). Moreover, the germline p.G101W *CDKN2A* mutation was detected in one of each pair of siblings. Extraction of the expression data was carried out using the Whole Human Genome Microarray 4×44K (Agilent). Generation, pre-processing and differential expression and pathway based analyses from this dataset (GSE44805) has been previously reported [8].

Expression profiling in healthy skin tissue was obtained using Illumina Human HT-12 V3 BeadChips (Illumina) in 21 of 705 female individuals from the TwinsUK Cohort (http://www.muther.ac.uk/public.html). The only eligibility criterion for the TwinsUK Cohort was twin status and therefore the sample is held to be representative of the general population [27, 28]. For the present study, individuals were selected based on phenotypical data since the genetic status of the *MC1R* gene was not available. Fourteen red hair individuals (two monozygotic twin pairs, one dizygotic twin pair, and 8 singletons) and 7 with black hair (all singletons) of Caucasian ancestry were selected. The mean age for the RHC individuals was 50.88 years old (range=43.33-59.84), while for the BHC individuals was 62.04 years old (range=49.23-74.54). All red hair individuals but two (missing the *MC1R* genotype) carry at least one RHC allele. For the pair without genetic data, multiple photographs taken in different years were used in order to confirm that they actually had red hair. Non RHC alleles were detected in black hair individuals. Probe expression levels were log transformed and quantile normalized, and only probes mapping uniquely to genes of known function and not containing common SNPs were included in the analysis, as detailed elsewhere [28], resulting in 16,646 genes. Mean expression profile was used when the same gene was targeted by multiple probes. Written informed consent was provided by all the twins, and the Guy's and St Thomas’ Hospital NHS Trust Research Ethics Committee approved the study.

Generation, pre-processing and analysis of whole genome gene expression in melanoma tumors from the Leeds Melanoma Cohort (LMC, ethical approval MREC 1/3/57, PIAG 3-09(d)/2003) has been described elsewhere [29]. This data contains 204 melanoma primaries expression-profiled using DASL array HT12 v4. Gene expression was compared between 20 melanoma tumors from RHC and 6 melanoma tumors from BHC patients. All RHC melanoma patients carried at least one RHC allele). One patient has missing MC1R genotype. We observed that among black haired individuals, one patient carried two RHC variants. One patient was missing their MC1R genotype.

### Statistical analyses

Standardization of gene expression data across arrays from co-cultured keratinocytes and melanocytes was performed with Agilent Processed Signal (Agilent Feature Extraction Software) using quantile normalization [30]. Differential gene expression analysis was carried out using the *limma* [31] package from Bioconductor. Multiple testing adjustment of p-values was performed according to Benjamini and Hochberg (Benjamini & Hochberg, 1995; Benjamini and Yekutieli, 2001) controlling False Discovery Rate (FDR = 5%).

Protein-protein interaction (PPI) networks were built based on differentially expressed genes. Identification of statistically significant PPI networks within the set of differentially expressed genes was carried out by SNOW [32] as implemented in Babelomics 5 suite [33], where approaches for protein-protein interaction data from 5 databases such as IntAct Molecular Interaction Database [34], Molecular INTeraction database [35], Biomolecular Interaction Network Database [36], Database of Interacting Proteins [37] and Human Protein Reference database [38] are included for functional profiling of genomic data, evaluating the cooperative behavior of a list of genes as a functional module. SNOW calculates the Minimal connected network (MCN) for the proteins/genes in the list. The topology of this MCN is then compared against 10,000 random MCNs with same size range to obtain a p-value. Statistically significant connected networks of differentially expressed genes were obtained for up and down regulated transcripts in RHC co-cultured melanocytes and keratinocytes. Further visualization and evaluation of networks was conducted using CytoscapeWeb [39]. Networks were evaluated using two topological parameters, namely degree and betweenness centrality. Degree centrality counts the number of edges connected to each node (protein/gene). Betweenness centrality counts the number of times each node is included in the shortest path between any two other nodes, indicating which node(s) have the larger influence on the communication between proteins/genes included in the network. Nodes were classified based on the mean (μ) and standard deviation (σ) of degree centrality value calculated over all proteins/genes in: lower connected node (degree<μ+2σ), medium connected node (μ+2σ>degree<μ+3σ), and highly connected node (degree>μ+3σ). Highly connected nodes or those not highly connected nodes but with high betweenness centrality values were classify as top nodes (hub genes).

Differential gene expression analysis in healthy skin tissue was carried out using the limma R package controlling for family structure and batch effects. Benjamini and Hochberg's method, as implemented in *limma*, was used to control the false discovery rate at 10%. Differential expression of the hub genes identified in the gene expression data from co-cultured melanocytes and keratinocytes was assessed firstly by analysing the whole gene expression dataset and then by restricting it to hub genes only.

Whole differential gene expression was evaluated by SNOW following the same analyses as in co-cultured melanocytes-keratinocytes data.

Differential expression analysis in tumors was restricted to the genes that were identified as hub genes in the melanocytes-keratinocytes dataset (N=23). Examination of the probe list in the Illumina DASL whole genome array confirmed that 33 probes were arrayed covering all 23 genes selected as hub genes in networks. Differential expression in tumors from 20 RHC vs 6 BHC patients from LMC was performed using linear regression and adjusting for potential batch effect. Analysis was conducted using STATA v12 software.

## SUPPLEMENTARY MATERIALS FIGURES AND TABLES






