# Supplementary material for: Genomic expression differences between cutaneous cells from red hair color individuals and black hair color individuals based on bioinformatic analysis
Source: Oncotarget. 2016 Dec 24;8(7):11589–99. doi: 10.18632/oncotarget.14140 (PMC5355288; doi:10.18632/oncotarget.14140)
Supplement: Supplementary file 1 [file oncotarget-08-11589-s001.pdf]

Genomic expression differences between cutaneous cells from red hair color individuals and black hair color individuals based on bioinformatic analysis

SUPPLEMENTARY TABLES

Supplementary Table 1: Atributes of nodes from networks of up-regulated genes (N=557)

See Supplementary File 1

Supplementary Table 2: Atributes of nodes from networks of down-regulated genes (N=450)

See Supplementary File 1

Supplementary Table 3: First connections of the *ATG4C* gene: Table shows the first connections of the *ATG4C* gene within the network of up-regulated transcripts. Name gene and KEGGKEGG pathway in which gene is associated with, is indicated. NA= Not associated KEGGKEGG pathway.

See Supplementary File 1

Supplementary Table 4: First connections of the *ATG10*: Table shows the first connections of the *ATG10* gene within the network of up-regulated transcripts. Name gene and KEGG pathway in which gene is associated with, is indicated. NA= Not associated KEGG pathway.

See Supplementary File 1

Supplementary Table 5: First connection of *BRCA1*: Table shows the first connections of *BRCA1* gene within the network of up-regulated transcripts. Name gene and KEGG pathway in which gene is associated with, is indicated. NA= Not associated KEGG pathway.

See Supplementary File 1

Supplementary Table 6: First connections of the *BUB1B*

GEN	KEGG
ANAPC16	NA
ANAPC4	HTLV-I infection (hsa05166), Progesterone-mediated oocyte maturation (hsa04914), Oocyte meiosis (hsa04114), Ubiquitin mediated proteolysis (hsa04120), Cell cycle (hsa04110)
AP2B1	Endocytosis (hsa04144), Synaptic vesicle cycle (hsa04721), Endocrine and other factor-regulated calcium reabsorption (hsa04961), Huntington's disease (hsa05016)
BUB1	Progesterone-mediated oocyte maturation (hsa04914), Cell cycle (hsa04110), Oocyte meiosis (hsa04114)
BUB3	Cell cycle (hsa04110), HTLV-I infection (hsa05166)
CDC20	HTLV-I infection (hsa05166), Pyrimidine metabolism (hsa00240), Metabolic pathways (hsa01100), Oocyte meiosis (hsa04114), Viral carcinogenesis (Viral carcinogenesis (hsa05203)), Cell cycle (hsa04110), Ubiquitin mediated proteolysis (hsa04120)
CDC27	Oocyte meiosis (hsa04114), Ubiquitin mediated proteolysis (hsa04120), Cell cycle (hsa04110), Ubiquinone and other terpenoid-quinone biosynthesis (hsa00130), HTLV-I infection (hsa05166), Progesterone-mediated oocyte maturation (hsa04914)
CENPE	Pathways in cancer (hsa05200), HTLV-I infection (hsa05166), Signaling pathways regulating pluripotency of stem cells (hsa04550), Hippo signaling pathway (hsa04390), Wnt signaling pathway (hsa04310), Basal cell carcinoma (hsa05217), Melanogenesis (hsa04916), Notch signaling pathway (hsa04330)
MAD2L1	HTLV-I infection (hsa05166), Progesterone-mediated oocyte maturation (hsa04914), Cell cycle (hsa04110), Oocyte meiosis (hsa04114)
MIS12	NA
PARP1	NF-kappa B signaling pathway (hsa04064), Purine metabolism (hsa00230), Metabolic pathways (hsa01100), Base excision repair (hsa03410)
UBE2C	Ubiquitin mediated proteolysis (hsa04120)

Table shows the first connections of the *BUB1B* gene within the network of up-regulated transcripts. Name gene and KEGG pathway in which gene is associated with, is indicated. NA= Not associated KEGG pathway**.**

Supplementary Table 7: First connection of *CDK1*. Table shows the first connections of the *CDK1* gene within the network of up-regulated transcripts. Name gene and KEGG pathway in which gene is associated with, is indicated. NA= Not associated KEGG pathway.

See Supplementary File 1

Supplementary Table 8: First connections of the *GBAS* gene. Table shows the first connections of the *GBAS* gene within the network of up-regulated transcripts. Name gene and KEGG pathway in which gene is associated with, is indicated. NA= Not associated.

See Supplementary File 1

Supplementary Table 9: First connections of the *ICT1* gene. Table shows the first connections of the *ICT1* gene within the network of up-regulated transcripts. Name gene and KEGG pathway in which gene is associated with, is indicated. NA= Not associated KEGG pathway.

See Supplementary File 1

Supplementary Table 10: First connectios of *PCNA*. Table shows the first connections of the *PCNA* gene within the network of up-regulated transcripts. Name gene and KEGG pathway in which gene is associated with, is indicated. NA= Not associated KEGG pathway.

See Supplementary File 1

Supplementary Table 11: First connections of the *PIK3C3*. Table shows the first connections of the *PIK3C3* gene within the network of up-regulated transcripts. Name gene and KEGG pathway in which gene is associated with, is indicated. NA= Not associated KEGG pathway.

See Supplementary File 1

Supplementary Table 12: First connections of the *PRKAA1*. Table shows the first connections of the *PRKAA1* gene within the network of up-regulated transcripts. Name gene and KEGG pathway in which gene is associated with, is indicated. NA= Not associated KEGG pathway.

See Supplementary File 1

Supplementary Table 13: First connections of the SNX2

GEN	KEGG
FNBP1	NA
SNX4	Endocytosis (hsa04144)
SNX6	Endocytosis (hsa04144)

Table shows the first connections of the *SNX2* gene within the network of up-regulated transcripts. Name gene and KEGG pathway in which gene is associated with, is indicated. NA= Not associated KEGG pathway.

Supplementary Table 14: First connections of the *RPA1*. Table shows the first connections of the *RPA1* gene within the network of up-regulated transcripts. Name gene and KEGG pathway in which gene is associated with, is indicated. NA= Not associated KEGG pathway.

See Supplementary File 1

Supplementary Table 15: First connections of the *GABARPAL1*. Table shows the first connections of the *GABARPAL1* gene within the network of down-regulated transcripts. Name gene and KEGG pathway in which gene is associated with, is indicated. NA= Not associated KEGG pathway.

See Supplementary File 1

Supplementary Table 16: First connection of *CLN3*. Table shows the first connections of the *CLN3* gene within the network of down-regulated transcripts. Name gene and KEGG pathway in which gene is associated with, is indicated. NA= Not associated KEGG pathway.

See Supplementary File 1

Supplementary Table 17: First connections of the *GABARAPL2*. Table shows the first connections of the *GABARAPL2* gene within the network of up-regulated transcripts. Name gene and KEGG pathway in which gene is associated with, is indicated. NA= Not associated KEGG pathway.

See Supplementary File 1

Supplementary Table 18: First connections of the *MAP1LC3A*. Table shows the first connections of the *MAP1LC3A* gene within the network of down-regulated transcripts. Name gene and KEGG pathway in which gene is associated with, is indicated. NA= Not associated KEGG pathway.

See Supplementary File 1

Supplementary Table 19: First connections of the *MAPL1C3B*. Table shows the first connections of the *MAPL1C3B* gene within the network of down-regulated transcripts. Name gene and KEGG pathway in which gene is associated with, is indicated. NA= Not associated KEGG pathway.

See Supplementary File 1

Supplementary Table 20: First connections of the *PABPC1*. Table shows the first connections of the *PABPC1* gene within the network of down-regulated transcripts. Name gene and KEGG pathway in which gene is associated with, is indicated. NA= Not associated KEGG pathway.

See Supplementary File 1

Supplementary Table 21: First connections of the *SMAD3*. Table shows the first connections of the *SMAD3* gene within the network of down-regulated transcripts. Name gene and KEGG pathway in which gene is associated with, is indicated. NA= Not associated KEGG pathway.

See Supplementary File 1

Supplementary Table 22: First connections of the *SQSTM1*. Table shows the first connections of the *SQSTM1* gene within the network of down-regulated transcripts. Name gene and KEGG pathway in which gene is associated with, is indicated. NA= Not associated KEGG pathway.

See Supplementary File 1

Supplementary Table 23: First connections of the *TRAF2*. Table shows the first connections of the *TRAF2* gene within the network of down-regulated transcripts. Name gene and KEGG pathway in which gene is associated with, is indicated. NA= Not associated KEGG pathway.

See Supplementary File 1

Supplementary Table 24: First connections of the *WIPI2*. Table shows the first connections of the *WIPI2* gene within the network of down-regulated transcripts. Name gene and KEGG pathway in which gene is associated with, is indicated. NA= Not associated KEGG pathway.

See Supplementary File 1

Supplementary Table 25: First connections of the *YWHAG*. Table shows the first connections of the *YWHAG* gene within the network of down-regulated transcripts. Name gene and KEGG pathway in which gene is associated with, is indicated. NA= Not associated KEGG pathway.

See Supplementary File 1

Supplementary Table 26: List of differentially expressed genes in healthy skin from Red hair color individuals vs Black hair color individuals. Table contains Log2 Fold Change (logFc), P-Value and adjusted P-value (adj.P-Val) for each gene.

See Supplementary File 2
