# Supplementary material for: Genomic expression differences between cutaneous cells from red hair color individuals and black hair color individuals based on bioinformatic analysis
Source: Oncotarget. 2016 Dec 24;8(7):11589–99. doi: 10.18632/oncotarget.14140 (PMC5355288; doi:10.18632/oncotarget.14140)
Supplement: Supplementary file 2 [file oncotarget-08-11589-s002.doc]

**SUPPLEMENTARY DATA**

**Table 1.** Atributes of nodes from networks of up-regulated genes (N=557).

| Canonical  Name | Betweenness Centrality | Closeness  Centrality | Degree | Eccentricity |
| --- | --- | --- | --- | --- |
| AASS | 0 | 0.21864261 | 1 | 8 |
| ABCB7 | 0 | 0.21864261 | 1 | 8 |
| ABCF2 | 0 | 0.25956145 | 1 | 7 |
| ACAT1 | 0 | 0.28121547 | 1 | 7 |
| ACOT9 | 0 | 0.21864261 | 1 | 8 |
| ACTL6A | 1.56E-04 | 0.24400767 | 3 | 8 |
| ADSL | 0 | 0.28121547 | 1 | 7 |
| AGL | 0 | 0.28121547 | 1 | 7 |
| AHI1 | 0 | 0.20238569 | 1 | 8 |
| AKR1B1 | 0 | 0.28121547 | 1 | 7 |
| AKR1C3 | 0 | 0.20015729 | 1 | 9 |
| ALDH6A1 | 0 | 0.18178571 | 1 | 9 |
| ALDH7A1 | 2.78E-04 | 0.29303397 | 2 | 7 |
| ANAPC10 | 0.00392927 | 0.23284538 | 4 | 7 |
| ANAPC16 | 0 | 0.24541948 | 4 | 7 |
| ANAPC4 | 3.41E-04 | 0.24565637 | 5 | 7 |
| ANP32E | 0 | 0.28121547 | 1 | 7 |
| AP2B1 | 0.01537514 | 0.29610239 | 6 | 6 |
| APLF | 0.00503778 | 0.27117741 | 5 | 6 |
| ASCC3 | 0 | 0.21696505 | 1 | 8 |
| ASF1B | 0.00469637 | 0.2357573 | 4 | 7 |
| ASPM | 0 | 0.20238569 | 1 | 8 |
| ATF2 | 6.61E-04 | 0.2463698 | 2 | 7 |
| ATG10 | 0.11402764 | 0.34886909 | 50 | 6 |
| ATG3 | 0.00335117 | 0.28805886 | 12 | 7 |
| ATG4C | 0.08956554 | 0.35103448 | 42 | 6 |
| ATM | 0.00982336 | 0.2853139 | 15 | 7 |
| ATP5C1 | 0.05038267 | 0.3225602 | 11 | 6 |
| ATP5F1 | 0 | 0.21864261 | 1 | 8 |
| ATP5L | 0 | 0.21864261 | 1 | 8 |
| AURKA | 0.02441993 | 0.28724605 | 9 | 6 |
| AURKB | 0.01005111 | 0.27204703 | 6 | 7 |
| BAG2 | 1.35E-04 | 0.27618014 | 3 | 7 |
| BARD1 | 0.03280053 | 0.30315664 | 13 | 7 |
| BIRC3 | 5.13E-04 | 0.22044175 | 2 | 7 |
| BIRC5 | 0.00513014 | 0.26183128 | 4 | 7 |
| BLM | 0.00696468 | 0.29404968 | 18 | 6 |
| BNIP1 | 0 | 0.1892897 | 1 | 9 |
| BNIP3 | 0 | 0.1892897 | 1 | 9 |
| BORA | 8.65E-04 | 0.23696462 | 2 | 7 |
| BRCA1 | 0.05165136 | 0.32113565 | 27 | 6 |
| BRIX1 | 0 | 0.26183128 | 2 | 7 |
| BTF3 | 7.20E-04 | 0.26441558 | 2 | 7 |
| BUB1 | 0.00700351 | 0.23094374 | 9 | 7 |
| BUB1B | 0.04412561 | 0.29019384 | 12 | 6 |
| BUB3 | 0.00848479 | 0.31517028 | 4 | 6 |
| C13orf34 | 8.65E-04 | 0.23696462 | 2 | 7 |
| C14orf156 | 0 | 0.21864261 | 1 | 8 |
| C16orf59 | 0 | 0.21120332 | 1 | 8 |
| C17orf42 | 0 | 0.21864261 | 1 | 8 |
| C1QBP | 0.03721253 | 0.336863 | 5 | 6 |
| C3orf26 | 0 | 0.25956145 | 1 | 7 |
| C3orf63 | 0 | 0.15207649 | 1 | 10 |
| C4orf27 | 0 | 0.21007016 | 1 | 8 |
| C7orf36 | 0 | 0.22642349 | 1 | 8 |
| CA12 | 0 | 0.24400767 | 1 | 7 |
| CACYBP | 0.00611942 | 0.26510417 | 3 | 7 |
| CASC5 | 0 | 0.1955436 | 3 | 8 |
| CBX3 | 0.00895489 | 0.25260546 | 7 | 7 |
| CBX5 | 0.02242152 | 0.27074468 | 11 | 6 |
| CCBL2 | 5.58E-04 | 0.29052511 | 3 | 7 |
| CCDC109B | 0 | 0.21761437 | 1 | 8 |
| CCDC53 | 0 | 0.17069081 | 1 | 9 |
| CCDC59 | 0 | 0.22835352 | 1 | 7 |
| CCDC90B | 0.0083312 | 0.2415757 | 4 | 8 |
| CCDC99 | 0 | 0.22642349 | 1 | 8 |
| CCNA2 | 0.01136295 | 0.30773881 | 13 | 6 |
| CCNB1 | 0.0068339 | 0.2861158 | 10 | 6 |
| CCNB1IP1 | 0 | 0.25929699 | 2 | 7 |
| CCNB2 | 5.16E-04 | 0.26959746 | 3 | 6 |
| CCNE1 | 0.00724212 | 0.28627672 | 8 | 7 |
| CCNF | 0 | 0.25929699 | 2 | 7 |
| CCNH | 0.00392927 | 0.21288164 | 2 | 8 |
| CCT2 | 0.00540249 | 0.30533893 | 7 | 7 |
| CCT6A | 0.00263309 | 0.30171903 | 6 | 7 |
| CD320 | 0 | 0.21404542 | 1 | 8 |
| CDC20 | 0.03035142 | 0.29575828 | 12 | 6 |
| CDC25A | 1.10E-04 | 0.24565637 | 3 | 7 |
| CDC25C | 0.00393424 | 0.2857945 | 5 | 6 |
| CDC27 | 0.02607278 | 0.28324986 | 12 | 6 |
| CDC45 | 0.00200233 | 0.2918578 | 10 | 7 |
| CDC6 | 0.00308756 | 0.2849944 | 12 | 7 |
| CDC7 | 9.34E-04 | 0.27439353 | 10 | 6 |
| CDCA2 | 0 | 0.25642317 | 1 | 7 |
| CDCA5 | 0 | 0.18036853 | 1 | 8 |
| CDCA8 | 0.00464788 | 0.22413034 | 5 | 7 |
| CDK1 | 0.08397531 | 0.34461747 | 27 | 6 |
| CDKN1C | 0.00103752 | 0.27161153 | 5 | 7 |
| CDKN2C | 0 | 0.19464627 | 1 | 9 |
| CDKN3 | 0 | 0.25642317 | 1 | 7 |
| CDT1 | 0.02570314 | 0.32113565 | 16 | 6 |
| CENPA | 0.00136683 | 0.29002849 | 6 | 6 |
| CENPE | 0.01203087 | 0.22774049 | 4 | 7 |
| CENPH | 0.00130869 | 0.19142535 | 2 | 8 |
| CEP192 | 0 | 0.22324561 | 1 | 7 |
| CEP70 | 3.52E-04 | 0.22774049 | 2 | 8 |
| CEP72 | 0.00428163 | 0.22794447 | 3 | 8 |
| CHAF1A | 0.02145286 | 0.30225653 | 12 | 6 |
| CHAF1B | 3.61E-04 | 0.23818437 | 3 | 7 |
| CHD1L | 0.00892315 | 0.27633008 | 9 | 6 |
| CHEK1 | 0.00515166 | 0.30047226 | 9 | 7 |
| CHTF18 | 0.00894335 | 0.26945474 | 7 | 7 |
| CKAP5 | 0.00289939 | 0.24307545 | 3 | 7 |
| CNPY2 | 0 | 0.21705757 | 1 | 8 |
| COPS3 | 0 | 0.20800981 | 3 | 8 |
| COPS4 | 2.55E-04 | 0.21173045 | 4 | 7 |
| COPS5 | 0.01725968 | 0.26223596 | 10 | 7 |
| COPS8 | 0 | 0.20800981 | 3 | 8 |
| CPSF2 | 0.00334054 | 0.28183832 | 2 | 7 |
| CSE1L | 0.00269745 | 0.30552221 | 4 | 7 |
| DARS2 | 0 | 0.28121547 | 1 | 7 |
| DBF4 | 0.00543974 | 0.27380312 | 9 | 6 |
| DCI | 0 | 0.21864261 | 1 | 8 |
| DDB2 | 8.78E-04 | 0.24829268 | 3 | 7 |
| DEK | 5.72E-04 | 0.27753544 | 3 | 6 |
| DHX29 | 0 | 0.25956145 | 1 | 7 |
| DLAT | 0.01026481 | 0.29069103 | 3 | 6 |
| DLD | 0.02519269 | 0.32052897 | 5 | 6 |
| DLEU1 | 0.00689622 | 0.23851921 | 4 | 8 |
| DLGAP5 | 0 | 0.25642317 | 1 | 7 |
| DNAJC10 | 0 | 0.2587697 | 1 | 7 |
| DNAJC9 | 0 | 0.24684772 | 1 | 7 |
| DNM1L | 0.00474189 | 0.28277778 | 2 | 7 |
| DOCK11 | 0 | 0.25995914 | 1 | 7 |
| DOCK5 | 3.39E-06 | 0.23423838 | 2 | 8 |
| DPM1 | 0.00241846 | 0.2687434 | 2 | 7 |
| DSC1 | 0 | 0.16977985 | 1 | 9 |
| DSCC1 | 0 | 0.24215033 | 2 | 7 |
| DSN1 | 9.83E-04 | 0.22025097 | 6 | 7 |
| DTL | 0.01174141 | 0.28215078 | 2 | 7 |
| DUT | 0.00863794 | 0.26510417 | 3 | 7 |
| DYNC1LI1 | 0.00171242 | 0.28822197 | 3 | 6 |
| DZIP3 | 0 | 0.1977467 | 1 | 8 |
| EBNA1BP2 | 6.41E-04 | 0.23919173 | 2 | 7 |
| ECI1 | 0 | 0.21864261 | 1 | 8 |
| ECT2 | 0 | 0.22979684 | 1 | 7 |
| EIF2S2 | 0.02088994 | 0.31036585 | 5 | 7 |
| ELP4 | 0 | 0.19013821 | 1 | 9 |
| ENSG00000115128 | 0.00451783 | 0.30773881 | 5 | 7 |
| ENSG00000132356 | 0.15791641 | 0.3503097 | 54 | 6 |
| ENSG00000197065 | 5.22E-04 | 0.21668795 | 2 | 8 |
| EPOR | 0 | 0.22979684 | 1 | 7 |
| EPRS | 2.78E-04 | 0.29303397 | 2 | 7 |
| ERLIN2 | 0 | 0.21864261 | 1 | 8 |
| ESD | 0 | 0.28121547 | 1 | 7 |
| ESPL1 | 0 | 0.18107435 | 1 | 8 |
| EXO1 | 0 | 0.25411882 | 3 | 7 |
| EXOSC1 | 0 | 0.16092317 | 1 | 10 |
| EXOSC2 | 0 | 0.25956145 | 1 | 7 |
| EXOSC8 | 0.00392927 | 0.19171375 | 2 | 9 |
| EXOSC9 | 0 | 0.25956145 | 1 | 7 |
| EZH2 | 1.13E-04 | 0.23316537 | 2 | 7 |
| FAM175A | 4.33E-05 | 0.2568113 | 3 | 7 |
| FANCA | 0.01830682 | 0.29905993 | 15 | 6 |
| FANCB | 0 | 0.23104857 | 4 | 7 |
| FANCC | 4.54E-04 | 0.26441558 | 5 | 7 |
| FANCD2 | 0.01004816 | 0.25655242 | 9 | 7 |
| FANCE | 1.05E-04 | 0.23413063 | 5 | 7 |
| FANCG | 0.00220794 | 0.2671916 | 8 | 7 |
| FANCI | 0 | 0.22582076 | 2 | 8 |
| FANCL | 0 | 0.23104857 | 4 | 7 |
| FANCM | 0.00233286 | 0.27060074 | 9 | 7 |
| FAR1 | 0 | 0.2587697 | 1 | 7 |
| FARSB | 6.95E-04 | 0.29870892 | 2 | 7 |
| FBXO5 | 0.00247514 | 0.24805068 | 3 | 7 |
| FEN1 | 0.00419504 | 0.31226994 | 8 | 7 |
| FEZ1 | 0.01389588 | 0.26761304 | 11 | 7 |
| FHL1 | 0 | 0.28121547 | 1 | 7 |
| FKBP3 | 0 | 0.25956145 | 1 | 7 |
| FNBP1 | 0 | 0.6 | 1 | 2 |
| FOXM1 | 0 | 0.22265967 | 1 | 8 |
| FXR1 | 8.88E-04 | 0.27031333 | 2 | 7 |
| G3BP1 | 0.01021032 | 0.27693145 | 5 | 7 |
| GART | 6.48E-04 | 0.30100532 | 4 | 7 |
| GBAS | 0.28717873 | 0.39093702 | 77 | 6 |
| GCSH | 0 | 0.21761437 | 1 | 8 |
| GDPD2 | 0 | 0.24400767 | 1 | 7 |
| GEMIN2 | 0.00239929 | 0.24817162 | 5 | 8 |
| GEMIN6 | 1.55E-06 | 0.1990614 | 2 | 9 |
| GGH | 0 | 0.25956145 | 1 | 7 |
| GLUD1 | 0 | 0.21864261 | 1 | 8 |
| GLUD2 | 0.00430772 | 0.18672047 | 2 | 9 |
| GMNN | 0 | 0.26386729 | 2 | 7 |
| GNB5 | 0 | 0.22866128 | 1 | 7 |
| GNL3 | 0 | 0.26183128 | 2 | 7 |
| GNPNAT1 | 0 | 0.28121547 | 1 | 7 |
| GSTM4 | 0 | 0.21761437 | 1 | 8 |
| GTF3C3 | 0 | 0.22403169 | 1 | 7 |
| GTPBP10 | 0 | 0.21864261 | 1 | 8 |
| GTSE1 | 0 | 0.22403169 | 1 | 7 |
| H2AFV | 0 | 0.25995914 | 1 | 7 |
| H2AFZ | 0 | 0.21659574 | 1 | 7 |
| HAT1 | 0.00186928 | 0.28028634 | 3 | 7 |
| HAUS1 | 0.0162632 | 0.27483801 | 10 | 7 |
| HAUS2 | 2.38E-05 | 0.21854873 | 5 | 8 |
| HAUS4 | 2.38E-05 | 0.21854873 | 5 | 8 |
| HAUS6 | 8.59E-04 | 0.22462489 | 6 | 8 |
| HAUS7 | 8.37E-04 | 0.22265967 | 5 | 8 |
| HAUS8 | 0 | 0.21668795 | 4 | 8 |
| HCFC2 | 0 | 0.18075284 | 1 | 9 |
| HDAC6 | 6.78E-04 | 0.25386534 | 2 | 7 |
| HDDC2 | 0 | 0.28121547 | 1 | 7 |
| HIST1H1A | 0.00327572 | 0.27814208 | 5 | 6 |
| HIST1H4L | 0.03110562 | 0.32754183 | 17 | 6 |
| HJURP | 0.00134975 | 0.3038806 | 5 | 6 |
| HNRNPC | 0.01313734 | 0.30351819 | 4 | 7 |
| HPRT1 | 0.00527252 | 0.2853139 | 5 | 7 |
| HSDL2 | 0 | 0.2587697 | 1 | 7 |
| ICT1 | 0.17748861 | 0.27967033 | 54 | 7 |
| IDH3A | 0 | 0.28121547 | 1 | 7 |
| IKBKAP | 0.00936094 | 0.23467036 | 7 | 8 |
| IMMT | 0.02940946 | 0.30736715 | 11 | 6 |
| ING2 | 0.00193225 | 0.2687434 | 4 | 7 |
| INVS | 0 | 0.21705757 | 1 | 8 |
| ISCA1 | 0 | 0.21864261 | 1 | 8 |
| ITGB3BP | 0.00534239 | 0.26579634 | 4 | 7 |
| KCTD3 | 1.94E-04 | 0.19222054 | 2 | 8 |
| KIAA0101 | 0 | 0.26102564 | 1 | 7 |
| KIAA1377 | 0.03799328 | 0.29252874 | 19 | 7 |
| KIF13A | 0 | 0.22979684 | 1 | 7 |
| KIF15 | 6.07E-04 | 0.24805068 | 2 | 6 |
| KIF20A | 0 | 0.21395544 | 1 | 8 |
| KIF20B | 0 | 0.23896714 | 1 | 8 |
| KIF22 | 0 | 0.23521257 | 1 | 7 |
| KIF2A | 0 | 0.25956145 | 1 | 7 |
| KIF2C | 0.00372197 | 0.22652425 | 2 | 7 |
| KIFC1 | 0 | 0.18178571 | 1 | 9 |
| KLHL13 | 0.00194451 | 0.24284351 | 3 | 7 |
| KPNA2 | 0.01352901 | 0.31323077 | 8 | 7 |
| LBR | 0 | 0.21567797 | 2 | 7 |
| LDHB | 3.88E-04 | 0.29627474 | 4 | 7 |
| LIG1 | 0 | 0.26102564 | 1 | 7 |
| LIN9 | 0 | 0.21181856 | 1 | 7 |
| LLPH | 0 | 0.230839 | 1 | 8 |
| LMNB1 | 6.01E-05 | 0.22958954 | 2 | 8 |
| LRP8 | 0 | 0.18893838 | 1 | 8 |
| LRR1 | 0 | 0.21208333 | 2 | 8 |
| LSM3 | 0.00193981 | 0.24307545 | 5 | 8 |
| LSM5 | 4.72E-04 | 0.2414611 | 4 | 8 |
| LSM7 | 0.00840824 | 0.23685435 | 5 | 8 |
| LYAR | 0.0198026 | 0.29994107 | 12 | 7 |
| MAD2L1 | 0.02406022 | 0.30497304 | 8 | 6 |
| MAGEH1 | 0 | 0.23863104 | 1 | 8 |
| MAGOH | 0.00427229 | 0.22532094 | 3 | 8 |
| MCF2 | 0 | 0.21724285 | 1 | 7 |
| MCM10 | 0.00842378 | 0.26902748 | 11 | 7 |
| MCM2 | 0.0130743 | 0.29627474 | 18 | 6 |
| MCM3 | 0.02862731 | 0.33289732 | 17 | 6 |
| MCM4 | 0.01054225 | 0.32420382 | 18 | 6 |
| MCM5 | 0.0048333 | 0.32235592 | 13 | 6 |
| MCM6 | 0.01592427 | 0.32523962 | 15 | 6 |
| MCM8 | 7.90E-06 | 0.25222993 | 3 | 7 |
| MED21 | 0.00392927 | 0.2434242 | 2 | 7 |
| MED30 | 0 | 0.19584456 | 1 | 8 |
| MINA | 0 | 0.25956145 | 1 | 7 |
| MIS12 | 0.01822228 | 0.24272771 | 11 | 7 |
| MKI67 | 0.01216881 | 0.30011792 | 4 | 6 |
| MLH1 | 0.00371824 | 0.28773318 | 10 | 6 |
| MMS22L | 5.45E-04 | 0.22314774 | 2 | 7 |
| MNAT1 | 0 | 0.17557779 | 1 | 9 |
| MPP1 | 0 | 0.24400767 | 1 | 7 |
| MRPL1 | 0 | 0.21864261 | 1 | 8 |
| MRPL11 | 0 | 0.21864261 | 1 | 8 |
| MRPL13 | 0 | 0.21864261 | 1 | 8 |
| MRPL16 | 0 | 0.21864261 | 1 | 8 |
| MRPL18 | 0 | 0.21864261 | 1 | 8 |
| MRPL19 | 0 | 0.21864261 | 1 | 8 |
| MRPL22 | 0 | 0.21864261 | 1 | 8 |
| MRPL24 | 0 | 0.21864261 | 1 | 8 |
| MRPL3 | 0 | 0.21864261 | 1 | 8 |
| MRPL32 | 0 | 0.21864261 | 1 | 8 |
| MRPL39 | 0 | 0.21864261 | 1 | 8 |
| MRPL40 | 0 | 0.21864261 | 1 | 8 |
| MRPL42 | 0 | 0.19013821 | 1 | 9 |
| MRPL45 | 0 | 0.21696505 | 1 | 8 |
| MRPL47 | 0 | 0.21864261 | 1 | 8 |
| MRPL48 | 0 | 0.21864261 | 1 | 8 |
| MRPL50 | 0 | 0.21864261 | 1 | 8 |
| MRPS10 | 0 | 0.21864261 | 1 | 8 |
| MRPS21 | 0 | 0.21864261 | 1 | 8 |
| MRPS22 | 0 | 0.21864261 | 1 | 8 |
| MRPS23 | 0 | 0.21864261 | 1 | 8 |
| MRPS28 | 0 | 0.21864261 | 1 | 8 |
| MRPS33 | 0 | 0.25086249 | 1 | 7 |
| MRRF | 0 | 0.21864261 | 1 | 8 |
| MSH2 | 0.00947481 | 0.31634556 | 11 | 7 |
| MSH6 | 0.00673674 | 0.31169626 | 11 | 7 |
| MTDH | 0 | 0.26183128 | 2 | 7 |
| MTERFD1 | 0 | 0.21864261 | 1 | 8 |
| MTHFD1 | 6.48E-04 | 0.30100532 | 4 | 7 |
| MUTYH | 0 | 0.27248394 | 2 | 7 |
| MYBL2 | 0.00528964 | 0.26860158 | 5 | 6 |
| MYCBP | 0 | 0.21705757 | 1 | 8 |
| NAA38 | 0.00352344 | 0.22188317 | 4 | 7 |
| NAP1L1 | 0.00224492 | 0.31575682 | 4 | 7 |
| NASP | 3.23E-04 | 0.29575828 | 2 | 6 |
| NCAPD2 | 0.00351363 | 0.2630491 | 6 | 6 |
| NCAPG | 0.00364722 | 0.28404018 | 6 | 6 |
| NCAPG2 | 0 | 0.20182395 | 1 | 7 |
| NCAPH | 0.00314622 | 0.28854875 | 5 | 6 |
| NCBP1 | 0.00785081 | 0.23337918 | 3 | 8 |
| NCL | 0.00290807 | 0.32461735 | 7 | 7 |
| NDC80 | 0.01506187 | 0.20573969 | 11 | 8 |
| NDUFA4 | 1.35E-04 | 0.27618014 | 3 | 7 |
| NDUFA9 | 0 | 0.21864261 | 1 | 8 |
| NDUFB3 | 0 | 0.24400767 | 1 | 7 |
| NDUFB9 | 5.61E-04 | 0.24192015 | 3 | 7 |
| NDUFS1 | 0.00471026 | 0.27468969 | 2 | 7 |
| NEK2 | 0.00305503 | 0.22512163 | 2 | 7 |
| NIF3L1 | 0.00767314 | 0.24853516 | 7 | 7 |
| NIPSNAP3A | 4.45E-04 | 0.23952941 | 2 | 7 |
| NLRP2 | 0 | 0.22592099 | 1 | 7 |
| NME1 | 0.01304872 | 0.2773842 | 6 | 6 |
| NME1-NME2 | 0.01090658 | 0.26318511 | 9 | 7 |
| NOC3L | 0 | 0.25956145 | 1 | 7 |
| NOP16 | 0 | 0.25956145 | 1 | 7 |
| NPM1 | 0.06052796 | 0.35946328 | 17 | 6 |
| NSL1 | 1.20E-04 | 0.21770744 | 4 | 7 |
| NUDT21 | 0.00180864 | 0.23125852 | 3 | 8 |
| NUF2 | 4.97E-05 | 0.196753 | 4 | 8 |
| NUP107 | 0.00587457 | 0.28199446 | 4 | 7 |
| NUP155 | 0 | 0.28121547 | 1 | 7 |
| NUP37 | 0 | 0.22015571 | 2 | 8 |
| NUP54 | 0 | 0.2285586 | 1 | 7 |
| NUP85 | 3.87E-06 | 0.22025097 | 3 | 8 |
| OAT | 0 | 0.21864261 | 1 | 8 |
| ORC1 | 0.00275 | 0.28215078 | 14 | 7 |
| ORC3 | 0.00238386 | 0.29473075 | 10 | 7 |
| ORC5 | 0.00227562 | 0.30207715 | 12 | 7 |
| ORC6 | 5.26E-04 | 0.27588076 | 11 | 7 |
| OSBPL6 | 0 | 0.22403169 | 1 | 7 |
| PAICS | 0.00106926 | 0.26210093 | 2 | 7 |
| PARK7 | 0.00169289 | 0.29354095 | 3 | 7 |
| PARP1 | 0.05225644 | 0.34184016 | 19 | 5 |
| PBK | 0.00117889 | 0.26888537 | 2 | 7 |
| PCGF6 | 0 | 0.20174396 | 1 | 8 |
| PCMT1 | 0 | 0.2587697 | 1 | 7 |
| PCNA | 0.05785576 | 0.35298197 | 28 | 6 |
| PDCD5 | 0 | 0.22642349 | 1 | 8 |
| PDK1 | 0.00392927 | 0.2255206 | 2 | 7 |
| PDK3 | 0.00126077 | 0.24792986 | 2 | 7 |
| PFAS | 0.00106771 | 0.30369928 | 3 | 6 |
| PHB2 | 0.00578626 | 0.28953356 | 3 | 7 |
| PIK3C3 | 0.03270153 | 0.33464826 | 30 | 6 |
| PIK3R1 | 0.03602401 | 0.29818395 | 13 | 6 |
| PKMYT1 | 0 | 0.25929699 | 2 | 7 |
| PLAC8 | 3.30E-04 | 0.24180523 | 2 | 8 |
| PLEKHF2 | 0.00392927 | 0.20981039 | 2 | 8 |
| PLK1 | 0.03341072 | 0.28854875 | 17 | 6 |
| PLK1S1 | 0 | 0.18569865 | 1 | 9 |
| PMPCB | 0.01036351 | 0.29941176 | 2 | 6 |
| PNO1 | 0 | 0.24400767 | 1 | 7 |
| POLA1 | 0.00237512 | 0.28773318 | 7 | 6 |
| POLA2 | 0.00926153 | 0.28309232 | 6 | 6 |
| POLD3 | 0 | 0.26102564 | 1 | 7 |
| POLR2H | 0 | 0.20843571 | 1 | 8 |
| POP1 | 0.00857057 | 0.26496616 | 4 | 7 |
| POP5 | 0 | 0.20955126 | 1 | 8 |
| PPIA | 0.00341456 | 0.32358551 | 7 | 7 |
| PRDX3 | 0.00151808 | 0.29766082 | 5 | 7 |
| PRDX4 | 0.01456878 | 0.28773318 | 4 | 6 |
| PRIM1 | 1.16E-05 | 0.26102564 | 3 | 7 |
| PRIM2 | 0 | 0.26089185 | 2 | 7 |
| PRPF38A | 0 | 0.22072853 | 1 | 7 |
| PRPSAP2 | 0 | 0.28121547 | 1 | 7 |
| PRTFDC1 | 1.67E-04 | 0.23136364 | 2 | 8 |
| PSIP1 | 0 | 0.20937886 | 1 | 8 |
| PSMA1 | 0.01761608 | 0.27002653 | 11 | 7 |
| PSMA2 | 0.00303524 | 0.25616507 | 8 | 7 |
| PSMA3 | 0.00189345 | 0.2375175 | 6 | 7 |
| PSMA5 | 0.01271204 | 0.2918578 | 7 | 7 |
| PSMA6 | 0.00307759 | 0.25086249 | 8 | 7 |
| PSMB3 | 1.21E-04 | 0.22794447 | 4 | 7 |
| PSMC1 | 0.00819828 | 0.29575828 | 4 | 7 |
| PSMD12 | 0.00185901 | 0.26817703 | 3 | 7 |
| PSPH | 0 | 0.19085114 | 1 | 8 |
| PTCD3 | 0 | 0.21864261 | 1 | 8 |
| PTPN2 | 0 | 0.20126532 | 1 | 9 |
| PTPRS | 0 | 0.2285586 | 2 | 8 |
| PTTG1 | 0.00392927 | 0.22101607 | 2 | 7 |
| PWP1 | 2.18E-04 | 0.27262989 | 3 | 7 |
| RAD1 | 1.90E-05 | 0.26223596 | 2 | 7 |
| RAD18 | 0 | 0.23521257 | 1 | 8 |
| RAD21 | 0.00797745 | 0.21996543 | 4 | 7 |
| RAD50 | 3.43E-04 | 0.27513514 | 7 | 7 |
| RAD51 | 0.01898297 | 0.30736715 | 11 | 7 |
| RAD51AP1 | 0 | 0.23521257 | 1 | 8 |
| RAD51C | 0 | 0.23521257 | 1 | 8 |
| RAD54B | 0.00143625 | 0.24805068 | 3 | 7 |
| RAN | 0.01755674 | 0.29696616 | 8 | 7 |
| RANGRF | 0 | 0.22907291 | 1 | 8 |
| RARS2 | 0 | 0.22006053 | 2 | 8 |
| RBBP7 | 0.01592976 | 0.30867192 | 9 | 6 |
| RBBP8 | 5.81E-04 | 0.25348606 | 5 | 7 |
| RBBP9 | 0 | 0.21404542 | 1 | 8 |
| RBFA | 0 | 0.21864261 | 1 | 8 |
| RBL1 | 0.0104678 | 0.27219251 | 10 | 7 |
| RBX1 | 0.00284838 | 0.23327223 | 6 | 7 |
| RECQL | 1.32E-04 | 0.24601257 | 2 | 7 |
| REV3L | 0 | 0.23380799 | 1 | 7 |
| RFC2 | 0.00989806 | 0.3179263 | 8 | 6 |
| RFC3 | 0 | 0.26210093 | 3 | 7 |
| RFC4 | 0.00185747 | 0.30279595 | 6 | 7 |
| RFC5 | 0 | 0.26845992 | 4 | 7 |
| RG9MTD1 | 0.01509317 | 0.22207679 | 5 | 8 |
| RIBC2 | 3.91E-04 | 0.2355391 | 2 | 7 |
| RMI1 | 0 | 0.26988335 | 4 | 7 |
| RPA1 | 0.05273707 | 0.34720327 | 25 | 6 |
| RPA3 | 5.52E-04 | 0.27219251 | 5 | 6 |
| RPL10A | 6.95E-04 | 0.29870892 | 2 | 7 |
| RPL23A | 0.01794681 | 0.32544757 | 8 | 7 |
| RPL26L1 | 0 | 0.25956145 | 1 | 7 |
| RPL35A | 7.20E-04 | 0.26441558 | 2 | 7 |
| RPP40 | 0 | 0.20955126 | 1 | 8 |
| RPS15A | 9.58E-04 | 0.30369928 | 3 | 7 |
| RPS21 | 6.25E-04 | 0.28595506 | 2 | 7 |
| RPS24 | 0.00560256 | 0.31112469 | 5 | 7 |
| RRAD | 0 | 0.21724285 | 1 | 7 |
| RRM1 | 0.00420714 | 0.29337176 | 3 | 7 |
| RRM2 | 0 | 0.22692822 | 1 | 8 |
| RSL24D1 | 0 | 0.230839 | 1 | 8 |
| RUVBL1 | 0.01866064 | 0.31381011 | 9 | 7 |
| SAP30 | 0.00162009 | 0.24365725 | 3 | 7 |
| SDHB | 0.00392927 | 0.25903308 | 2 | 7 |
| SDHD | 0 | 0.20582289 | 1 | 8 |
| SEH1L | 0.0019569 | 0.28183832 | 3 | 7 |
| SGK3 | 0 | 0.1840868 | 1 | 8 |
| SGOL1 | 0.00141464 | 0.23242009 | 3 | 7 |
| SGOL2 | 0 | 0.18315941 | 1 | 8 |
| SHMT1 | 0 | 0.19265708 | 1 | 9 |
| SIN3B | 0.00422542 | 0.22130435 | 3 | 8 |
| SIP1 | 0.00239929 | 0.24817162 | 5 | 8 |
| SIRT3 | 0.00504458 | 0.24180523 | 4 | 7 |
| SKA1 | 0.00784308 | 0.18886827 | 4 | 8 |
| SKA2 | 0 | 0.15896315 | 2 | 9 |
| SKA3 | 0 | 0.15896315 | 2 | 9 |
| SKIV2L2 | 6.95E-04 | 0.29870892 | 2 | 7 |
| SKP2 | 0.00532458 | 0.25863821 | 8 | 7 |
| SLC14A2 | 0 | 0.19705768 | 1 | 8 |
| SLC25A12 | 0 | 0.21864261 | 1 | 8 |
| SLC25A4 | 0.02088805 | 0.31772784 | 4 | 6 |
| SLIRP | 0 | 0.21864261 | 1 | 8 |
| SMC2 | 0.00868655 | 0.25273088 | 7 | 6 |
| SMC4 | 0.00601361 | 0.28969835 | 5 | 6 |
| SMUG1 | 0 | 0.23370064 | 1 | 8 |
| SMYD2 | 0.00938071 | 0.25361236 | 7 | 7 |
| SNAPIN | 0.0048151 | 0.2453012 | 4 | 7 |
| SNRPA1 | 0 | 0.20750102 | 1 | 8 |
| SNRPB2 | 0.00492673 | 0.26169666 | 3 | 7 |
| SNRPD3 | 0.01382209 | 0.30424387 | 7 | 7 |
| SNRPE | 0.01865053 | 0.31093464 | 10 | 7 |
| SNRPF | 0.00179598 | 0.24708738 | 5 | 8 |
| SNRPG | 0.00665863 | 0.29575828 | 7 | 7 |
| SNUPN | 0 | 0.22907291 | 1 | 8 |
| SNX2 | 0.66666667 | 1 | 3 | 1 |
| SNX4 | 0 | 0.75 | 2 | 2 |
| SNX5 | 0 | 0.23031674 | 1 | 7 |
| SNX6 | 0 | 0.75 | 2 | 2 |
| SOD1 | 0 | 0.28262077 | 2 | 7 |
| SOX7 | 0.0340944 | 0.2770822 | 16 | 7 |
| SPAG5 | 0.00164885 | 0.25929699 | 2 | 7 |
| SPC24 | 0 | 0.196753 | 4 | 8 |
| SPC25 | 0 | 0.196753 | 4 | 8 |
| SRBD1 | 6.17E-04 | 0.26318511 | 2 | 7 |
| STAG1 | 0 | 0.18036853 | 1 | 8 |
| STAR | 0 | 0.21120332 | 1 | 8 |
| STMN1 | 0 | 0.21705757 | 1 | 8 |
| SUB1 | 0.00464903 | 0.26469059 | 3 | 7 |
| SUPT16H | 0.00251021 | 0.31226994 | 5 | 6 |
| SUV39H1 | 0.0013026 | 0.2495098 | 3 | 7 |
| TACC3 | 5.75E-04 | 0.2125261 | 2 | 7 |
| TAF9 | 0.00200384 | 0.24914342 | 3 | 8 |
| TDP1 | 0 | 0.22207679 | 1 | 8 |
| TFB1M | 0.01459602 | 0.27967033 | 2 | 6 |
| TGDS | 0 | 0.22979684 | 1 | 7 |
| THAP1 | 0 | 0.2285586 | 1 | 7 |
| THOC7 | 0 | 0.19913928 | 1 | 8 |
| TIPIN | 0 | 0.25785208 | 1 | 7 |
| TK1 | 0 | 0.19464627 | 1 | 9 |
| TMPO | 0.00207149 | 0.26959746 | 3 | 7 |
| TOM1L1 | 0.00660457 | 0.17928848 | 3 | 9 |
| TOP2A | 0.00249693 | 0.2865991 | 5 | 6 |
| TOP2B | 0 | 0.26183128 | 2 | 6 |
| TPX2 | 2.45E-04 | 0.24203519 | 2 | 7 |
| TRIP13 | 0.01374132 | 0.30479042 | 7 | 7 |
| TSC22D3 | 0 | 0.1734833 | 1 | 9 |
| TTC27 | 0 | 0.25995914 | 1 | 7 |
| TTF2 | 0.00322443 | 0.23456221 | 3 | 7 |
| TTK | 9.77E-04 | 0.22363796 | 2 | 7 |
| TUBA1B | 7.95E-04 | 0.23532131 | 2 | 7 |
| TXN | 0.02281794 | 0.32503193 | 8 | 7 |
| TXNDC9 | 0.00106334 | 0.25173096 | 4 | 7 |
| UBE2C | 0.00120867 | 0.26142784 | 4 | 6 |
| UBE2N | 0.00781985 | 0.2463698 | 5 | 7 |
| UCHL5 | 0.00741957 | 0.25185552 | 10 | 8 |
| UQCRH | 0 | 0.19479525 | 1 | 8 |
| USP1 | 0.00392927 | 0.20441767 | 2 | 8 |
| USP13 | 0.00151539 | 0.21111572 | 2 | 8 |
| USP45 | 3.72E-04 | 0.26156218 | 2 | 7 |
| VAV3 | 0 | 0.22979684 | 1 | 7 |
| VRK1 | 1.82E-04 | 0.23652416 | 2 | 7 |
| VRK2 | 0 | 0.22907291 | 1 | 8 |
| WDHD1 | 0 | 0.28121547 | 1 | 7 |
| WDR12 | 0 | 0.18075284 | 1 | 9 |
| WDR5 | 0.00785081 | 0.22053726 | 3 | 8 |
| WHSC1 | 0 | 0.18126781 | 1 | 9 |
| WIBG | 0 | 0.18395374 | 1 | 9 |
| WRN | 0.00518271 | 0.29169054 | 8 | 6 |
| WWOX | 1.77E-04 | 0.22763864 | 2 | 8 |
| XRCC4 | 0 | 0.21341719 | 1 | 7 |
| YARS2 | 0.01285118 | 0.25123396 | 2 | 7 |
| YEATS4 | 0 | 0.23952941 | 2 | 8 |
| ZBTB16 | 0.02678934 | 0.27799017 | 11 | 7 |
| ZHX1 | 0.00433856 | 0.25012285 | 3 | 8 |

**Table 2.** Atributes of nodes from networks of down-regulated genes (N=450)

| **canonicalName** | **BetweennessCentrality** | **ClosenessCentrality** | **Degree** | **Eccentricity** |
| --- | --- | --- | --- | --- |
| A2M | 0.01459837 | 0.22282609 | 4 | 9 |
| ABL2 | 0.00206757 | 0.21948608 | 4 | 10 |
| ACTG1 | 0.01258225 | 0.26214834 | 6 | 7 |
| ACVR1 | 0 | 0.13407456 | 1 | 12 |
| ADAMTS1 | 0 | 0.18230325 | 1 | 10 |
| ADAR | 5.92E-04 | 0.28571429 | 5 | 8 |
| ADCY6 | 0 | 0.21750663 | 1 | 8 |
| ADD1 | 0 | 0.23781903 | 1 | 9 |
| AFAP1 | 0 | 0.20843925 | 1 | 9 |
| AGPAT6 | 7.80E-05 | 0.26181354 | 2 | 8 |
| AKAP13 | 0.00781763 | 0.25030525 | 2 | 7 |
| AKAP8L | 0.00491228 | 0.2709848 | 3 | 8 |
| AKIRIN2 | 0 | 0.20377734 | 1 | 8 |
| ANKFY1 | 0 | 0.25481666 | 1 | 9 |
| ANKS1A | 0.0024854 | 0.25737602 | 2 | 8 |
| ANXA11 | 0 | 0.21432305 | 1 | 9 |
| APOE | 0 | 0.18230325 | 1 | 10 |
| ARHGAP17 | 3.32E-04 | 0.21090535 | 2 | 10 |
| ARIH1 | 0 | 0.21843367 | 1 | 10 |
| ATF3 | 0.00995186 | 0.27815468 | 4 | 9 |
| ATG13 | 0.00459903 | 0.312262 | 15 | 7 |
| ATP1B1 | 0 | 0.23892774 | 1 | 8 |
| ATP2A2 | 0.00174321 | 0.26797386 | 3 | 8 |
| ATXN1 | 0.08883996 | 0.27777778 | 19 | 7 |
| ATXN2 | 0.00581588 | 0.27834352 | 2 | 8 |
| AXIN1 | 0.00632662 | 0.24969549 | 6 | 9 |
| BAG3 | 0.03448326 | 0.28591353 | 4 | 8 |
| BAHD1 | 0.00200946 | 0.24521531 | 2 | 8 |
| BAK1 | 0.01206966 | 0.20833333 | 4 | 8 |
| BANP | 0.00253267 | 0.25883838 | 2 | 8 |
| BATF | 3.56E-04 | 0.24260355 | 2 | 9 |
| BATF2 | 0 | 0.21750663 | 1 | 8 |
| BCAR1 | 0.03790113 | 0.27333333 | 8 | 8 |
| BCL2A1 | 5.96E-06 | 0.13562686 | 2 | 11 |
| BCL2L1 | 0.03687698 | 0.21601686 | 6 | 8 |
| BCL3 | 8.13E-04 | 0.24160283 | 3 | 9 |
| BCR | 0.01132282 | 0.25015253 | 5 | 9 |
| BICD2 | 0 | 0.17982456 | 1 | 11 |
| BMF | 0.00243306 | 0.15672783 | 2 | 10 |
| BMPR2 | 0.00487805 | 0.15477539 | 2 | 11 |
| BOK | 0.00243306 | 0.15672783 | 2 | 10 |
| BRAF | 0.00487805 | 0.24698795 | 2 | 8 |
| C10orf10 | 1.87E-04 | 0.22676991 | 2 | 9 |
| C10orf2 | 0 | 0.25982256 | 1 | 8 |
| CALML3 | 0.00213291 | 0.26248399 | 2 | 8 |
| CAMK2G | 7.80E-05 | 0.26181354 | 2 | 8 |
| CBL | 0.06721989 | 0.2973169 | 14 | 8 |
| CCDC130 | 0 | 0.2420307 | 1 | 8 |
| CCND1 | 0.00871624 | 0.25513379 | 7 | 9 |
| CCNDBP1 | 2.84E-04 | 0.20357498 | 2 | 10 |
| CCNK | 0.00188077 | 0.22539857 | 2 | 8 |
| CD24P4 | 0 | 0.20068527 | 1 | 10 |
| CD36 | 0 | 0.20068527 | 1 | 10 |
| CD3EAP | 0 | 0.19533111 | 1 | 9 |
| CDC42EP1 | 0 | 0.21432305 | 1 | 9 |
| CDCP1 | 0 | 0.20843925 | 1 | 9 |
| CDKN1A | 0.00221284 | 0.24404762 | 4 | 9 |
| CFLAR | 0.02076464 | 0.24683925 | 3 | 8 |
| CFTR | 0.04356229 | 0.28452464 | 14 | 8 |
| CGNL1 | 0 | 0.24669073 | 1 | 8 |
| CHERP | 1.66E-05 | 0.25657071 | 2 | 9 |
| CIC | 0 | 0.21750663 | 1 | 8 |
| CLIP2 | 0 | 0.19570406 | 1 | 10 |
| CLN3 | 0.06342269 | 0.31369549 | 30 | 7 |
| CLU | 8.25E-04 | 0.25355597 | 2 | 9 |
| CNNM3 | 0 | 0.23892774 | 1 | 8 |
| COL18A1 | 0 | 0.23617512 | 1 | 9 |
| CRNN | 0 | 0.25982256 | 1 | 8 |
| CRTC2 | 0 | 0.24669073 | 1 | 8 |
| CRTC3 | 0 | 0.24669073 | 1 | 8 |
| CRYAB | 0.00973224 | 0.18287244 | 2 | 10 |
| CSDA | 0.00325691 | 0.29517639 | 11 | 8 |
| CSK | 0.01090788 | 0.26098027 | 6 | 9 |
| CTDP1 | 0 | 0.162763 | 1 | 11 |
| CTSA | 0 | 0.2581864 | 2 | 9 |
| CTSB | 2.60E-04 | 0.27929155 | 3 | 8 |
| CTSD | 6.55E-05 | 0.26920552 | 2 | 8 |
| CXXC5 | 0 | 0.24117647 | 1 | 9 |
| CYP2S1 | 0 | 0.24419297 | 1 | 9 |
| DAB2 | 0.01891033 | 0.26520052 | 5 | 9 |
| DAPK1 | 4.72E-04 | 0.20971867 | 2 | 9 |
| DBP | 0 | 0.23335231 | 1 | 9 |
| DDIT3 | 0.03209001 | 0.3041543 | 17 | 8 |
| DDX24 | 0 | 0.25965801 | 2 | 9 |
| DFFA | 0.00781763 | 0.25030525 | 2 | 7 |
| DIP2B | 0 | 0.25982256 | 1 | 8 |
| DLC1 | 0 | 0.18859246 | 1 | 10 |
| DMPK | 0 | 0.21750663 | 1 | 8 |
| DNAJA1 | 0.03347259 | 0.32852564 | 17 | 7 |
| DNAJB2 | 0 | 0.25625 | 1 | 8 |
| DOK4 | 0 | 0.20993344 | 1 | 10 |
| DTX3L | 0.01183313 | 0.24346793 | 4 | 9 |
| DUSP6 | 0 | 0.19078641 | 1 | 11 |
| DVL3 | 0.01351686 | 0.24061033 | 7 | 8 |
| DYNLRB1 | 0.02108878 | 0.24317912 | 3 | 7 |
| ECM1 | 0 | 0.17935258 | 1 | 10 |
| EHD1 | 0.00487805 | 0.20654912 | 2 | 9 |
| EHD3 | 0 | 0.17126149 | 1 | 10 |
| EIF2AK2 | 0.0033554 | 0.28671329 | 6 | 8 |
| EIF4G1 | 5.05E-04 | 0.29475198 | 3 | 7 |
| ELF3 | 0.00487805 | 0.17581475 | 2 | 10 |
| EML3 | 0 | 0.24669073 | 1 | 8 |
| ENSG00000091073 | 0 | 0.21432305 | 1 | 9 |
| ENSG00000099882 | 0 | 0.20408163 | 1 | 10 |
| ENSG00000105663 | 0.00889557 | 0.26214834 | 3 | 8 |
| ENSG00000143674 | 0 | 0.18755718 | 1 | 10 |
| ENSG00000147507 | 0.02571139 | 0.25091799 | 10 | 9 |
| EPDR1 | 0 | 0.26867628 | 2 | 8 |
| EPHB2 | 0 | 0.20448878 | 2 | 10 |
| EPS8 | 0.00188452 | 0.22577093 | 2 | 9 |
| ERBB2 | 0.02699862 | 0.26554404 | 10 | 9 |
| EXTL3 | 0 | 0.20408163 | 1 | 10 |
| FAM134C | 0 | 0.23892774 | 1 | 8 |
| FAM3C | 0 | 0.23892774 | 1 | 8 |
| FAM40A | 0.00487805 | 0.17998244 | 2 | 11 |
| FAM46A | 0.00487805 | 0.21773765 | 2 | 8 |
| FAT1 | 0 | 0.22162162 | 1 | 9 |
| FBXW11 | 0.03177279 | 0.27929155 | 8 | 9 |
| FBXW5 | 0 | 0.20950434 | 1 | 9 |
| FLCN | 0 | 0.2490887 | 1 | 9 |
| FOXO1 | 0.01014329 | 0.27590848 | 3 | 8 |
| FOXO4 | 0 | 0.24117647 | 1 | 9 |
| FTH1 | 0 | 0.19570406 | 1 | 10 |
| FUCA1 | 0 | 0.22078621 | 1 | 9 |
| FURIN | 0 | 0.17779705 | 1 | 10 |
| FYCO1 | 0.04163756 | 0.32283465 | 21 | 8 |
| FZR1 | 0 | 0.24743512 | 1 | 8 |
| GABARAPL1 | 0.05412111 | 0.33144705 | 38 | 8 |
| GABARAPL2 | 0.08625664 | 0.34166667 | 42 | 8 |
| GADD45B | 0 | 0.1754386 | 1 | 11 |
| GADD45G | 0.00675167 | 0.23335231 | 4 | 10 |
| GAK | 0.00147845 | 0.25371287 | 2 | 9 |
| GBP2 | 0 | 0.25261861 | 1 | 8 |
| GDI1 | 1.87E-04 | 0.25949367 | 3 | 7 |
| GGA1 | 0.0048025 | 0.24595081 | 3 | 9 |
| GMEB1 | 0.0019109 | 0.25030525 | 2 | 9 |
| GMEB2 | 0 | 0.21750663 | 1 | 8 |
| GOLGA2 | 0.00974417 | 0.18644839 | 3 | 9 |
| GOLGA3 | 0 | 0.2420307 | 1 | 8 |
| GPC4 | 0 | 0.25982256 | 1 | 8 |
| GRN | 0 | 0.22162162 | 1 | 9 |
| GSK3A | 0.00612207 | 0.25122549 | 3 | 8 |
| GTF2I | 0.02490657 | 0.29390681 | 8 | 8 |
| GTF2IRD1 | 0 | 0.24117647 | 1 | 9 |
| GTPBP1 | 0 | 0.2709848 | 3 | 8 |
| HABP4 | 0 | 0.20854527 | 1 | 10 |
| HERC5 | 0.01059273 | 0.24075161 | 4 | 9 |
| HEY1 | 0.00487805 | 0.24146054 | 2 | 9 |
| HINFP | 0 | 0.2420307 | 1 | 8 |
| HIST1H1C | 0.00839056 | 0.30574198 | 13 | 7 |
| HIST1H2BK | 0.00973224 | 0.21298701 | 2 | 9 |
| HIST1H2BL | 1.66E-05 | 0.2629891 | 3 | 9 |
| HIVEP2 | 0.00182553 | 0.25450031 | 2 | 8 |
| HLA-B | 0.03665294 | 0.30850263 | 13 | 7 |
| HSF1 | 0.00679383 | 0.25689223 | 4 | 9 |
| HSPA2 | 0.00414431 | 0.28913963 | 6 | 8 |
| HSPA6 | 0.00262162 | 0.29223093 | 8 | 8 |
| HSPB8 | 0.01456259 | 0.22318998 | 2 | 9 |
| HTT | 0.01418216 | 0.28611305 | 5 | 8 |
| IGF1R | 0.01542254 | 0.25982256 | 5 | 8 |
| IKBKB | 0.02235282 | 0.30688623 | 8 | 7 |
| INPP5K | 0.00562647 | 0.27224436 | 3 | 8 |
| INPPL1 | 7.86E-04 | 0.23864959 | 2 | 9 |
| ITFG3 | 0 | 0.19294118 | 1 | 10 |
| JUNB | 0.00645287 | 0.26623377 | 4 | 9 |
| KCTD17 | 0 | 0.20377734 | 1 | 8 |
| KCTD5 | 0 | 0.14958045 | 1 | 11 |
| KDM2A | 0 | 0.24117647 | 1 | 9 |
| KDM5A | 9.94E-04 | 0.20780537 | 3 | 10 |
| KIAA0174 | 0 | 0.21750663 | 1 | 8 |
| KIAA0930 | 0 | 0.24669073 | 1 | 8 |
| KIAA1267 | 0.00479011 | 0.26231606 | 2 | 8 |
| KIAA1683 | 1.87E-04 | 0.22676991 | 2 | 9 |
| KIF13B | 0 | 0.22078621 | 1 | 9 |
| KIT | 0.00194527 | 0.24160283 | 4 | 9 |
| KLHDC10 | 0.00244139 | 0.28591353 | 11 | 8 |
| KLHL24 | 0 | 0.24419297 | 1 | 9 |
| KLK13 | 0 | 0.18230325 | 1 | 10 |
| KPNA6 | 6.38E-04 | 0.26485788 | 4 | 9 |
| KRBA1 | 0 | 0.20088192 | 1 | 9 |
| KRT13 | 0.01001394 | 0.31881804 | 14 | 7 |
| KRT19 | 0.00213291 | 0.26248399 | 2 | 8 |
| KRT75 | 0.01292486 | 0.32359905 | 16 | 7 |
| LARP1 | 0.00593573 | 0.29016277 | 7 | 8 |
| LDOC1L | 0 | 0.23335231 | 1 | 9 |
| LMO2 | 6.53E-04 | 0.22066738 | 2 | 10 |
| LNX1 | 0.01385207 | 0.25577043 | 6 | 7 |
| LPXN | 0.00572242 | 0.22007515 | 2 | 9 |
| LRSAM1 | 0.03396308 | 0.27278776 | 4 | 8 |
| LSR | 0 | 0.24669073 | 1 | 8 |
| LTBR | 7.42E-04 | 0.24550898 | 3 | 8 |
| LZTS2 | 0.00126736 | 0.24404762 | 2 | 8 |
| MAFF | 0 | 0.20603015 | 1 | 10 |
| MAP1LC3A | 0.03631602 | 0.33117932 | 31 | 8 |
| MAP1LC3B | 0.04804423 | 0.33551555 | 38 | 8 |
| MAP2K3 | 0.00487805 | 0.17558887 | 2 | 11 |
| MAP3K10 | 0.00587346 | 0.23072594 | 3 | 9 |
| MAP3K4 | 0.01503098 | 0.2126556 | 4 | 10 |
| MAP7D1 | 0 | 0.26762402 | 2 | 8 |
| MAPKAPK2 | 3.76E-04 | 0.22031166 | 2 | 9 |
| MARCKSL1 | 0 | 0.23335231 | 1 | 9 |
| MARK2 | 0.01273868 | 0.28314917 | 5 | 8 |
| MARK4 | 0.00151619 | 0.25625 | 3 | 9 |
| MAX | 0.02832537 | 0.24317912 | 7 | 9 |
| MCL1 | 0.01456855 | 0.18560435 | 4 | 9 |
| MDK | 0 | 0.20780537 | 1 | 8 |
| MED15 | 0.02939887 | 0.26198083 | 5 | 8 |
| MED22 | 0 | 0.17219656 | 1 | 10 |
| MED9 | 0.00487805 | 0.20791075 | 2 | 9 |
| MICALL1 | 0 | 0.24669073 | 1 | 8 |
| MME | 0.00876037 | 0.23892774 | 6 | 9 |
| MMP14 | 0.00582544 | 0.21613073 | 3 | 9 |
| MNT | 0 | 0.19570406 | 1 | 10 |
| MRAS | 0 | 0.19816336 | 1 | 9 |
| MXD1 | 2.69E-04 | 0.19711538 | 2 | 10 |
| MXD4 | 0 | 0.19570406 | 1 | 10 |
| MYH9 | 0.01716457 | 0.27333333 | 9 | 8 |
| MYL9 | 0 | 0.21477213 | 1 | 9 |
| MYO1C | 0.00444073 | 0.23361823 | 2 | 7 |
| NAPA | 0.00973224 | 0.15151515 | 2 | 10 |
| NCOA1 | 0.01965551 | 0.27242525 | 8 | 9 |
| NCOA6 | 0.01866071 | 0.29119318 | 8 | 8 |
| NCOR2 | 0.0078986 | 0.26502909 | 7 | 9 |
| NEBL | 0 | 0.21166753 | 1 | 9 |
| NEDD4L | 0.0019604 | 0.24743512 | 2 | 8 |
| NFKBIB | 0.00561188 | 0.24260355 | 4 | 8 |
| NFKBIE | 1.29E-04 | 0.22841226 | 2 | 9 |
| NLRC5 | 0 | 0.23495702 | 1 | 8 |
| NPEPPS | 0.00979167 | 0.26623377 | 4 | 7 |
| NR2F6 | 0.00487805 | 0.22294725 | 3 | 10 |
| OLFM2 | 0 | 0.17725897 | 1 | 11 |
| OTUB2 | 0 | 0.22727273 | 1 | 9 |
| OTUD5 | 0 | 0.19692603 | 1 | 9 |
| PABPC1 | 0.14489991 | 0.36283186 | 24 | 7 |
| PARD6B | 0 | 0.24893746 | 1 | 9 |
| PARP9 | 0 | 0.19589107 | 1 | 10 |
| PCBP1 | 0.00475743 | 0.31857032 | 13 | 7 |
| PCDH1 | 0 | 0.24117647 | 1 | 9 |
| PDE4A | 0 | 0.20068527 | 1 | 10 |
| PDK4 | 0 | 0.21166753 | 1 | 9 |
| PDPK1 | 0.02952609 | 0.30325444 | 16 | 8 |
| PGLS | 0 | 0.21432305 | 1 | 9 |
| PHF23 | 0 | 0.21443515 | 1 | 9 |
| PHLDA1 | 0 | 0.21432305 | 1 | 9 |
| PHLDA3 | 0.00671117 | 0.21210554 | 4 | 8 |
| PIAS3 | 0.01167926 | 0.26332691 | 4 | 9 |
| PICALM | 0.00359392 | 0.24404762 | 2 | 9 |
| PINK1 | 0 | 0.25137952 | 1 | 9 |
| PITX1 | 0 | 0.19403691 | 1 | 9 |
| PLCG1 | 0.04239955 | 0.25625 | 13 | 9 |
| PLSCR1 | 0.04999808 | 0.27260638 | 13 | 8 |
| PLXNB2 | 0 | 0.20408163 | 1 | 10 |
| PML | 0.02020793 | 0.27498323 | 9 | 8 |
| POM121 | 0.01809922 | 0.22853958 | 3 | 8 |
| PPARG | 0.014242 | 0.25932954 | 7 | 9 |
| PPARGC1A | 4.83E-04 | 0.22828508 | 3 | 10 |
| PPME1 | 0 | 0.16505636 | 1 | 11 |
| PPP1R13L | 0 | 0.22007515 | 1 | 10 |
| PPP2R2D | 0.00487805 | 0.19759036 | 2 | 10 |
| PPRC1 | 0 | 0.18568841 | 2 | 10 |
| PRICKLE1 | 0 | 0.19403691 | 1 | 9 |
| PRKCD | 0.01857728 | 0.26315789 | 6 | 8 |
| PRKD1 | 0 | 0.20408163 | 1 | 10 |
| PRKD2 | 0 | 0.19458946 | 1 | 10 |
| PRRC2B | 0.00132363 | 0.21925134 | 2 | 8 |
| PTK2B | 0.02590257 | 0.2581864 | 7 | 9 |
| PTPRH | 0 | 0.21477213 | 1 | 9 |
| PTPRK | 0.00575386 | 0.26131294 | 5 | 9 |
| QPCT | 1.95E-05 | 0.24969549 | 2 | 9 |
| RAB11B | 0.00730515 | 0.21533613 | 3 | 10 |
| RAB11FIP3 | 1.19E-05 | 0.17741238 | 2 | 11 |
| RAB11FIP4 | 0.0024271 | 0.21511018 | 2 | 10 |
| RAB11FIP5 | 0.02172693 | 0.27351568 | 4 | 9 |
| RAB24 | 0.00145529 | 0.29390681 | 10 | 8 |
| RAB5B | 0 | 0.16970199 | 1 | 11 |
| RAD54L2 | 0.01188285 | 0.27333333 | 2 | 8 |
| RAI14 | 0 | 0.24669073 | 1 | 8 |
| RANGAP1 | 4.09E-04 | 0.27967258 | 3 | 7 |
| RARG | 0 | 0.18238434 | 1 | 11 |
| RELB | 0.00443676 | 0.25593009 | 6 | 8 |
| RERE | 0.00520551 | 0.21843367 | 3 | 9 |
| RGS12 | 0 | 0.20019531 | 1 | 10 |
| RHBDD2 | 0 | 0.23892774 | 1 | 8 |
| RIN3 | 0.00487805 | 0.204285 | 2 | 10 |
| RIPK4 | 0 | 0.20843925 | 1 | 9 |
| RNF10 | 0 | 0.17849369 | 1 | 10 |
| RNF123 | 1.66E-05 | 0.2629891 | 3 | 9 |
| RNF43 | 0 | 0.21331946 | 1 | 9 |
| RPS15A | 0.00376241 | 0.30325444 | 13 | 8 |
| RPTN | 3.70E-04 | 0.28413028 | 7 | 8 |
| RRP1 | 0 | 0.2590019 | 2 | 9 |
| RTN4 | 0.01456259 | 0.1781834 | 2 | 9 |
| RUNX1 | 4.53E-04 | 0.24954352 | 3 | 9 |
| RUNX3 | 0.00733021 | 0.24536206 | 3 | 9 |
| RUSC2 | 0 | 0.15720859 | 1 | 10 |
| S100A7 | 8.65E-04 | 0.26214834 | 2 | 8 |
| S100P | 0 | 0.21477213 | 1 | 9 |
| SAFB2 | 3.60E-05 | 0.26383526 | 4 | 9 |
| SAP30BP | 3.47E-04 | 0.20897044 | 2 | 10 |
| SART1 | 0 | 0.19570406 | 1 | 10 |
| SATB1 | 0 | 0.21578947 | 1 | 9 |
| SCNN1A | 3.36E-04 | 0.22343324 | 2 | 9 |
| SDF4 | 0.00200008 | 0.26147959 | 2 | 8 |
| SEC16A | 1.10E-04 | 0.28295376 | 4 | 8 |
| SERPINB13 | 0 | 0.2490887 | 1 | 9 |
| SETBP1 | 0 | 0.24419297 | 1 | 9 |
| SF1 | 0.01036494 | 0.29202279 | 6 | 8 |
| SGTA | 0 | 0.23590334 | 1 | 8 |
| SH3BP5L | 0 | 0.24669073 | 1 | 8 |
| SH3GL1 | 0.0017704 | 0.25625 | 2 | 9 |
| SHB | 6.79E-04 | 0.21287643 | 2 | 10 |
| SHC2 | 0 | 0.20993344 | 1 | 10 |
| SIRPA | 0 | 0.15162722 | 1 | 11 |
| SKI | 0 | 0.24939173 | 2 | 9 |
| SLC23A3 | 0 | 0.20634122 | 1 | 9 |
| SLC35A2 | 0 | 0.21432305 | 1 | 9 |
| SLC35E1 | 0 | 0.23892774 | 1 | 8 |
| SLC9A3R1 | 0.00229744 | 0.22905028 | 3 | 9 |
| SMAD3 | 0.14742737 | 0.31758327 | 27 | 8 |
| SMARCA4 | 0.05228806 | 0.31178707 | 17 | 8 |
| SMCR7L | 0 | 0.24669073 | 1 | 8 |
| SNCG | 0 | 0.17741238 | 1 | 9 |
| SORT1 | 9.88E-04 | 0.24984765 | 2 | 9 |
| SOX7 | 0.02287808 | 0.26973684 | 4 | 8 |
| SP1 | 0.0171561 | 0.28198074 | 8 | 9 |
| SP110 | 0 | 0.19711538 | 1 | 10 |
| SPHK2 | 0 | 0.20068527 | 1 | 10 |
| SPINT1 | 0 | 0.17888307 | 1 | 9 |
| SPNS1 | 0 | 0.17771998 | 1 | 9 |
| SPRY4 | 0 | 0.22930649 | 1 | 9 |
| SQSTM1 | 0.12141035 | 0.35072712 | 39 | 7 |
| ST6GALNAC6 | 0 | 0.21750663 | 1 | 8 |
| STAC2 | 0 | 0.21750663 | 1 | 8 |
| STAT2 | 2.89E-04 | 0.25465839 | 2 | 9 |
| STAT3 | 0.05112531 | 0.31131359 | 13 | 8 |
| STAT5A | 0 | 0.21421108 | 1 | 10 |
| STK24 | 0 | 0.15258653 | 1 | 12 |
| STK38 | 0.00487805 | 0.1943128 | 2 | 10 |
| STS | 0.00167962 | 0.24818402 | 2 | 9 |
| STX12 | 0.00487805 | 0.13170575 | 2 | 11 |
| STX6 | 0 | 0.11641113 | 1 | 12 |
| SUPT5H | 0.01295732 | 0.24580336 | 3 | 9 |
| SVIL | 0.00110769 | 0.23085586 | 2 | 9 |
| SYMPK | 0 | 0.20448878 | 1 | 10 |
| SYNPO | 0 | 0.24669073 | 1 | 8 |
| TAOK2 | 0 | 0.14941691 | 1 | 12 |
| TCF7 | 0.00130991 | 0.21613073 | 2 | 10 |
| TECPR1 | 0.02335302 | 0.3089676 | 14 | 8 |
| TECPR2 | 1.66E-05 | 0.2629891 | 3 | 9 |
| TFE3 | 0 | 0.24117647 | 1 | 9 |
| TFG | 0.06531531 | 0.33772652 | 18 | 7 |
| THRB | 0.00462467 | 0.24390244 | 6 | 9 |
| TIMP2 | 0.00587846 | 0.21556257 | 3 | 8 |
| TJP3 | 0 | 0.22162162 | 1 | 9 |
| TM4SF1 | 3.29E-05 | 0.26181354 | 2 | 8 |
| TM9SF1 | 9.70E-04 | 0.242891 | 2 | 8 |
| TMEM102 | 0 | 0.24669073 | 1 | 8 |
| TMSB4X | 0 | 0.20780537 | 1 | 8 |
| TNFRSF11A | 0 | 0.24231678 | 2 | 8 |
| TNFRSF14 | 5.74E-04 | 0.26938239 | 3 | 7 |
| TNKS1BP1 | 0 | 0.21068859 | 1 | 9 |
| TNPO3 | 0 | 0.17506405 | 1 | 9 |
| TNS3 | 0.00542595 | 0.23229462 | 3 | 9 |
| TOB1 | 0.00487805 | 0.26675342 | 2 | 8 |
| TP53INP2 | 0 | 0.25481666 | 1 | 9 |
| TPP1 | 0.02202412 | 0.28531663 | 5 | 8 |
| TRAF2 | 0.13543615 | 0.31906615 | 27 | 7 |
| TRAF3 | 0.00520564 | 0.24506874 | 6 | 8 |
| TRAF4 | 4.75E-04 | 0.25277435 | 2 | 8 |
| TRIM11 | 0 | 0.2077001 | 1 | 9 |
| TRIM17 | 0 | 0.17849369 | 1 | 10 |
| TRIM2 | 0.00487805 | 0.17864924 | 2 | 10 |
| TRIM21 | 0.07585156 | 0.336894 | 18 | 8 |
| TRIM56 | 0 | 0.17849369 | 1 | 10 |
| TRIM63 | 0.01094249 | 0.26832461 | 4 | 8 |
| TRIP10 | 0.008032 | 0.25657071 | 4 | 9 |
| TSC2 | 0.01368537 | 0.26485788 | 6 | 8 |
| TTLL12 | 0 | 0.23590334 | 1 | 8 |
| TUBA4A | 0.05768565 | 0.34395973 | 19 | 7 |
| TUBB3 | 7.80E-05 | 0.26181354 | 2 | 8 |
| UBE2H | 0.0247944 | 0.21716102 | 6 | 9 |
| UBE2R2 | 7.19E-04 | 0.25673137 | 2 | 9 |
| ULK1 | 0.00742878 | 0.31881804 | 19 | 8 |
| USF1 | 0.00973224 | 0.22777778 | 3 | 9 |
| USF2 | 0 | 0.18568841 | 2 | 10 |
| USP31 | 0 | 0.2420307 | 1 | 8 |
| VAT1 | 0 | 0.23892774 | 1 | 8 |
| VDR | 0.00539471 | 0.25705329 | 5 | 9 |
| VPS11 | 0.00155282 | 0.21995708 | 2 | 9 |
| VPS18 | 7.85E-04 | 0.20459082 | 2 | 10 |
| WBP2 | 0.00155002 | 0.26885246 | 4 | 9 |
| WDR45L | 0 | 0.2490887 | 1 | 9 |
| WFS1 | 0 | 0.22162162 | 1 | 9 |
| WIPI2 | 0.10525821 | 0.34424853 | 34 | 7 |
| WWC3 | 0.00139187 | 0.21913415 | 2 | 8 |
| WWP2 | 0.00419782 | 0.26147959 | 4 | 9 |
| YTHDF1 | 0 | 0.25982256 | 1 | 8 |
| YWHAG | 0.148895 | 0.32721468 | 29 | 7 |
| ZBTB43 | 0 | 0.24375743 | 2 | 8 |
| ZBTB7A | 0 | 0.20961145 | 1 | 10 |
| ZC3HAV1 | 0 | 0.2490887 | 1 | 9 |
| ZG16B | 0 | 0.23892774 | 1 | 8 |
| ZMIZ2 | 0 | 0.20854527 | 1 | 10 |
| ZNF212 | 0.00704786 | 0.23563218 | 4 | 10 |
| ZNF250 | 0 | 0.15720859 | 1 | 10 |
| ZNF506 | 0 | 0.21578947 | 1 | 9 |
| ZNF581 | 1.77E-04 | 0.22282609 | 2 | 8 |
| ZNF609 | 0 | 0.21750663 | 1 | 8 |
| ZNF646 | 0 | 0.2420307 | 1 | 8 |
| ZNF773 | 0 | 0.21036429 | 1 | 10 |
| ZRANB1 | 0.01458644 | 0.21913415 | 3 | 10 |

**Table 3. First connections of the *ATG4C* gene:** Table shows the first connections of the *ATG4C* gene within the network of up-regulated transcripts . Name gene and KEGGKEGG pathway in which gene is associated with, is indicated. NA= Not associated KEGGKEGG pathway.

| GEN | KEGG |
| --- | --- |
| ATP5C1 | Alzheimer's disease (hsa05010),Metabolic pathways (hsa01100),Parkinson's disease (hsa05012),Oxidative phosphorylation (hsa00190),Huntington's disease (hsa05016),Aminoacyl-tRNA biosynthesis (hsa00970) |
| BAG2 | Protein processing in endoplasmic reticulum (hsa04141) |
| C1QBP | Herpes simplex infection (hsa05168) |
| CCBL2 | Chemical carcinogenesis (hsa05204),Metabolic pathways (hsa01100),Tryptophan metabolism (hsa00380),Selenocompound metabolism (hsa00450) |
| CCT2 | Aminoacyl-tRNA biosynthesis (hsa00970) |
| CCT6A | NA |
| CDC45 | Cell cycle (hsa04110) |
| CDK1 | Cell cycle (hsa04110),Oocyte meiosis (hsa04114),p53 signaling pathway (hsa04115),Gap junction (hsa04540),Progesterone-mediated oocyte maturation (hsa04914),Herpes simplex infection (hsa05168),Epstein-Barr virus infection (hsa05169),Viral carcinogenesis (Viral carcinogenesis (hsa05203)) |
| DLD | Pyruvate metabolism (hsa00620),Citrate cycle (TCA cycle) (hsa00020),Glyoxylate and dicarboxylate metabolism (hsa00630),Carbon metabolism (hsa01200),Glycolysis and Gluconeogenesis (hsa00010),Glycine, serine and threonine metabolism (hsa00260),Valine, leucine and isoleucine degradation (hsa00280),Metabolic pathways (hsa01100),Glutamatergic synapse (hsa04724),Neuroactive ligand-receptor interaction (hsa04080) |
| DOCK11 | Metabolic pathways (hsa01100) |
| DYNC1LI1 | Phagosome (hsa04145),Vasopressin-regulated water reabsorption (hsa04962),Salmonella infection (hsa05132) |
| GART | One carbon pool by folate (hsa00670),Metabolic pathways (hsa01100),Purine metabolism (hsa00230) |
| H2AFV | Systemic lupus erythematosus (hsa05322),Alcoholism (hsa05034) |
| HJURP | NA |
| HPRT1 | Adherens junction (hsa04520),Focal adhesion (hsa04510),Regulation of actin cytoskeleton (hsa04810),Systemic lupus erythematosus (hsa05322),Amoebiasis (hsa05146),Tight junction (hsa04530),Arrhythmogenic right ventricular cardiomyopathy (ARVC) (hsa05412),Leukocyte transendothelial migration (hsa04670),Drug metabolism - other enzymes (hsa00983),Viral carcinogenesis (Viral carcinogenesis (hsa05203)),Purine metabolism (hsa00230),Metabolic pathways (hsa01100) |
| LDHB | Glucagon signaling pathway (hsa04922),Pyruvate metabolism (hsa00620),Propanoate metabolism (hsa00640),MicroRNAs in cancer (hsa05206),Cysteine and methionine metabolism (hsa00270),Metabolic pathways (hsa01100),Glycolysis and Gluconeogenesis (hsa00010) |
| MCM3 | Non-alcoholic fatty liver disease (NAFLD) (hsa04932),Cell cycle (hsa04110),DNA replication (hsa03030),Insulin resistance (hsa04931) |
| MSH6 | NF-kappa B signaling pathway (hsa04064),Pathways in cancer (hsa05200),RNA transport (hsa03013),MicroRNAs in cancer (hsa05206),Ubiquitin mediated proteolysis (hsa04120),Mismatch repair (hsa03430),Colorectal cancer (hsa05210) |
| MTHFD1 | Metabolic pathways (hsa01100),One carbon pool by folate (hsa00670),Glyoxylate and dicarboxylate metabolism (hsa00630) |
| NAP1L1 | NA |
| NCL | Pathogenic Escherichia coli infection (Pathogenic Escherichia coli infection (hsa05130)) |
| NDUFA4 | Huntington's disease (hsa05016),Non-alcoholic fatty liver disease (NAFLD) (hsa04932),Oxidative phosphorylation (hsa00190),Parkinson's disease (hsa05012),Alzheimer's disease (hsa05010),Metabolic pathways (hsa01100) |
| NME1 | Purine metabolism (hsa00230),Metabolic pathways (hsa01100),Transcriptional misregulation in cancer (hsa05202),Pathways in cancer (hsa05200),Pyrimidine metabolism (hsa00240),Thyroid hormone synthesis (hsa04918),Thyroid cancer (hsa05216) |
| NPM1 | NA |
| PCNA | Hepatitis B (hsa05161),HTLV-I infection (hsa05166),Mismatch repair (hsa03430),Base excision repair (hsa03410),Endocytosis (hsa04144),Cell cycle (hsa04110),Nucleotide excision repair (hsa03420),DNA replication (hsa03030) |
| PHB2 | NA |
| PIK3C3 | Tuberculosis (hsa05152),Regulation of autophagy (hsa04140),Metabolic pathways (hsa01100),Phosphatidylinositol signaling system (hsa04070),Arginine and proline metabolism (hsa00330),Inositol phosphate metabolism (hsa00562),Phagosome (hsa04145) |
| PPIA | Notch signaling pathway (hsa04330) |
| PRDX3 | Ubiquitin mediated proteolysis (hsa04120) |
| PSMA5 | Proteasome (hsa03050) |
| PSMC1 | Viral carcinogenesis (Viral carcinogenesis (hsa05203)),Epstein-Barr virus infection (hsa05169),Chemokine signaling pathway (hsa04062),Cytokine-cytokine receptor interaction (hsa04060),Proteasome (hsa03050) |
| PSMD12 | Proteasome (hsa03050),Epstein-Barr virus infection (hsa05169) |
| RAD51 | Homologous recombination (hsa03440),Fanconi anemia pathway (hsa03460),Pathways in cancer (hsa05200),Ubiquitin mediated proteolysis (hsa04120),Pancreatic cancer (hsa05212) |
| RPL23A | Glycosphingolipid biosynthesis - globo series (hsa00603),Metabolic pathways (hsa01100),Glycosphingolipid biosynthesis - lacto and neolacto series (hsa00601),Ribosome (hsa03010) |
| RPS24 | Leukocyte transendothelial migration (hsa04670),Rap1 signaling pathway (hsa04015),Phospholipase D signaling pathway (hsa04072),cAMP signaling pathway (hsa04024),Serotonergic synapse (hsa04726),Adrenergic signaling in cardiomyocytes (hsa04261),Ribosome (hsa03010),Long-term potentiation (hsa04720) |
| RUVBL1 | Wnt signaling pathway (hsa04310) |
| SF3B6 | Spliceosome (hsa03040) |
| SLC25A4 | Parkinson's disease (hsa05012),HTLV-I infection (hsa05166),Osteoclast differentiation (hsa04380),Huntington's disease (hsa05016),Calcium signaling pathway (hsa04020),cGMP-PKG signaling pathway (hsa04022) |
| SNRPD3 | Spliceosome (hsa03040),Systemic lupus erythematosus (hsa05322) |
| TRIP13 | NA |
| TTC27 | NA |
| TXN | NA |

**Table 4. First connections of the *ATG10*:** Table shows the first connections of the *ATG10* gene within the network of up-regulated transcripts . Name gene and KEGG pathway in which gene is associated with, is indicated. NA= Not associated KEGG pathway.

| GEN | KEGG |
| --- | --- |
| ALDH7A1 | Lysine biosynthesis (hsa00300),Propanoate metabolism (hsa00640),Pyruvate metabolism (hsa00620),Biosynthesis of amino acids (hsa01230),Fatty acid metabolism (hsa00071),Butanoate metabolism (hsa00650),Lysine degradation (hsa00310),Glycolysis and Gluconeogenesis (hsa00010),Histidine metabolism (hsa00340),Tryptophan metabolism (hsa00380),Arginine and proline metabolism (hsa00330),Metabolic pathways (hsa01100),Valine, leucine and isoleucine degradation (hsa00280),Glycine, serine and threonine metabolism (hsa00260),Glycerolipid metabolism (hsa00561),beta-Alanine metabolism (hsa00410),Ascorbate and aldarate metabolism (hsa00053) |
| ATG3 | Regulation of autophagy (hsa04140) |
| ATP5C1 | Alzheimer's disease (hsa05010),Metabolic pathways (hsa01100),Parkinson's disease (hsa05012),Oxidative phosphorylation (hsa00190),Huntington's disease (hsa05016),Aminoacyl-tRNA biosynthesis (hsa00970) |
| BAG2 | Protein processing in endoplasmic reticulum (hsa04141) |
| BUB3 | Cell cycle (hsa04110),HTLV-I infection (hsa05166) |
| C1QBP | Herpes simplex infection (hsa05168) |
| CACYBP | Wnt signaling pathway (hsa04310) |
| CCT2 | Aminoacyl-tRNA biosynthesis (hsa00970) |
| CCT6A | NA |
| CHEK1 | Viral carcinogenesis (Viral carcinogenesis (hsa05203)),Cell cycle (hsa04110),p53 signaling pathway (hsa04115),HTLV-I infection (hsa05166) |
| CSE1L | NA |
| DLD | Pyruvate metabolism (hsa00620),Citrate cycle (TCA cycle) (hsa00020),Glyoxylate and dicarboxylate metabolism (hsa00630),Carbon metabolism (hsa01200),Glycolysis and Gluconeogenesis (hsa00010),Glycine, serine and threonine metabolism (hsa00260),Valine, leucine and isoleucine degradation (hsa00280),Metabolic pathways (hsa01100),Glutamatergic synapse (hsa04724),Neuroactive ligand-receptor interaction (hsa04080) |
| DNAJC10 | Protein processing in endoplasmic reticulum (hsa04141) |
| DPM1 | N-Glycan biosynthesis (hsa00510),Metabolic pathways (hsa01100) |
| DUT | Metabolic pathways (hsa01100),Pyrimidine metabolism (hsa00240) |
| DYNC1LI1 | Phagosome (hsa04145),Vasopressin-regulated water reabsorption (hsa04962),Salmonella infection (hsa05132) |
| EPRS | Glutathione metabolism (hsa00480),Carbon metabolism (hsa01200),Metabolic pathways (hsa01100),2-Oxocarboxylic acid metabolism (hsa01210),Peroxisome (hsa04146),Biosynthesis of amino acids (hsa01230),Citrate cycle (TCA cycle) (hsa00020),Porphyrin and chlorophyll metabolism (hsa00860),Aminoacyl-tRNA biosynthesis (hsa00970) |
| FAR1 | Peroxisome (hsa04146) |
| GART | One carbon pool by folate (hsa00670),Metabolic pathways (hsa01100),Purine metabolism (hsa00230) |
| HAT1 | Alcoholism (hsa05034),Maturity onset diabetes of the young (hsa04950) |
| HPRT1 | Adherens junction (hsa04520),Focal adhesion (hsa04510),Regulation of actin cytoskeleton (hsa04810),Systemic lupus erythematosus (hsa05322),Amoebiasis (hsa05146),Tight junction (hsa04530),Arrhythmogenic right ventricular cardiomyopathy (ARVC) (hsa05412),Leukocyte transendothelial migration (hsa04670),Drug metabolism - other enzymes (hsa00983),Viral carcinogenesis (Viral carcinogenesis (hsa05203)),Purine metabolism (hsa00230),Metabolic pathways (hsa01100) |
| HSDL2 | NA |
| MCM3 | Non-alcoholic fatty liver disease (NAFLD) (hsa04932),Cell cycle (hsa04110),DNA replication (hsa03030),Insulin resistance (hsa04931) |
| MCM5 | Vitamin digestion and absorption (hsa04977),DNA replication (hsa03030),Cell cycle (hsa04110) |
| MSH6 | NF-kappa B signaling pathway (hsa04064),Pathways in cancer (hsa05200),RNA transport (hsa03013),MicroRNAs in cancer (hsa05206),Ubiquitin mediated proteolysis (hsa04120),Mismatch repair (hsa03430),Colorectal cancer (hsa05210) |
| MTHFD1 | Metabolic pathways (hsa01100),One carbon pool by folate (hsa00670),Glyoxylate and dicarboxylate metabolism (hsa00630) |
| NAP1L1 | NA |
| NCL | Pathogenic Escherichia coli infection (Pathogenic Escherichia coli infection (hsa05130)) |
| NDUFA4 | Huntington's disease (hsa05016),Non-alcoholic fatty liver disease (NAFLD) (hsa04932),Oxidative phosphorylation (hsa00190),Parkinson's disease (hsa05012),Alzheimer's disease (hsa05010),Metabolic pathways (hsa01100) |
| NDUFS1 | Parkinson's disease (hsa05012),Alzheimer's disease (hsa05010),Metabolic pathways (hsa01100),Huntington's disease (hsa05016),Non-alcoholic fatty liver disease (NAFLD) (hsa04932),Oxidative phosphorylation (hsa00190) |
| NPM1 | NA |
| PAICS | Purine metabolism (hsa00230),Metabolic pathways (hsa01100) |
| PCMT1 | NA |
| PCNA | Hepatitis B (hsa05161),HTLV-I infection (hsa05166),Mismatch repair (hsa03430),Base excision repair (hsa03410),Endocytosis (hsa04144),Cell cycle (hsa04110),Nucleotide excision repair (hsa03420),DNA replication (hsa03030) |
| PFAS | Circadian entrainment (hsa04713),Circadian rhythm (hsa04710),Metabolic pathways (hsa01100),Purine metabolism (hsa00230),Herpes simplex infection (hsa05168) |
| PPIA | Notch signaling pathway (hsa04330) |
| PRDX3 | Ubiquitin mediated proteolysis (hsa04120) |
| PRDX4 | NA |
| PSMA5 | Proteasome (hsa03050) |
| RBBP7 | NA |
| RPL23A | Glycosphingolipid biosynthesis - globo series (hsa00603),Metabolic pathways (hsa01100),Glycosphingolipid biosynthesis - lacto and neolacto series (hsa00601),Ribosome (hsa03010) |
| RPS21 | Ribosome (hsa03010) |
| RRM1 | Metabolic pathways (hsa01100),Purine metabolism (hsa00230),T cell receptor signaling pathway (hsa04660),Glutathione metabolism (hsa00480),Fc gamma R-mediated phagocytosis (hsa04666),Leukocyte transendothelial migration (hsa04670),Fc epsilon RI signaling pathway (hsa04664),Chemokine signaling pathway (hsa04062),B cell receptor signaling pathway (hsa04662),Natural killer cell mediated cytotoxicity (hsa04650),cAMP signaling pathway (hsa04024),Focal adhesion (hsa04510),Regulation of actin cytoskeleton (hsa04810),Pyrimidine metabolism (hsa00240) |
| RUVBL1 | Wnt signaling pathway (hsa04310) |
| SDHB | Morphine addiction (hsa05032),Ras signaling pathway (hsa04014),Retrograde endocannabinoid signaling (hsa04723),Huntington's disease (hsa05016),Non-alcoholic fatty liver disease (NAFLD) (hsa04932),Alcoholism (hsa05034),Chemokine signaling pathway (hsa04062),GABAergic synapse (hsa04727),Oxidative phosphorylation (hsa00190),Circadian entrainment (hsa04713),PI3K-Akt signaling pathway (hsa04151),Serotonergic synapse (hsa04726),Citrate cycle (TCA cycle) (hsa00020),Pathways in cancer (hsa05200),Glutamatergic synapse (hsa04724),Parkinson's disease (hsa05012),Dopaminergic synapse (hsa04728),Carbon metabolism (hsa01200),Alzheimer's disease (hsa05010),Metabolic pathways (hsa01100),Cholinergic synapse (hsa04725) |
| SF3B6 | Spliceosome (hsa03040) |
| SLC25A4 | Parkinson's disease (hsa05012),HTLV-I infection (hsa05166),Osteoclast differentiation (hsa04380),Huntington's disease (hsa05016),Calcium signaling pathway (hsa04020),cGMP-PKG signaling pathway (hsa04022) |
| TMPO | NA |
| TRIP13 | NA |
| TXN | NA |

**Table 5. First connection of *BRCA1*:** Table shows the first connections of *BRCA1* gene within the network of up-regulated transcripts. Name gene and KEGG pathway in which gene is associated with, is indicated. NA= Not associated KEGG pathway.

| GEN | KEGG |
| --- | --- |
| ATM | Mineral absorption (hsa04978),MicroRNAs in cancer (hsa05206),Cell cycle (hsa04110),Apoptosis (hsa04210),Transcriptional misregulation in cancer (hsa05202),FoxO signaling pathway (hsa04068),HTLV-I infection (hsa05166),NF-kappa B signaling pathway (hsa04064),p53 signaling pathway (hsa04115) |
| AURKA | Oocyte meiosis (hsa04114) |
| BARD1 | Oxytocin signaling pathway (hsa04921),Calcium signaling pathway (hsa04020),Hematopoietic cell lineage (hsa04640),Metabolic pathways (hsa01100),Nicotinate and nicotinamide metabolism (hsa00760),Salivary secretion (hsa04970),Epstein-Barr virus infection (hsa05169),Pancreatic secretion (hsa04972) |
| BLM | Homologous recombination (hsa03440),Fanconi anemia pathway (hsa03460) |
| CCNA2 | Viral carcinogenesis (Viral carcinogenesis (hsa05203)),Cell cycle (hsa04110),Epstein-Barr virus infection (hsa05169),AMPK signaling pathway (hsa04152),Progesterone-mediated oocyte maturation (hsa04914),Hepatitis B (hsa05161) |
| CCNB1 | Cell cycle (hsa04110),Metabolic pathways (hsa01100),Oocyte meiosis (hsa04114),FoxO signaling pathway (hsa04068),Retinol metabolism (hsa00830),Fatty acid metabolism (hsa00071),Progesterone-mediated oocyte maturation (hsa04914),p53 signaling pathway (hsa04115),Vascular smooth muscle contraction (hsa04270),Arachidonic acid metabolism (hsa00590) |
| CCNE1 | Oocyte meiosis (hsa04114),Aldosterone synthesis and secretion (hsa04925),Metabolic pathways (hsa01100),MicroRNAs in cancer (hsa05206),Small cell lung cancer (hsa05222),Cell cycle (hsa04110),Viral carcinogenesis (Viral carcinogenesis (hsa05203)),Measles (hsa05162),Pathways in cancer (hsa05200),Hepatitis B (hsa05161),p53 signaling pathway (hsa04115),PI3K-Akt signaling pathway (hsa04151),Steroid hormone biosynthesis (hsa00140),Prostate cancer (hsa05215) |
| CDK1 | Cell cycle (hsa04110),Oocyte meiosis (hsa04114),p53 signaling pathway (hsa04115),Gap junction (hsa04540),Progesterone-mediated oocyte maturation (hsa04914),Herpes simplex infection (hsa05168),Epstein-Barr virus infection (hsa05169),Viral carcinogenesis (Viral carcinogenesis (hsa05203)) |
| CHEK1 | Viral carcinogenesis (Viral carcinogenesis (hsa05203)),Cell cycle (hsa04110),p53 signaling pathway (hsa04115),HTLV-I infection (hsa05166) |
| FAM175A | NA |
| FANCA | Dilated cardiomyopathy (hsa05414),Focal adhesion (hsa04510),Regulation of actin cytoskeleton (hsa04810),Bacterial invasion of epithelial cells (hsa05100),Adherens junction (hsa04520),Gastric acid secretion (hsa04971),Hypertrophic cardiomyopathy (HCM) (hsa05410),Rap1 signaling pathway (hsa04015),Phagosome (hsa04145),Fanconi anemia pathway (hsa03460),Platelet activation (hsa04611),Oxytocin signaling pathway (hsa04921),Tight junction (hsa04530),Thyroid hormone signaling pathway (hsa04919),Arrhythmogenic right ventricular cardiomyopathy (ARVC) (hsa05412),Salmonella infection (hsa05132),Leukocyte transendothelial migration (hsa04670),Pathogenic Escherichia coli infection (Pathogenic Escherichia coli infection (hsa05130)),Influenza A (hsa05164),Hippo signaling pathway (hsa04390),Shigellosis (hsa05131),Viral myocarditis (Viral myocarditis (hsa05416)),Vibrio cholerae infection (hsa05110),Proteoglycans in cancer (hsa05205) |
| FANCD2 | Fanconi anemia pathway (hsa03460) |
| HIST1H4L | Osteoclast differentiation (hsa04380),Alcoholism (hsa05034),Systemic lupus erythematosus (hsa05322),NF-kappa B signaling pathway (hsa04064),Pathways in cancer (hsa05200),HTLV-I infection (hsa05166),Legionellosis (hsa05134),Viral carcinogenesis (Viral carcinogenesis (hsa05203)),Epstein-Barr virus infection (hsa05169),MAPK signaling pathway (hsa04010) |
| KPNA2 | Influenza A (hsa05164) |
| MED21 | NA |
| MLH1 | Glutamatergic synapse (hsa04724),Bile secretion (hsa04976),GnRH signaling pathway (hsa04912),Mismatch repair (hsa03430),Pancreatic secretion (hsa04972),Insulin secretion (hsa04911),Melanogenesis (hsa04916),Colorectal cancer (hsa05210),Oocyte meiosis (hsa04114),Ovarian steroidogenesis (hsa04913),Regulation of lipolysis in adipocytes (hsa04923),Purine metabolism (hsa00230),Aldosterone synthesis and secretion (hsa04925),Endometrial cancer (hsa05213),Endocrine and other factor-regulated calcium reabsorption (hsa04961),Cholinergic synapse (hsa04725),Salivary secretion (hsa04970),Dilated cardiomyopathy (hsa05414),Thyroid hormone synthesis (hsa04918),Morphine addiction (hsa05032),cGMP-PKG signaling pathway (hsa04022),Retrograde endocannabinoid signaling (hsa04723),Gastric acid secretion (hsa04971),cAMP signaling pathway (hsa04024),Gap junction (hsa04540),Rap1 signaling pathway (hsa04015),Estrogen signaling pathway (hsa04915),Phospholipase D signaling pathway (hsa04072),Fanconi anemia pathway (hsa03460),Adrenergic signaling in cardiomyocytes (hsa04261),Platelet activation (hsa04611),Renin secretion (hsa04924),Oxytocin signaling pathway (hsa04921),HTLV-I infection (hsa05166),Inflammatory mediator regulation of TRP channels (hsa04750),GABAergic synapse (hsa04727),Chemokine signaling pathway (hsa04062),Taste transduction (hsa04742),Progesterone-mediated oocyte maturation (hsa04914),Circadian entrainment (hsa04713),Vasopressin-regulated water reabsorption (hsa04962),Pathways in cancer (hsa05200),Vascular smooth muscle contraction (hsa04270) |
| MSH2 | Melanogenesis (hsa04916),Insulin secretion (hsa04911),Pancreatic secretion (hsa04972),Mismatch repair (hsa03430),GnRH signaling pathway (hsa04912),Ubiquitin mediated proteolysis (hsa04120),Protein processing in endoplasmic reticulum (hsa04141),Bile secretion (hsa04976),Glutamatergic synapse (hsa04724),Salivary secretion (hsa04970),Cholinergic synapse (hsa04725),Aldosterone synthesis and secretion (hsa04925),Purine metabolism (hsa00230),Regulation of lipolysis in adipocytes (hsa04923),Ovarian steroidogenesis (hsa04913),Oocyte meiosis (hsa04114),Colorectal cancer (hsa05210),Phospholipase D signaling pathway (hsa04072),Estrogen signaling pathway (hsa04915),Rap1 signaling pathway (hsa04015),Gap junction (hsa04540),cAMP signaling pathway (hsa04024),Gastric acid secretion (hsa04971),Calcium signaling pathway (hsa04020),Retrograde endocannabinoid signaling (hsa04723),cGMP-PKG signaling pathway (hsa04022),Morphine addiction (hsa05032),Thyroid hormone synthesis (hsa04918),Dilated cardiomyopathy (hsa05414),Vascular smooth muscle contraction (hsa04270),Pathways in cancer (hsa05200),Circadian entrainment (hsa04713),Progesterone-mediated oocyte maturation (hsa04914),Taste transduction (hsa04742),Chemokine signaling pathway (hsa04062),GABAergic synapse (hsa04727),Inflammatory mediator regulation of TRP channels (hsa04750),HTLV-I infection (hsa05166),Oxytocin signaling pathway (hsa04921),Platelet activation (hsa04611),Adrenergic signaling in cardiomyocytes (hsa04261),Long-term potentiation (hsa04720) |
| MSH6 | NF-kappa B signaling pathway (hsa04064),Pathways in cancer (hsa05200),RNA transport (hsa03013),MicroRNAs in cancer (hsa05206),Ubiquitin mediated proteolysis (hsa04120),Mismatch repair (hsa03430),Colorectal cancer (hsa05210) |
| NPM1 | NA |
| ORC3 | Cell cycle (hsa04110) |
| PIK3R1 | Influenza A (hsa05164),mTOR signaling pathway (hsa04150),Glioma (hsa05214),Choline metabolism in cancer (hsa05231),Rap1 signaling pathway (hsa04015),Estrogen signaling pathway (hsa04915),Phospholipase D signaling pathway (hsa04072),Renal cell carcinoma (hsa05211),Small cell lung cancer (hsa05222),Pancreatic cancer (hsa05212),Viral carcinogenesis (Viral carcinogenesis (hsa05203)),Signaling pathways regulating pluripotency of stem cells (hsa04550),Neurotrophin signaling pathway (hsa04722),Non-alcoholic fatty liver disease (NAFLD) (hsa04932),cAMP signaling pathway (hsa04024),Leukocyte transendothelial migration (hsa04670),Osteoclast differentiation (hsa04380),Apoptosis (hsa04210),Insulin resistance (hsa04931),Epstein-Barr virus infection (hsa05169),cGMP-PKG signaling pathway (hsa04022),Ras signaling pathway (hsa04014),Bacterial invasion of epithelial cells (hsa05100),VEGF signaling pathway (hsa04370),Toxoplasmosis (hsa05145),Hepatitis C (hsa05160),Central carbon metabolism in cancer (hsa05230),Non-small cell lung cancer (hsa05223),Regulation of actin cytoskeleton (hsa04810),Prostate cancer (hsa05215),Focal adhesion (hsa04510),TNF signaling pathway (hsa04668),Type II diabetes mellitus (hsa04930),Pathways in cancer (hsa05200),Cholinergic synapse (hsa04725),Melanoma (hsa05218),Acute myeloid leukemia (hsa05221),Insulin signaling pathway (hsa04910),Prolactin signaling pathway (hsa04917),Proteoglycans in cancer (hsa05205),Thyroid hormone signaling pathway (hsa04919),Chronic myeloid leukemia (hsa05220),T cell receptor signaling pathway (hsa04660),Chemokine signaling pathway (hsa04062),Carbohydrate digestion and absorption (hsa04973),Phosphatidylinositol signaling system (hsa04070),Chagas disease (American trypanosomiasis) (hsa05142),Endometrial cancer (hsa05213),Fc epsilon RI signaling pathway (hsa04664),Hepatitis B (hsa05161),HIF-1 signaling pathway (hsa04066),Jak-STAT signaling pathway (hsa04630),PI3K-Akt signaling pathway (hsa04151),Progesterone-mediated oocyte maturation (hsa04914),Sphingolipid signaling pathway (hsa04071),ErbB signaling pathway (hsa04012),Oxytocin signaling pathway (hsa04921),Fc gamma R-mediated phagocytosis (hsa04666),AMPK signaling pathway (hsa04152),Inflammatory mediator regulation of TRP channels (hsa04750),Toll-like receptor signaling pathway (hsa04620),Amoebiasis (hsa05146),HTLV-I infection (hsa05166),Colorectal cancer (hsa05210),Aldosterone-regulated sodium reabsorption (hsa04960),Measles (hsa05162),Natural killer cell mediated cytotoxicity (hsa04650),Adrenergic signaling in cardiomyocytes (hsa04261),Platelet activation (hsa04611),FoxO signaling pathway (hsa04068),Regulation of lipolysis in adipocytes (hsa04923),B cell receptor signaling pathway (hsa04662) |
| RAD50 | Non-homologous end-joining (hsa03450),Homologous recombination (hsa03440) |
| RAD51 | Homologous recombination (hsa03440),Fanconi anemia pathway (hsa03460),Pathways in cancer (hsa05200),Ubiquitin mediated proteolysis (hsa04120),Pancreatic cancer (hsa05212) |
| RBBP7 | NA |
| RBBP8 | NA |
| RBL1 | Viral carcinogenesis (Viral carcinogenesis (hsa05203)),TGF-beta signaling pathway (hsa04350),Cell cycle (hsa04110) |
| TOP2A | NA |

**Table 7. First connection of *CDK1.*** Table shows the first connections of the *CDK1* gene within the network of up-regulated transcripts. Name gene and KEGG pathway in which gene is associated with, is indicated. NA= Not associated KEGG pathway

| GEN | KEGG |
| --- | --- |
| ATG4C | Regulation of autophagy (hsa04140) |
| AURKB | NA |
| BIRC5 | Metabolic pathways (hsa01100),Valine, leucine and isoleucine degradation (hsa00280),Colorectal cancer (hsa05210),Hippo signaling pathway (hsa04390),Hepatitis B (hsa05161),Pathways in cancer (hsa05200) |
| BRCA1 | Riboflavin metabolism (hsa00740),Lipoic acid metabolism (hsa00785),Alanine, aspartate and glutamate metabolism (hsa00250),Arginine and proline metabolism (hsa00330),Graft-versus-host disease (hsa05332),Cysteine and methionine metabolism (hsa00270),Endometrial cancer (hsa05213),Tryptophan metabolism (hsa00380),N-Glycan biosynthesis (hsa00510),Primary immunodeficiency (hsa05340),Nicotinate and nicotinamide metabolism (hsa00760),Glycine, serine and threonine metabolism (hsa00260),RIG-I-like receptor signaling pathway (hsa04622),Valine, leucine and isoleucine degradation (hsa00280),Glycosylphosphatidylinositol(GPI)-anchor biosynthesis (hsa00563),Glycerolipid metabolism (hsa00561),Starch and sucrose metabolism (hsa00500),SNARE interactions in vesicular transport (hsa04130),Autoimmune thyroid disease (hsa05320),Lysine degradation (hsa00310),Glycolysis and Gluconeogenesis (hsa00010),Aldosterone-regulated sodium reabsorption (hsa04960),Glutathione metabolism (hsa00480),Histidine metabolism (hsa00340),Retinol metabolism (hsa00830),Vibrio cholerae infection (hsa05110),Mismatch repair (hsa03430),ABC transporters (hsa02010),Drug metabolism - other enzymes (hsa00983),Vitamin B6 metabolism (hsa00750),Pathogenic Escherichia coli infection (Pathogenic Escherichia coli infection (hsa05130)),Base excision repair (hsa03410),Prion diseases (hsa05020),Basal cell carcinoma (hsa05217),Sphingolipid metabolism (hsa00600),Amyotrophic lateral sclerosis (ALS) (Amyotrophic lateral sclerosis (ALS) (hsa05014)),RNA polymerase (hsa03020),beta-Alanine metabolism (hsa00410),Phenylalanine metabolism (hsa00360),Nucleotide excision repair (hsa03420),Type II diabetes mellitus (hsa04930),DNA replication (hsa03030),Ubiquitin mediated proteolysis (hsa04120),Ether lipid metabolism (hsa00565),Non-small cell lung cancer (hsa05223),Allograft rejection (hsa05330),Notch signaling pathway (hsa04330),MicroRNAs in cancer (hsa05206),Glycosphingolipid biosynthesis - ganglio series (hsa00604),Taste transduction (hsa04742),PI3K-Akt signaling pathway (hsa04151),Propanoate metabolism (hsa00640),Metabolism of xenobiotics by cytochrome P450 (hsa00980),Amino sugar and nucleotide sugar metabolism (hsa00520),Pyruvate metabolism (hsa00620),Maturity onset diabetes of the young (hsa04950),Fructose and mannose metabolism (hsa00051),Acute myeloid leukemia (hsa05221),Melanoma (hsa05218),Citrate cycle (TCA cycle) (hsa00020),Arachidonic acid metabolism (hsa00590),Fanconi anemia pathway (hsa03460),Asthma (hsa05310),NOD-like receptor signaling pathway (hsa04621),Type I diabetes mellitus (hsa04940),Cardiac muscle contraction (hsa04260),Pentose phosphate pathway (hsa00030),Intestinal immune network for IgA production (hsa04672),Thiamine metabolism (hsa00730),Mucin type O-Glycan biosynthesis (hsa00512),Pantothenate and CoA biosynthesis (hsa00770),RNA degradation (hsa03018),Tyrosine metabolism (hsa00350),Inositol phosphate metabolism (hsa00562),Aminoacyl-tRNA biosynthesis (hsa00970),Porphyrin and chlorophyll metabolism (hsa00860),Fatty acid metabolism (hsa00071),Hedgehog signaling pathway (hsa04340),Cytosolic DNA-sensing pathway (hsa04623),mTOR signaling pathway (hsa04150),Drug metabolism - cytochrome P450 (hsa00982),Selenocompound metabolism (hsa00450),Proteasome (hsa03050),Homologous recombination (hsa03440) |
| CCNA2 | Viral carcinogenesis (Viral carcinogenesis (hsa05203)),Cell cycle (hsa04110),Epstein-Barr virus infection (hsa05169),AMPK signaling pathway (hsa04152),Progesterone-mediated oocyte maturation (hsa04914),Hepatitis B (hsa05161) |
| CCNB1 | Cell cycle (hsa04110),Metabolic pathways (hsa01100),Oocyte meiosis (hsa04114),FoxO signaling pathway (hsa04068),Retinol metabolism (hsa00830),Fatty acid metabolism (hsa00071),Progesterone-mediated oocyte maturation (hsa04914),p53 signaling pathway (hsa04115),Vascular smooth muscle contraction (hsa04270),Arachidonic acid metabolism (hsa00590) |
| CCNB1IP1 | NA |
| CCNB2 | HTLV-I infection (hsa05166),p53 signaling pathway (hsa04115),Progesterone-mediated oocyte maturation (hsa04914),FoxO signaling pathway (hsa04068),Oocyte meiosis (hsa04114),Cell cycle (hsa04110) |
| CCNE1 | Oocyte meiosis (hsa04114),Aldosterone synthesis and secretion (hsa04925),Metabolic pathways (hsa01100),MicroRNAs in cancer (hsa05206),Small cell lung cancer (hsa05222),Cell cycle (hsa04110),Viral carcinogenesis (Viral carcinogenesis (hsa05203)),Measles (hsa05162),Pathways in cancer (hsa05200),Hepatitis B (hsa05161),p53 signaling pathway (hsa04115),PI3K-Akt signaling pathway (hsa04151),Steroid hormone biosynthesis (hsa00140),Prostate cancer (hsa05215) |
| CCNF | MicroRNAs in cancer (hsa05206),Steroid biosynthesis (hsa00100),Metabolic pathways (hsa01100) |
| CDC25C | Amino sugar and nucleotide sugar metabolism (hsa00520),Progesterone-mediated oocyte maturation (hsa04914),Oocyte meiosis (hsa04114),MicroRNAs in cancer (hsa05206),Cell cycle (hsa04110) |
| CDCA2 | NA |
| CDKN3 | Non-homologous end-joining (hsa03450),Hematopoietic cell lineage (hsa04640) |
| CDT1 | NA |
| DLGAP5 | NA |
| FANCA | Dilated cardiomyopathy (hsa05414),Focal adhesion (hsa04510),Regulation of actin cytoskeleton (hsa04810),Bacterial invasion of epithelial cells (hsa05100),Adherens junction (hsa04520),Gastric acid secretion (hsa04971),Hypertrophic cardiomyopathy (HCM) (hsa05410),Rap1 signaling pathway (hsa04015),Phagosome (hsa04145),Fanconi anemia pathway (hsa03460),Platelet activation (hsa04611),Oxytocin signaling pathway (hsa04921),Tight junction (hsa04530),Thyroid hormone signaling pathway (hsa04919),Arrhythmogenic right ventricular cardiomyopathy (ARVC) (hsa05412),Salmonella infection (hsa05132),Leukocyte transendothelial migration (hsa04670),Pathogenic Escherichia coli infection (Pathogenic Escherichia coli infection (hsa05130)),Influenza A (hsa05164),Hippo signaling pathway (hsa04390),Shigellosis (hsa05131),Viral myocarditis (Viral myocarditis (hsa05416)),Vibrio cholerae infection (hsa05110),Proteoglycans in cancer (hsa05205) |
| FANCC | Fanconi anemia pathway (hsa03460) |
| FANCG | Cytokine-cytokine receptor interaction (hsa04060),Jak-STAT signaling pathway (hsa04630),Fanconi anemia pathway (hsa03460),Toxoplasmosis (hsa05145),Tuberculosis (hsa05152),Epstein-Barr virus infection (hsa05169) |
| FEN1 | Non-homologous end-joining (hsa03450),Base excision repair (hsa03410),DNA replication (hsa03030) |
| GBAS | Complement and coagulation cascades (hsa04610),Measles (hsa05162) |
| GEN | KEGG |
| HIST1H1A | Parkinson's disease (hsa05012),Alzheimer's disease (hsa05010),Metabolic pathways (hsa01100),Huntington's disease (hsa05016),Non-alcoholic fatty liver disease (NAFLD) (hsa04932),Oxidative phosphorylation (hsa00190) |
| MKI67 | NA |
| PBK | NA |
| PKMYT1 | Progesterone-mediated oocyte maturation (hsa04914),Oocyte meiosis (hsa04114),Cell cycle (hsa04110) |
| SPAG5 | NA |
| USP45 | NA |
| ZBTB16 | Pathways in cancer (hsa05200),Acute myeloid leukemia (hsa05221),Transcriptional misregulation in cancer (hsa05202) |

**Table 8. First connections of the *GBAS* gene.** Table shows the first connections of the *GBAS* gene within the network of up-regulated transcripts. Name gene and KEGG pathway in which gene is associated with, is indicated. NA= Not associated

| GEN | KEGG |
| --- | --- |
| ACAT1 | Signaling pathways regulating pluripotency of stem cells (hsa04550),Glyoxylate and dicarboxylate metabolism (hsa00630),Fatty acid metabolism (hsa00071),Butanoate metabolism (hsa00650),Propanoate metabolism (hsa00640),Cytokine-cytokine receptor interaction (hsa04060),Pyruvate metabolism (hsa00620),TGF-beta signaling pathway (hsa04350),Terpenoid backbone biosynthesis (hsa00900),Tryptophan metabolism (hsa00380),Metabolic pathways (hsa01100),Valine, leucine and isoleucine degradation (hsa00280),Carbon metabolism (hsa01200),Lysine degradation (hsa00310),Synthesis and degradation of ketone bodies (hsa00072),Fatty acid metabolism (hsa01212) |
| ADSL | cGMP-PKG signaling pathway (hsa04022),Calcium signaling pathway (hsa04020),Huntington's disease (hsa05016),HTLV-I infection (hsa05166),Parkinson's disease (hsa05012),Purine metabolism (hsa00230),Metabolic pathways (hsa01100),Alanine, aspartate and glutamate metabolism (hsa00250) |
| AGL | Starch and sucrose metabolism (hsa00500),Metabolic pathways (hsa01100),Regulation of lipolysis in adipocytes (hsa04923),Neuroactive ligand-receptor interaction (hsa04080),Sphingolipid signaling pathway (hsa04071),Renin secretion (hsa04924),cAMP signaling pathway (hsa04024),cGMP-PKG signaling pathway (hsa04022),Morphine addiction (hsa05032) |
| AKR1B1 | Galactose metabolism (hsa00052),Metabolic pathways (hsa01100),Glycerolipid metabolism (hsa00561),Pentose and glucuronate interconversions (hsa00040),Endocytosis (hsa04144),Fructose and mannose metabolism (hsa00051),Pyruvate metabolism (hsa00620) |
| ALDH7A1 | Lysine biosynthesis (hsa00300),Propanoate metabolism (hsa00640),Pyruvate metabolism (hsa00620),Biosynthesis of amino acids (hsa01230),Fatty acid metabolism (hsa00071),Butanoate metabolism (hsa00650),Lysine degradation (hsa00310),Glycolysis and Gluconeogenesis (hsa00010),Histidine metabolism (hsa00340),Tryptophan metabolism (hsa00380),Arginine and proline metabolism (hsa00330),Metabolic pathways (hsa01100),Valine, leucine and isoleucine degradation (hsa00280),Glycine, serine and threonine metabolism (hsa00260),Glycerolipid metabolism (hsa00561),beta-Alanine metabolism (hsa00410),Ascorbate and aldarate metabolism (hsa00053) |
| ANP32E | NA |
| AP2B1 | Endocytosis (hsa04144),Synaptic vesicle cycle (hsa04721),Endocrine and other factor-regulated calcium reabsorption (hsa04961),Huntington's disease (hsa05016) |
| ATP5C1 | Alzheimer's disease (hsa05010),Metabolic pathways (hsa01100),Parkinson's disease (hsa05012),Oxidative phosphorylation (hsa00190),Huntington's disease (hsa05016),Aminoacyl-tRNA biosynthesis (hsa00970) |
| BUB3 | Cell cycle (hsa04110),HTLV-I infection (hsa05166) |
| C1QBP | Herpes simplex infection (hsa05168) |
| CCT2 | Aminoacyl-tRNA biosynthesis (hsa00970) |
| CCT6A | NA |
| CDK1 | Cell cycle (hsa04110),Oocyte meiosis (hsa04114),p53 signaling pathway (hsa04115),Gap junction (hsa04540),Progesterone-mediated oocyte maturation (hsa04914),Herpes simplex infection (hsa05168),Epstein-Barr virus infection (hsa05169),Viral carcinogenesis (Viral carcinogenesis (hsa05203)) |
| CPSF2 | mRNA surveillance pathway (hsa03015) |
| CSE1L | NA |
| DARS2 | Aminoacyl-tRNA biosynthesis (hsa00970) |
| DLAT | Citrate cycle (TCA cycle) (hsa00020),Pyruvate metabolism (hsa00620),Glycolysis and Gluconeogenesis (hsa00010),Carbon metabolism (hsa01200),Metabolic pathways (hsa01100) |
| DLD | Pyruvate metabolism (hsa00620),Citrate cycle (TCA cycle) (hsa00020),Glyoxylate and dicarboxylate metabolism (hsa00630),Carbon metabolism (hsa01200),Glycolysis and Gluconeogenesis (hsa00010),Glycine, serine and threonine metabolism (hsa00260),Valine, leucine and isoleucine degradation (hsa00280),Metabolic pathways (hsa01100),Glutamatergic synapse (hsa04724),Neuroactive ligand-receptor interaction (hsa04080) |
| DNM1L | TNF signaling pathway (hsa04668) |
| DTL | NA |
| EIF2S2 | RNA transport (hsa03013) |
| EPRS | Glutathione metabolism (hsa00480),Carbon metabolism (hsa01200),Metabolic pathways (hsa01100),2-Oxocarboxylic acid metabolism (hsa01210),Peroxisome (hsa04146),Biosynthesis of amino acids (hsa01230),Citrate cycle (TCA cycle) (hsa00020),Porphyrin and chlorophyll metabolism (hsa00860),Aminoacyl-tRNA biosynthesis (hsa00970) |
| ESD | Carbon metabolism (hsa01200) |
| FARSB | Aminoacyl-tRNA biosynthesis (hsa00970) |
| FEN1 | Non-homologous end-joining (hsa03450),Base excision repair (hsa03410),DNA replication (hsa03030) |
| FHL1 | Leukocyte transendothelial migration (hsa04670),T cell receptor signaling pathway (hsa04660),Chemokine signaling pathway (hsa04062),Jak-STAT signaling pathway (hsa04630) |
| GART | One carbon pool by folate (hsa00670),Metabolic pathways (hsa01100),Purine metabolism (hsa00230) |
| GNPNAT1 | Amino sugar and nucleotide sugar metabolism (hsa00520) |
| HDDC2 | NA |
| HNRNPC | Spliceosome (hsa03040) |
| IDH3A | Carbon metabolism (hsa01200),Metabolic pathways (hsa01100),2-Oxocarboxylic acid metabolism (hsa01210),Citrate cycle (TCA cycle) (hsa00020),Biosynthesis of amino acids (hsa01230) |
| LDHB | Glucagon signaling pathway (hsa04922),Pyruvate metabolism (hsa00620),Propanoate metabolism (hsa00640),MicroRNAs in cancer (hsa05206),Cysteine and methionine metabolism (hsa00270),Metabolic pathways (hsa01100),Glycolysis and Gluconeogenesis (hsa00010) |
| MAD2L1 | HTLV-I infection (hsa05166),Progesterone-mediated oocyte maturation (hsa04914),Cell cycle (hsa04110),Oocyte meiosis (hsa04114) |
| MCM3 | Non-alcoholic fatty liver disease (NAFLD) (hsa04932),Cell cycle (hsa04110),DNA replication (hsa03030),Insulin resistance (hsa04931) |
| MCM4 | Cell cycle (hsa04110),DNA replication (hsa03030) |
| MCM5 | Vitamin digestion and absorption (hsa04977),DNA replication (hsa03030),Cell cycle (hsa04110) |
| MCM6 | Cell cycle (hsa04110),DNA replication (hsa03030),Ribosome biogenesis in eukaryotes (hsa03008) |
| MSH2 | Melanogenesis (hsa04916),Insulin secretion (hsa04911),Pancreatic secretion (hsa04972),Mismatch repair (hsa03430),GnRH signaling pathway (hsa04912),Ubiquitin mediated proteolysis (hsa04120),Protein processing in endoplasmic reticulum (hsa04141),Bile secretion (hsa04976),Glutamatergic synapse (hsa04724),Salivary secretion (hsa04970),Cholinergic synapse (hsa04725),Aldosterone synthesis and secretion (hsa04925),Purine metabolism (hsa00230),Regulation of lipolysis in adipocytes (hsa04923),Ovarian steroidogenesis (hsa04913),Oocyte meiosis (hsa04114),Colorectal cancer (hsa05210),Phospholipase D signaling pathway (hsa04072),Estrogen signaling pathway (hsa04915),Rap1 signaling pathway (hsa04015),Gap junction (hsa04540),cAMP signaling pathway (hsa04024),Gastric acid secretion (hsa04971),Calcium signaling pathway (hsa04020),Retrograde endocannabinoid signaling (hsa04723),cGMP-PKG signaling pathway (hsa04022),Morphine addiction (hsa05032),Thyroid hormone synthesis (hsa04918),Dilated cardiomyopathy (hsa05414),Vascular smooth muscle contraction (hsa04270),Pathways in cancer (hsa05200),Circadian entrainment (hsa04713),Progesterone-mediated oocyte maturation (hsa04914),Taste transduction (hsa04742),Chemokine signaling pathway (hsa04062),GABAergic synapse (hsa04727),Inflammatory mediator regulation of TRP channels (hsa04750),HTLV-I infection (hsa05166),Oxytocin signaling pathway (hsa04921),Platelet activation (hsa04611),Adrenergic signaling in cardiomyocytes (hsa04261),Long-term potentiation (hsa04720) |
| MTHFD1 | Metabolic pathways (hsa01100),One carbon pool by folate (hsa00670),Glyoxylate and dicarboxylate metabolism (hsa00630) |
| NAP1L1 | NA |
| NASP | NA |
| NCAPH | NA |
| NCL | Pathogenic Escherichia coli infection (Pathogenic Escherichia coli infection (hsa05130)) |
| NPM1 | NA |
| NUP107 | RNA transport (hsa03013) |
| NUP155 | RNA transport (hsa03013) |
| ORC5 | Cell cycle (hsa04110) |
| PARK7 | Parkinson's disease (hsa05012) |
| PARP1 | NF-kappa B signaling pathway (hsa04064),Purine metabolism (hsa00230),Metabolic pathways (hsa01100),Base excision repair (hsa03410) |
| PCNA | Hepatitis B (hsa05161),HTLV-I infection (hsa05166),Mismatch repair (hsa03430),Base excision repair (hsa03410),Endocytosis (hsa04144),Cell cycle (hsa04110),Nucleotide excision repair (hsa03420),DNA replication (hsa03030) |
| PFAS | Circadian entrainment (hsa04713),Circadian rhythm (hsa04710),Metabolic pathways (hsa01100),Purine metabolism (hsa00230),Herpes simplex infection (hsa05168) |
| PMPCB | NA |
| PPIA | Notch signaling pathway (hsa04330) |
| PRPSAP2 | NA |
| PSMC1 | Viral carcinogenesis (Viral carcinogenesis (hsa05203)),Epstein-Barr virus infection (hsa05169),Chemokine signaling pathway (hsa04062),Cytokine-cytokine receptor interaction (hsa04060),Proteasome (hsa03050) |
| RAN | Ras signaling pathway (hsa04014),Osteoclast differentiation (hsa04380),Phospholipase D signaling pathway (hsa04072),Sphingolipid signaling pathway (hsa04071),HTLV-I infection (hsa05166),Fc epsilon RI signaling pathway (hsa04664),RNA transport (hsa03013),Chronic myeloid leukemia (hsa05220),Ribosome biogenesis in eukaryotes (hsa03008),Epstein-Barr virus infection (hsa05169),Fc gamma R-mediated phagocytosis (hsa04666) |
| RFC2 | Nucleotide excision repair (hsa03420),DNA replication (hsa03030),Mismatch repair (hsa03430) |
| RFC4 | RNA transport (hsa03013),Nucleotide excision repair (hsa03420),DNA replication (hsa03030),Mismatch repair (hsa03430) |
| RPA1 | Homologous recombination (hsa03440),Fanconi anemia pathway (hsa03460),RNA transport (hsa03013),DNA replication (hsa03030),Nucleotide excision repair (hsa03420),Mismatch repair (hsa03430) |
| RPL10A | Protein processing in endoplasmic reticulum (hsa04141),Ribosome (hsa03010) |
| RPL23A | Glycosphingolipid biosynthesis - globo series (hsa00603),Metabolic pathways (hsa01100),Glycosphingolipid biosynthesis - lacto and neolacto series (hsa00601),Ribosome (hsa03010) |
| RPS15A | Ribosome (hsa03010) |
| RPS24 | Leukocyte transendothelial migration (hsa04670),Rap1 signaling pathway (hsa04015),Phospholipase D signaling pathway (hsa04072),cAMP signaling pathway (hsa04024),Serotonergic synapse (hsa04726),Adrenergic signaling in cardiomyocytes (hsa04261),Ribosome (hsa03010),Long-term potentiation (hsa04720) |
| RRM1 | Metabolic pathways (hsa01100),Purine metabolism (hsa00230),T cell receptor signaling pathway (hsa04660),Glutathione metabolism (hsa00480),Fc gamma R-mediated phagocytosis (hsa04666),Leukocyte transendothelial migration (hsa04670),Fc epsilon RI signaling pathway (hsa04664),Chemokine signaling pathway (hsa04062),B cell receptor signaling pathway (hsa04662),Natural killer cell mediated cytotoxicity (hsa04650),cAMP signaling pathway (hsa04024),Focal adhesion (hsa04510),Regulation of actin cytoskeleton (hsa04810),Pyrimidine metabolism (hsa00240) |
| RUVBL1 | Wnt signaling pathway (hsa04310) |
| SEH1L | RNA transport (hsa03013) |
| SF3B6 | Spliceosome (hsa03040) |
| SKIV2L2 | RNA degradation (hsa03018) |
| SLC25A4 | Parkinson's disease (hsa05012),HTLV-I infection (hsa05166),Osteoclast differentiation (hsa04380),Huntington's disease (hsa05016),Calcium signaling pathway (hsa04020),cGMP-PKG signaling pathway (hsa04022) |
| SMC4 | NA |
| SNRPD3 | Spliceosome (hsa03040),Systemic lupus erythematosus (hsa05322) |
| SNRPE | Spliceosome (hsa03040) |
| SOD1 | Oocyte meiosis (hsa04114),Regulation of lipolysis in adipocytes (hsa04923),Ovarian steroidogenesis (hsa04913),Salivary secretion (hsa04970),Cholinergic synapse (hsa04725),Purine metabolism (hsa00230),Aldosterone synthesis and secretion (hsa04925),GnRH signaling pathway (hsa04912),Glutamatergic synapse (hsa04724),Bile secretion (hsa04976),Pancreatic secretion (hsa04972),Amyotrophic lateral sclerosis (ALS) (Amyotrophic lateral sclerosis (ALS) (hsa05014)),Prion diseases (hsa05020),Melanogenesis (hsa04916),Insulin secretion (hsa04911),Oxytocin signaling pathway (hsa04921),Inflammatory mediator regulation of TRP channels (hsa04750),HTLV-I infection (hsa05166),Long-term potentiation (hsa04720),Adrenergic signaling in cardiomyocytes (hsa04261),Platelet activation (hsa04611),Peroxisome (hsa04146),Pathways in cancer (hsa05200),Vascular smooth muscle contraction (hsa04270),Chemokine signaling pathway (hsa04062),GABAergic synapse (hsa04727),Circadian entrainment (hsa04713),Progesterone-mediated oocyte maturation (hsa04914),Taste transduction (hsa04742),cGMP-PKG signaling pathway (hsa04022),Morphine addiction (hsa05032),Calcium signaling pathway (hsa04020),Huntington's disease (hsa05016),Retrograde endocannabinoid signaling (hsa04723),Thyroid hormone synthesis (hsa04918),Dilated cardiomyopathy (hsa05414),Gap junction (hsa04540),Estrogen signaling pathway (hsa04915),Phospholipase D signaling pathway (hsa04072),Rap1 signaling pathway (hsa04015),Gastric acid secretion (hsa04971),cAMP signaling pathway (hsa04024) |
| SUPT16H | NA |
| TRIP13 | NA |
| TXN | NA |
| WDHD1 | NA |

**Table 9. First connections of the *ICT1* gene**. Table shows the first connections of the *ICT1* gene within the network of up-regulated transcripts. Name gene and KEGG pathway in which gene is associated with, is indicated. NA= Not associated KEGG pathway.

| GEN | KEGG |
| --- | --- |
| AASS | Lysine degradation (hsa00310),Metabolic pathways (hsa01100),Lysine biosynthesis (hsa00300) |
| ABCB7 | Biosynthesis of amino acids (hsa01230),Citrate cycle (TCA cycle) (hsa00020),Glyoxylate and dicarboxylate metabolism (hsa00630),Metabolic pathways (hsa01100),Carbon metabolism (hsa01200),2-Oxocarboxylic acid metabolism (hsa01210),ABC transporters (hsa02010) |
| ACOT9 | NA |
| ATP5C1 | Alzheimer's disease (hsa05010),Metabolic pathways (hsa01100),Parkinson's disease (hsa05012),Oxidative phosphorylation (hsa00190),Huntington's disease (hsa05016),Aminoacyl-tRNA biosynthesis (hsa00970) |
| ATP5F1 | Apoptosis (hsa04210),Parkinson's disease (hsa05012),Metabolic pathways (hsa01100),Alzheimer's disease (hsa05010),Non-alcoholic fatty liver disease (NAFLD) (hsa04932),TNF signaling pathway (hsa04668),Huntington's disease (hsa05016),Oxidative phosphorylation (hsa00190),Pertussis (hsa05133),Legionellosis (hsa05134) |
| ATP5L | Metabolic pathways (hsa01100),Oxidative phosphorylation (hsa00190) |
| C14orf156 | NA |
| C17orf42 | NA |
| C1QBP | Herpes simplex infection (hsa05168) |
| DCI | Fatty acid metabolism (hsa00071) |
| DLD | Pyruvate metabolism (hsa00620),Citrate cycle (TCA cycle) (hsa00020),Glyoxylate and dicarboxylate metabolism (hsa00630),Carbon metabolism (hsa01200),Glycolysis and Gluconeogenesis (hsa00010),Glycine, serine and threonine metabolism (hsa00260),Valine, leucine and isoleucine degradation (hsa00280),Metabolic pathways (hsa01100),Glutamatergic synapse (hsa04724),Neuroactive ligand-receptor interaction (hsa04080) |
| ECI1 | Fatty acid metabolism (hsa00071) |
| ERLIN2 | Porphyrin and chlorophyll metabolism (hsa00860) |
| GLUD1 | Arginine biosynthesis (hsa00220),Metabolic pathways (hsa01100),Arginine and proline metabolism (hsa00330),Alanine, aspartate and glutamate metabolism (hsa00250),Proximal tubule bicarbonate reclamation (hsa04964),Carbon metabolism (hsa01200),D-Glutamine and D-glutamate metabolism (hsa00471),Nitrogen metabolism (hsa00910) |
| GTPBP10 | NA |
| ISCA1 | NA |
| MRPL1 | Ribosome (hsa03010) |
| MRPL11 | Ribosome (hsa03010) |
| MRPL13 | Ribosome (hsa03010) |
| MRPL16 | Ribosome (hsa03010) |
| MRPL18 | Ribosome (hsa03010) |
| MRPL19 | Ribosome (hsa03010) |
| MRPL22 | Ribosome (hsa03010) |
| MRPL24 | Ribosome (hsa03010) |
| MRPL3 | Influenza A (hsa05164),Viral carcinogenesis (Viral carcinogenesis (hsa05203)),Herpes simplex infection (hsa05168),Osteoclast differentiation (hsa04380),Hepatitis C (hsa05160),Jak-STAT signaling pathway (hsa04630),Measles (hsa05162),Ribosome (hsa03010) |
| MRPL32 | Ribosome (hsa03010) |
| MRPL39 | NA |
| MRPL40 | NA |
| MRPL47 | NA |
| MRPL48 | NA |
| MRPL50 | NA |
| MRPS10 | Ribosome (hsa03010) |
| MRPS21 | Ribosome (hsa03010) |
| MRPS22 | NA |
| MRPS23 | NA |
| MRPS28 | NA |
| MRRF | NA |
| MTERFD1 | NA |
| NDUFA9 | Parkinson's disease (hsa05012),Alzheimer's disease (hsa05010),Metabolic pathways (hsa01100),Huntington's disease (hsa05016),Non-alcoholic fatty liver disease (NAFLD) (hsa04932),Oxidative phosphorylation (hsa00190) |
| NDUFS1 | Parkinson's disease (hsa05012),Alzheimer's disease (hsa05010),Metabolic pathways (hsa01100),Huntington's disease (hsa05016),Non-alcoholic fatty liver disease (NAFLD) (hsa04932),Oxidative phosphorylation (hsa00190) |
| OAT | Metabolic pathways (hsa01100),Arginine and proline metabolism (hsa00330),Transcriptional misregulation in cancer (hsa05202),MicroRNAs in cancer (hsa05206) |
| PDK3 | Neuroactive ligand-receptor interaction (hsa04080) |
| PHB2 | NA |
| PMPCB | NA |
| PRDX4 | NA |
| PTCD3 | NA |
| RARS2 | Aminoacyl-tRNA biosynthesis (hsa00970) |
| RBFA | NA |
| RG9MTD1 | NA |
| SLC25A12 | NA |
| SLC25A4 | Parkinson's disease (hsa05012),HTLV-I infection (hsa05166),Osteoclast differentiation (hsa04380),Huntington's disease (hsa05016),Calcium signaling pathway (hsa04020),cGMP-PKG signaling pathway (hsa04022) |
| SLIRP | NA |
| TFB1M | NA |
| YARS2 | Aminoacyl-tRNA biosynthesis (hsa00970) |

**Table 10. First connectios of *PCNA*.** Table shows the first connections of the *PCNA* gene within the network of up-regulated transcripts. Name gene and KEGG pathway in which gene is associated with, is indicated. NA= Not associated KEGG pathway.

| GEN | KEGG |
| --- | --- |
| ATG10 | Regulation of autophagy (hsa04140) |
| ATG4C | Regulation of autophagy (hsa04140) |
| BARD1 | Oxytocin signaling pathway (hsa04921),Calcium signaling pathway (hsa04020),Hematopoietic cell lineage (hsa04640),Metabolic pathways (hsa01100),Nicotinate and nicotinamide metabolism (hsa00760),Salivary secretion (hsa04970),Epstein-Barr virus infection (hsa05169),Pancreatic secretion (hsa04972) |
| CCNA2 | Viral carcinogenesis (Viral carcinogenesis (hsa05203)),Cell cycle (hsa04110),Epstein-Barr virus infection (hsa05169),AMPK signaling pathway (hsa04152),Progesterone-mediated oocyte maturation (hsa04914),Hepatitis B (hsa05161) |
| CDC25C | Amino sugar and nucleotide sugar metabolism (hsa00520),Progesterone-mediated oocyte maturation (hsa04914),Oocyte meiosis (hsa04114),MicroRNAs in cancer (hsa05206),Cell cycle (hsa04110) |
| CDC6 | Cell cycle (hsa04110) |
| CDKN1C | Metabolic pathways (hsa01100),Cysteine and methionine metabolism (hsa00270),Cell cycle (hsa04110),MicroRNAs in cancer (hsa05206) |
| CDT1 | NA |
| CHAF1A | Calcium signaling pathway (hsa04020),cGMP-PKG signaling pathway (hsa04022),Pathways in cancer (hsa05200),Melanogenesis (hsa04916),Neuroactive ligand-receptor interaction (hsa04080) |
| CHTF18 | NA |
| FEN1 | Non-homologous end-joining (hsa03450),Base excision repair (hsa03410),DNA replication (hsa03030) |
| GBAS | Complement and coagulation cascades (hsa04610),Measles (hsa05162) |
| ING2 | NA |
| KIAA0101 | NA |
| LIG1 | Base excision repair (hsa03410),Mismatch repair (hsa03430),Nucleotide excision repair (hsa03420),DNA replication (hsa03030) |
| MSH2 | Melanogenesis (hsa04916),Insulin secretion (hsa04911),Pancreatic secretion (hsa04972),Mismatch repair (hsa03430),GnRH signaling pathway (hsa04912),Ubiquitin mediated proteolysis (hsa04120),Protein processing in endoplasmic reticulum (hsa04141),Bile secretion (hsa04976),Glutamatergic synapse (hsa04724),Salivary secretion (hsa04970),Cholinergic synapse (hsa04725),Aldosterone synthesis and secretion (hsa04925),Purine metabolism (hsa00230),Regulation of lipolysis in adipocytes (hsa04923),Ovarian steroidogenesis (hsa04913),Oocyte meiosis (hsa04114),Colorectal cancer (hsa05210),Phospholipase D signaling pathway (hsa04072),Estrogen signaling pathway (hsa04915),Rap1 signaling pathway (hsa04015),Gap junction (hsa04540),cAMP signaling pathway (hsa04024),Gastric acid secretion (hsa04971),Calcium signaling pathway (hsa04020),Retrograde endocannabinoid signaling (hsa04723),cGMP-PKG signaling pathway (hsa04022),Morphine addiction (hsa05032),Thyroid hormone synthesis (hsa04918),Dilated cardiomyopathy (hsa05414),Vascular smooth muscle contraction (hsa04270),Pathways in cancer (hsa05200),Circadian entrainment (hsa04713),Progesterone-mediated oocyte maturation (hsa04914),Taste transduction (hsa04742),Chemokine signaling pathway (hsa04062),GABAergic synapse (hsa04727),Inflammatory mediator regulation of TRP channels (hsa04750),HTLV-I infection (hsa05166),Oxytocin signaling pathway (hsa04921),Platelet activation (hsa04611),Adrenergic signaling in cardiomyocytes (hsa04261),Long-term potentiation (hsa04720) |
| MSH6 | NF-kappa B signaling pathway (hsa04064),Pathways in cancer (hsa05200),RNA transport (hsa03013),MicroRNAs in cancer (hsa05206),Ubiquitin mediated proteolysis (hsa04120),Mismatch repair (hsa03430),Colorectal cancer (hsa05210) |
| MUTYH | Base excision repair (hsa03410),Insulin secretion (hsa04911),Renin secretion (hsa04924) |
| PARP1 | NF-kappa B signaling pathway (hsa04064),Purine metabolism (hsa00230),Metabolic pathways (hsa01100),Base excision repair (hsa03410) |
| PCNA | Hepatitis B (hsa05161),HTLV-I infection (hsa05166),Mismatch repair (hsa03430),Base excision repair (hsa03410),Endocytosis (hsa04144),Cell cycle (hsa04110),Nucleotide excision repair (hsa03420),DNA replication (hsa03030) |
| PIK3C3 | Tuberculosis (hsa05152),Regulation of autophagy (hsa04140),Metabolic pathways (hsa01100),Phosphatidylinositol signaling system (hsa04070),Arginine and proline metabolism (hsa00330),Inositol phosphate metabolism (hsa00562),Phagosome (hsa04145) |
| POLD3 | HTLV-I infection (hsa05166),Pyrimidine metabolism (hsa00240),Homologous recombination (hsa03440),Metabolic pathways (hsa01100),Purine metabolism (hsa00230),Nucleotide excision repair (hsa03420),DNA replication (hsa03030),Base excision repair (hsa03410),Mismatch repair (hsa03430) |
| PPIA | Notch signaling pathway (hsa04330) |
| RFC2 | Nucleotide excision repair (hsa03420),DNA replication (hsa03030),Mismatch repair (hsa03430) |
| RFC3 | Mismatch repair (hsa03430),Bile secretion (hsa04976),DNA replication (hsa03030),Nucleotide excision repair (hsa03420) |
| RFC4 | RNA transport (hsa03013),Nucleotide excision repair (hsa03420),DNA replication (hsa03030),Mismatch repair (hsa03430) |
| RFC5 | DNA replication (hsa03030),Nucleotide excision repair (hsa03420),Mismatch repair (hsa03430),Amyotrophic lateral sclerosis (ALS) (Amyotrophic lateral sclerosis (ALS) (hsa05014)) |
| RPA1 | Homologous recombination (hsa03440),Fanconi anemia pathway (hsa03460),RNA transport (hsa03013),DNA replication (hsa03030),Nucleotide excision repair (hsa03420),Mismatch repair (hsa03430) |
| WRN | Metabolism of xenobiotics by cytochrome P450 (hsa00980),Fatty acid metabolism (hsa00071),Tyrosine metabolism (hsa00350),Drug metabolism - cytochrome P450 (hsa00982),Metabolic pathways (hsa01100),Retinol metabolism (hsa00830),Carbon metabolism (hsa01200),Glycolysis and Gluconeogenesis (hsa00010),Chemical carcinogenesis (hsa05204) |

**Table 11. First connections of the *PIK3C3*.** Table shows the first connections of the *PIK3C3* gene within the network of up-regulated transcripts. Name gene and KEGG pathway in which gene is associated with, is indicated. NA= Not associated KEGG pathway.

| GEN | KEGG |
| --- | --- |
| ATG4C | Regulation of autophagy (hsa04140) |
| BAG2 | Protein processing in endoplasmic reticulum (hsa04141) |
| CCT2 | Aminoacyl-tRNA biosynthesis (hsa00970) |
| CCT6A | NA |
| DLD | Pyruvate metabolism (hsa00620),Citrate cycle (TCA cycle) (hsa00020),Glyoxylate and dicarboxylate metabolism (hsa00630),Carbon metabolism (hsa01200),Glycolysis and Gluconeogenesis (hsa00010),Glycine, serine and threonine metabolism (hsa00260),Valine, leucine and isoleucine degradation (hsa00280),Metabolic pathways (hsa01100),Glutamatergic synapse (hsa04724),Neuroactive ligand-receptor interaction (hsa04080) |
| GART | One carbon pool by folate (hsa00670),Metabolic pathways (hsa01100),Purine metabolism (hsa00230) |
| HPRT1 | Adherens junction (hsa04520),Focal adhesion (hsa04510),Regulation of actin cytoskeleton (hsa04810),Systemic lupus erythematosus (hsa05322),Amoebiasis (hsa05146),Tight junction (hsa04530),Arrhythmogenic right ventricular cardiomyopathy (ARVC) (hsa05412),Leukocyte transendothelial migration (hsa04670),Drug metabolism - other enzymes (hsa00983),Viral carcinogenesis (Viral carcinogenesis (hsa05203)),Purine metabolism (hsa00230),Metabolic pathways (hsa01100) |
| IMMT | NA |
| LDHB | Glucagon signaling pathway (hsa04922),Pyruvate metabolism (hsa00620),Propanoate metabolism (hsa00640),MicroRNAs in cancer (hsa05206),Cysteine and methionine metabolism (hsa00270),Metabolic pathways (hsa01100),Glycolysis and Gluconeogenesis (hsa00010) |
| MCM3 | Non-alcoholic fatty liver disease (NAFLD) (hsa04932),Cell cycle (hsa04110),DNA replication (hsa03030),Insulin resistance (hsa04931) |
| MCM5 | Vitamin digestion and absorption (hsa04977),DNA replication (hsa03030),Cell cycle (hsa04110) |
| MRPS33 | NA |
| MTHFD1 | Metabolic pathways (hsa01100),One carbon pool by folate (hsa00670),Glyoxylate and dicarboxylate metabolism (hsa00630) |
| NCL | Pathogenic Escherichia coli infection (Pathogenic Escherichia coli infection (hsa05130)) |
| NDUFA4 | Huntington's disease (hsa05016),Non-alcoholic fatty liver disease (NAFLD) (hsa04932),Oxidative phosphorylation (hsa00190),Parkinson's disease (hsa05012),Alzheimer's disease (hsa05010),Metabolic pathways (hsa01100) |
| NPM1 | NA |
| ORC3 | Cell cycle (hsa04110) |
| PCNA | Hepatitis B (hsa05161),HTLV-I infection (hsa05166),Mismatch repair (hsa03430),Base excision repair (hsa03410),Endocytosis (hsa04144),Cell cycle (hsa04110),Nucleotide excision repair (hsa03420),DNA replication (hsa03030) |
| PPIA | Notch signaling pathway (hsa04330) |
| PRDX3 | Ubiquitin mediated proteolysis (hsa04120) |
| PRDX4 | NA |
| RPS15A | Ribosome (hsa03010) |
| RPS24 | Leukocyte transendothelial migration (hsa04670),Rap1 signaling pathway (hsa04015),Phospholipase D signaling pathway (hsa04072),cAMP signaling pathway (hsa04024),Serotonergic synapse (hsa04726),Adrenergic signaling in cardiomyocytes (hsa04261),Ribosome (hsa03010),Long-term potentiation (hsa04720) |
| SF3B6 | Spliceosome (hsa03040) |
| SNRPD3 | Spliceosome (hsa03040),Systemic lupus erythematosus (hsa05322) |
| SNRPE | Spliceosome (hsa03040) |
| SNRPG | Spliceosome (hsa03040) |
| TMPO | NA |
| TRIP13 | NA |
| TXN | NA |

**Table 12. First connections of the *PRKAA1*.** Table shows the first connections of the *PRKAA1* gene within the network of up-regulated transcripts. Name gene and KEGG pathway in which gene is associated with, is indicated. NA= Not associated KEGG pathway.

| GEN | KEGG |
| --- | --- |
| ABCF2 | Phagosome (hsa04145),Rap1 signaling pathway (hsa04015),Hypertrophic cardiomyopathy (HCM) (hsa05410),Adherens junction (hsa04520),Bacterial invasion of epithelial cells (hsa05100),Regulation of actin cytoskeleton (hsa04810),Dilated cardiomyopathy (hsa05414),Focal adhesion (hsa04510),Arrhythmogenic right ventricular cardiomyopathy (ARVC) (hsa05412),Thyroid hormone signaling pathway (hsa04919),Oxytocin signaling pathway (hsa04921),Tight junction (hsa04530),Platelet activation (hsa04611),Influenza A (hsa05164),Pathogenic Escherichia coli infection (Pathogenic Escherichia coli infection (hsa05130)),Leukocyte transendothelial migration (hsa04670),Salmonella infection (hsa05132),Proteoglycans in cancer (hsa05205),Vibrio cholerae infection (hsa05110),Shigellosis (hsa05131),Viral myocarditis (Viral myocarditis (hsa05416)),Hippo signaling pathway (hsa04390) |
| BRIX1 | NA |
| BTF3 | NA |
| C1QBP | Herpes simplex infection (hsa05168) |
| C3orf26 | NA |
| CCBL2 | Chemical carcinogenesis (hsa05204),Metabolic pathways (hsa01100),Tryptophan metabolism (hsa00380),Selenocompound metabolism (hsa00450) |
| DEK | MAPK signaling pathway (hsa04010),Phospholipase D signaling pathway (hsa04072),Regulation of actin cytoskeleton (hsa04810),cGMP-PKG signaling pathway (hsa04022),Vascular smooth muscle contraction (hsa04270),Pathways in cancer (hsa05200),Long-term depression (hsa04730),Sphingolipid signaling pathway (hsa04071) |
| DHX29 | NA |
| EIF2S2 | RNA transport (hsa03013) |
| EXOSC2 | RNA degradation (hsa03018) |
| EXOSC9 | RNA degradation (hsa03018) |
| FARSB | Aminoacyl-tRNA biosynthesis (hsa00970) |
| FKBP3 | NA |
| FXR1 | RNA transport (hsa03013),Glycerolipid metabolism (hsa00561),Alzheimer's disease (hsa05010),PPAR signaling pathway (hsa03320) |
| G3BP1 | NA |
| GGH | Folate biosynthesis (hsa00790),N-Glycan biosynthesis (hsa00510),Metabolic pathways (hsa01100) |
| GNL3 | Ribosome biogenesis in eukaryotes (hsa03008) |
| HIST1H4L | Osteoclast differentiation (hsa04380),Alcoholism (hsa05034),Systemic lupus erythematosus (hsa05322),NF-kappa B signaling pathway (hsa04064),Pathways in cancer (hsa05200),HTLV-I infection (hsa05166),Legionellosis (hsa05134),Viral carcinogenesis (Viral carcinogenesis (hsa05203)),Epstein-Barr virus infection (hsa05169),MAPK signaling pathway (hsa04010) |
| HNRNPC | Spliceosome (hsa03040) |
| IMMT | NA |
| KIF2A | Amoebiasis (hsa05146) |
| KPNA2 | Influenza A (hsa05164) |
| LYAR | NA |
| MINA | NA |
| MKI67 | NA |
| MTDH | NA |
| NAP1L1 | NA |
| NCL | Pathogenic Escherichia coli infection (Pathogenic Escherichia coli infection (hsa05130)) |
| NOC3L | NA |
| NOP16 | NA |
| NPM1 | NA |
| PARP1 | NF-kappa B signaling pathway (hsa04064),Purine metabolism (hsa00230),Metabolic pathways (hsa01100),Base excision repair (hsa03410) |
| POP1 | Ribosome biogenesis in eukaryotes (hsa03008),RNA transport (hsa03013) |
| PPIA | Notch signaling pathway (hsa04330) |
| PRDX3 | Ubiquitin mediated proteolysis (hsa04120) |
| PRKAA1 | FoxO signaling pathway (hsa04068),Regulation of autophagy (hsa04140),mTOR signaling pathway (hsa04150),PI3K-Akt signaling pathway (hsa04151),AMPK signaling pathway (hsa04152),Longevity regulating pathway - mammal (hsa04211),Longevity regulating pathway - multiple species (hsa04213),Circadian rhythm (hsa04710),Insulin signaling pathway (hsa04910),Thyroid hormone signaling pathway (hsa04920),Oxytocin signaling pathway (hsa04921),Glucagon signaling pathway (hsa04922),Insulin resistance (hsa04931),Non-alcoholic fatty liver disease (NAFLD) (hsa04932),Hypertrophic cardiomyopathy (HCM) (hsa05410) |
| PWP1 | NA |
| RFC2 | Nucleotide excision repair (hsa03420),DNA replication (hsa03030),Mismatch repair (hsa03430) |
| RPA1 | Homologous recombination (hsa03440),Fanconi anemia pathway (hsa03460),RNA transport (hsa03013),DNA replication (hsa03030),Nucleotide excision repair (hsa03420),Mismatch repair (hsa03430) |
| RPL10A | Protein processing in endoplasmic reticulum (hsa04141),Ribosome (hsa03010) |
| RPL23A | Glycosphingolipid biosynthesis - globo series (hsa00603),Metabolic pathways (hsa01100),Glycosphingolipid biosynthesis - lacto and neolacto series (hsa00601),Ribosome (hsa03010) |
| RPL26L1 | Ribosome (hsa03010) |
| RPL35A | Ribosome (hsa03010) |
| RPS15A | Ribosome (hsa03010) |
| RPS21 | Ribosome (hsa03010) |
| RPS24 | Leukocyte transendothelial migration (hsa04670),Rap1 signaling pathway (hsa04015),Phospholipase D signaling pathway (hsa04072),cAMP signaling pathway (hsa04024),Serotonergic synapse (hsa04726),Adrenergic signaling in cardiomyocytes (hsa04261),Ribosome (hsa03010),Long-term potentiation (hsa04720) |
| SKIV2L2 | RNA degradation (hsa03018) |
| SNRPB2 | Spliceosome (hsa03040) |
| SNRPE | Spliceosome (hsa03040) |
| SNRPG | Spliceosome (hsa03040) |
| SRBD1 | NA |
| SUB1 | NA |
| SUPT16H | NA |
| TFB1M | NA |
| TXN | NA |

**Table 14. First connections of the *RPA1*.** Table shows the first connections of the *RPA1* gene within the network of up-regulated transcripts. Name gene and KEGG pathway in which gene is associated with, is indicated. NA= Not associated KEGG pathway.

| GEN | KKEG |
| --- | --- |
| APLF | NA |
| BLM | Homologous recombination (hsa03440),Fanconi anemia pathway (hsa03460) |
| CCNA2 | Viral carcinogenesis (Viral carcinogenesis (hsa05203)),Cell cycle (hsa04110),Epstein-Barr virus infection (hsa05169),AMPK signaling pathway (hsa04152),Progesterone-mediated oocyte maturation (hsa04914),Hepatitis B (hsa05161) |
| CENPA | NA |
| CHAF1A | Calcium signaling pathway (hsa04020),cGMP-PKG signaling pathway (hsa04022),Pathways in cancer (hsa05200),Melanogenesis (hsa04916),Neuroactive ligand-receptor interaction (hsa04080) |
| CHD1L | NA |
| FANCA | Dilated cardiomyopathy (hsa05414),Focal adhesion (hsa04510),Regulation of actin cytoskeleton (hsa04810),Bacterial invasion of epithelial cells (hsa05100),Adherens junction (hsa04520),Gastric acid secretion (hsa04971),Hypertrophic cardiomyopathy (HCM) (hsa05410),Rap1 signaling pathway (hsa04015),Phagosome (hsa04145),Fanconi anemia pathway (hsa03460),Platelet activation (hsa04611),Oxytocin signaling pathway (hsa04921),Tight junction (hsa04530),Thyroid hormone signaling pathway (hsa04919),Arrhythmogenic right ventricular cardiomyopathy (ARVC) (hsa05412),Salmonella infection (hsa05132),Leukocyte transendothelial migration (hsa04670),Pathogenic Escherichia coli infection (Pathogenic Escherichia coli infection (hsa05130)),Influenza A (hsa05164),Hippo signaling pathway (hsa04390),Shigellosis (hsa05131),Viral myocarditis (Viral myocarditis (hsa05416)),Vibrio cholerae infection (hsa05110),Proteoglycans in cancer (hsa05205) |
| FANCM | Fanconi anemia pathway (hsa03460) |
| GBAS | Complement and coagulation cascades (hsa04610),Measles (hsa05162) |
| MCM2 | DNA replication (hsa03030),Cell cycle (hsa04110) |
| MCM4 | Cell cycle (hsa04110),DNA replication (hsa03030) |
| MCM6 | Cell cycle (hsa04110),DNA replication (hsa03030),Ribosome biogenesis in eukaryotes (hsa03008) |
| MUTYH | Base excision repair (hsa03410),Insulin secretion (hsa04911),Renin secretion (hsa04924) |
| ORC6 | Cell cycle (hsa04110) |
| PCNA | Hepatitis B (hsa05161),HTLV-I infection (hsa05166),Mismatch repair (hsa03430),Base excision repair (hsa03410),Endocytosis (hsa04144),Cell cycle (hsa04110),Nucleotide excision repair (hsa03420),DNA replication (hsa03030) |
| POLA1 | Purine metabolism (hsa00230),Metabolic pathways (hsa01100),DNA replication (hsa03030),Pyrimidine metabolism (hsa00240) |
| PRIM1 | Cell adhesion molecules (CAMs) (hsa04514),Pyrimidine metabolism (hsa00240),Purine metabolism (hsa00230),Metabolic pathways (hsa01100),DNA replication (hsa03030) |
| PRIM2 | Type II diabetes mellitus (hsa04930),DNA replication (hsa03030),PPAR signaling pathway (hsa03320),Metabolic pathways (hsa01100),Purine metabolism (hsa00230),AMPK signaling pathway (hsa04152),Non-alcoholic fatty liver disease (NAFLD) (hsa04932),Pyrimidine metabolism (hsa00240),Thyroid hormone signaling pathway (hsa04920) |
| PRKAA1 | FoxO signaling pathway (hsa04068),Regulation of autophagy (hsa04140),mTOR signaling pathway (hsa04150),PI3K-Akt signaling pathway (hsa04151),AMPK signaling pathway (hsa04152),Longevity regulating pathway - mammal (hsa04211),Longevity regulating pathway - multiple species (hsa04213),Circadian rhythm (hsa04710),Insulin signaling pathway (hsa04910),Thyroid hormone signaling pathway (hsa04920),Oxytocin signaling pathway (hsa04921),Glucagon signaling pathway (hsa04922),Insulin resistance (hsa04931),Non-alcoholic fatty liver disease (NAFLD) (hsa04932),Hypertrophic cardiomyopathy (HCM) (hsa05410) |
| RAD1 | NA |
| RAD51 | Homologous recombination (hsa03440),Fanconi anemia pathway (hsa03460),Pathways in cancer (hsa05200),Ubiquitin mediated proteolysis (hsa04120),Pancreatic cancer (hsa05212) |
| RMI1 | Fanconi anemia pathway (hsa03460) |
| RPA3 | Spliceosome (hsa03040),Mismatch repair (hsa03430),DNA replication (hsa03030),Nucleotide excision repair (hsa03420),Fanconi anemia pathway (hsa03460),Homologous recombination (hsa03440) |
| TIPIN | NA |
| WRN | Metabolism of xenobiotics by cytochrome P450 (hsa00980),Fatty acid metabolism (hsa00071),Tyrosine metabolism (hsa00350),Drug metabolism - cytochrome P450 (hsa00982),Metabolic pathways (hsa01100),Retinol metabolism (hsa00830),Carbon metabolism (hsa01200),Glycolysis and Gluconeogenesis (hsa00010),Chemical carcinogenesis (hsa05204) |

**Table 15. First connections of the *GABARPAL1*.** Table shows the first connections of the *GABARPAL1* gene within the network of down-regulated transcripts. Name gene and KEGG pathway in which gene is associated with, is indicated. NA= Not associated KEGG pathway.

| GEN | KEGG |
| --- | --- |
| ADAR | ABC transporters (hsa02010),Influenza A (hsa05164),Peroxisome (hsa04146),Measles (hsa05162),Cytosolic DNA-sensing pathway (hsa04623) |
| ATG13 | Regulation of autophagy (hsa04140),Longevity regulating pathway - mammal (hsa04211) |
| CHERP | Spliceosome (hsa03040) |
| CLU | NA |
| CSDA | Tight junction (hsa04530) |
| CTSA | Lysosome (hsa04142),Renin-angiotensin system (hsa04614) |
| CTSD | Metabolic pathways (hsa01100),Tuberculosis (hsa05152),Glycosphingolipid biosynthesis - lacto and neolacto series (hsa00601),Sphingolipid signaling pathway (hsa04071),Other types of O-glycan biosynthesis (hsa00514),Lysosome (hsa04142) |
| EPDR1 | NA |
| FLCN | Renal cell carcinoma (hsa05211) |
| FYCO1 | NA |
| GABARAPL2 | Regulation of autophagy (hsa04140),FoxO signaling pathway (hsa04068),GABAergic synapse (hsa04727) |
| GTF2I | Platelet activation (hsa04611),Oxytocin signaling pathway (hsa04921),Vascular smooth muscle contraction (hsa04270),Regulation of actin cytoskeleton (hsa04810),Basal transcription factors (hsa03022),Focal adhesion (hsa04510),cGMP-PKG signaling pathway (hsa04022),cAMP signaling pathway (hsa04024),Proteoglycans in cancer (hsa05205),Herpes simplex infection (hsa05168) |
| HIST1H1C | Parkinson's disease (hsa05012),Alzheimer's disease (hsa05010),Metabolic pathways (hsa01100),Huntington's disease (hsa05016),Non-alcoholic fatty liver disease (NAFLD) (hsa04932),Oxidative phosphorylation (hsa00190) |
| HIST1H2BL | Alcoholism (hsa05034),Systemic lupus erythematosus (hsa05322),Viral carcinogenesis (Viral carcinogenesis (hsa05203)) |
| HSPA6 | Estrogen signaling pathway (hsa04915),Toxoplasmosis (hsa05145),Legionellosis (hsa05134),Antigen processing and presentation (hsa04612),Measles (hsa05162),Endocytosis (hsa04144),Influenza A (hsa05164),Epstein-Barr virus infection (hsa05169),Protein processing in endoplasmic reticulum (hsa04141),MAPK signaling pathway (hsa04010),Spliceosome (hsa03040) |
| KPNA6 | NA |
| KRT13 | NA |
| KRT75 | NA |
| LARP1 | NA |
| MYH9 | Tight junction (hsa04530),Regulation of actin cytoskeleton (hsa04810),Salmonella infection (hsa05132) |
| PABPC1 | Hepatitis C (hsa05160),RNA degradation (hsa03018),mRNA surveillance pathway (hsa03015),RNA transport (hsa03013),Jak-STAT signaling pathway (hsa04630),Ubiquitin mediated proteolysis (hsa04120) |
| PCBP1 | ABC transporters (hsa02010),Bile secretion (hsa04976),Spliceosome (hsa03040) |
| QPCT | NA |
| RNF123 | NA |
| RPTN | NA |
| SAFB2 | NA |
| SEC16A | NA |
| SERPINB13 | Amoebiasis (hsa05146) |
| SMARCA4 | Influenza A (hsa05164) |
| SQSTM1 | Osteoclast differentiation (hsa04380) |
| TECPR2 | NA |
| TFG | Thyroid cancer (hsa05216),Pathways in cancer (hsa05200) |
| TRIM21 | Systemic lupus erythematosus (hsa05322) |
| TUBA4A | Phagosome (hsa04145),Gap junction (hsa04540),Pathogenic Escherichia coli infection (Pathogenic Escherichia coli infection (hsa05130)) |
| ULK1 | mTOR signaling pathway (hsa04150),Regulation of autophagy (hsa04140),AMPK signaling pathway (hsa04152) |
| WBP2 | NA |
| WDR45L | NA |
| ZC3HAV1 | NA |

**Table 16. First connection of *CLN3*.** Table shows the first connections of the *CLN3* gene within the network of down-regulated transcripts. Name gene and KEGG pathway in which gene is associated with, is indicated. NA= Not associated KEGG pathway.

| GEN | KEGG |
| --- | --- |
| AGPAT6 | Glycerolipid metabolism (hsa00561),Metabolic pathways (hsa01100),Glycerophospholipid metabolism (hsa00564),Ether lipid metabolism (hsa00565) |
| ATP1B1 | Endocrine and other factor-regulated calcium reabsorption (hsa04961),Carbohydrate digestion and absorption (hsa04973),Salivary secretion (hsa04970),Aldosterone-regulated sodium reabsorption (hsa04960),Proximal tubule bicarbonate reclamation (hsa04964),Insulin secretion (hsa04911),Pancreatic secretion (hsa04972),Bile secretion (hsa04976),Mineral absorption (hsa04978),Thyroid hormone signaling pathway (hsa04919),Adrenergic signaling in cardiomyocytes (hsa04261),Cardiac muscle contraction (hsa04260),cAMP signaling pathway (hsa04024),Gastric acid secretion (hsa04971),Protein digestion and absorption (hsa04974),Thyroid hormone synthesis (hsa04918),cGMP-PKG signaling pathway (hsa04022) |
| ATP2A2 | Arrhythmogenic right ventricular cardiomyopathy (ARVC) (hsa05412),Thyroid hormone signaling pathway (hsa04919),Cardiac muscle contraction (hsa04260),Adrenergic signaling in cardiomyocytes (hsa04261),Hypertrophic cardiomyopathy (HCM) (hsa05410),cAMP signaling pathway (hsa04024),cGMP-PKG signaling pathway (hsa04022),Calcium signaling pathway (hsa04020),Dilated cardiomyopathy (hsa05414),Alzheimer's disease (hsa05010),Pancreatic secretion (hsa04972) |
| CAMK2G | ErbB signaling pathway (hsa04012),Dopaminergic synapse (hsa04728),Amphetamine addiction (hsa05031),Oocyte meiosis (hsa04114),Proteoglycans in cancer (hsa05205),Cholinergic synapse (hsa04725),Aldosterone synthesis and secretion (hsa04925),GnRH signaling pathway (hsa04912),Wnt signaling pathway (hsa04310),Insulin secretion (hsa04911),Melanogenesis (hsa04916),Glioma (hsa05214),Tuberculosis (hsa05152),Inflammatory mediator regulation of TRP channels (hsa04750),Oxytocin signaling pathway (hsa04921),Adrenergic signaling in cardiomyocytes (hsa04261),Long-term potentiation (hsa04720),Circadian entrainment (hsa04713),HIF-1 signaling pathway (hsa04066),Calcium signaling pathway (hsa04020),Olfactory transduction (hsa04740),Glucagon signaling pathway (hsa04922),Neurotrophin signaling pathway (hsa04722),cAMP signaling pathway (hsa04024),Gastric acid secretion (hsa04971) |
| CNNM3 | NA |
| CSDA | Tight junction (hsa04530) |
| CTSB | Metabolic pathways (hsa01100),Lysosome (hsa04142),Renin secretion (hsa04924),Glycosphingolipid biosynthesis - lacto and neolacto series (hsa00601),Antigen processing and presentation (hsa04612) |
| DNAJA1 | Protein processing in endoplasmic reticulum (hsa04141) |
| FAM134C | RNA degradation (hsa03018),Spliceosome (hsa03040) |
| FAM3C | NA |
| GTF2I | Platelet activation (hsa04611),Oxytocin signaling pathway (hsa04921),Vascular smooth muscle contraction (hsa04270),Regulation of actin cytoskeleton (hsa04810),Basal transcription factors (hsa03022),Focal adhesion (hsa04510),cGMP-PKG signaling pathway (hsa04022),cAMP signaling pathway (hsa04024),Proteoglycans in cancer (hsa05205),Herpes simplex infection (hsa05168) |
| HIST1H1C | Parkinson's disease (hsa05012),Alzheimer's disease (hsa05010),Metabolic pathways (hsa01100),Huntington's disease (hsa05016),Non-alcoholic fatty liver disease (NAFLD) (hsa04932),Oxidative phosphorylation (hsa00190) |
| HLA-B | Endocytosis (hsa04144),Phagosome (hsa04145),Cell adhesion molecules (CAMs) (hsa04514),Antigen processing and presentation (hsa04612),Natural killer cell mediated cytotoxicity (hsa04650),Type I diabetes mellitus (hsa04940),HTLV-I infection (hsa05166),Herpes simplex infection (hsa05168),Epstein-Barr virus infection (hsa05169),Viral carcinogenesis (Viral carcinogenesis (hsa05203)),Autoimmune thyroid disease (hsa05320),Allograft rejection (hsa05330),Graft-versus-host disease (hsa05332),Viral myocarditis (Viral myocarditis (hsa05416)) |
| KRT13 | NA |
| KRT75 | NA |
| MYH9 | Tight junction (hsa04530),Regulation of actin cytoskeleton (hsa04810),Salmonella infection (hsa05132) |
| NPEPPS | NA |
| PABPC1 | Hepatitis C (hsa05160),RNA degradation (hsa03018),mRNA surveillance pathway (hsa03015),RNA transport (hsa03013),Jak-STAT signaling pathway (hsa04630),Ubiquitin mediated proteolysis (hsa04120) |
| PCBP1 | ABC transporters (hsa02010),Bile secretion (hsa04976),Spliceosome (hsa03040) |
| RHBDD2 | NA |
| RPS15A | Ribosome (hsa03010) |
| SLC35E1 | NA |
| TFG | Thyroid cancer (hsa05216),Pathways in cancer (hsa05200) |
| TM9SF1 | NA |
| TPP1 | Nucleotide excision repair (hsa03420),Lysosome (hsa04142) |
| TRIM21 | Systemic lupus erythematosus (hsa05322) |
| TUBA4A | Phagosome (hsa04145),Gap junction (hsa04540),Pathogenic Escherichia coli infection (Pathogenic Escherichia coli infection (hsa05130)) |
| TUBB3 | Phagosome (hsa04145),Gap junction (hsa04540),Pathogenic Escherichia coli infection (Pathogenic Escherichia coli infection (hsa05130)) |
| VAT1 | NA |
| ZG16B | NA |

**Table 17**. **First connections of the GABARAPL2.** Table shows the first connections of the *GABARAPL2* gene within the network of up-regulated transcripts. Name gene and KEGG pathway in which gene is associated with, is indicated. NA= Not associated KEGG pathway.

| GEN | KEGG |
| --- | --- |
| ADAR | ABC transporters (hsa02010),Influenza A (hsa05164),Peroxisome (hsa04146),Measles (hsa05162),Cytosolic DNA-sensing pathway (hsa04623) |
| ANKFY1 | NA |
| ATG13 | Regulation of autophagy (hsa04140),Longevity regulating pathway - mammal (hsa04211) |
| CSDA | Tight junction (hsa04530) |
| CTSA | Lysosome (hsa04142),Renin-angiotensin system (hsa04614) |
| DDX24 | NA |
| DNAJA1 | Protein processing in endoplasmic reticulum (hsa04141) |
| EIF2AK2 | Natural killer cell mediated cytotoxicity (hsa04650),Measles (hsa05162),Hepatitis C (hsa05160),Protein processing in endoplasmic reticulum (hsa04141),Herpes simplex infection (hsa05168),Epstein-Barr virus infection (hsa05169),Viral carcinogenesis (Viral carcinogenesis (hsa05203)),Influenza A (hsa05164) |
| EIF4G1 | RNA transport (hsa03013),Viral myocarditis (Viral myocarditis (hsa05416)) |
| FYCO1 | NA |
| GABARAPL1 | Regulation of autophagy (hsa04140),FoxO signaling pathway (hsa04068),Salivary secretion (hsa04970),Measles (hsa05162),Natural killer cell mediated cytotoxicity (hsa04650),GABAergic synapse (hsa04727) |
| HIST1H1C | Parkinson's disease (hsa05012),Alzheimer's disease (hsa05010),Metabolic pathways (hsa01100),Huntington's disease (hsa05016),Non-alcoholic fatty liver disease (NAFLD) (hsa04932),Oxidative phosphorylation (hsa00190) |
| HIST1H2BL | Alcoholism (hsa05034),Systemic lupus erythematosus (hsa05322),Viral carcinogenesis (Viral carcinogenesis (hsa05203)) |
| HSPA2 | Measles (hsa05162),Legionellosis (hsa05134),Antigen processing and presentation (hsa04612),Estrogen signaling pathway (hsa04915),Toxoplasmosis (hsa05145),MAPK signaling pathway (hsa04010),Spliceosome (hsa03040),Endocytosis (hsa04144),Influenza A (hsa05164),Protein processing in endoplasmic reticulum (hsa04141),Epstein-Barr virus infection (hsa05169) |
| HSPA6 | Estrogen signaling pathway (hsa04915),Toxoplasmosis (hsa05145),Legionellosis (hsa05134),Antigen processing and presentation (hsa04612),Measles (hsa05162),Endocytosis (hsa04144),Influenza A (hsa05164),Epstein-Barr virus infection (hsa05169),Protein processing in endoplasmic reticulum (hsa04141),MAPK signaling pathway (hsa04010),Spliceosome (hsa03040) |
| KPNA6 | NA |
| KRT13 | NA |
| KRT75 | NA |
| LARP1 | NA |
| LRSAM1 | NA |
| MAP1LC3A | NA |
| MAP1LC3B | NA |
| PABPC1 | Hepatitis C (hsa05160),RNA degradation (hsa03018),mRNA surveillance pathway (hsa03015),RNA transport (hsa03013),Jak-STAT signaling pathway (hsa04630),Ubiquitin mediated proteolysis (hsa04120) |
| PCBP1 | ABC transporters (hsa02010),Bile secretion (hsa04976),Spliceosome (hsa03040) |
| RAB11FIP5 | Endocytosis (hsa04144) |
| RANGAP1 | RNA transport (hsa03013) |
| RNF123 | NA |
| RPS15A | Ribosome (hsa03010) |
| RPTN | NA |
| RRP1 | NA |
| SAFB2 | NA |
| SF1 | NA |
| SMARCA4 | Influenza A (hsa05164) |
| SQSTM1 | Osteoclast differentiation (hsa04380) |
| TECPR2 | NA |
| TFG | Thyroid cancer (hsa05216),Pathways in cancer (hsa05200) |
| TP53INP2 | NA |
| TRIM21 | Systemic lupus erythematosus (hsa05322) |
| TUBA4A | Phagosome (hsa04145),Gap junction (hsa04540),Pathogenic Escherichia coli infection (Pathogenic Escherichia coli infection (hsa05130)) |
| UBE2R2 | Ubiquitin mediated proteolysis (hsa04120),Herpes simplex infection (hsa05168) |
| ULK1 | mTOR signaling pathway (hsa04150),Regulation of autophagy (hsa04140),AMPK signaling pathway (hsa04152) |
| WBP2 | NA |

**Table** **18**. **First connections of the *MAP1LC3A*.** Table shows the first connections of the *MAP1LC3A* gene within the network of down-regulated transcripts. Name gene and KEGG pathway in which gene is associated with, is indicated. NA= Not associated KEGG pathway.

| GEN | KEGG |
| --- | --- |
| ADAR | ABC transporters (hsa02010),Influenza A (hsa05164),Peroxisome (hsa04146),Measles (hsa05162),Cytosolic DNA-sensing pathway (hsa04623) |
| CSDA | Tight junction (hsa04530) |
| DNAJA1 | Protein processing in endoplasmic reticulum (hsa04141) |
| FBXW11 | Hedgehog signaling pathway (hsa04340),Ubiquitin mediated proteolysis (hsa04120),Wnt signaling pathway (hsa04310),Hippo signaling pathway (hsa04390),Shigellosis (hsa05131),Oocyte meiosis (hsa04114),Circadian rhythm (hsa04710) |
| FYCO1 | NA |
| GABARAPL2 | Regulation of autophagy (hsa04140),FoxO signaling pathway (hsa04068),GABAergic synapse (hsa04727) |
| GAK | NA |
| GDI1 | NA |
| GTF2I | Platelet activation (hsa04611),Oxytocin signaling pathway (hsa04921),Vascular smooth muscle contraction (hsa04270),Regulation of actin cytoskeleton (hsa04810),Basal transcription factors (hsa03022),Focal adhesion (hsa04510),cGMP-PKG signaling pathway (hsa04022),cAMP signaling pathway (hsa04024),Proteoglycans in cancer (hsa05205),Herpes simplex infection (hsa05168) |
| GTPBP1 | Lysosome (hsa04142),Glycosaminoglycan degradation (hsa00531),Metabolic pathways (hsa01100) |
| HIST1H1C | Parkinson's disease (hsa05012),Alzheimer's disease (hsa05010),Metabolic pathways (hsa01100),Huntington's disease (hsa05016),Non-alcoholic fatty liver disease (NAFLD) (hsa04932),Oxidative phosphorylation (hsa00190) |
| HSPA2 | Measles (hsa05162),Legionellosis (hsa05134),Antigen processing and presentation (hsa04612),Estrogen signaling pathway (hsa04915),Toxoplasmosis (hsa05145),MAPK signaling pathway (hsa04010),Spliceosome (hsa03040),Endocytosis (hsa04144),Influenza A (hsa05164),Protein processing in endoplasmic reticulum (hsa04141),Epstein-Barr virus infection (hsa05169) |
| HSPA6 | Estrogen signaling pathway (hsa04915),Toxoplasmosis (hsa05145),Legionellosis (hsa05134),Antigen processing and presentation (hsa04612),Measles (hsa05162),Endocytosis (hsa04144),Influenza A (hsa05164),Epstein-Barr virus infection (hsa05169),Protein processing in endoplasmic reticulum (hsa04141),MAPK signaling pathway (hsa04010),Spliceosome (hsa03040) |
| KRT13 | NA |
| KRT75 | NA |
| MAP1LC3B | NA |
| MAP7D1 | NA |
| PABPC1 | Hepatitis C (hsa05160),RNA degradation (hsa03018),mRNA surveillance pathway (hsa03015),RNA transport (hsa03013),Jak-STAT signaling pathway (hsa04630),Ubiquitin mediated proteolysis (hsa04120) |
| PARD6B | Endocytosis (hsa04144),Hippo signaling pathway (hsa04390),Rap1 signaling pathway (hsa04015),Tight junction (hsa04530) |
| PCBP1 | ABC transporters (hsa02010),Bile secretion (hsa04976),Spliceosome (hsa03040) |
| RPS15A | Ribosome (hsa03010) |
| RPTN | NA |
| RRP1 | NA |
| SAFB2 | NA |
| SEC16A | NA |
| SMARCA4 | Influenza A (hsa05164) |
| SQSTM1 | Osteoclast differentiation (hsa04380) |
| TFG | Thyroid cancer (hsa05216),Pathways in cancer (hsa05200) |
| TRIM21 | Systemic lupus erythematosus (hsa05322) |
| TUBA4A | Phagosome (hsa04145),Gap junction (hsa04540),Pathogenic Escherichia coli infection (Pathogenic Escherichia coli infection (hsa05130)) |
| WBP2 | NA |

**Table 19**. **First connections of the *MAPL1C3B*.** Table shows the first connections of the *MAPL1C3B* gene within the network of down-regulated transcripts. Name gene and KEGG pathway in which gene is associated with, is indicated. NA= Not associated KEGG pathway.

| GEN | KEGG |
| --- | --- |
| AKAP8L | Epstein-Barr virus infection (hsa05169) |
| ATG13 | Regulation of autophagy (hsa04140),Longevity regulating pathway - mammal (hsa04211) |
| CHERP | Spliceosome (hsa03040) |
| CSDA | Tight junction (hsa04530) |
| DDX24 | NA |
| DNAJA1 | Protein processing in endoplasmic reticulum (hsa04141) |
| EIF2AK2 | Natural killer cell mediated cytotoxicity (hsa04650),Measles (hsa05162),Hepatitis C (hsa05160),Protein processing in endoplasmic reticulum (hsa04141),Herpes simplex infection (hsa05168),Epstein-Barr virus infection (hsa05169),Viral carcinogenesis (Viral carcinogenesis (hsa05203)),Influenza A (hsa05164) |
| FBXW11 | Hedgehog signaling pathway (hsa04340),Ubiquitin mediated proteolysis (hsa04120),Wnt signaling pathway (hsa04310),Hippo signaling pathway (hsa04390),Shigellosis (hsa05131),Oocyte meiosis (hsa04114),Circadian rhythm (hsa04710) |
| FYCO1 | NA |
| GABARAPL2 | Regulation of autophagy (hsa04140),FoxO signaling pathway (hsa04068),GABAergic synapse (hsa04727) |
| GTPBP1 | Lysosome (hsa04142),Glycosaminoglycan degradation (hsa00531),Metabolic pathways (hsa01100) |
| HIST1H1C | Parkinson's disease (hsa05012),Alzheimer's disease (hsa05010),Metabolic pathways (hsa01100),Huntington's disease (hsa05016),Non-alcoholic fatty liver disease (NAFLD) (hsa04932),Oxidative phosphorylation (hsa00190) |
| HIST1H2BL | Alcoholism (hsa05034),Systemic lupus erythematosus (hsa05322),Viral carcinogenesis (Viral carcinogenesis (hsa05203)) |
| HSPA2 | Measles (hsa05162),Legionellosis (hsa05134),Antigen processing and presentation (hsa04612),Estrogen signaling pathway (hsa04915),Toxoplasmosis (hsa05145),MAPK signaling pathway (hsa04010),Spliceosome (hsa03040),Endocytosis (hsa04144),Influenza A (hsa05164),Protein processing in endoplasmic reticulum (hsa04141),Epstein-Barr virus infection (hsa05169) |
| HSPA6 | Estrogen signaling pathway (hsa04915),Toxoplasmosis (hsa05145),Legionellosis (hsa05134),Antigen processing and presentation (hsa04612),Measles (hsa05162),Endocytosis (hsa04144),Influenza A (hsa05164),Epstein-Barr virus infection (hsa05169),Protein processing in endoplasmic reticulum (hsa04141),MAPK signaling pathway (hsa04010),Spliceosome (hsa03040) |
| HTT | Thyroid hormone signaling pathway (hsa04919),Dorso-ventral axis formation (hsa04320),Huntington's disease (hsa05016),Prion diseases (hsa05020),MicroRNAs in cancer (hsa05206),Notch signaling pathway (hsa04330) |
| KPNA6 | NA |
| KRT13 | NA |
| KRT75 | NA |
| LARP1 | NA |
| MAP1LC3A | NA |
| MAP1LC3B | NA |
| PABPC1 | Hepatitis C (hsa05160),RNA degradation (hsa03018),mRNA surveillance pathway (hsa03015),RNA transport (hsa03013),Jak-STAT signaling pathway (hsa04630),Ubiquitin mediated proteolysis (hsa04120) |
| PCBP1 | ABC transporters (hsa02010),Bile secretion (hsa04976),Spliceosome (hsa03040) |
| PINK1 | Parkinson's disease (hsa05012) |
| RAB24 | TGF-beta signaling pathway (hsa04350),Endocytosis (hsa04144) |
| RNF123 | NA |
| RPS15A | Ribosome (hsa03010) |
| RPTN | NA |
| SAFB2 | NA |
| SH3GL1 | Endocytosis (hsa04144) |
| SMARCA4 | Influenza A (hsa05164) |
| SQSTM1 | Osteoclast differentiation (hsa04380) |
| TECPR1 | NA |
| TECPR2 | NA |
| TFG | Thyroid cancer (hsa05216),Pathways in cancer (hsa05200) |
| TRIM21 | Systemic lupus erythematosus (hsa05322) |
| TUBA4A | Phagosome (hsa04145),Gap junction (hsa04540),Pathogenic Escherichia coli infection (Pathogenic Escherichia coli infection (hsa05130)) |
| ULK1 | mTOR signaling pathway (hsa04150),Regulation of autophagy (hsa04140),AMPK signaling pathway (hsa04152) |

**Table 20. First connections of the *PABPC1*.** Table shows the first connections of the *PABPC1* gene within the network of down-regulated transcripts. Name gene and KEGG pathway in which gene is associated with, is indicated. NA= Not associated KEGG pathway.

| GEN | KEGG |
| --- | --- |
| ATG13 | Regulation of autophagy (hsa04140),Longevity regulating pathway - mammal (hsa04211) |
| ATXN2 | Fat digestion and absorption (hsa04975),Phospholipase D signaling pathway (hsa04072),Glycerophospholipid metabolism (hsa00564),Glycerolipid metabolism (hsa00561),Metabolic pathways (hsa01100) |
| CLN3 | Neurotrophin signaling pathway (hsa04722),Ras signaling pathway (hsa04014),Axon guidance (hsa04360),Chronic myeloid leukemia (hsa05220),Lysosome (hsa04142),Pathways in cancer (hsa05200),Pathogenic Escherichia coli infection (Pathogenic Escherichia coli infection (hsa05130)),Cell cycle (hsa04110),MicroRNAs in cancer (hsa05206),Nucleotide excision repair (hsa03420),ErbB signaling pathway (hsa04012),Shigellosis (hsa05131),Viral myocarditis (Viral myocarditis (hsa05416)) |
| DDIT3 | Protein processing in endoplasmic reticulum (hsa04141),MAPK signaling pathway (hsa04010),Transcriptional misregulation in cancer (hsa05202),Non-alcoholic fatty liver disease (NAFLD) (hsa04932) |
| EIF4G1 | RNA transport (hsa03013),Viral myocarditis (Viral myocarditis (hsa05416)) |
| FYCO1 | NA |
| GABARAPL1 | Regulation of autophagy (hsa04140),FoxO signaling pathway (hsa04068),Salivary secretion (hsa04970),Measles (hsa05162),Natural killer cell mediated cytotoxicity (hsa04650),GABAergic synapse (hsa04727) |
| GABARAPL2 | Regulation of autophagy (hsa04140),FoxO signaling pathway (hsa04068),GABAergic synapse (hsa04727) |
| HLA-B | Endocytosis (hsa04144),Phagosome (hsa04145),Cell adhesion molecules (CAMs) (hsa04514),Antigen processing and presentation (hsa04612),Natural killer cell mediated cytotoxicity (hsa04650),Type I diabetes mellitus (hsa04940),HTLV-I infection (hsa05166),Herpes simplex infection (hsa05168),Epstein-Barr virus infection (hsa05169),Viral carcinogenesis (Viral carcinogenesis (hsa05203)),Autoimmune thyroid disease (hsa05320),Allograft rejection (hsa05330),Graft-versus-host disease (hsa05332),Viral myocarditis (Viral myocarditis (hsa05416)) |
| KLHDC10 | NA |
| MAP1LC3A | NA |
| MAP1LC3B | NA |
| PCBP1 | ABC transporters (hsa02010),Bile secretion (hsa04976),Spliceosome (hsa03040) |
| PDPK1 | mTOR signaling pathway (hsa04150),Choline metabolism in cancer (hsa05231),Neurotrophin signaling pathway (hsa04722),Toxoplasmosis (hsa05145),Hepatitis C (hsa05160),Focal adhesion (hsa04510),Prostate cancer (hsa05215),Thyroid hormone signaling pathway (hsa04919),Insulin signaling pathway (hsa04910),Fc epsilon RI signaling pathway (hsa04664),PI3K-Akt signaling pathway (hsa04151),Sphingolipid signaling pathway (hsa04071),PPAR signaling pathway (hsa03320),Insulin resistance (hsa04931),Non-small cell lung cancer (hsa05223),Proteoglycans in cancer (hsa05205),Endometrial cancer (hsa05213),T cell receptor signaling pathway (hsa04660),AMPK signaling pathway (hsa04152),FoxO signaling pathway (hsa04068),Aldosterone-regulated sodium reabsorption (hsa04960) |
| RAB24 | TGF-beta signaling pathway (hsa04350),Endocytosis (hsa04144) |
| SMAD3 | Adherens junction (hsa04520),Signaling pathways regulating pluripotency of stem cells (hsa04550),HTLV-I infection (hsa05166),Pathways in cancer (hsa05200),Chronic myeloid leukemia (hsa05220),Hepatitis B (hsa05161),Chagas disease (American trypanosomiasis) (hsa05142),Endocytosis (hsa04144),Cell cycle (hsa04110),Pancreatic cancer (hsa05212),Wnt signaling pathway (hsa04310),TGF-beta signaling pathway (hsa04350),Colorectal cancer (hsa05210),FoxO signaling pathway (hsa04068),Hippo signaling pathway (hsa04390),Inflammatory bowel disease (hsa05321) |
| SOX7 | NA |
| SQSTM1 | Osteoclast differentiation (hsa04380) |
| TECPR1 | NA |
| TOB1 | RNA degradation (hsa03018) |
| TRAF2 | NF-kappa B signaling pathway (hsa04064),Pathways in cancer (hsa05200),Thyroid hormone signaling pathway (hsa04920),Sphingolipid signaling pathway (hsa04071),Non-alcoholic fatty liver disease (NAFLD) (hsa04932),Osteoclast differentiation (hsa04380),Hepatitis C (hsa05160),TNF signaling pathway (hsa04668),RIG-I-like receptor signaling pathway (hsa04622),MAPK signaling pathway (hsa04010),Small cell lung cancer (hsa05222),Apoptosis (hsa04210),Viral carcinogenesis (Viral carcinogenesis (hsa05203)),Epstein-Barr virus infection (hsa05169),Protein processing in endoplasmic reticulum (hsa04141),Herpes simplex infection (hsa05168) |
| ULK1 | mTOR signaling pathway (hsa04150),Regulation of autophagy (hsa04140),AMPK signaling pathway (hsa04152) |
| WIPI2 | NA |
| YWHAG | Cell cycle (hsa04110),Viral carcinogenesis (Viral carcinogenesis (hsa05203)),Epstein-Barr virus infection (hsa05169),Oocyte meiosis (hsa04114),Hippo signaling pathway (hsa04390),PI3K-Akt signaling pathway (hsa04151) |

**Table 21.** **First connections of the SMAD3.** Table shows the first connections of the *SMAD3* gene within the network of down-regulated transcripts. Name gene and KEGG pathway in which gene is associated with, is indicated. NA= Not associated KEGG pathway.

| GEN | KEGG |
| --- | --- |
| ATF3 | MAPK signaling pathway (hsa04010),Arrhythmogenic right ventricular cardiomyopathy (ARVC) (hsa05412),Cardiac muscle contraction (hsa04260),Adrenergic signaling in cardiomyocytes (hsa04261),Oxytocin signaling pathway (hsa04921),HTLV-I infection (hsa05166),Hypertrophic cardiomyopathy (HCM) (hsa05410),Dilated cardiomyopathy (hsa05414) |
| CXXC5 | NA |
| DAB2 | Endocytosis (hsa04144) |
| FOXO1 | Insulin resistance (hsa04931),AMPK signaling pathway (hsa04152),Transcriptional misregulation in cancer (hsa05202),FoxO signaling pathway (hsa04068),Glucagon signaling pathway (hsa04922),Prostate cancer (hsa05215),Thyroid hormone signaling pathway (hsa04919),Insulin signaling pathway (hsa04910),Pathways in cancer (hsa05200) |
| FOXO4 | Adrenergic signaling in cardiomyocytes (hsa04261),Cardiac muscle contraction (hsa04260),Ras signaling pathway (hsa04014),Dilated cardiomyopathy (hsa05414),Hypertrophic cardiomyopathy (HCM) (hsa05410),FoxO signaling pathway (hsa04068) |
| GGA1 | Lysosome (hsa04142) |
| GMEB1 | NA |
| GTF2IRD1 | Herpes simplex infection (hsa05168),cGMP-PKG signaling pathway (hsa04022),Basal transcription factors (hsa03022) |
| HEY1 | NA |
| JUNB | Ribosome (hsa03010),TNF signaling pathway (hsa04668),Osteoclast differentiation (hsa04380) |
| KDM2A | NA |
| KIAA1267 | NA |
| MAX | Terpenoid backbone biosynthesis (hsa00900),Glycosaminoglycan biosynthesis - chondroitin sulfate and dermatan sulfate (hsa00532),Pathogenic Escherichia coli infection (Pathogenic Escherichia coli infection (hsa05130)),Small cell lung cancer (hsa05222),beta-Alanine metabolism (hsa00410),Glutathione metabolism (hsa00480),Transcriptional misregulation in cancer (hsa05202),Arginine and proline metabolism (hsa00330),Cysteine and methionine metabolism (hsa00270),RIG-I-like receptor signaling pathway (hsa04622),MAPK signaling pathway (hsa04010),Cytosolic DNA-sensing pathway (hsa04623),Pathways in cancer (hsa05200) |
| MED15 | NA |
| MLL4 | Lysine degradation (hsa00310) |
| NCOA6 | NA |
| PABPC1 | Hepatitis C (hsa05160),RNA degradation (hsa03018),mRNA surveillance pathway (hsa03015),RNA transport (hsa03013),Jak-STAT signaling pathway (hsa04630),Ubiquitin mediated proteolysis (hsa04120) |
| PCDH1 | Vasopressin-regulated water reabsorption (hsa04962) |
| PIAS3 | Ubiquitin mediated proteolysis (hsa04120),Jak-STAT signaling pathway (hsa04630) |
| PML | Hippo signaling pathway (hsa04390),Transcriptional misregulation in cancer (hsa05202),Herpes simplex infection (hsa05168),Ubiquitin mediated proteolysis (hsa04120),Endocytosis (hsa04144),Influenza A (hsa05164),Acute myeloid leukemia (hsa05221),Pathways in cancer (hsa05200) |
| RAB11FIP5 | Endocytosis (hsa04144) |
| RUNX1 | Chemokine signaling pathway (hsa04062),GABAergic synapse (hsa04727),Chagas disease (American trypanosomiasis) (hsa05142),Circadian entrainment (hsa04713),Progesterone-mediated oocyte maturation (hsa04914),Pathways in cancer (hsa05200),Acute myeloid leukemia (hsa05221),Vascular smooth muscle contraction (hsa04270),Chronic myeloid leukemia (hsa05220),Long-term potentiation (hsa04720),Platelet activation (hsa04611),Adrenergic signaling in cardiomyocytes (hsa04261),Oxytocin signaling pathway (hsa04921),Inflammatory mediator regulation of TRP channels (hsa04750),HTLV-I infection (hsa05166),Amoebiasis (hsa05146),Gastric acid secretion (hsa04971),cAMP signaling pathway (hsa04024),Gap junction (hsa04540),Rap1 signaling pathway (hsa04015),Phospholipase D signaling pathway (hsa04072),Estrogen signaling pathway (hsa04915),Dilated cardiomyopathy (hsa05414),Thyroid hormone synthesis (hsa04918),cGMP-PKG signaling pathway (hsa04022),Morphine addiction (hsa05032),Calcium signaling pathway (hsa04020),Retrograde endocannabinoid signaling (hsa04723),Primary immunodeficiency (hsa05340),Purine metabolism (hsa00230),Aldosterone synthesis and secretion (hsa04925),Salivary secretion (hsa04970),Cholinergic synapse (hsa04725),Oocyte meiosis (hsa04114),Ovarian steroidogenesis (hsa04913),Regulation of lipolysis in adipocytes (hsa04923),Transcriptional misregulation in cancer (hsa05202),Neuroactive ligand-receptor interaction (hsa04080),Pancreatic secretion (hsa04972),Insulin secretion (hsa04911),Melanogenesis (hsa04916),Glutamatergic synapse (hsa04724),Bile secretion (hsa04976),GnRH signaling pathway (hsa04912) |
| RUNX3 | NA |
| SKI | NA |
| SP1 | Pantothenate and CoA biosynthesis (hsa00770),Pentose phosphate pathway (hsa00030),Ubiquinone and other terpenoid-quinone biosynthesis (hsa00130),Intestinal immune network for IgA production (hsa04672),Mucin type O-Glycan biosynthesis (hsa00512),Glycosphingolipid biosynthesis - lacto and neolacto series (hsa00601),Type I diabetes mellitus (hsa04940),Cardiac muscle contraction (hsa04260),Arachidonic acid metabolism (hsa00590),NOD-like receptor signaling pathway (hsa04621),Asthma (hsa05310),Acute myeloid leukemia (hsa05221),Melanoma (hsa05218),Fructose and mannose metabolism (hsa00051),Citrate cycle (TCA cycle) (hsa00020),Maturity onset diabetes of the young (hsa04950),Pyruvate metabolism (hsa00620),Propanoate metabolism (hsa00640),Nitrogen metabolism (hsa00910),Amino sugar and nucleotide sugar metabolism (hsa00520),Taste transduction (hsa04742),Homologous recombination (hsa03440),Other glycan degradation (hsa00511),Steroid hormone biosynthesis (hsa00140),One carbon pool by folate (hsa00670),Huntington's disease (hsa05016),Folate biosynthesis (hsa00790),D-Glutamine and D-glutamate metabolism (hsa00471),Sulfur metabolism (hsa00920),Thyroid cancer (hsa05216),Selenocompound metabolism (hsa00450),Basal transcription factors (hsa03022),Choline metabolism in cancer (hsa05231),mTOR signaling pathway (hsa04150),Cytosolic DNA-sensing pathway (hsa04623),Estrogen signaling pathway (hsa04915),Fatty acid metabolism (hsa00071),Hedgehog signaling pathway (hsa04340),Butanoate metabolism (hsa00650),Inositol phosphate metabolism (hsa00562),Aminoacyl-tRNA biosynthesis (hsa00970),Tyrosine metabolism (hsa00350),RNA degradation (hsa03018),Histidine metabolism (hsa00340),Transcriptional misregulation in cancer (hsa05202),Galactose metabolism (hsa00052),Primary bile acid biosynthesis (hsa00120),Vibrio cholerae infection (hsa05110),Retinol metabolism (hsa00830),Glycosaminoglycan biosynthesis - heparan sulfate and heparin (hsa00534),Aldosterone-regulated sodium reabsorption (hsa04960),Glycolysis and Gluconeogenesis (hsa00010),Lysine degradation (hsa00310),Autoimmune thyroid disease (hsa05320),SNARE interactions in vesicular transport (hsa04130),Glycerolipid metabolism (hsa00561),Starch and sucrose metabolism (hsa00500),Circadian rhythm (hsa04710),Nicotinate and nicotinamide metabolism (hsa00760),Glycosaminoglycan biosynthesis - keratan sulfate (hsa00533),Valine, leucine and isoleucine degradation (hsa00280),RIG-I-like receptor signaling pathway (hsa04622),Bladder cancer (hsa05219),Primary immunodeficiency (hsa05340),Riboflavin metabolism (hsa00740),N-Glycan biosynthesis (hsa00510),Endometrial cancer (hsa05213),Graft-versus-host disease (hsa05332),Alanine, aspartate and glutamate metabolism (hsa00250),Arginine and proline metabolism (hsa00330),Notch signaling pathway (hsa04330),Allograft rejection (hsa05330),Glycosaminoglycan biosynthesis - chondroitin sulfate and dermatan sulfate (hsa00532),Non-small cell lung cancer (hsa05223),Type II diabetes mellitus (hsa04930),Nucleotide excision repair (hsa03420),Phenylalanine metabolism (hsa00360),Ether lipid metabolism (hsa00565),DNA replication (hsa03030),Amyotrophic lateral sclerosis (ALS) (Amyotrophic lateral sclerosis (ALS) (hsa05014)),Pathogenic Escherichia coli infection (Pathogenic Escherichia coli infection (hsa05130)),Sphingolipid metabolism (hsa00600),Basal cell carcinoma (hsa05217),Base excision repair (hsa03410),TGF-beta signaling pathway (hsa04350),Drug metabolism - other enzymes (hsa00983),Renin-angiotensin system (hsa04614),Mismatch repair (hsa03430) |
| TFE3 | Transcriptional misregulation in cancer (hsa05202) |
| WWP2 | Ubiquitin mediated proteolysis (hsa04120) |

**Table 22.** **First connections of the *SQSTM1*.** Table shows the first connections of the *SQSTM1* gene within the network of down-regulated transcripts. Name gene and KEGG pathway in which gene is associated with, is indicated. NA= Not associated KEGG pathway.

| GEN | KEGG |
| --- | --- |
| BAG3 | Cytokine-cytokine receptor interaction (hsa04060) |
| C10orf2 | Metabolic pathways (hsa01100),Arginine and proline metabolism (hsa00330) |
| CALML3 | Phototransduction (hsa04744),Amphetamine addiction (hsa05031),Dopaminergic synapse (hsa04728),Oocyte meiosis (hsa04114),Alzheimer's disease (hsa05010),Aldosterone synthesis and secretion (hsa04925),Phosphatidylinositol signaling system (hsa04070),Salivary secretion (hsa04970),GnRH signaling pathway (hsa04912),Tuberculosis (hsa05152),Melanogenesis (hsa04916),Wnt signaling pathway (hsa04310),Glioma (hsa05214),Adrenergic signaling in cardiomyocytes (hsa04261),Renin secretion (hsa04924),Long-term potentiation (hsa04720),Inflammatory mediator regulation of TRP channels (hsa04750),Oxytocin signaling pathway (hsa04921),Circadian entrainment (hsa04713),Pertussis (hsa05133),Vascular smooth muscle contraction (hsa04270),Insulin signaling pathway (hsa04910),Calcium signaling pathway (hsa04020),Ras signaling pathway (hsa04014),Glucagon signaling pathway (hsa04922),Olfactory transduction (hsa04740),cGMP-PKG signaling pathway (hsa04022),Neurotrophin signaling pathway (hsa04722),cAMP signaling pathway (hsa04024),Gastric acid secretion (hsa04971),Alcoholism (hsa05034),Estrogen signaling pathway (hsa04915),Rap1 signaling pathway (hsa04015),Hedgehog signaling pathway (hsa04340) |
| CRNN | Cytokine-cytokine receptor interaction (hsa04060),Chemokine signaling pathway (hsa04062) |
| CSDA | Tight junction (hsa04530) |
| CTSB | Metabolic pathways (hsa01100),Lysosome (hsa04142),Renin secretion (hsa04924),Glycosphingolipid biosynthesis - lacto and neolacto series (hsa00601),Antigen processing and presentation (hsa04612) |
| DIP2B | NA |
| DNAJA1 | Protein processing in endoplasmic reticulum (hsa04141) |
| EPDR1 | NA |
| GABARAPL1 | Regulation of autophagy (hsa04140),FoxO signaling pathway (hsa04068),Salivary secretion (hsa04970),Measles (hsa05162),Natural killer cell mediated cytotoxicity (hsa04650),GABAergic synapse (hsa04727) |
| GABARAPL2 | Regulation of autophagy (hsa04140),FoxO signaling pathway (hsa04068),GABAergic synapse (hsa04727) |
| GPC4 | Wnt signaling pathway (hsa04310) |
| GTF2I | Platelet activation (hsa04611),Oxytocin signaling pathway (hsa04921),Vascular smooth muscle contraction (hsa04270),Regulation of actin cytoskeleton (hsa04810),Basal transcription factors (hsa03022),Focal adhesion (hsa04510),cGMP-PKG signaling pathway (hsa04022),cAMP signaling pathway (hsa04024),Proteoglycans in cancer (hsa05205),Herpes simplex infection (hsa05168) |
| GTPBP1 | Lysosome (hsa04142),Glycosaminoglycan degradation (hsa00531),Metabolic pathways (hsa01100) |
| HLA-B | Endocytosis (hsa04144),Phagosome (hsa04145),Cell adhesion molecules (CAMs) (hsa04514),Antigen processing and presentation (hsa04612),Natural killer cell mediated cytotoxicity (hsa04650),Type I diabetes mellitus (hsa04940),HTLV-I infection (hsa05166),Herpes simplex infection (hsa05168),Epstein-Barr virus infection (hsa05169),Viral carcinogenesis (Viral carcinogenesis (hsa05203)),Autoimmune thyroid disease (hsa05320),Allograft rejection (hsa05330),Graft-versus-host disease (hsa05332),Viral myocarditis (Viral myocarditis (hsa05416)) |
| HSPA6 | Estrogen signaling pathway (hsa04915),Toxoplasmosis (hsa05145),Legionellosis (hsa05134),Antigen processing and presentation (hsa04612),Measles (hsa05162),Endocytosis (hsa04144),Influenza A (hsa05164),Epstein-Barr virus infection (hsa05169),Protein processing in endoplasmic reticulum (hsa04141),MAPK signaling pathway (hsa04010),Spliceosome (hsa03040) |
| HTT | Thyroid hormone signaling pathway (hsa04919),Dorso-ventral axis formation (hsa04320),Huntington's disease (hsa05016),Prion diseases (hsa05020),MicroRNAs in cancer (hsa05206),Notch signaling pathway (hsa04330) |
| IKBKB | T cell receptor signaling pathway (hsa04660),MAPK signaling pathway (hsa04010),RIG-I-like receptor signaling pathway (hsa04622),Epithelial cell signaling in Helicobacter pylori infection (hsa05120),Shigellosis (hsa05131),FoxO signaling pathway (hsa04068),Toll-like receptor signaling pathway (hsa04620),Apoptosis (hsa04210),Influenza A (hsa05164),Pancreatic cancer (hsa05212),Small cell lung cancer (hsa05222),Herpes simplex infection (hsa05168),Type II diabetes mellitus (hsa04930),Insulin resistance (hsa04931),Epstein-Barr virus infection (hsa05169),MicroRNAs in cancer (hsa05206),Chemokine signaling pathway (hsa04062),Chagas disease (American trypanosomiasis) (hsa05142),Hepatitis B (hsa05161),PI3K-Akt signaling pathway (hsa04151),Pathways in cancer (hsa05200),Thyroid hormone signaling pathway (hsa04920),Insulin signaling pathway (hsa04910),Acute myeloid leukemia (hsa05221),NF-kappa B signaling pathway (hsa04064),Chronic myeloid leukemia (hsa05220),NOD-like receptor signaling pathway (hsa04621),B cell receptor signaling pathway (hsa04662),HTLV-I infection (hsa05166),Non-alcoholic fatty liver disease (NAFLD) (hsa04932),Neurotrophin signaling pathway (hsa04722),Osteoclast differentiation (hsa04380),Cytosolic DNA-sensing pathway (hsa04623),mTOR signaling pathway (hsa04150),Prostate cancer (hsa05215),TNF signaling pathway (hsa04668),Ras signaling pathway (hsa04014),Toxoplasmosis (hsa05145),Hepatitis C (hsa05160) |
| INPP5K | Insulin signaling pathway (hsa04910),Inositol phosphate metabolism (hsa00562),Phosphatidylinositol signaling system (hsa04070),Metabolic pathways (hsa01100) |
| KRT13 | NA |
| KRT19 | NA |
| KRT75 | NA |
| MAP1LC3A | NA |
| MAP1LC3B | NA |
| MAP7D1 | NA |
| MARK2 | NA |
| PABPC1 | Hepatitis C (hsa05160),RNA degradation (hsa03018),mRNA surveillance pathway (hsa03015),RNA transport (hsa03013),Jak-STAT signaling pathway (hsa04630),Ubiquitin mediated proteolysis (hsa04120) |
| PCBP1 | ABC transporters (hsa02010),Bile secretion (hsa04976),Spliceosome (hsa03040) |
| RAD54L2 | NA |
| RPS15A | Ribosome (hsa03010) |
| SEC16A | NA |
| SQSTM1 | Osteoclast differentiation (hsa04380) |
| TFG | Thyroid cancer (hsa05216),Pathways in cancer (hsa05200) |
| TPP1 | Nucleotide excision repair (hsa03420),Lysosome (hsa04142) |
| TRIM21 | Systemic lupus erythematosus (hsa05322) |
| TRIM63 | NA |
| TUBA4A | Phagosome (hsa04145),Gap junction (hsa04540),Pathogenic Escherichia coli infection (Pathogenic Escherichia coli infection (hsa05130)) |
| ULK1 | mTOR signaling pathway (hsa04150),Regulation of autophagy (hsa04140),AMPK signaling pathway (hsa04152) |
| WIPI2 | NA |
| YTHDF1 | NA |

**Table 23. First connections of the *TRAF2*.** Table shows the first connections of the *TRAF2* gene within the network of down-regulated transcripts. Name gene and KEGG pathway in which gene is associated with, is indicated. NA= Not associated KEGG pathway.

| GEN | KEGG |
| --- | --- |
| ACTG1 | Vibrio cholerae infection (hsa05110),Hippo signaling pathway (hsa04390),Viral myocarditis (Viral myocarditis (hsa05416)),Shigellosis (hsa05131),Proteoglycans in cancer (hsa05205),Salmonella infection (hsa05132),Pathogenic Escherichia coli infection (Pathogenic Escherichia coli infection (hsa05130)),Influenza A (hsa05164),Leukocyte transendothelial migration (hsa04670),Tight junction (hsa04530),Oxytocin signaling pathway (hsa04921),Platelet activation (hsa04611),Thyroid hormone signaling pathway (hsa04919),Arrhythmogenic right ventricular cardiomyopathy (ARVC) (hsa05412),Bacterial invasion of epithelial cells (hsa05100),Adherens junction (hsa04520),Focal adhesion (hsa04510),Dilated cardiomyopathy (hsa05414),Regulation of actin cytoskeleton (hsa04810),Rap1 signaling pathway (hsa04015),Hypertrophic cardiomyopathy (HCM) (hsa05410),Phagosome (hsa04145) |
| ATXN1 | NA |
| BAHD1 | NA |
| BANP | NA |
| CCDC130 | NA |
| CFLAR | Apoptosis (hsa04210),TNF signaling pathway (hsa04668),Chagas disease (American trypanosomiasis) (hsa05142),NF-kappa B signaling pathway (hsa04064) |
| DNAJA1 | Protein processing in endoplasmic reticulum (hsa04141) |
| EIF4G1 | RNA transport (hsa03013),Viral myocarditis (Viral myocarditis (hsa05416)) |
| GOLGA3 | NA |
| HINFP | NA |
| HIST1H1C | Parkinson's disease (hsa05012),Alzheimer's disease (hsa05010),Metabolic pathways (hsa01100),Huntington's disease (hsa05016),Non-alcoholic fatty liver disease (NAFLD) (hsa04932),Oxidative phosphorylation (hsa00190) |
| IKBKB | T cell receptor signaling pathway (hsa04660),MAPK signaling pathway (hsa04010),RIG-I-like receptor signaling pathway (hsa04622),Epithelial cell signaling in Helicobacter pylori infection (hsa05120),Shigellosis (hsa05131),FoxO signaling pathway (hsa04068),Toll-like receptor signaling pathway (hsa04620),Apoptosis (hsa04210),Influenza A (hsa05164),Pancreatic cancer (hsa05212),Small cell lung cancer (hsa05222),Herpes simplex infection (hsa05168),Type II diabetes mellitus (hsa04930),Insulin resistance (hsa04931),Epstein-Barr virus infection (hsa05169),MicroRNAs in cancer (hsa05206),Chemokine signaling pathway (hsa04062),Chagas disease (American trypanosomiasis) (hsa05142),Hepatitis B (hsa05161),PI3K-Akt signaling pathway (hsa04151),Pathways in cancer (hsa05200),Thyroid hormone signaling pathway (hsa04920),Insulin signaling pathway (hsa04910),Acute myeloid leukemia (hsa05221),NF-kappa B signaling pathway (hsa04064),Chronic myeloid leukemia (hsa05220),NOD-like receptor signaling pathway (hsa04621),B cell receptor signaling pathway (hsa04662),HTLV-I infection (hsa05166),Non-alcoholic fatty liver disease (NAFLD) (hsa04932),Neurotrophin signaling pathway (hsa04722),Osteoclast differentiation (hsa04380),Cytosolic DNA-sensing pathway (hsa04623),mTOR signaling pathway (hsa04150),Prostate cancer (hsa05215),TNF signaling pathway (hsa04668),Ras signaling pathway (hsa04014),Toxoplasmosis (hsa05145),Hepatitis C (hsa05160) |
| KIAA1267 | NA |
| KRT13 | NA |
| KRT75 | NA |
| LNX1 | Signaling pathways regulating pluripotency of stem cells (hsa04550),Hippo signaling pathway (hsa04390) |
| LTBR | Steroid hormone biosynthesis (hsa00140),Cytokine-cytokine receptor interaction (hsa04060),NF-kappa B signaling pathway (hsa04064),HIF-1 signaling pathway (hsa04066),HTLV-I infection (hsa05166),Intestinal immune network for IgA production (hsa04672),Viral carcinogenesis (Viral carcinogenesis (hsa05203)),Metabolic pathways (hsa01100),Primary bile acid biosynthesis (hsa00120) |
| LZTS2 | NA |
| PABPC1 | Hepatitis C (hsa05160),RNA degradation (hsa03018),mRNA surveillance pathway (hsa03015),RNA transport (hsa03013),Jak-STAT signaling pathway (hsa04630),Ubiquitin mediated proteolysis (hsa04120) |
| TFG | Thyroid cancer (hsa05216),Pathways in cancer (hsa05200) |
| TNFRSF11A | Osteoclast differentiation (hsa04380),Rheumatoid arthritis (hsa05323),Prolactin signaling pathway (hsa04917),NF-kappa B signaling pathway (hsa04064),Cytokine-cytokine receptor interaction (hsa04060) |
| TNFRSF14 | Cytokine-cytokine receptor interaction (hsa04060),Herpes simplex infection (hsa05168) |
| TRAF3 | Pathways in cancer (hsa05200),NF-kappa B signaling pathway (hsa04064),Hepatitis C (hsa05160),TNF signaling pathway (hsa04668),RIG-I-like receptor signaling pathway (hsa04622),Toll-like receptor signaling pathway (hsa04620),Small cell lung cancer (hsa05222),Viral carcinogenesis (Viral carcinogenesis (hsa05203)),Epstein-Barr virus infection (hsa05169),Herpes simplex infection (hsa05168) |
| TUBA4A | Phagosome (hsa04145),Gap junction (hsa04540),Pathogenic Escherichia coli infection (Pathogenic Escherichia coli infection (hsa05130)) |
| USP31 | NA |
| ZBTB43 | NA |
| ZNF646 | NA |

**Table 24. First connections of the *WIPI2*.** Table shows the first connections of the *WIPI2* gene within the network of down-regulated transcripts. Name gene and KEGG pathway in which gene is associated with, is indicated. NA= Not associated KEGG pathway.

| GEN | KEGG |
| --- | --- |
| ADAR | ABC transporters (hsa02010),Influenza A (hsa05164),Peroxisome (hsa04146),Measles (hsa05162),Cytosolic DNA-sensing pathway (hsa04623) |
| AGPAT6 | Glycerolipid metabolism (hsa00561),Metabolic pathways (hsa01100),Glycerophospholipid metabolism (hsa00564),Ether lipid metabolism (hsa00565) |
| AKAP8L | Epstein-Barr virus infection (hsa05169) |
| ATP2A2 | Arrhythmogenic right ventricular cardiomyopathy (ARVC) (hsa05412),Thyroid hormone signaling pathway (hsa04919),Cardiac muscle contraction (hsa04260),Adrenergic signaling in cardiomyocytes (hsa04261),Hypertrophic cardiomyopathy (HCM) (hsa05410),cAMP signaling pathway (hsa04024),cGMP-PKG signaling pathway (hsa04022),Calcium signaling pathway (hsa04020),Dilated cardiomyopathy (hsa05414),Alzheimer's disease (hsa05010),Pancreatic secretion (hsa04972) |
| BAG3 | Cytokine-cytokine receptor interaction (hsa04060) |
| CAMK2G | ErbB signaling pathway (hsa04012),Dopaminergic synapse (hsa04728),Amphetamine addiction (hsa05031),Oocyte meiosis (hsa04114),Proteoglycans in cancer (hsa05205),Cholinergic synapse (hsa04725),Aldosterone synthesis and secretion (hsa04925),GnRH signaling pathway (hsa04912),Wnt signaling pathway (hsa04310),Insulin secretion (hsa04911),Melanogenesis (hsa04916),Glioma (hsa05214),Tuberculosis (hsa05152),Inflammatory mediator regulation of TRP channels (hsa04750),Oxytocin signaling pathway (hsa04921),Adrenergic signaling in cardiomyocytes (hsa04261),Long-term potentiation (hsa04720),Circadian entrainment (hsa04713),HIF-1 signaling pathway (hsa04066),Calcium signaling pathway (hsa04020),Olfactory transduction (hsa04740),Glucagon signaling pathway (hsa04922),Neurotrophin signaling pathway (hsa04722),cAMP signaling pathway (hsa04024),Gastric acid secretion (hsa04971) |
| CTSB | Metabolic pathways (hsa01100),Lysosome (hsa04142),Renin secretion (hsa04924),Glycosphingolipid biosynthesis - lacto and neolacto series (hsa00601),Antigen processing and presentation (hsa04612) |
| CTSD | Metabolic pathways (hsa01100),Tuberculosis (hsa05152),Glycosphingolipid biosynthesis - lacto and neolacto series (hsa00601),Sphingolipid signaling pathway (hsa04071),Other types of O-glycan biosynthesis (hsa00514),Lysosome (hsa04142) |
| DNAJA1 | Protein processing in endoplasmic reticulum (hsa04141) |
| DNAJB2 | Protein processing in endoplasmic reticulum (hsa04141),PI3K-Akt signaling pathway (hsa04151),Pathways in cancer (hsa05200),Focal adhesion (hsa04510),Ras signaling pathway (hsa04014),Rap1 signaling pathway (hsa04015) |
| ELF3 | NA |
| HLA-B | Endocytosis (hsa04144),Phagosome (hsa04145),Cell adhesion molecules (CAMs) (hsa04514),Antigen processing and presentation (hsa04612),Natural killer cell mediated cytotoxicity (hsa04650),Type I diabetes mellitus (hsa04940),HTLV-I infection (hsa05166),Herpes simplex infection (hsa05168),Epstein-Barr virus infection (hsa05169),Viral carcinogenesis (Viral carcinogenesis (hsa05203)),Autoimmune thyroid disease (hsa05320),Allograft rejection (hsa05330),Graft-versus-host disease (hsa05332),Viral myocarditis (Viral myocarditis (hsa05416)) |
| HSPA2 | Measles (hsa05162),Legionellosis (hsa05134),Antigen processing and presentation (hsa04612),Estrogen signaling pathway (hsa04915),Toxoplasmosis (hsa05145),MAPK signaling pathway (hsa04010),Spliceosome (hsa03040),Endocytosis (hsa04144),Influenza A (hsa05164),Protein processing in endoplasmic reticulum (hsa04141),Epstein-Barr virus infection (hsa05169) |
| HSPA6 | Estrogen signaling pathway (hsa04915),Toxoplasmosis (hsa05145),Legionellosis (hsa05134),Antigen processing and presentation (hsa04612),Measles (hsa05162),Endocytosis (hsa04144),Influenza A (hsa05164),Epstein-Barr virus infection (hsa05169),Protein processing in endoplasmic reticulum (hsa04141),MAPK signaling pathway (hsa04010),Spliceosome (hsa03040) |
| KRT13 | NA |
| KRT75 | NA |
| NPEPPS | NA |
| PABPC1 | Hepatitis C (hsa05160),RNA degradation (hsa03018),mRNA surveillance pathway (hsa03015),RNA transport (hsa03013),Jak-STAT signaling pathway (hsa04630),Ubiquitin mediated proteolysis (hsa04120) |
| PCBP1 | ABC transporters (hsa02010),Bile secretion (hsa04976),Spliceosome (hsa03040) |
| RANGAP1 | RNA transport (hsa03013) |
| RPS15A | Ribosome (hsa03010) |
| RPTN | NA |
| S100A7 | Synaptic vesicle cycle (hsa04721) |
| SDF4 | NA |
| SEC16A | NA |
| SF1 | NA |
| SMARCA4 | Influenza A (hsa05164) |
| SQSTM1 | Osteoclast differentiation (hsa04380) |
| STAT3 | Pancreatic cancer (hsa05212),Viral carcinogenesis (Viral carcinogenesis (hsa05203)),MicroRNAs in cancer (hsa05206),Epstein-Barr virus infection (hsa05169),Insulin resistance (hsa04931),Inflammatory bowel disease (hsa05321),Proteoglycans in cancer (hsa05205),FoxO signaling pathway (hsa04068),Signaling pathways regulating pluripotency of stem cells (hsa04550),Hepatitis C (hsa05160),Toxoplasmosis (hsa05145),Acute myeloid leukemia (hsa05221),Thyroid hormone signaling pathway (hsa04920),Pathways in cancer (hsa05200),Prolactin signaling pathway (hsa04917),Chemokine signaling pathway (hsa04062),Jak-STAT signaling pathway (hsa04630),HIF-1 signaling pathway (hsa04066),Hepatitis B (hsa05161),Measles (hsa05162) |
| TFG | Thyroid cancer (hsa05216),Pathways in cancer (hsa05200) |
| TPP1 | Nucleotide excision repair (hsa03420),Lysosome (hsa04142) |
| TRIM21 | Systemic lupus erythematosus (hsa05322) |
| TUBA4A | Phagosome (hsa04145),Gap junction (hsa04540),Pathogenic Escherichia coli infection (Pathogenic Escherichia coli infection (hsa05130)) |
| TUBB3 | Phagosome (hsa04145),Gap junction (hsa04540),Pathogenic Escherichia coli infection (Pathogenic Escherichia coli infection (hsa05130)) |
| YWHAG | Cell cycle (hsa04110),Viral carcinogenesis (Viral carcinogenesis (hsa05203)),Epstein-Barr virus infection (hsa05169),Oocyte meiosis (hsa04114),Hippo signaling pathway (hsa04390),PI3K-Akt signaling pathway (hsa04151) |

**Table 25. First connections of the YWHAG.** Table shows the first connections of the *YWHAG* gene within the network of down-regulated transcripts. Name gene and KEGG pathway in which gene is associated with, is indicated. NA= Not associated KEGG pathway.

| GEN | KEGG |
| --- | --- |
| AKAP13 | NA |
| ANKS1A | NA |
| BRAF | Rap1 signaling pathway (hsa04015),Renal cell carcinoma (hsa05211),Alcoholism (hsa05034),mTOR signaling pathway (hsa04150),cAMP signaling pathway (hsa04024),Neurotrophin signaling pathway (hsa04722),Hepatitis C (hsa05160),Thyroid cancer (hsa05216),Focal adhesion (hsa04510),Prostate cancer (hsa05215),Regulation of actin cytoskeleton (hsa04810),Chronic myeloid leukemia (hsa05220),Vascular smooth muscle contraction (hsa04270),Insulin signaling pathway (hsa04910),Acute myeloid leukemia (hsa05221),Melanoma (hsa05218),Pathways in cancer (hsa05200),Serotonergic synapse (hsa04726),Progesterone-mediated oocyte maturation (hsa04914),Chemokine signaling pathway (hsa04062),Natural killer cell mediated cytotoxicity (hsa04650),Long-term potentiation (hsa04720),Long-term depression (hsa04730),Pancreatic cancer (hsa05212),Glioma (hsa05214),Non-small cell lung cancer (hsa05223),Proteoglycans in cancer (hsa05205),MAPK signaling pathway (hsa04010),Endometrial cancer (hsa05213),Bladder cancer (hsa05219),ErbB signaling pathway (hsa04012),FoxO signaling pathway (hsa04068),Colorectal cancer (hsa05210) |
| CBL | ErbB signaling pathway (hsa04012),T cell receptor signaling pathway (hsa04660),Proteoglycans in cancer (hsa05205),Ubiquitin mediated proteolysis (hsa04120),Endocytosis (hsa04144),Chronic myeloid leukemia (hsa05220),Pathways in cancer (hsa05200),Insulin signaling pathway (hsa04910),Bacterial invasion of epithelial cells (hsa05100) |
| CGNL1 | NA |
| CRTC2 | AMPK signaling pathway (hsa04152),Base excision repair (hsa03410),Insulin resistance (hsa04931),PI3K-Akt signaling pathway (hsa04151),HTLV-I infection (hsa05166),Glucagon signaling pathway (hsa04922) |
| CRTC3 | HTLV-I infection (hsa05166) |
| DFFA | Apoptosis (hsa04210) |
| EML3 | NA |
| FOXO1 | Insulin resistance (hsa04931),AMPK signaling pathway (hsa04152),Transcriptional misregulation in cancer (hsa05202),FoxO signaling pathway (hsa04068),Glucagon signaling pathway (hsa04922),Prostate cancer (hsa05215),Thyroid hormone signaling pathway (hsa04919),Insulin signaling pathway (hsa04910),Pathways in cancer (hsa05200) |
| GSK3A | Cell cycle (hsa04110),MAPK signaling pathway (hsa04010),FoxO signaling pathway (hsa04068),Dopaminergic synapse (hsa04728),Non-alcoholic fatty liver disease (NAFLD) (hsa04932),NF-kappa B signaling pathway (hsa04064),p53 signaling pathway (hsa04115),Chemokine signaling pathway (hsa04062) |
| HIVEP2 | NA |
| IGF1R | Pathways in cancer (hsa05200),Melanoma (hsa05218),Thyroid hormone signaling pathway (hsa04920),PI3K-Akt signaling pathway (hsa04151),Progesterone-mediated oocyte maturation (hsa04914),HIF-1 signaling pathway (hsa04066),Long-term depression (hsa04730),Rap1 signaling pathway (hsa04015),Signaling pathways regulating pluripotency of stem cells (hsa04550),Non-alcoholic fatty liver disease (NAFLD) (hsa04932),cAMP signaling pathway (hsa04024),Adherens junction (hsa04520),Glucagon signaling pathway (hsa04922),Ras signaling pathway (hsa04014),Hepatitis C (hsa05160),Focal adhesion (hsa04510),Prostate cancer (hsa05215),Proteoglycans in cancer (hsa05205),Transcriptional misregulation in cancer (hsa05202),AMPK signaling pathway (hsa04152),Oocyte meiosis (hsa04114),FoxO signaling pathway (hsa04068),Ovarian steroidogenesis (hsa04913),Glioma (hsa05214),Endocytosis (hsa04144),Insulin resistance (hsa04931),PPAR signaling pathway (hsa03320) |
| KIAA0930 | NA |
| LARP1 | NA |
| LSR | NA |
| MARK2 | NA |
| MICALL1 | NA |
| NEDD4L | Ubiquitin mediated proteolysis (hsa04120),Endocytosis (hsa04144),Aldosterone-regulated sodium reabsorption (hsa04960) |
| PABPC1 | Hepatitis C (hsa05160),RNA degradation (hsa03018),mRNA surveillance pathway (hsa03015),RNA transport (hsa03013),Jak-STAT signaling pathway (hsa04630),Ubiquitin mediated proteolysis (hsa04120) |
| PRKCD | Chemokine signaling pathway (hsa04062),Vascular smooth muscle contraction (hsa04270),Tight junction (hsa04530),Inflammatory mediator regulation of TRP channels (hsa04750),Neurotrophin signaling pathway (hsa04722),Estrogen signaling pathway (hsa04915),Fc gamma R-mediated phagocytosis (hsa04666),Type II diabetes mellitus (hsa04930),Insulin resistance (hsa04931),GnRH signaling pathway (hsa04912) |
| RAI14 | NA |
| SH3BP5L | NA |
| SMCR7L | NA |
| SYNPO | NA |
| TMEM102 | NA |
| TRIM21 | Systemic lupus erythematosus (hsa05322) |
| TSC2 | Chemical carcinogenesis (hsa05204),Endocytosis (hsa04144),Glutamatergic synapse (hsa04724),Metabolic pathways (hsa01100),Glycolysis and Gluconeogenesis (hsa00010),Retinol metabolism (hsa00830),AMPK signaling pathway (hsa04152),Tyrosine metabolism (hsa00350),Fatty acid metabolism (hsa00071),Phospholipase D signaling pathway (hsa04072),mTOR signaling pathway (hsa04150),Choline metabolism in cancer (hsa05231),Drug metabolism - cytochrome P450 (hsa00982),Morphine addiction (hsa05032),p53 signaling pathway (hsa04115),PI3K-Akt signaling pathway (hsa04151),Metabolism of xenobiotics by cytochrome P450 (hsa00980),Chemokine signaling pathway (hsa04062),Thyroid hormone signaling pathway (hsa04919),Insulin signaling pathway (hsa04910) |
| WIPI2 | NA |
