# Supplementary material for: Genomic expression differences between cutaneous cells from red hair color individuals and black hair color individuals based on bioinformatic analysis
Source: Oncotarget. 2016 Dec 24;8(7):11589–99. doi: 10.18632/oncotarget.14140 (PMC5355288; doi:10.18632/oncotarget.14140)
Supplement: Supplementary file 3 [file oncotarget-08-11589-s003.docx]

**SUPPLEMENTARY TABLE**

Table 26: List of differentially expressed genes in healthy skin from Red hair color individuals vs Black hair color individuals. Table contains Log2 Fold Change (logFc), P-Value and adjusted P-value ( adj.P-Val) for each gene.

| **Gene Name** | **logFC** | **P-Value** | **adj.P-Val** |
| --- | --- | --- | --- |
| SLC5A8 | -0.85257554713072 | 0.00427325696572766 | 0.0604869349077403 |
| ITPRIPL2 | -0.823113533572678 | 0.0109612733246618 | 0.0967129608577777 |
| EIF2C2 | -0.818759468283935 | 0.00111125619940126 | 0.0327204957769608 |
| GNL3L | -0.80993337083513 | 0.000794186285429586 | 0.029106626873633 |
| SYNJ2BP | -0.809268829781875 | 0.000644745932253538 | 0.0278046472365304 |
| RSPRY1 | -0.761669765397495 | 0.000161187958645493 | 0.0239757643173249 |
| PDPR | -0.744482726197432 | 6,92E+14 | 0.0239757643173249 |
| S100P | -0.721196608334363 | 0.00664888960469081 | 0.0743626983977651 |
| RNF165 | -0.705954890950299 | 0.000736726106745748 | 0.0287947341006642 |
| ZNF148 | -0.694778271037621 | 0.00726905195290495 | 0.0780612096308319 |
| PTGR2 | -0.687694695340837 | 0.0105269238389929 | 0.0950277517483059 |
| C9orf130 | -0.681562338519095 | 0.00717312969787872 | 0.0772955591829716 |
| CLDN5 | -0.661249060612869 | 0.00303154946826406 | 0.0520111234256005 |
| FAM107A | -0.659098485872645 | 0.0016721132565231 | 0.038604711883611 |
| PRICKLE1 | -0.653313033949086 | 0.00439100139777286 | 0.0608597912300807 |
| ZNF93 | -0.646518707302037 | 0.000779781914523079 | 0.029106626873633 |
| FAM129A | -0.646374115365722 | 0.000375696503472408 | 0.024952349438868 |
| NIPAL1 | -0.634572792626537 | 0.0015210271500221 | 0.0372715043388988 |
| TGFBRAP1 | -0.630591743248227 | 0.00023159481387817 | 0.0239757643173249 |
| ATXN3 | -0.622690980276803 | 0.00678717055776044 | 0.0750692631923457 |
| BRD3 | -0.621753214699743 | 0.0001735403332174 | 0.0239757643173249 |
| EVI5 | -0.621545153990304 | 0.00819934258530658 | 0.0827442748264649 |
| HOXC9 | -0.620947889920183 | 0.000355670906106137 | 0.0248077604528682 |
| EXPH5 | -0.615644548865646 | 0.00459136948743355 | 0.0615361807470361 |
| TNPO3 | -0.615162334882372 | 0.000801668651694875 | 0.029106626873633 |
| ZNF324 | -0.593277537020801 | 0.000291203963334146 | 0.0247792613550641 |
| AHDC1 | -0.582796796771471 | 3,97E+14 | 0.0239757643173249 |
| PIAS4 | -0.582781050670674 | 0.000195610566891892 | 0.0239757643173249 |
| TMEM45B | -0.574243843773457 | 0.00199232137818573 | 0.0418397471995718 |
| PHKA2 | -0.564617261395188 | 0.00198260481024759 | 0.0418281871627141 |
| BGN | -0.562564152492059 | 0.00195569180853002 | 0.0416086425260897 |
| PEG3 | -0.560781504789608 | 0.00149807288361432 | 0.0371085137211963 |
| ZNF364 | -0.559184898348369 | 0.000777574595698878 | 0.029106626873633 |
| HSD11B2 | -0.558884021589612 | 0.00877331781370652 | 0.0861088728342917 |
| WDR73 | -0.555630053363439 | 0.000557120704841774 | 0.0275377535242345 |
| ZNF827 | -0.554657247126835 | 0.000858558573351686 | 0.030150983147705 |
| RDH13 | -0.553746699972138 | 0.00207253611581381 | 0.042382599734443 |
| SRGAP1 | -0.553705119067281 | 0.0003165558772965 | 0.0247792613550641 |
| GPR137 | -0.542901093641101 | 0.00472511341740883 | 0.0622596314598132 |
| UBN2 | -0.5390026204446 | 0.00195720095505997 | 0.0416086425260897 |
| COX19 | -0.536337075527216 | 0.00496864184831376 | 0.0636109406478712 |
| ZNF763 | -0.527361431841748 | 0.00037624893122407 | 0.024952349438868 |
| DCLRE1C | -0.523504652218628 | 0.00331990080340577 | 0.0538101935477045 |
| IGSF9B | -0.523242488107122 | 0.000213430191973712 | 0.0239757643173249 |
| C14orf173 | -0.523071658040523 | 0.00174861635143288 | 0.0393876424708413 |
| MIDN | -0.522627522486527 | 0.00127461499010131 | 0.034555767304929 |
| CARM1 | -0.52206304794641 | 0.0012063836837431 | 0.0335326583455989 |
| TMEM184A | -0.516569172862302 | 0.00912570752119496 | 0.0883206882574871 |
| ACTN4 | -0.513929990701874 | 0.000302260954404542 | 0.0247792613550641 |
| MGC12760 | -0.512752510779595 | 0.00091342517192009 | 0.0308415322754195 |
| CTSB | -0.50944080719782 | 0.000102637535809078 | 0.0239757643173249 |
| SCUBE2 | -0.508662040817803 | 0.000315080330748917 | 0.0247792613550641 |
| HSD3B7 | -0.504346697644799 | 0.000373247753313437 | 0.024952349438868 |
| ACVRL1 | -0.498942007026749 | 0.000392248119371705 | 0.0254060785800054 |
| CLIP3 | -0.498661306534056 | 0.000678321634025984 | 0.0280182181637631 |
| EHD1 | -0.498320600062105 | 0.000388938268038462 | 0.0253892800383068 |
| RNF150 | -0.491523367179036 | 0.00617003902861709 | 0.0712250136410264 |
| RHOQ | -0.489345283652978 | 0.00482861927303559 | 0.0628929549444057 |
| SLC15A3 | -0.485083327997072 | 0.000787681189278986 | 0.029106626873633 |
| P2RY11 | -0.48459521714362 | 0.00256508689282526 | 0.0475674471659167 |
| C21orf2 | -0.483330047720885 | 0.00854846404277256 | 0.0845897276567415 |
| ZNF689 | -0.482389885322871 | 0.000216469107383474 | 0.0239757643173249 |
| LRP3 | -0.478810701458498 | 0.000286617868839061 | 0.0247792613550641 |
| ZNF641 | -0.476801636287765 | 7,22E+14 | 0.0239757643173249 |
| RBPMS2 | -0.476475640303413 | 0.000877569383388887 | 0.0304962121691716 |
| P76 | -0.475092492183815 | 0.000745944310732691 | 0.028858331434898 |
| APM-1 | -0.475036657304558 | 0.0006269896365545 | 0.0278046472365304 |
| EEF1A2 | -0.474108776631397 | 0.00221979339639491 | 0.043728616421763 |
| HOXA6 | -0.473194202317825 | 0.000710050058019511 | 0.0284807066645609 |
| HSPA12B | -0.472898195030905 | 0.000902258916612425 | 0.0306331953292983 |
| WFS1 | -0.472706309560259 | 0.000129749373676251 | 0.0239757643173249 |
| MGC3020 | -0.471589269441391 | 0.000800784352586168 | 0.029106626873633 |
| CES2 | -0.471538830571002 | 5,29E+14 | 0.0239757643173249 |
| NINJ2 | -0.467751074214026 | 0.00394679585103357 | 0.0584505015447552 |
| ATG12 | -0.466677514438257 | 0.000864255078257491 | 0.0302235084720046 |
| C11orf2 | -0.465604832843755 | 0.00206750855993781 | 0.0423491468277786 |
| PLAC2 | -0.464973867464072 | 0.000736907168501919 | 0.0287947341006642 |
| CBX6 | -0.464257452056485 | 1,96E+14 | 0.0239757643173249 |
| KCTD7 | -0.462271453045833 | 0.000632742366305429 | 0.0278046472365304 |
| CBLB | -0.46134216280301 | 1,52E+14 | 0.0239757643173249 |
| NEK8 | -0.460654274358256 | 0.000714202953922915 | 0.0285486269019627 |
| TMEM137 | -0.458972807607566 | 0.00953749234566599 | 0.0903254705065237 |
| GGA2 | -0.458754473523497 | 0.000661458627037489 | 0.0278046472365304 |
| FLOT2 | -0.458728106972012 | 0.00418117356721961 | 0.0596910936534627 |
| TIMP1 | -0.45803269953503 | 0.00918956885258343 | 0.0885240527315416 |
| PDPN | -0.45783297969687 | 8,37E+14 | 0.0239757643173249 |
| MTHFR | -0.455973876321286 | 0.00251713036797122 | 0.0471848559743794 |
| REXO1 | -0.454514091268413 | 0.00401757464737568 | 0.0588521882250363 |
| EVI1 | -0.454500815677538 | 0.00169121030830514 | 0.0386171286585011 |
| GJC2 | -0.454453410890789 | 0.00252518970979791 | 0.0472826860622002 |
| KHSRP | -0.454322500383011 | 0.000103909754075243 | 0.0239757643173249 |
| C1QTNF5 | -0.454189125438701 | 0.00594544908994698 | 0.0698432925555804 |
| KIAA0408 | -0.453702456052909 | 0.010294316436936 | 0.0937926608698616 |
| ANPEP | -0.453537494751882 | 0.00177904074628754 | 0.0396969333280193 |
| CCNT1 | -0.453317325135238 | 0.000118569967298365 | 0.0239757643173249 |
| ATOH8 | -0.452301589339135 | 0.000823143746524039 | 0.0296581186247601 |
| TMEM205 | -0.450680332308253 | 0.00392386234442545 | 0.0583705206303003 |
| SLIT2 | -0.450637192152947 | 0.000879381343337881 | 0.0304962121691716 |
| FAM65A | -0.450585404126444 | 0.00016708188186825 | 0.0239757643173249 |
| CCDC92 | -0.449994762488616 | 0.001423831972284 | 0.0362402247869106 |
| DACT2 | -0.449444153465611 | 0.00060137783735694 | 0.0276956297030149 |
| FLJ42957 | -0.446907210300875 | 0.00966589238393535 | 0.0909325424619606 |
| RRAGD | -0.445734219197624 | 0.00109029841886448 | 0.0324969469383994 |
| KLHL28 | -0.444320035520578 | 0.000405008985215729 | 0.0256150411917438 |
| DIP2C | -0.444147239500701 | 0.00389813717185355 | 0.0581070091478916 |
| RRAGC | -0.444003886641705 | 0.00124473971560788 | 0.0340931970336374 |
| SPIN1 | -0.443977060324023 | 0.00460620516043657 | 0.0616106045420321 |
| PHF15 | -0.44386527023105 | 0.000282910168714542 | 0.0247792613550641 |
| RRBP1 | -0.441199638972487 | 2,50E+14 | 0.0239757643173249 |
| SPATA20 | -0.44087710933689 | 0.00356149195637097 | 0.0553730541650761 |
| CAPNS1 | -0.440843661467895 | 0.000183555113665136 | 0.0239757643173249 |
| ARHGEF2 | -0.440275021104991 | 0.000310926277684316 | 0.0247792613550641 |
| STK32B | -0.439919807248509 | 0.000504293840821259 | 0.0263977209883983 |
| RAMP2 | -0.43917249322305 | 0.000265121346030848 | 0.0242484061869753 |
| ZNF503 | -0.438466677123506 | 0.000471312246695408 | 0.025891456847287 |
| BDP1 | -0.437079721038364 | 0.0002470252703626 | 0.0239757643173249 |
| GSK3B | -0.436133417561018 | 0.00436536230956039 | 0.0608597912300807 |
| ZNF385D | -0.432916971500588 | 0.000374567028034285 | 0.024952349438868 |
| ATG10 | -0.431423724723597 | 0.00106711108624977 | 0.0324144728863388 |
| PAPD5 | -0.430330796346532 | 0.000572818517760288 | 0.0276956297030149 |
| CARHSP1 | -0.428146866736234 | 0.00184812190972604 | 0.0404787333017101 |
| MAP1A | -0.42713355895759 | 0.00544442317981357 | 0.0667362800082302 |
| GFOD1 | -0.425279706518471 | 0.000610284728426021 | 0.0278046472365304 |
| GLIPR2 | -0.424684855379814 | 0.00259950107546512 | 0.0477174940925986 |
| TMEM156 | -0.424244545427065 | 0.00299815893115335 | 0.051833729843994 |
| MOBKL2A | -0.423205160048621 | 0.000151632342986629 | 0.0239757643173249 |
| C9orf106 | -0.421235043322822 | 4,13E+14 | 0.0239757643173249 |
| RABGAP1 | -0.421098607820692 | 0.00491259034213978 | 0.0632933272718721 |
| TRAPPC9 | -0.415693751593633 | 0.00141614461460788 | 0.0362130573107614 |
| KIAA0427 | -0.41551872303586 | 0.00139161839617313 | 0.0361386580697316 |
| PACS1 | -0.414604469970831 | 0.000190906653341784 | 0.0239757643173249 |
| TLN1 | -0.414362220397245 | 0.00758582393084139 | 0.0796679023045967 |
| ZNF213 | -0.414006344291311 | 0.000625407893428275 | 0.0278046472365304 |
| DLC1 | -0.413784667040233 | 0.000232710188314766 | 0.0239757643173249 |
| POLR3A | -0.41251065380043 | 0.000873971811125433 | 0.0304962121691716 |
| TOB1 | -0.41175202811736 | 0.0114379115097712 | 0.0990096073799539 |
| ZC3H3 | -0.409382302137006 | 0.00207889338672869 | 0.0424475053654717 |
| NDUFS7 | -0.408091068402157 | 0.00450897342280271 | 0.0613513815919643 |
| PPP1R13B | -0.407676082723626 | 0.0022276735365267 | 0.0437950007361145 |
| ENTPD4 | -0.405285032384356 | 0.000994522594335108 | 0.0316242619277637 |
| MYOM1 | -0.405162241329146 | 0.00453148037366879 | 0.0613759335232633 |
| AXUD1 | -0.404849728559687 | 0.00163429661174432 | 0.0383625158602805 |
| BIN1 | -0.404782962399904 | 0.00374550738651992 | 0.057199739409184 |
| PKD1 | -0.404546166846868 | 0.00499259012837222 | 0.0636832607485701 |
| SNTA1 | -0.403835053302032 | 0.000149604011850278 | 0.0239757643173249 |
| SPC24 | -0.40341762203284 | 0.00574507677686419 | 0.0685537978693057 |
| PIP5K1C | -0.402855398130074 | 0.000595038898125212 | 0.0276956297030149 |
| CCDC149 | -0.402832147994426 | 0.00185549055242873 | 0.0404803351713351 |
| LLGL1 | -0.401964940374315 | 0.00406671564209751 | 0.0592312575102934 |
| GLI3 | -0.401892580315199 | 0.00544339823189671 | 0.0667362800082302 |
| CHD8 | -0.398536219026903 | 0.0036235158886957 | 0.0561611224238628 |
| MRPL2 | -0.396113489464975 | 0.00551984922864769 | 0.0671428233574088 |
| RAB40C | -0.395097932629575 | 0.00223162854582779 | 0.0437950007361145 |
| PPM1F | -0.395089985897297 | 0.00137713190117316 | 0.0360397922668013 |
| EDC4 | -0.393628825209855 | 0.00434160893298949 | 0.0607314473097 |
| VARS2 | -0.393580187353682 | 0.00583946167394044 | 0.0691349068452437 |
| LAMC3 | -0.393492805006129 | 0.0101459901291395 | 0.0930524251733646 |
| CCDC102A | -0.392839442705923 | 3,00E+14 | 0.0239757643173249 |
| ZYX | -0.391660049948766 | 0.00115291054020035 | 0.0333327826408104 |
| BMP2 | -0.391531296568528 | 0.00800383129199637 | 0.0819887850378902 |
| IRF1 | -0.39147039432364 | 0.00850429544372664 | 0.0844214755402375 |
| CCND3 | -0.391248529408551 | 0.000440534985658037 | 0.0256403684309919 |
| C6orf15 | -0.391007197552855 | 0.00220509913950362 | 0.0435939195679065 |
| CHST3 | -0.390447808886098 | 0.00623573282088986 | 0.0716298955379469 |
| GATS | -0.39041901114564 | 0.0111980554576846 | 0.0979006466116691 |
| FICD | -0.388745679440228 | 0.00107253702934513 | 0.0324608207099618 |
| ACTR1A | -0.388538907381289 | 0.000357005287234908 | 0.0248077604528682 |
| SPAG7 | -0.38753467217758 | 0.0106300575121841 | 0.095364576630716 |
| R3HCC1 | -0.386828160153661 | 0.00105795265681259 | 0.0324144728863388 |
| GFRA2 | -0.386221857417076 | 0.00116712224759724 | 0.0334387554793522 |
| BANP | -0.385261427090241 | 0.00199012015070855 | 0.0418397471995718 |
| HLA-DMA | -0.384575336977184 | 0.0113160350062294 | 0.0985697115194631 |
| MTF1 | -0.384245663718364 | 0.000926722690842771 | 0.0309763572525477 |
| CHST12 | -0.383654527331037 | 0.000569920321685484 | 0.0276956297030149 |
| PSCD4 | -0.383598629750053 | 0.00895317047632651 | 0.0869512693984428 |
| TSSC4 | -0.383539126128139 | 0.00378195036476838 | 0.0573382465694033 |
| C22orf32 | -0.383265115798069 | 0.000212240253536527 | 0.0239757643173249 |
| SAMD4B | -0.383100940672168 | 0.000436797855556044 | 0.0256150411917438 |
| RIMS3 | -0.38285728373074 | 0.00448285133180784 | 0.0611651994010436 |
| BCAS4 | -0.382477746304923 | 0.000520747065046348 | 0.0267541840887701 |
| ZDHHC1 | -0.381680926300022 | 0.00166668975429914 | 0.0385993730251762 |
| C20orf20 | -0.381377091265761 | 0.0102227487118956 | 0.0935610652120838 |
| C7orf50 | -0.378302370043889 | 0.00306496800967291 | 0.0521465049409614 |
| TNFAIP8L1 | -0.376909911720037 | 0.00105338042901761 | 0.0324144728863388 |
| LUZP1 | -0.376717705206887 | 0.0014235049265072 | 0.0362402247869106 |
| HIPK2 | -0.37644727268776 | 0.00173709481181653 | 0.0392876090183397 |
| VPS18 | -0.376213820639829 | 9,64E+14 | 0.0239757643173249 |
| ATRIP | -0.376132758507417 | 0.000622016479774719 | 0.0278046472365304 |
| SCAMP2 | -0.37565679531586 | 0.000254482291238497 | 0.0239757643173249 |
| SNX8 | -0.375411364072824 | 0.00037581124950461 | 0.024952349438868 |
| ARL2 | -0.375201069505346 | 0.00152256535807108 | 0.0372715043388988 |
| ZNF264 | -0.374028681973585 | 0.00775110568416605 | 0.0805036596592242 |
| SLC6A8 | -0.373839587053572 | 0.00624511356257654 | 0.0716298955379469 |
| ZNF467 | -0.373087073417179 | 0.00236762262336679 | 0.0453526423343655 |
| NUDC | -0.372056125963914 | 0.00218225058927456 | 0.0433541600152024 |
| SPNS1 | -0.372029945927646 | 0.00798057300243073 | 0.0819887850378902 |
| TRMT61A | -0.370570883326192 | 0.00499856545707774 | 0.0637106589575162 |
| POU2F1 | -0.370489848323822 | 0.000111546264486609 | 0.0239757643173249 |
| PMPCA | -0.370135802579189 | 0.00595910233535408 | 0.0698539440977121 |
| ZSWIM1 | -0.370084434777992 | 0.000173745439588482 | 0.0239757643173249 |
| TAOK2 | -0.36929720724689 | 0.000289315276916212 | 0.0247792613550641 |
| SH3BP1 | -0.369295042898749 | 0.00301867491058906 | 0.0519099819851916 |
| GSTO2 | -0.367644649086831 | 0.00468102050864134 | 0.0619890750889767 |
| LMNA | -0.367594931232658 | 0.00179718205757794 | 0.0398878567072566 |
| RASAL3 | -0.366282290651169 | 0.00461374107414597 | 0.0616106045420321 |
| TFB1M | -0.366120287755051 | 0.00106258153613656 | 0.0324144728863388 |
| TRIM38 | -0.365864363105947 | 0.00184782660136324 | 0.0404787333017101 |
| ZBTB47 | -0.365633660088998 | 0.00400761034936324 | 0.0588217851090614 |
| SLC4A5 | -0.363687266107541 | 0.00755925826517735 | 0.0795394520114679 |
| HECTD3 | -0.363476474873702 | 0.00141724576635658 | 0.0362130573107614 |
| RNASEH1 | -0.362802119039052 | 0.00118154604201239 | 0.0335326583455989 |
| IMP4 | -0.362789091823752 | 0.005975294967234 | 0.0698539440977121 |
| FES | -0.362632308001349 | 0.0020975860223924 | 0.0425809962545658 |
| PAK2 | -0.362622862344075 | 0.00100915771127778 | 0.0319361963154561 |
| EPHX1 | -0.362278294614724 | 0.00338116019037139 | 0.0543270198155619 |
| PRO0628 | -0.361765528203663 | 0.00581144096547358 | 0.069048712570502 |
| NOL9 | -0.361716681718087 | 0.001041811582039 | 0.0324144728863388 |
| VPS37B | -0.361101286543307 | 0.00455421842880208 | 0.0614339707988974 |
| PTRF | -0.361047545034691 | 0.0100051314926259 | 0.0920354298588498 |
| SUPT5H | -0.360965134860411 | 0.000326995723051314 | 0.0247792613550641 |
| LAT1-3TM | -0.360387484049717 | 0.0035026181119524 | 0.0551872990775131 |
| KCNRG | -0.360327381817796 | 0.000242147570213107 | 0.0239757643173249 |
| HARS | -0.3602488837482 | 0.00760557768917499 | 0.0798214325967535 |
| RBBP4 | -0.359546955682842 | 0.00487125651663495 | 0.0630536049579357 |
| RIN1 | -0.359531327123724 | 0.0102452784473257 | 0.0935610652120838 |
| MXD4 | -0.359391868743656 | 0.00971732310381683 | 0.0909443189567861 |
| HAVCR2 | -0.359161370706907 | 0.00670216903700038 | 0.0746175179343524 |
| ADAM15 | -0.35827334329976 | 0.00538418723873861 | 0.0663607090902304 |
| POLR1B | -0.358088332840331 | 0.000932930220247198 | 0.0310591128924697 |
| OSBPL5 | -0.357321247089082 | 0.0062040904771299 | 0.0715188989489642 |
| WNT10A | -0.357076631114314 | 0.0028404078827607 | 0.0503529601878963 |
| TMEM158 | -0.356669123665538 | 0.00337377885121923 | 0.0543132715255274 |
| PLEKHF1 | -0.356139015316317 | 0.0114234176016157 | 0.0990074683178536 |
| ZNF212 | -0.355322658908793 | 0.00114698565152087 | 0.0333327826408104 |
| PLOD3 | -0.355180029166697 | 0.00315238144958581 | 0.0526692992607438 |
| SMCR7L | -0.353404397096767 | 0.000504049524424129 | 0.0263977209883983 |
| IER5L | -0.353093586894007 | 7,34E+14 | 0.0239757643173249 |
| WDR68 | -0.352481713029871 | 0.00104704300515521 | 0.0324144728863388 |
| SLC9A1 | -0.351975853192827 | 0.00746165666624372 | 0.0790119191261405 |
| INTS1 | -0.351803111542447 | 0.00633451275242052 | 0.0722593335060296 |
| GRINA | -0.351697155305719 | 0.00508810278404298 | 0.0644570463798931 |
| CLCN7 | -0.350632302337468 | 0.00424401651869614 | 0.0602780707936996 |
| DFFA | -0.350255703762397 | 0.000918971316664365 | 0.0309034273478687 |
| XAB2 | -0.34987781373988 | 0.00430780642384051 | 0.0606151696798386 |
| WARS | -0.349027712614727 | 0.000125354860743692 | 0.0239757643173249 |
| ZC3H5 | -0.348379522500549 | 0.00165159374463662 | 0.0384994955549433 |
| HOXC8 | -0.347540852403681 | 0.00485482393640219 | 0.0629814901556248 |
| IRX3 | -0.346929878516522 | 0.00177372168462617 | 0.0396339918722651 |
| ACPT | -0.346620052649299 | 0.000802591717829962 | 0.029106626873633 |
| DKFZp686O24166 | -0.346553537365509 | 0.00365725680538119 | 0.0565261808564301 |
| FBXO31 | -0.345580565183905 | 0.00096869616332626 | 0.031419702037191 |
| SAPS1 | -0.345574625350382 | 0.000387041356600289 | 0.0253649229211355 |
| ISG20L2 | -0.34522539388776 | 0.000617401569169282 | 0.0278046472365304 |
| TMEM222 | -0.344300965014825 | 0.000287894517945229 | 0.0247792613550641 |
| HGS | -0.343981679677669 | 0.0102558268905096 | 0.0935610652120838 |
| TLR5 | -0.342270522176084 | 0.00165998680101044 | 0.038592374706173 |
| ATXN2 | -0.341207576209378 | 0.00248347870166438 | 0.0467646905745535 |
| ZNF787 | -0.34119556108353 | 0.00428598927959584 | 0.0605127884208247 |
| KIAA1602 | -0.340582991471989 | 0.00430696275684606 | 0.0606151696798386 |
| GTPBP6 | -0.340197016113515 | 0.0105039383698755 | 0.0950074239595838 |
| COG1 | -0.340098601563767 | 0.000133868339218992 | 0.0239757643173249 |
| CLN3 | -0.339073182756304 | 0.000368170835540854 | 0.024952349438868 |
| TBC1D13 | -0.338244259094426 | 0.000629074672909076 | 0.0278046472365304 |
| RABAC1 | -0.338029762289121 | 0.010359086106482 | 0.0941765960286727 |
| SUV39H1 | -0.337982125555774 | 0.00206835614387745 | 0.0423491468277786 |
| CBX3 | -0.337014410208819 | 0.00597281854047035 | 0.0698539440977121 |
| HADHA | -0.336536751249161 | 0.0015490782086199 | 0.0373709505227347 |
| CLPTM1 | -0.336480827332531 | 0.0107204167787853 | 0.0956334714360448 |
| SIPA1 | -0.336350399021265 | 0.000252248016639915 | 0.0239757643173249 |
| FAM116B | -0.335809521411443 | 0.000347782047374224 | 0.0248077604528682 |
| PRRX2 | -0.335748689949895 | 0.00382518562720074 | 0.0574699709016684 |
| KCNK6 | -0.33573560711504 | 0.00306050314186484 | 0.0521465049409614 |
| C17orf75 | -0.335715545854615 | 0.0022681734746626 | 0.0441590826423786 |
| NDST2 | -0.335637621386091 | 0.00812147541780479 | 0.0827341791994925 |
| PLA2G2A | -0.335218338896219 | 0.0108068165290141 | 0.0960580521779395 |
| UGCGL1 | -0.33495424782835 | 0.000420381046122342 | 0.0256150411917438 |
| DACT3 | -0.334485389085714 | 0.00154512945925134 | 0.0373382380162451 |
| PTOV1 | -0.334008288127497 | 0.00217132500780587 | 0.0433541600152024 |
| ENO2 | -0.333253107553749 | 0.00472078923604828 | 0.0622596314598132 |
| GPI | -0.333141917210818 | 0.00972059118845147 | 0.0909443189567861 |
| MFSD10 | -0.332926985643739 | 0.00502409165340558 | 0.0638892510791362 |
| TRNP1 | -0.332827301580988 | 0.0061296739294457 | 0.0708573279371896 |
| PFKP | -0.332126526206084 | 0.00947699660797334 | 0.0900937096152623 |
| ABHD14B | -0.332024669877968 | 0.00140607525487251 | 0.0362130573107614 |
| NFYB | -0.330953026385489 | 0.00244679186436746 | 0.0462307575190247 |
| KLHL22 | -0.330712842172975 | 0.00048862429386345 | 0.0263094915391259 |
| ZBTB43 | -0.330505052851679 | 0.010903212046562 | 0.0965398232590806 |
| NPLOC4 | -0.329850425152965 | 0.00674162047449833 | 0.0748606499828485 |
| RDBP | -0.329409321447504 | 0.00389527280760867 | 0.0581070091478916 |
| TBL1X | -0.329392229041222 | 0.00282442297421519 | 0.0501831199600755 |
| ZNF282 | -0.329122191627304 | 0.00152517094254267 | 0.0372804633033265 |
| EPN1 | -0.328939315194879 | 0.003289057287701 | 0.0536235529981105 |
| FLJ45256 | -0.328653733106864 | 0.00412053647477918 | 0.0594849012931304 |
| CCDC49 | -0.327921914108153 | 0.000660482289170048 | 0.0278046472365304 |
| RPUSD1 | -0.327892181125391 | 0.000937026521661013 | 0.0311332205181022 |
| GPC6 | -0.32786968124423 | 0.000656689586911771 | 0.0278046472365304 |
| GAS2L3 | -0.327187760260365 | 0.00311508755963415 | 0.0524553107461149 |
| PTP4A2 | -0.327187380895628 | 0.00304825625725335 | 0.0521465049409614 |
| MRTO4 | -0.327094410453375 | 0.0108141769663258 | 0.0960580521779395 |
| ALKBH8 | -0.326648689979658 | 0.00140740129772357 | 0.0362130573107614 |
| GAA | -0.326336238394419 | 0.00193997189082573 | 0.0415477529772433 |
| ATP6AP1 | -0.325517390359375 | 0.00141360952043013 | 0.0362130573107614 |
| ZNF669 | -0.325347886752571 | 0.00452909223165763 | 0.0613759335232633 |
| EFHD2 | -0.325004487725817 | 0.00201798028496913 | 0.0418844137451324 |
| PKM2 | -0.324786239181166 | 0.000815408426774481 | 0.0295071492871478 |
| CARD9 | -0.324289282736602 | 0.0070107082255156 | 0.0765749666154414 |
| FAM113B | -0.32397448460278 | 0.000446867645270243 | 0.0256705006083636 |
| ECM1 | -0.323740682902387 | 0.00343125830710901 | 0.0545527466858994 |
| KRT9 | -0.323690891583647 | 0.00683462358961837 | 0.0752140989717031 |
| ZNF672 | -0.323207127770191 | 0.00399352107457995 | 0.058795379716606 |
| ANKRD16 | -0.322446285132931 | 0.000857757984600602 | 0.030150983147705 |
| GPBAR1 | -0.322347555938693 | 0.000371670951485356 | 0.024952349438868 |
| ICA1 | -0.322193558849264 | 0.0113910278844707 | 0.0990074683178536 |
| CAPN5 | -0.321751500113558 | 0.00463742618534583 | 0.0617388604775303 |
| C22orf25 | -0.321539769547817 | 0.000423127595309941 | 0.0256150411917438 |
| TRIM8 | -0.321126752992616 | 0.00117515997942446 | 0.0334960839340747 |
| BRMS1 | -0.320014058261734 | 0.00525828017343666 | 0.0656141917294053 |
| ABLIM2 | -0.318912080174135 | 0.00490061985735815 | 0.0632933272718721 |
| TTLL12 | -0.318502046655163 | 0.00716949425870115 | 0.0772955591829716 |
| NR2C2 | -0.318332070889093 | 0.00769220722338358 | 0.0802786717494941 |
| C1orf85 | -0.31797082845669 | 0.00122732210924057 | 0.0338245096530109 |
| LIMS2 | -0.317821040122909 | 0.00852196353225382 | 0.0844384553320816 |
| SNAPC2 | -0.317409073484645 | 0.00727339517826627 | 0.0780612096308319 |
| GNL1 | -0.316729045520071 | 0.00266147891518667 | 0.0484715295647672 |
| RHBDD2 | -0.316368069516521 | 0.0044545105253596 | 0.061098881984569 |
| PHLDA3 | -0.316331030435554 | 0.0034016662109876 | 0.0544462843731727 |
| ZNF668 | -0.316237413888816 | 0.00140260242518337 | 0.0362130573107614 |
| LONP2 | -0.31553937254137 | 0.00603171919808295 | 0.0702871641237173 |
| ITGA5 | -0.315434977147231 | 0.00642956560881553 | 0.0727815399630157 |
| ZNF767 | -0.313638723455237 | 0.0114928822851757 | 0.0990772169654038 |
| GIT1 | -0.313508180128758 | 0.00145210286283465 | 0.0365131484210659 |
| OBFC2B | -0.313369542251533 | 5,61E+14 | 0.0239757643173249 |
| ANXA11 | -0.313317204356058 | 0.00256897362742756 | 0.0475674471659167 |
| CDC42EP4 | -0.31306135449424 | 0.00527162583392084 | 0.0656273476573112 |
| PDE2A | -0.312564593249125 | 0.00547603601923913 | 0.0670743896808348 |
| PPM1M | -0.312420117247038 | 0.00476170292185143 | 0.0624120526276684 |
| NKG7 | -0.312323036183301 | 0.00816126657123999 | 0.0827442748264649 |
| PCIF1 | -0.311995161612211 | 0.00419069072500488 | 0.0597756964939427 |
| PRKAB1 | -0.311948452148754 | 0.00821834240708905 | 0.0827601498538441 |
| C17orf63 | -0.311492718705493 | 0.000360549094091167 | 0.0249002611973077 |
| ATG7 | -0.311414596512455 | 0.000925663367378738 | 0.0309763572525477 |
| PCNXL3 | -0.311344101037162 | 0.0034943584641304 | 0.0551872990775131 |
| LINGO4 | -0.311184986095635 | 0.000437022209447028 | 0.0256150411917438 |
| TWF2 | -0.311017737612836 | 0.00331866563125513 | 0.0538101935477045 |
| C14orf153 | -0.310515070768805 | 0.00842196354646042 | 0.0840479647448322 |
| LYN | -0.310208409369445 | 0.00989807572648683 | 0.0916370236613458 |
| RPAP2 | -0.310000049944787 | 0.00529766879654307 | 0.0656273476573112 |
| GALK1 | -0.309875634631672 | 0.00228797518956432 | 0.0443714994259888 |
| YIPF6 | -0.309687718859261 | 0.00569484221194128 | 0.068198808244586 |
| G6PC3 | -0.30945169952161 | 0.0015224457971591 | 0.0372715043388988 |
| CD320 | -0.309403900102985 | 0.00528979695989138 | 0.0656273476573112 |
| MRPS18A | -0.309317715564606 | 0.00423007693861475 | 0.0601313925876867 |
| C20orf94 | -0.30915662094912 | 0.0095327275249901 | 0.0903254705065237 |
| INF2 | -0.308971469838741 | 0.000325217724559333 | 0.0247792613550641 |
| KRTCAP2 | -0.308203809317353 | 0.00309723775272679 | 0.0523765739733842 |
| GP1BA | -0.308176171604708 | 0.000157314912660091 | 0.0239757643173249 |
| GDPD5 | -0.308130660704743 | 0.00771630903728279 | 0.08037591346919 |
| DBN1 | -0.307838766038172 | 0.000762075028952378 | 0.0290452776012764 |
| PRPH | -0.307335693811468 | 0.00873324332649559 | 0.0858674355657682 |
| STXBP2 | -0.305854732310274 | 0.00592947662432153 | 0.0698398436072017 |
| NTHL1 | -0.305593450028788 | 0.003156778143092 | 0.0526692992607438 |
| RNF220 | -0.305523646325336 | 0.000652455213446867 | 0.0278046472365304 |
| NRM | -0.305109537097913 | 0.0026501835123501 | 0.0483716609063375 |
| SRPRB | -0.30508290192265 | 0.00999549058842198 | 0.0920270665568984 |
| GPN2 | -0.304779279725874 | 0.000695506719629955 | 0.0280690958739751 |
| ICAM3 | -0.303787764991133 | 0.00497195107408569 | 0.0636109406478712 |
| BIN3 | -0.303769424201315 | 0.00556823343304693 | 0.0674806458925102 |
| ODZ3 | -0.303593011842343 | 0.0045706873199855 | 0.0614598093641915 |
| IL17RA | -0.303589808202914 | 0.000417756996633224 | 0.0256150411917438 |
| C20orf27 | -0.303495298431253 | 0.0047579074461506 | 0.0624120526276684 |
| MNT | -0.303363354675165 | 0.00481648855458748 | 0.062833282507573 |
| FSCN1 | -0.303001649214343 | 0.0115868865970353 | 0.0991286993899792 |
| BMP8B | -0.302528138051962 | 0.00925904395677208 | 0.088935975594015 |
| STAT5A | -0.302141070727825 | 0.000229632267183278 | 0.0239757643173249 |
| METT10D | -0.301301780902346 | 0.00341966373372013 | 0.0545527466858994 |
| FBXO27 | -0.301287377408803 | 7,30E+14 | 0.0239757643173249 |
| SEPN1 | -0.300945287842543 | 0.000240127321844857 | 0.0239757643173249 |
| KLK1 | -0.300431735429593 | 0.00776676666216155 | 0.0805517743665677 |
| ZDHHC24 | -0.300317669051016 | 0.000557504682065782 | 0.0275377535242345 |
| HNRNPUL2 | -0.29933636654337 | 0.00659038775742188 | 0.0739701010686295 |
| ATL3 | -0.299150145090301 | 0.00481313504254623 | 0.062833282507573 |
| SPOP | -0.298817633412649 | 6,30E+13 | 0.0239757643173249 |
| MRPL37 | -0.298800707294326 | 0.00445509899394521 | 0.061098881984569 |
| TPRG1L | -0.298734078746001 | 0.000646677110684955 | 0.0278046472365304 |
| SARM1 | -0.298160358099405 | 0.000398225558469142 | 0.0255940642713411 |
| NICN1 | -0.297464265952023 | 0.00165023386354983 | 0.0384994955549433 |
| IRF2BP1 | -0.297189522966582 | 0.00225962085437311 | 0.0440954381510228 |
| FOXRED2 | -0.297084259666208 | 0.000332300633300548 | 0.0247792613550641 |
| SYT11 | -0.296771483766078 | 0.00113425198032964 | 0.0332407719446606 |
| PODXL2 | -0.296526014281978 | 0.00851179064868732 | 0.0844381806543797 |
| PLEKHB2 | -0.296051893865513 | 0.000887497038189929 | 0.0306272188766164 |
| ST6GALNAC6 | -0.295810598522196 | 0.00582892718904701 | 0.0691151993141894 |
| BCL3 | -0.295296919884527 | 0.00535359133896864 | 0.0661140889449935 |
| C18orf45 | -0.294975436716648 | 0.00464389367564536 | 0.0617388604775303 |
| C10orf54 | -0.294326549662202 | 0.00064411462062182 | 0.0278046472365304 |
| CD2BP2 | -0.294243630100668 | 0.00356338420409146 | 0.0553730541650761 |
| WDR22 | -0.294044346564127 | 0.000471500392020513 | 0.025891456847287 |
| FBXW9 | -0.293816330061876 | 0.00363000731722517 | 0.0561864963251574 |
| ARHGDIA | -0.293244522713449 | 0.0052795078723833 | 0.0656273476573112 |
| MFN2 | -0.293206076112456 | 0.00704345250392382 | 0.0767814737264675 |
| NEK6 | -0.292985297415388 | 0.000184867596063758 | 0.0239757643173249 |
| SSU72 | -0.2924488150461 | 0.000587396597091153 | 0.0276956297030149 |
| XPNPEP2 | -0.292448356438145 | 0.00693471138340785 | 0.0760442725218756 |
| TSPAN33 | -0.292227341721463 | 0.000642422781075927 | 0.0278046472365304 |
| CNNM3 | -0.29216006001178 | 0.000116978326245552 | 0.0239757643173249 |
| DDR2 | -0.292052637891067 | 0.00118767012919351 | 0.0335326583455989 |
| B3GALT6 | -0.291795288882217 | 0.00160007268024962 | 0.038049728336336 |
| DNM1 | -0.291547937948012 | 0.00860861382701175 | 0.0849430858117591 |
| ZNF581 | -0.291284587859622 | 0.00119127548559699 | 0.0335326583455989 |
| MED15 | -0.291052093071198 | 5,54E+14 | 0.0239757643173249 |
| SLCO4A1 | -0.290647227867829 | 0.000512427489685305 | 0.0265727974869209 |
| HIC2 | -0.290494526295786 | 0.000982301320397452 | 0.0314972979171622 |
| CENTA1 | -0.290382331651564 | 0.000670168564271685 | 0.0279546184311492 |
| UBXN2A | -0.290133978855821 | 0.00526383638331379 | 0.0656273476573112 |
| CCDC101 | -0.290091300498875 | 0.000861961978644771 | 0.0302067770453071 |
| PPP1R16B | -0.289856035155296 | 0.000413813620428378 | 0.0256150411917438 |
| QPRT | -0.289535233661336 | 0.0112816404596002 | 0.0984193520591214 |
| C19orf53 | -0.289481109873149 | 0.00376132175541785 | 0.057231226636824 |
| SLC24A6 | -0.288848319005718 | 0.00353402252959842 | 0.0553508848719995 |
| FSTL3 | -0.288720600115831 | 0.00571318029999214 | 0.0683201144207394 |
| FLJ20021 | -0.288651065029605 | 0.00714302936620009 | 0.0772598225014728 |
| C19orf24 | -0.288584442602844 | 0.00104956200350998 | 0.0324144728863388 |
| C9orf90 | -0.288340395634569 | 0.000274547536671077 | 0.0244391352696618 |
| FLYWCH1 | -0.288117179281215 | 0.00896533170421899 | 0.0870186073168683 |
| C17orf62 | -0.287630838527705 | 0.00970377257320844 | 0.0909443189567861 |
| COL8A1 | -0.286690982733458 | 0.00083005793198071 | 0.0297696230455384 |
| JMJD8 | -0.286468931211788 | 0.00468975566816057 | 0.0620553838252789 |
| MAD2L2 | -0.286258489470536 | 0.00657312179828407 | 0.0739140228795615 |
| TESK1 | -0.285882663166625 | 0.00770372179300607 | 0.0803484667709142 |
| ZER1 | -0.284767657526014 | 0.00249004612206302 | 0.046782514388105 |
| CTSA | -0.284499643504834 | 0.00311631575679517 | 0.0524553107461149 |
| FXYD5 | -0.283863422452818 | 0.00303764195547221 | 0.052021181060484 |
| C2 | -0.283692443766964 | 0.0010707580643269 | 0.0324608207099618 |
| SETD1A | -0.283393821677306 | 0.00151042488339242 | 0.0372481964577041 |
| TOP3A | -0.283254835025116 | 0.000171893198948709 | 0.0239757643173249 |
| SPSB1 | -0.28239477020641 | 0.0102412906122724 | 0.0935610652120838 |
| WNT11 | -0.282265687103311 | 0.00424764457511951 | 0.060278168454765 |
| UBQLNL | -0.281808874851437 | 0.0011731896654633 | 0.0334960839340747 |
| LFNG | -0.280638937014519 | 0.00236429614590264 | 0.0453410986690039 |
| CAMK1 | -0.280306842238434 | 0.00274087229445656 | 0.0494307261251613 |
| CCDC124 | -0.279702324049051 | 0.00458697378274417 | 0.0615268054694274 |
| OGFR | -0.279157122470004 | 0.0105476518493715 | 0.0950943212573529 |
| MYO1C | -0.279119966723515 | 0.000973272203561617 | 0.031419702037191 |
| KLHDC4 | -0.278654852444299 | 0.0042953192979264 | 0.0605418162855909 |
| SFMBT2 | -0.278473276108162 | 0.00219719128910031 | 0.0435410073790045 |
| UBE2O | -0.278403968014986 | 0.0116871651757999 | 0.0996978781352102 |
| RAVER1 | -0.278301321335364 | 0.00140583162022202 | 0.0362130573107614 |
| FXC1 | -0.277970323330508 | 0.000623584356372541 | 0.0278046472365304 |
| NAV2 | -0.277873291410565 | 0.00043149577764142 | 0.0256150411917438 |
| AP2B1 | -0.277742498070608 | 0.011544605486402 | 0.0991043202782726 |
| NUDT18 | -0.277106063229569 | 0.0109552101383749 | 0.0967129608577777 |
| PNKP | -0.276519774628567 | 0.0110322572593635 | 0.097011597643616 |
| SEMA6A | -0.276393777378335 | 0.00989439108400693 | 0.0916370236613458 |
| UBL4A | -0.276271152810959 | 0.0066356298047575 | 0.074331557018838 |
| LRRC28 | -0.276238428544564 | 0.0114212371763225 | 0.0990074683178536 |
| MSRB2 | -0.275832180217301 | 0.00957963446042731 | 0.0905522971199733 |
| KIAA0195 | -0.275391014513511 | 0.010271258253678 | 0.0936338252413606 |
| RBM42 | -0.275082437465126 | 0.00196723899731847 | 0.0416624177472815 |
| DDX51 | -0.2742212978382 | 0.00118216560710583 | 0.0335326583455989 |
| C17orf53 | -0.273434721197426 | 0.0104554381802947 | 0.0946390559810692 |
| SIL1 | -0.273333148977516 | 0.00143172451753748 | 0.0362611369418848 |
| AMDHD2 | -0.27318660049919 | 0.00997984175728728 | 0.0919847430187176 |
| SNRPD3 | -0.272359922171907 | 0.00551990566395302 | 0.0671428233574088 |
| IQSEC1 | -0.27234733957934 | 0.00279344117769909 | 0.0499995933806226 |
| LRP5 | -0.271446481947594 | 0.00582949296149958 | 0.0691151993141894 |
| CPSF3L | -0.271319579946999 | 0.0115182970877118 | 0.0990871179958914 |
| PI4KB | -0.271243392488412 | 0.00330304090472005 | 0.0537988443248239 |
| TNS1 | -0.270501488779475 | 0.00168921122412349 | 0.0386171286585011 |
| SPHK2 | -0.269730942865475 | 0.00311656267739443 | 0.0524553107461149 |
| GHDC | -0.269692577001323 | 0.00202329050562397 | 0.04191314831657 |
| KLHL35 | -0.268877711884152 | 0.00752518241852814 | 0.0793734021940377 |
| GNAI2 | -0.268767616334986 | 0.0100327851813629 | 0.0921665243537342 |
| DHX58 | -0.268363898479144 | 0.0108937420543045 | 0.0965073072038063 |
| ZNF524 | -0.26821746479463 | 0.00893593884714881 | 0.086891251737291 |
| C2orf24 | -0.267695612446178 | 0.00138379109263437 | 0.0360397922668013 |
| FLJ39827 | -0.267476602869644 | 0.00161996106501657 | 0.0381412615109841 |
| MAGEL2 | -0.267138313613713 | 0.00172935236233468 | 0.0391657135012558 |
| ADAT3 | -0.267058546338413 | 0.00440074618909707 | 0.0609441107019216 |
| WDR24 | -0.266700490277991 | 0.00109556934436953 | 0.0325253161553371 |
| TTYH3 | -0.266625649994737 | 0.000259259736700617 | 0.0239757643173249 |
| SLC17A7 | -0.266451484016718 | 7,15E+14 | 0.0239757643173249 |
| SSH1 | -0.266167895248948 | 0.00949927818218601 | 0.0902470992296014 |
| KIAA1539 | -0.265826399841443 | 0.00153933377480996 | 0.0373382380162451 |
| RPS6KA4 | -0.265596599081497 | 0.00986906872932647 | 0.0914702216416305 |
| FXYD6 | -0.265470291867259 | 0.00995587048043543 | 0.0918146371287137 |
| EMILIN1 | -0.265460281052781 | 0.00570379854734758 | 0.0682569594673959 |
| COL4A3BP | -0.265187432424558 | 0.00383407335551585 | 0.0574972838521773 |
| SUV39H2 | -0.265094432419927 | 0.000840275884784739 | 0.0297767990079848 |
| C6orf106 | -0.2648785705231 | 0.000238253237284328 | 0.0239757643173249 |
| MAP1S | -0.264225005201748 | 0.00044722126495407 | 0.0256705006083636 |
| SMPD2 | -0.263508196746781 | 0.00991522499762674 | 0.091723643850332 |
| PSMC3 | -0.262416587986435 | 0.0094641784672144 | 0.0900785910209195 |
| MOSPD3 | -0.261923745554232 | 0.00557972339706896 | 0.0675291243042718 |
| MAP3K11 | -0.261910983790028 | 0.00783955714127479 | 0.0810038908588828 |
| GTF2F1 | -0.26129787164101 | 0.00602848616051713 | 0.0702871641237173 |
| TSPAN17 | -0.261220933336144 | 0.00914270203686314 | 0.0884308065692178 |
| FAM188B | -0.260585677950568 | 0.00708358786279624 | 0.0769820180111139 |
| PLEKHO1 | -0.260289829474683 | 0.000169976192240712 | 0.0239757643173249 |
| SRM | -0.260127257437611 | 0.0104245769833037 | 0.094513893498951 |
| ECHS1 | -0.259740820132783 | 0.00765983016579228 | 0.0800427875981965 |
| ZNF564 | -0.259567739181729 | 0.00460686526437111 | 0.0616106045420321 |
| C15orf39 | -0.259324691632053 | 0.00108830286905534 | 0.0324969469383994 |
| SFRS15 | -0.258997475227878 | 0.00073453809840315 | 0.0287947341006642 |
| NLGN2 | -0.258969531727335 | 0.00564958238786661 | 0.0679010457966987 |
| FBXL15 | -0.258817831525593 | 0.00727850697094836 | 0.0780657390711382 |
| TREX2 | -0.258645167992034 | 0.00161250390344311 | 0.0381274715578325 |
| C1orf50 | -0.258590838095157 | 0.000659125184509041 | 0.0278046472365304 |
| ORMDL3 | -0.258247211915536 | 0.00248801905516525 | 0.046782514388105 |
| EXT1 | -0.258213853061572 | 0.00669864880422387 | 0.0746175179343524 |
| FAM102A | -0.258133838847249 | 0.00115797898874475 | 0.0333489935063063 |
| ZNF160 | -0.257911958339385 | 0.00400114267345838 | 0.0588217851090614 |
| ST3GAL1 | -0.257779340359575 | 0.00149075819351747 | 0.0370474414028692 |
| TMUB1 | -0.257015933325556 | 0.0078985717418361 | 0.0814619734910804 |
| NRTN | -0.256366807313173 | 0.00792269584724652 | 0.0815689242363127 |
| TBC1D16 | -0.256306188520435 | 0.0115402247611051 | 0.0991043202782726 |
| NME6 | -0.255155593086749 | 0.000553156307049042 | 0.0275377535242345 |
| MAP4K2 | -0.25504776930457 | 0.000849963617714884 | 0.0299756236874618 |
| ZNF292 | -0.254283778306075 | 0.00722497216656883 | 0.0777420081995506 |
| TTC39C | -0.254274135971884 | 0.00238767154807276 | 0.0454750349991065 |
| MLST8 | -0.254188240167376 | 0.00255970051339644 | 0.0475674471659167 |
| BSG | -0.25405756101056 | 0.00228120818789719 | 0.0443609713735241 |
| MYO1G | -0.253999205315138 | 0.002915183590965 | 0.051241970491239 |
| TMEM115 | -0.253690157360953 | 0.00591037199608317 | 0.0698254451716114 |
| BIRC3 | -0.253267436477914 | 0.00883294936759601 | 0.0864901618664725 |
| BRSK1 | -0.252973622045775 | 0.0100388093515818 | 0.0921709986025539 |
| CORO7 | -0.252908007385418 | 0.00748503486184342 | 0.0792049845401199 |
| MFHAS1 | -0.252816036078357 | 0.00464098288658848 | 0.0617388604775303 |
| B3GALT4 | -0.251618650768862 | 0.00894866092267782 | 0.0869512693984428 |
| GMEB1 | -0.25154902827258 | 0.00456434914119762 | 0.0614598093641915 |
| PLSCR3 | -0.250971080651429 | 0.0096024594158984 | 0.0905864719066928 |
| FDX1L | -0.250837523971003 | 0.00262988560273333 | 0.0481066766407682 |
| MGC3196 | -0.250292173200337 | 0.00183756561085772 | 0.0404787333017101 |
| DECR2 | -0.250238170063431 | 0.0105126539296952 | 0.0950074239595838 |
| RUNX3 | -0.250121190561821 | 0.00414316095835301 | 0.0596085197171515 |
| FLJ23754 | -0.249940742815481 | 0.00257325956729261 | 0.0475688381311017 |
| ANTXR1 | -0.249732038883849 | 0.00381031921304904 | 0.0573996141361216 |
| RIPK1 | -0.249622893211945 | 0.0100074569292634 | 0.0920354298588498 |
| VPS37D | -0.249607376549786 | 0.00254177067230975 | 0.0474694403764939 |
| AP1M1 | -0.249603969287757 | 0.00446622912447857 | 0.061098881984569 |
| NRSN2 | -0.249398016818499 | 0.00136610788177748 | 0.0360389743672517 |
| SLC16A5 | -0.24877560665352 | 0.00612784555320082 | 0.0708573279371896 |
| SLC22A18AS | -0.248281860685167 | 0.00145540105475458 | 0.0365408837970509 |
| DPRXP4 | -0.247779328631069 | 0.000799450960987766 | 0.029106626873633 |
| FERMT3 | -0.247713168147294 | 0.00280208047607186 | 0.0500881522232834 |
| ADRB2 | -0.247267622349104 | 0.00470104423278082 | 0.0621060176975155 |
| RAPGEF1 | -0.247128357363239 | 0.00527026087258344 | 0.0656273476573112 |
| AP2A2 | -0.246927877754403 | 0.00270881142793667 | 0.0489055043703187 |
| C6orf64 | -0.245997194698011 | 0.0111216403788671 | 0.0975913683429741 |
| WBSCR22 | -0.245231322151077 | 0.00498309903909792 | 0.0636109406478712 |
| ZNF234 | -0.245152720620576 | 0.00305788130568575 | 0.0521465049409614 |
| PCNX | -0.24421870281171 | 0.0065944749480864 | 0.0739701010686295 |
| TMEM127 | -0.243933557877689 | 0.00338628121073402 | 0.0543306492394007 |
| PFN1 | -0.242575605393904 | 0.00819348650611212 | 0.0827442748264649 |
| SLC22A18 | -0.242220625862568 | 0.000893738777895895 | 0.0306272188766164 |
| HOXB7 | -0.24192341359825 | 0.00667040384436272 | 0.0744705180370636 |
| C9orf64 | -0.241892353706184 | 0.00189132257663734 | 0.0410468782408152 |
| SLC30A3 | -0.241808186954268 | 0.00077141437774276 | 0.029106626873633 |
| C12orf52 | -0.241602286504941 | 0.00157267453964418 | 0.0376555014846483 |
| AKNA | -0.241594949070469 | 0.00201209194622991 | 0.0418844137451324 |
| PBX1 | -0.241446557666448 | 0.000717153283921853 | 0.028559171206132 |
| PTK6 | -0.241303208959769 | 0.00939508818160937 | 0.0896735308893747 |
| TACO1 | -0.240717210246399 | 0.00826974731916043 | 0.0829766207804367 |
| PGLS | -0.239871447523524 | 0.00739806191256696 | 0.078688906451495 |
| MRPS2 | -0.239789735817987 | 0.00465729875043846 | 0.0617388604775303 |
| DYRK1B | -0.239501351844189 | 0.00136899237519923 | 0.0360397922668013 |
| DHX8 | -0.239019779382864 | 0.00108380752082856 | 0.0324969469383994 |
| PAPSS2 | -0.238998997551736 | 0.000241405292092276 | 0.0239757643173249 |
| LASP1 | -0.238861428739 | 0.00414220670239166 | 0.0596085197171515 |
| C19orf40 | -0.238751620444275 | 0.000700541151964894 | 0.0281671691198252 |
| C14orf139 | -0.238556858521659 | 0.00398759019271165 | 0.058795379716606 |
| GIGYF1 | -0.237975382583827 | 0.000253463869436074 | 0.0239757643173249 |
| NFIC | -0.237767367845189 | 0.00600004846845877 | 0.0700889872322559 |
| GALM | -0.237677468601781 | 0.00830602149553595 | 0.0831901527164209 |
| CLIP2 | -0.237345909224515 | 0.000756259808264119 | 0.0290452776012764 |
| ABP1 | -0.236980493556088 | 0.00336459361810428 | 0.0542178367540793 |
| ACTRT1 | -0.236612216699006 | 8,63E+14 | 0.0239757643173249 |
| ZKSCAN2 | -0.236526752664918 | 0.000191640144432106 | 0.0239757643173249 |
| RNF26 | -0.236171429335796 | 0.00551023657968164 | 0.0671428233574088 |
| ADAM17 | -0.236076798916143 | 0.00843075037783233 | 0.0840852431332517 |
| FIBP | -0.235569155207728 | 0.00497906253922133 | 0.0636109406478712 |
| FOXJ2 | -0.235222933434712 | 0.00836471896942784 | 0.0835768979382327 |
| TNS4 | -0.235091096186996 | 0.00750582483443705 | 0.0792778935241365 |
| GATAD2A | -0.234817538549363 | 0.0103943353700144 | 0.0944454730181545 |
| ANKRD47 | -0.234531284007859 | 0.00593177220151661 | 0.0698398436072017 |
| CYB561D2 | -0.233996905380892 | 0.00191455049260805 | 0.0412682425059276 |
| SNX11 | -0.23397685648514 | 0.0039948080295255 | 0.058795379716606 |
| ATG2A | -0.233455406974964 | 0.000579897956453732 | 0.0276956297030149 |
| DCBLD1 | -0.233004324579763 | 0.00756839777241734 | 0.0795853122676305 |
| TSPAN18 | -0.232742076454932 | 0.0102576561343297 | 0.0935610652120838 |
| COG2 | -0.23263975143339 | 0.00941363388890271 | 0.0897990542777505 |
| TRIM21 | -0.23263291048947 | 0.000794570388264065 | 0.029106626873633 |
| RELB | -0.232097736167049 | 0.0114117306415115 | 0.0990074683178536 |
| ADRA1B | -0.232061161252604 | 0.00149472252687324 | 0.0370807022091385 |
| FLJ11235 | -0.231549442675235 | 0.00337927802926859 | 0.0543270198155619 |
| CRLF1 | -0.230393252275302 | 0.00444594540256568 | 0.061098881984569 |
| AATF | -0.230295780670924 | 0.0114279778476417 | 0.0990074683178536 |
| KIAA1958 | -0.229637074330596 | 0.00209197105750698 | 0.0425188647414667 |
| CSRNP3 | -0.229382574981571 | 0.00203326937706346 | 0.0419923102364744 |
| GMIP | -0.229229760280629 | 0.00558924164973789 | 0.0675291243042718 |
| SKI | -0.228887652280749 | 0.00803201938806099 | 0.0821259181410708 |
| C20orf43 | -0.228712029034736 | 0.00191887658894557 | 0.0412682425059276 |
| SMARCC2 | -0.228638385054868 | 0.00960626601470487 | 0.0905864719066928 |
| DRG2 | -0.22856811102818 | 0.00874793120747807 | 0.0859103615809321 |
| SIPA1L3 | -0.228348804918494 | 5,10E+13 | 0.0239757643173249 |
| FIBCD1 | -0.228285970028532 | 0.00055698157930818 | 0.0275377535242345 |
| MAP3K14 | -0.228081033834012 | 0.00603659429802181 | 0.0702871641237173 |
| FARP2 | -0.22769930525544 | 0.00324569035392131 | 0.0532642362209362 |
| FAM109A | -0.227490025917975 | 0.011324104621593 | 0.098588412934643 |
| VPS41 | -0.227476561986151 | 0.00504766861352468 | 0.0640911454925491 |
| STRN | -0.227329581412461 | 3,48E+14 | 0.0239757643173249 |
| ZNF629 | -0.226664626554095 | 0.00161976491403794 | 0.0381412615109841 |
| ITPKB | -0.225747387611427 | 0.00682460107825133 | 0.0751835271664935 |
| MMP15 | -0.225608037205797 | 0.00531316594951904 | 0.0657079943504413 |
| TXNL4A | -0.224301559403338 | 0.00640265899943827 | 0.07270031494178 |
| P4HTM | -0.22405661881772 | 0.0111398469267059 | 0.0976481790110299 |
| OTUB1 | -0.223986823623689 | 0.00731170428195826 | 0.0781507943713978 |
| HEATR6 | -0.223847201332105 | 0.00657732666531734 | 0.0739140228795615 |
| CNNM1 | -0.223436250335661 | 0.00181108484217131 | 0.0400895189930633 |
| KLHL36 | -0.223422240408547 | 0.00493809123912633 | 0.0635235446418059 |
| LRRC33 | -0.223297709617827 | 0.00523661279176132 | 0.0654911018269413 |
| VWCE | -0.223213562879443 | 0.00218152654667632 | 0.0433541600152024 |
| C14orf93 | -0.223109328961433 | 0.0114608044651886 | 0.0990453088884465 |
| PGAP3 | -0.22267076315836 | 0.00487076124428626 | 0.0630536049579357 |
| ZNF784 | -0.222612859335292 | 0.00247942006691224 | 0.0467411397891518 |
| C14orf78 | -0.222592226199754 | 0.000346377319311555 | 0.0248077604528682 |
| BCKDK | -0.221867624533623 | 0.000193873378570876 | 0.0239757643173249 |
| GRASP | -0.221733008491631 | 0.00777552054303866 | 0.0805732334978883 |
| DKFZp686K1684 | -0.221369597370313 | 0.00729079216434013 | 0.0781446620590351 |
| ARFGAP2 | -0.221112511691565 | 0.00927898932808481 | 0.0889735347668778 |
| METTL13 | -0.221043850116356 | 0.00042334890943369 | 0.0256150411917438 |
| ZNF275 | -0.220729899005048 | 0.000199683949651155 | 0.0239757643173249 |
| VPS4A | -0.220127667221929 | 0.00313562026806009 | 0.0525105985735697 |
| GCLM | -0.219938608313699 | 0.00749478690747531 | 0.0792115700710058 |
| HMGB3L1 | -0.219203965578783 | 0.0031690839319883 | 0.0527525711318772 |
| SH2D3C | -0.219008528405506 | 0.000715173460177726 | 0.0285486269019627 |
| TCIRG1 | -0.217848236584202 | 0.00210896232535129 | 0.0427355592909381 |
| DENND3 | -0.217180319607203 | 0.0114239060880545 | 0.0990074683178536 |
| STOML1 | -0.217141198433203 | 0.000647239796784867 | 0.0278046472365304 |
| NLRX1 | -0.216923217248074 | 0.00677491488770725 | 0.074983532726579 |
| EHD4 | -0.216870098566707 | 0.00597003648789905 | 0.0698539440977121 |
| ZFPL1 | -0.216029028692191 | 0.00494331671414723 | 0.0635416602499573 |
| ZNF650 | -0.215749988902295 | 0.00960732038935356 | 0.0905864719066928 |
| NRBP1 | -0.215639851257887 | 0.00868828376768026 | 0.0855264172659998 |
| TFAP4 | -0.215299188145025 | 0.00256779904281414 | 0.0475674471659167 |
| CDC42EP2 | -0.215263715034445 | 0.00856465433846816 | 0.0846096356784219 |
| TMEM103 | -0.215098476808558 | 0.00572699336446277 | 0.0683870384109377 |
| DKFZp761E198 | -0.214821695329901 | 0.00356601670461141 | 0.0553730541650761 |
| ATP5SL | -0.213264204542498 | 0.00932803886891613 | 0.089238238512631 |
| ZNF660 | -0.21299727104288 | 0.00134530882812538 | 0.0356592527913615 |
| AGBL5 | -0.211753033619433 | 0.00040880949731705 | 0.0256150411917438 |
| ALKBH4 | -0.211600660543319 | 0.00643167399288694 | 0.0727815399630157 |
| PPME1 | -0.210659482314428 | 0.000357675267853441 | 0.0248077604528682 |
| PAIP1 | -0.210473388561452 | 0.000638060581943306 | 0.0278046472365304 |
| CCNY | -0.210178033966161 | 0.00464252432232335 | 0.0617388604775303 |
| REM2 | -0.21008674344313 | 0.000472846502557686 | 0.025891456847287 |
| PPM1G | -0.209016003563356 | 0.00136612957021121 | 0.0360389743672517 |
| ITGAL | -0.208650440657484 | 0.00680317340474143 | 0.0751835271664935 |
| RNF121 | -0.208572591771165 | 0.000239180643484056 | 0.0239757643173249 |
| SYDE1 | -0.207528558964482 | 0.00565415230951695 | 0.0679069403637944 |
| ZNF574 | -0.207516876417569 | 0.0013157947715426 | 0.0353473552332152 |
| C16orf35 | -0.207301723077192 | 0.00411936947196127 | 0.0594849012931304 |
| SPN | -0.207270101486471 | 0.00120102014235203 | 0.0335326583455989 |
| C1orf163 | -0.207023319550537 | 0.00972491215565777 | 0.0909443189567861 |
| SERGEF | -0.205913655437729 | 0.00887894026628903 | 0.0866235952798865 |
| EPS15L1 | -0.205609352084779 | 0.00318054595546978 | 0.0528120498985903 |
| RNMTL1 | -0.205499857812125 | 0.00240018236650709 | 0.0456089448320513 |
| MYOZ3 | -0.205422655810268 | 0.0068083711627704 | 0.0751835271664935 |
| ZNF618 | -0.205060573969634 | 0.00265738907833778 | 0.0484500532289273 |
| RAB9B | -0.204463735857593 | 0.00259118884949911 | 0.0477015502496782 |
| KIRREL | -0.204095952203322 | 0.00246996715983762 | 0.0466157294134434 |
| CD84 | -0.203995654588691 | 0.00169107730190235 | 0.0386171286585011 |
| FLJ37078 | -0.203656443283311 | 0.0108260102312857 | 0.0960637602094817 |
| DAXX | -0.201432364608635 | 0.0105404920296733 | 0.0950943212573529 |
| ELL | -0.201233848702684 | 0.00611834012274094 | 0.0708246798909219 |
| CHML | -0.201103678478041 | 0.00481264197924474 | 0.062833282507573 |
| KIAA1026 | -0.201007059140811 | 0.000425555502599405 | 0.0256150411917438 |
| C20orf29 | -0.200827804424207 | 0.00730407166521036 | 0.0781446620590351 |
| UNC13D | -0.19984663737023 | 0.00270858309693034 | 0.0489055043703187 |
| LARP4 | -0.198929012315925 | 0.000364709728335858 | 0.024952349438868 |
| WWOX | -0.19864286166123 | 0.00283523827287231 | 0.0503149000961967 |
| XPNPEP3 | -0.198485097386444 | 0.00810691684500199 | 0.0826379288437864 |
| EFNA4 | -0.197822228781473 | 0.00603782810375021 | 0.0702871641237173 |
| ELF4 | -0.197775251187408 | 0.00321661387022371 | 0.0531188040513332 |
| VRK3 | -0.197748356895402 | 0.000977155381042919 | 0.0314617572008519 |
| ADORA2A | -0.197422162757486 | 0.00132600991001212 | 0.0354589750767884 |
| CAPS | -0.196934993340578 | 0.00916162016813622 | 0.0884525407786117 |
| ALKBH6 | -0.19612734721531 | 0.00708497246143377 | 0.0769820180111139 |
| STX5 | -0.195486184451532 | 0.00554334958579344 | 0.0673045931474234 |
| CYP11A1 | -0.195277075106906 | 0.000991426882867418 | 0.0316155017092166 |
| ATG9A | -0.195120216829745 | 0.00525641936899635 | 0.0656141917294053 |
| CDCP1 | -0.194362521302436 | 0.00133553876526777 | 0.0355133838444844 |
| CHAF1A | -0.194260454732236 | 0.00687659234149 | 0.0756061797334495 |
| FLJ20309 | -0.194218535839359 | 0.00953935943472719 | 0.0903254705065237 |
| HRB | -0.19401433992886 | 0.00256698247115085 | 0.0475674471659167 |
| FAM119B | -0.193608248527959 | 0.000335895706396484 | 0.0247792613550641 |
| ILVBL | -0.19359247254577 | 0.00549886329253758 | 0.0671428233574088 |
| COPS6 | -0.193334398179102 | 0.00525335451840732 | 0.0656141917294053 |
| FAM12A | -0.193124267709013 | 0.00729714359939754 | 0.0781446620590351 |
| SASH3 | -0.193071511400628 | 0.00201434423574626 | 0.0418844137451324 |
| FURIN | -0.192293206788185 | 0.00269480736992309 | 0.048844453414623 |
| SHKBP1 | -0.191744710247523 | 0.00665069582283906 | 0.0743626983977651 |
| MSLN | -0.191474403598458 | 0.00192613553444327 | 0.0413709059436679 |
| C19orf48 | -0.191181147701034 | 0.00496178650301323 | 0.0636109406478712 |
| PIM2 | -0.19103099186761 | 0.00484347870183068 | 0.0629814901556248 |
| DYRK2 | -0.190023905393336 | 0.000615669867758003 | 0.0278046472365304 |
| LTA | -0.189969596724774 | 0.00513687227554942 | 0.0648281849118996 |
| PLA2G15 | -0.189866083491428 | 0.000780531243122303 | 0.029106626873633 |
| BAT3 | -0.18941633022229 | 0.00741709610698084 | 0.0788409845445741 |
| TRAPPC1 | -0.189006255431337 | 0.0063168824586081 | 0.0721694065929927 |
| RWDD2B | -0.187266221635399 | 0.00120916813888312 | 0.0335463547330807 |
| TNFRSF10C | -0.186729802930166 | 0.000149700871571019 | 0.0239757643173249 |
| ACMSD | -0.18616253458226 | 3,74E+14 | 0.0239757643173249 |
| HOXC10 | -0.185746460776223 | 0.0112607353727737 | 0.0982937603645469 |
| PARVB | -0.184960057294854 | 0.00738014615870892 | 0.0785987926793786 |
| TMIGD1 | -0.184959817570867 | 0.00408713515747979 | 0.0593492275292264 |
| SHC1 | -0.184770152908892 | 0.0097988011897209 | 0.0912895041829351 |
| CASZ1 | -0.184744908494142 | 0.00516880576144743 | 0.0648868331109003 |
| SLC35C1 | -0.184181321577643 | 0.00743412113837985 | 0.0788708607198668 |
| VHL | -0.183002417076671 | 0.000184392870560495 | 0.0239757643173249 |
| HM13 | -0.18245163265189 | 0.00712395368650974 | 0.0771537625671055 |
| BCORL1 | -0.182346487608318 | 0.000428670098238451 | 0.0256150411917438 |
| OR52N2 | -0.182277959799634 | 0.00580851749698582 | 0.069048712570502 |
| TEX261 | -0.182115196665427 | 0.00831693207546991 | 0.083249339343519 |
| YY1AP1 | -0.181861643481284 | 0.0042781274204804 | 0.0605044256935571 |
| CCNO | -0.181810789497735 | 0.00608520394990572 | 0.070604197382115 |
| FGR | -0.181091085495633 | 0.00213368100102193 | 0.0430602557342659 |
| ETNK2 | -0.180814528737265 | 0.00624814419807154 | 0.0716298955379469 |
| ZNF792 | -0.180074535538594 | 0.00454628528770671 | 0.0614063625819381 |
| SLC30A4 | -0.180071657035542 | 0.00575071405778005 | 0.0685719098895464 |
| C19orf44 | -0.179853374070868 | 0.00551202120666322 | 0.0671428233574088 |
| RSPO2 | -0.179340004079978 | 0.0013430710342467 | 0.035656715209044 |
| MIST | -0.17919268729415 | 0.00115196319297193 | 0.0333327826408104 |
| CAMK2B | -0.178803969453614 | 0.0024249845704056 | 0.0459324580617778 |
| HOXD3 | -0.178569906818997 | 0.00238281688562291 | 0.0454750349991065 |
| PRKCSH | -0.178364248006091 | 0.00454498827500097 | 0.0614063625819381 |
| DPM2 | -0.178047757662342 | 0.000776658675712006 | 0.029106626873633 |
| WDR21A | -0.177620610311051 | 0.00970469341276174 | 0.0909443189567861 |
| C19orf47 | -0.177329685737983 | 0.0053858775670372 | 0.0663607090902304 |
| PRR5 | -0.177195977785365 | 0.00630039359633294 | 0.0720765032663508 |
| TBN | -0.177129802362864 | 0.000192366065314932 | 0.0239757643173249 |
| FAM100B | -0.177128336946025 | 0.0066257531151255 | 0.0742708999019388 |
| SLC35C2 | -0.177055888371978 | 0.00256549519097758 | 0.0475674471659167 |
| SELPLG | -0.175903428870148 | 0.000639619749497119 | 0.0278046472365304 |
| RHBDD1 | -0.174036562748628 | 0.00579977433840807 | 0.0690086087470627 |
| ACSM5 | -0.173772785073696 | 0.00366496894494207 | 0.0565403828151119 |
| ANKK1 | -0.173771996807666 | 0.00951215576871665 | 0.0902732867309335 |
| PIGS | -0.17376919691052 | 0.0055700112613426 | 0.0674806458925102 |
| CDKN2D | -0.173749240553953 | 0.000571795115781542 | 0.0276956297030149 |
| SCAMP3 | -0.173579268155547 | 0.00416093675570869 | 0.0596627298595866 |
| DNASE2 | -0.173469512807111 | 0.00921062549205316 | 0.0886755765996049 |
| ITGA7 | -0.173044909493616 | 0.00157377125452662 | 0.0376555014846483 |
| RBMX2 | -0.172249810015167 | 0.00710569098819705 | 0.077056242468748 |
| PCNXL2 | -0.170984091030008 | 0.0020580322896882 | 0.0422416837165843 |
| PSORS1C1 | -0.170647916521361 | 0.00469710996934392 | 0.0621033300633034 |
| FLJ14082 | -0.170060932197907 | 0.00731460636973674 | 0.0781507943713978 |
| SNORD55 | -0.169425221358026 | 0.0057187885602378 | 0.0683380864132939 |
| BRUNOL6 | -0.169323778385424 | 0.00350806359381805 | 0.0551939759760825 |
| HCK | -0.16919591288428 | 0.00967995105386244 | 0.0909325424619606 |
| DNPEP | -0.168518865628107 | 0.00630442080714927 | 0.0720765032663508 |
| STK10 | -0.168252548128387 | 0.00775729124674971 | 0.0805036596592242 |
| CIB2 | -0.168096571338469 | 0.00922917891551752 | 0.0888028394379796 |
| TCEB3 | -0.167352749192246 | 0.0070008891019563 | 0.0765622856557754 |
| ISG20 | -0.167100005345909 | 0.0115052421238811 | 0.0990772169654038 |
| BDKRB1 | -0.166136181456186 | 0.00153558720008351 | 0.0373382380162451 |
| FKRP | -0.165196684468293 | 0.00994271044029948 | 0.0917441008809452 |
| PDK1 | -0.164686062434038 | 0.00178465124734754 | 0.039768814810371 |
| SPI1 | -0.164554893456079 | 0.0106792729004504 | 0.0954728126099659 |
| MGC33948 | -0.164002238775478 | 0.0043566696840885 | 0.0608597912300807 |
| SEC14L5 | -0.163202038545734 | 0.00076170113276527 | 0.0290452776012764 |
| FLJ16793 | -0.163072870949377 | 0.00865785476405653 | 0.0853783473948371 |
| OPA3 | -0.162523277970949 | 0.00414980520060293 | 0.0596303050885912 |
| C12orf34 | -0.162148014386508 | 0.000784536329834677 | 0.029106626873633 |
| C9orf25 | -0.161870901744369 | 0.00838102488813008 | 0.0836895862554369 |
| PHKA1 | -0.161016920061455 | 0.00748940560291654 | 0.0792049845401199 |
| SIX3 | -0.160852896049432 | 0.0060860039514263 | 0.070604197382115 |
| SHC2 | -0.160628786907682 | 0.00232823172300673 | 0.0448561866448727 |
| BLR1 | -0.160275350818716 | 0.00681508875526743 | 0.0751835271664935 |
| ZNF205 | -0.160159586044884 | 0.0115523640150752 | 0.0991043202782726 |
| C7orf29 | -0.159807639717511 | 0.00119134346812232 | 0.0335326583455989 |
| UNKL | -0.159726694985796 | 0.000796424567002809 | 0.029106626873633 |
| EDARADD | -0.159683429912384 | 0.00105389585892294 | 0.0324144728863388 |
| MCHR2 | -0.159512501628714 | 0.00470713477286668 | 0.0621371652887699 |
| RNU12 | -0.159437535287781 | 0.00144917922333749 | 0.0365131484210659 |
| EGLN2 | -0.158947080330509 | 0.00146881683101688 | 0.0368221761582937 |
| ZNF699 | -0.158181897783576 | 0.00296460388504233 | 0.0517825774086197 |
| C8orf58 | -0.156416727703497 | 0.00290738555656877 | 0.0511589217490949 |
| TRIM14 | -0.15399281461286 | 0.00805555220244852 | 0.0822288632498487 |
| RPS6KA2 | -0.153853869378713 | 0.00379268907233509 | 0.0573416006340508 |
| UNC45A | -0.153483791501546 | 0.00698355399281434 | 0.076428822987763 |
| UNC93B1 | -0.153288851180761 | 0.00983758557966584 | 0.0913819472986147 |
| MAP3K7IP1 | -0.152776336507843 | 0.00232721927392108 | 0.0448561866448727 |
| KCNIP3 | -0.152313639814899 | 0.00451123940097106 | 0.0613513815919643 |
| MADD | -0.151181213204628 | 0.00801708905295589 | 0.0820740863317982 |
| GPRIN3 | -0.150833924445203 | 0.005953993213698 | 0.0698450817725278 |
| PTPRU | -0.149849523813962 | 0.00378027910220813 | 0.0573382465694033 |
| SLCO3A1 | -0.149239272735427 | 0.00107493856199364 | 0.0324744597149658 |
| APOF | -0.148394086205297 | 0.000564496134402542 | 0.0276956297030149 |
| AGAP3 | -0.147012233360369 | 0.00319962672207756 | 0.0529960063837841 |
| CCM2 | -0.144896624918069 | 0.0109955957688259 | 0.0968939582678009 |
| ZNF688 | -0.144543083391437 | 0.00159046545608376 | 0.0379114033542458 |
| IGFL1 | -0.143789382266527 | 0.00825390029901231 | 0.0829487945839322 |
| KIAA1530 | -0.143410630528167 | 0.0103398784750429 | 0.094119096098697 |
| DNA2 | -0.143049264353331 | 0.00707270700693661 | 0.0769820180111139 |
| PDZD7 | -0.142557743938731 | 0.000979754239870564 | 0.0314845348974622 |
| HOXB6 | -0.142104907520361 | 0.000959705196575867 | 0.0313240249062782 |
| C17orf48 | -0.142041993494466 | 0.00381599810051506 | 0.0574331866014228 |
| FBS1 | -0.141522227285711 | 0.0111748830337914 | 0.0978236401205531 |
| C1orf183 | -0.141223697264953 | 0.00431725318804996 | 0.0606455667242866 |
| ADAMTS19 | -0.140553405798975 | 0.0106752565868102 | 0.0954728126099659 |
| CHST10 | -0.140179051156606 | 0.0102164171429136 | 0.0935610652120838 |
| HCN4 | -0.139406669982751 | 0.00641059647731419 | 0.0727408241045481 |
| RARA | -0.13933742509894 | 0.00516077170056866 | 0.064834872247295 |
| LYSMD4 | -0.139200239545906 | 0.0116140976417253 | 0.0992954644808212 |
| AAK1 | -0.138590528326803 | 0.00542221816071226 | 0.0666101897377871 |
| PTPRA | -0.137401817633677 | 0.000747202982604893 | 0.028858331434898 |
| LIN37 | -0.137089274643777 | 0.00980569707311594 | 0.0912895041829351 |
| CDK5R1 | -0.135319295448166 | 0.00339117773397437 | 0.0543306492394007 |
| C19orf45 | -0.135177433688141 | 0.00717419433723964 | 0.0772955591829716 |
| ABL1 | -0.133482583422297 | 0.00610068277545399 | 0.0706694262214385 |
| PHF19 | -0.133277463146925 | 0.0105514364629539 | 0.0950943212573529 |
| PILRA | -0.132145813666223 | 0.00800201525596279 | 0.0819887850378902 |
| GRB14 | -0.131753685610157 | 0.00341852933833907 | 0.0545527466858994 |
| ZNF407 | -0.130333928922464 | 0.0106128469322096 | 0.0953158786969703 |
| FAM26F | -0.130107399671127 | 0.000833068051594639 | 0.0297696230455384 |
| CDH23 | -0.129991357967824 | 0.00581628627066403 | 0.0690569909140324 |
| LMOD2 | -0.129903330664479 | 0.00853627398709122 | 0.0845299326526594 |
| C11orf31 | -0.129383316433202 | 0.010963436089068 | 0.0967129608577777 |
| DHDDS | -0.129126014804316 | 0.0114672381629798 | 0.0990453088884465 |
| ARID5A | -0.129012718749284 | 0.00857681804281922 | 0.0846795451605983 |
| KLRG1 | -0.128290733862013 | 0.0080470953687711 | 0.0822288632498487 |
| SH2D5 | -0.127965738603642 | 0.0106020841304094 | 0.0953158786969703 |
| sep-06 | -0.126416746175219 | 0.00575720257449374 | 0.0686001389083914 |
| OSCAR | -0.125844988792511 | 0.00818028461259192 | 0.0827442748264649 |
| MURC | -0.124740202264913 | 0.00992300861502197 | 0.091723643850332 |
| PRSS7 | -0.124114068211175 | 0.00196006260922271 | 0.0416163293279608 |
| UBE2D2 | -0.123627567942256 | 0.00104883099815988 | 0.0324144728863388 |
| TNFRSF4 | -0.123043516040585 | 0.00923881737062157 | 0.0888442252751974 |
| sep-01 | -0.122846583396019 | 0.00257762177065083 | 0.0475688381311017 |
| OR13C2 | -0.122346990081499 | 0.0106906367369486 | 0.0955213843925106 |
| C6orf182 | -0.122184432017636 | 0.00366277078819755 | 0.0565403828151119 |
| MTMR14 | -0.120860609167967 | 0.00279187668482068 | 0.0499995933806226 |
| AFAP1 | -0.120268596421521 | 0.00734761121420673 | 0.0784027796613366 |
| SHOX2 | -0.119523537607336 | 0.00165367892116932 | 0.0384994955549433 |
| UPP1 | -0.11942651369445 | 0.00259341000696617 | 0.0477015502496782 |
| SOHLH2 | -0.118926980254888 | 0.00125821312701495 | 0.0342785854538313 |
| LSR | -0.118693938295438 | 0.0112869484008688 | 0.0984193520591214 |
| GABRG2 | -0.117970615030359 | 0.00791948502352926 | 0.0815689242363127 |
| TCP11L1 | -0.117395760555963 | 0.00184001396308563 | 0.0404787333017101 |
| CYP4Z2P | -0.116824502573915 | 0.00299780441897151 | 0.051833729843994 |
| AKAP2 | -0.1156793999552 | 0.00770888867940519 | 0.080352010618271 |
| B3GALT1 | -0.115615120340092 | 0.0104423307237315 | 0.094571837446809 |
| REST | -0.115233643019171 | 0.0110085076432057 | 0.0969564117612707 |
| PML | -0.115183072704022 | 0.0016793172151295 | 0.0386171286585011 |
| C4orf12 | -0.114878259000273 | 0.0107468745344678 | 0.0957156091496793 |
| TMEM84 | -0.112448444369951 | 0.00999526788151328 | 0.0920270665568984 |
| SLC13A3 | -0.111435820041011 | 0.00431714959813149 | 0.0606455667242866 |
| XKR3 | -0.111270882737414 | 0.00490038634909272 | 0.0632933272718721 |
| CASC2 | -0.111010688566115 | 0.00369715212800539 | 0.0568787378214213 |
| GRAP | -0.110129877307262 | 0.00157670819024389 | 0.0376555014846483 |
| ACOT6 | -0.109575080855222 | 0.0107431513092606 | 0.0957156091496793 |
| CSDE1 | -0.107665845365359 | 0.00414145835411695 | 0.0596085197171515 |
| ZNF713 | -0.107211287866027 | 0.00383005631438085 | 0.0574888344537273 |
| TRAF3IP1 | -0.106628594106846 | 0.0116890208253131 | 0.0996978781352102 |
| FAM5B | -0.105453645088041 | 0.00930969246380064 | 0.0891733015067734 |
| SAPS2 | -0.104628990132317 | 0.0107283405477289 | 0.0956528959600942 |
| FSCN3 | -0.104474426807089 | 0.0115277342555942 | 0.0991043202782726 |
| LCP2 | -0.10278125894551 | 0.00950397482575341 | 0.0902470992296014 |
| FLJ42102 | -0.102436809009461 | 0.00944153762283559 | 0.0900136513572286 |
| CBX2 | -0.101631285140022 | 0.00993690900869446 | 0.0917414239371758 |
| PRDM2 | -0.0978220302679181 | 0.00322778028523761 | 0.0531450352404207 |
| ERI3 | -0.0922138857333349 | 0.00730464340765701 | 0.0781446620590351 |
| CD274 | -0.089293246486334 | 0.00957365891985378 | 0.0905472308976625 |
| KCNMA1 | -0.0891517470875956 | 0.0115156867694216 | 0.0990871179958914 |
| SMR3B | -0.0883759518586741 | 0.0107172925573303 | 0.0956334714360448 |
| BICD1 | -0.0856085083449465 | 0.00279221352668615 | 0.0499995933806226 |
| ZNF452 | -0.0855888991618864 | 0.00931053694694054 | 0.0891733015067734 |
| ZSCAN2 | -0.0824518335300237 | 0.0036319037634188 | 0.0561864963251574 |
| FLJ22531 | 0.0747081823003795 | 0.0105776066741796 | 0.095270971612081 |
| C1orf156 | 0.081196137956737 | 0.00986041136088742 | 0.0914538772428076 |
| ZNF174 | 0.0841318315297084 | 0.00980382300129176 | 0.0912895041829351 |
| AAA1 | 0.0852775094445433 | 0.00647049850605834 | 0.0730718576199777 |
| NEDD9 | 0.0869111992731356 | 0.00709938859242702 | 0.0770380850779271 |
| SNORD83A | 0.0884923764811699 | 0.00844295507641494 | 0.0841565450311396 |
| GLT8D1 | 0.0909411506926443 | 0.00485811806799365 | 0.0629814901556248 |
| RBP3 | 0.0932077587194395 | 0.00855566025406945 | 0.0845897276567415 |
| ANO7 | 0.0945402880343955 | 0.00539425581750442 | 0.066414779835931 |
| SLC12A6 | 0.0953649597632733 | 0.00458677977077741 | 0.0615268054694274 |
| C5orf42 | 0.096193528978278 | 0.00682082290674628 | 0.0751835271664935 |
| NCAM2 | 0.0963566536437595 | 0.00743317436989841 | 0.0788708607198668 |
| C18orf54 | 0.097420594678075 | 0.00351826475040122 | 0.0552500330520554 |
| BACH2 | 0.0995039325581972 | 0.00992396266817541 | 0.091723643850332 |
| OPRD1 | 0.100421764192706 | 0.00826788612099547 | 0.0829766207804367 |
| NDC80 | 0.10104861684115 | 0.00674975710184576 | 0.0748606499828485 |
| GTPBP8 | 0.103232830075406 | 0.0111386798937208 | 0.0976481790110299 |
| RIMBP2 | 0.103243835988357 | 0.00456692564221509 | 0.0614598093641915 |
| FGF2 | 0.103974163658427 | 0.00938611727705174 | 0.0896393047583496 |
| ATP5G2 | 0.10404127537256 | 0.00120327333938249 | 0.0335326583455989 |
| CSNK1G3 | 0.105809045660326 | 0.00888820742148541 | 0.0866235952798865 |
| KLK8 | 0.106081297447055 | 0.00594088296233391 | 0.0698398436072017 |
| ADAM10 | 0.106678935585013 | 0.00802495192580386 | 0.0821040871278003 |
| SLC22A3 | 0.107141662336986 | 0.00805690712246205 | 0.0822288632498487 |
| TMTC2 | 0.107175802118329 | 0.00742636716192861 | 0.0788708607198668 |
| FANCF | 0.108381790504331 | 0.0111210114560554 | 0.0975913683429741 |
| NOS2A | 0.110048705275648 | 0.00411227377786352 | 0.059472553697929 |
| UEVLD | 0.111458647653601 | 0.000400791833155823 | 0.0256150411917438 |
| SGCB | 0.112015758667729 | 0.00562140320376 | 0.0676600706650679 |
| ZC3H14 | 0.112934132869074 | 0.00137943334297931 | 0.0360397922668013 |
| TMEM88 | 0.113432854764847 | 0.00350323331003222 | 0.0551872990775131 |
| UBE2CBP | 0.11379885100956 | 0.00143221303612805 | 0.0362611369418848 |
| C6orf199 | 0.114276775886653 | 0.0113969187154404 | 0.0990074683178536 |
| SEC61A2 | 0.114565380059726 | 0.0106161023131192 | 0.0953158786969703 |
| FANCC | 0.115104527468982 | 0.00745536073040157 | 0.0789955026850824 |
| NCOA1 | 0.11630296396804 | 0.00956719625939435 | 0.0905375491380775 |
| METTL10 | 0.117668831712568 | 0.0105893189153756 | 0.0952809744136986 |
| C11orf47 | 0.117833969631479 | 0.0023858941208189 | 0.0454750349991065 |
| HNMT | 0.121176461092258 | 0.0116543727947708 | 0.0995886496620917 |
| ZNF543 | 0.122161441456418 | 0.00787306742631986 | 0.081299677654169 |
| MOCS2 | 0.12291618493853 | 0.00378667114344112 | 0.0573382465694033 |
| BRWD3 | 0.123537524646151 | 0.00315775325376801 | 0.0526692992607438 |
| SOCS4 | 0.123551187059105 | 0.00713589096019634 | 0.0772327964391601 |
| SLC24A1 | 0.124016152931135 | 0.0114317165749678 | 0.0990074683178536 |
| GCOM1 | 0.124854276134353 | 0.00817149878786173 | 0.0827442748264649 |
| THAP1 | 0.125431565612576 | 0.00454848282251169 | 0.0614063625819381 |
| PSMA1 | 0.125521909458925 | 0.0037212920162513 | 0.0569477178767905 |
| UGP2 | 0.126220045369101 | 0.00775491930153311 | 0.0805036596592242 |
| SNX7 | 0.126701571350662 | 0.00558634394820795 | 0.0675291243042718 |
| PDAP1 | 0.128863071617265 | 0.0115043679675266 | 0.0990772169654038 |
| CHRDL2 | 0.12921188714692 | 0.0100604855033442 | 0.0923190968515258 |
| ZNF835 | 0.129775331305193 | 0.00916055802784183 | 0.0884525407786117 |
| ZNF550 | 0.129817198563212 | 0.00676667439145204 | 0.0749421569661415 |
| FLJ25006 | 0.130276953268876 | 0.00464134364773453 | 0.0617388604775303 |
| BBS7 | 0.130321263182017 | 0.00285500910203877 | 0.0505042311504116 |
| SEL1L | 0.130458415083443 | 0.00883133109008265 | 0.0864901618664725 |
| CD164 | 0.130617978127393 | 0.00179069168872125 | 0.0398050163156387 |
| CLEC12B | 0.131673741437077 | 0.00635203133452128 | 0.0722857676521301 |
| C20orf118 | 0.132218268988838 | 0.00143336706162202 | 0.0362611369418848 |
| C10orf47 | 0.132391673808578 | 0.00829945973964078 | 0.0831744773185193 |
| C12orf48 | 0.132414191956936 | 0.000744968077840799 | 0.028858331434898 |
| ZNF311 | 0.132478422802103 | 0.00820682432647444 | 0.0827442748264649 |
| C14orf45 | 0.13366264506934 | 0.00735559288478118 | 0.0784376676233616 |
| C14orf101 | 0.133684546541398 | 0.00762966102051764 | 0.0798750843070509 |
| ZNF425 | 0.135474766122406 | 0.00917683154659753 | 0.0884525407786117 |
| ELOVL7 | 0.1360476356965 | 0.00172535522383269 | 0.039128423781906 |
| TOP1 | 0.137745530858207 | 0.00634211740071544 | 0.0722593335060296 |
| PGBD3 | 0.137896762105413 | 0.0111525796909885 | 0.0977083376506288 |
| GTDC1 | 0.138037404693444 | 0.00141841363490427 | 0.0362130573107614 |
| HOXD10 | 0.138872311206578 | 0.00281273755914992 | 0.0500881522232834 |
| BAI3 | 0.140648601627269 | 0.00155229390096128 | 0.0373943332494955 |
| NSF | 0.141367878055508 | 0.00100011062775007 | 0.0317101743038622 |
| MRPL35 | 0.14161784018012 | 0.00725235246010296 | 0.0779358676893957 |
| CSPG5 | 0.141878794339256 | 0.00120140057280616 | 0.0335326583455989 |
| FAM122A | 0.142534301893586 | 0.00908174468913034 | 0.0880458486285752 |
| HHAT | 0.142665735326732 | 0.00292574221091617 | 0.0512975380067001 |
| NID1 | 0.142893494092799 | 0.00561298754604757 | 0.0676076633078928 |
| ENPP5 | 0.143096842012857 | 0.0106137820361507 | 0.0953158786969703 |
| HNRNPA1 | 0.143207077500823 | 0.00352290930884155 | 0.0552708278557742 |
| PLSCR1 | 0.143555702437048 | 0.00583418341314134 | 0.0691215780036661 |
| SLC4A7 | 0.143685771428493 | 0.00653747296150244 | 0.0737281672880554 |
| HK2 | 0.14377535165435 | 0.0104114418425014 | 0.094513893498951 |
| C7 | 0.14384157882554 | 0.00478146048810731 | 0.0625813706760912 |
| GALNT12 | 0.143865074378343 | 0.00560241022853534 | 0.0675291243042718 |
| TLL1 | 0.143992829179946 | 0.000435216588068847 | 0.0256150411917438 |
| ZNF239 | 0.144209486173133 | 0.00187038370940468 | 0.0406454402437993 |
| ARMCX6 | 0.144531654368031 | 0.00886557634814033 | 0.0866235952798865 |
| TMEM33 | 0.144656371422738 | 0.00422928296411069 | 0.0601313925876867 |
| PLP1 | 0.144708686464991 | 0.0071132111080508 | 0.0770875729847745 |
| ARFIP1 | 0.145250716397672 | 0.00819669954903764 | 0.0827442748264649 |
| TRHR | 0.145474850297662 | 0.00307093208489418 | 0.0521465049409614 |
| PGM2L1 | 0.14602289278104 | 0.00979647966113156 | 0.0912895041829351 |
| THUMPD3 | 0.146081454994275 | 0.00177383899704659 | 0.0396339918722651 |
| GOLM1 | 0.146411031280899 | 0.00465841696262033 | 0.0617388604775303 |
| SH3GL3 | 0.146411750888299 | 0.0083231933854956 | 0.0832619453695671 |
| ZNF81 | 0.146727076956415 | 0.00127787756339132 | 0.034587886049125 |
| WDR7 | 0.147315849438765 | 0.00465618551216 | 0.0617388604775303 |
| BNC1 | 0.147393788987809 | 0.00389302044460896 | 0.0581070091478916 |
| UBXN8 | 0.147457176913271 | 0.0116911124666545 | 0.0996978781352102 |
| RBM18 | 0.147620172442614 | 0.00375922919951788 | 0.057231226636824 |
| PLXNA2 | 0.147680256509548 | 0.0096925426208273 | 0.0909443189567861 |
| ARHGAP20 | 0.14826177047158 | 0.00975747581279529 | 0.0911189020435559 |
| LPPR5 | 0.148343483809042 | 0.00141146902675011 | 0.0362130573107614 |
| USP6NL | 0.149417047427064 | 0.00390402507276055 | 0.0581273715216208 |
| CDC2 | 0.150165350771941 | 0.00161201895182761 | 0.0381274715578325 |
| ZNF684 | 0.151476351051164 | 0.0111774939029972 | 0.0978236401205531 |
| CCNT2 | 0.152152816520718 | 0.00446459143789186 | 0.061098881984569 |
| RPS6KC1 | 0.152220909568779 | 0.000900275172225443 | 0.0306331953292983 |
| UBE1DC1 | 0.152686138973921 | 0.000467929613106923 | 0.025891456847287 |
| C12orf5 | 0.15305352499525 | 0.00953064268003206 | 0.0903254705065237 |
| ZNF782 | 0.15324649123383 | 0.00644313796203775 | 0.0728122705472372 |
| KCNE1 | 0.153323696494019 | 0.00978297045801176 | 0.0912821335448788 |
| ESCO1 | 0.153355762081482 | 0.00619904990404744 | 0.0715103151093373 |
| MAOB | 0.154183683895344 | 0.0108913207777783 | 0.0965073072038063 |
| C2orf63 | 0.154794171621197 | 0.0114717863472861 | 0.0990453088884465 |
| ZNF485 | 0.15504287936503 | 0.000600685974428647 | 0.0276956297030149 |
| SNX22 | 0.155252871167793 | 0.00315118570345726 | 0.0526692992607438 |
| SLC6A19 | 0.155449090328862 | 0.00654310359149564 | 0.0737406125572217 |
| OXGR1 | 0.156459269650077 | 0.00303087135600044 | 0.0520111234256005 |
| PHF14 | 0.156928157253474 | 0.00331702188483305 | 0.0538101935477045 |
| C14orf126 | 0.157599020109297 | 0.00551108999717707 | 0.0671428233574088 |
| TIPRL | 0.157612685295758 | 0.0114821794121712 | 0.0990772169654038 |
| HEXA | 0.158204252231973 | 0.00946483277893985 | 0.0900785910209195 |
| CCDC5 | 0.158251410451616 | 0.00937893532366542 | 0.0896221339826261 |
| GULP1 | 0.158480140569412 | 0.00732854437014111 | 0.0782494865845856 |
| SNX24 | 0.158695566575012 | 0.00042234582354085 | 0.0256150411917438 |
| SLC30A5 | 0.160717597785543 | 0.0065760642177691 | 0.0739140228795615 |
| PIK3CA | 0.160732870530067 | 0.00110916077414923 | 0.0327204957769608 |
| CFI | 0.161902799630666 | 0.00902642007662178 | 0.0875604828644791 |
| ARMCX3 | 0.161939708984202 | 0.00668168788860262 | 0.0744965683815668 |
| SIAH1 | 0.162031971543702 | 0.00176172301514331 | 0.0394692346030627 |
| SF3B1 | 0.162331834198539 | 0.00613478970549039 | 0.0708672515181076 |
| TCEAL1 | 0.162561776008129 | 0.000723932195096129 | 0.0286236943457723 |
| CLK1 | 0.163290573311281 | 0.00465302710621343 | 0.0617388604775303 |
| C18orf1 | 0.163306767032718 | 0.00912601128216255 | 0.0883206882574871 |
| ZHX1 | 0.163617092701222 | 0.00184236977401441 | 0.0404787333017101 |
| RPAP3 | 0.163680526874884 | 0.00815301779333948 | 0.0827442748264649 |
| ZNF383 | 0.164591214150272 | 0.0112142616214104 | 0.0979394538037763 |
| ADAMTS1 | 0.165613659736548 | 0.00298242255405179 | 0.051833729843994 |
| TNFRSF17 | 0.16588572920442 | 0.00310181113253657 | 0.0523765739733842 |
| DQX1 | 0.166658276144513 | 0.00226225544761345 | 0.0440954381510228 |
| POP4 | 0.167417962090153 | 0.00385408245272489 | 0.0576933961403404 |
| MMRN2 | 0.167481202366858 | 0.000335920303157233 | 0.0247792613550641 |
| PPFIA1 | 0.16788978820187 | 0.00371552085514432 | 0.0569477178767905 |
| ASCC3 | 0.167967406538323 | 0.00299917981496553 | 0.051833729843994 |
| CTDSPL2 | 0.168343901559624 | 0.0110398202573921 | 0.0970268468873011 |
| MCMDC1 | 0.168461969977052 | 0.00154394433668388 | 0.0373382380162451 |
| PTPLAD1 | 0.168744156044024 | 0.00967139649228119 | 0.0909325424619606 |
| KCNK1 | 0.168779830579891 | 0.00530433618735293 | 0.0656475689031055 |
| RFX5 | 0.168971044483693 | 0.0084726021480015 | 0.0842502600690758 |
| VAPA | 0.170196162524161 | 0.000819312808349016 | 0.029584123661123 |
| NNT | 0.170607093060032 | 0.00496464584351085 | 0.0636109406478712 |
| NUP62CL | 0.171231992223523 | 0.00846465735144662 | 0.0842502600690758 |
| ZNF274 | 0.171754518040912 | 0.00214069851240608 | 0.043135763153786 |
| ZNF235 | 0.17187935027204 | 0.00588420335671352 | 0.0696151023993271 |
| APPBP2 | 0.172265173997942 | 0.00157365194252386 | 0.0376555014846483 |
| DHX40 | 0.172566972838577 | 0.000788218178118594 | 0.029106626873633 |
| PAG1 | 0.172800020998037 | 0.000216184483722983 | 0.0239757643173249 |
| CCDC91 | 0.173322008134747 | 0.00737150010451875 | 0.0785569723046217 |
| TMSB15A | 0.174038615144069 | 0.0106492501230716 | 0.095364576630716 |
| CD302 | 0.174137749466696 | 0.00946999485081155 | 0.0900785910209195 |
| RANBP6 | 0.174605730650965 | 0.00115370925448432 | 0.0333327826408104 |
| NGDN | 0.174869651091865 | 0.00116313023901214 | 0.0334071875607094 |
| C1GALT1C1 | 0.175168321516914 | 0.00106535675372215 | 0.0324144728863388 |
| USP1 | 0.175521189531071 | 0.00460816165021615 | 0.0616106045420321 |
| CEP120 | 0.175596187952815 | 0.00384027361938258 | 0.0575384290443226 |
| RWDD3 | 0.17563010477447 | 0.00377413460017614 | 0.0573382465694033 |
| HIF1A | 0.176661173580115 | 0.000115615674594613 | 0.0239757643173249 |
| PRPF38B | 0.176685198651243 | 0.00777851653436901 | 0.0805732334978883 |
| MECP2 | 0.177414668944046 | 0.00220100646265789 | 0.0435647486057114 |
| SNX5 | 0.177878074009469 | 0.00032691716724953 | 0.0247792613550641 |
| C1orf31 | 0.178452788070843 | 0.00755005062710151 | 0.0795394520114679 |
| SBF2 | 0.178962610114031 | 0.00238522447543452 | 0.0454750349991065 |
| MTBP | 0.178977518285911 | 0.0049257211742627 | 0.0634134220160687 |
| RASGRP1 | 0.179310818201001 | 0.0056779139601782 | 0.068094060361042 |
| ZNF502 | 0.179808521322765 | 0.00820368522220428 | 0.0827442748264649 |
| APBB2 | 0.180077481607015 | 0.00191749987170358 | 0.0412682425059276 |
| RMI1 | 0.180174549427064 | 0.00447434108579098 | 0.0610991646546978 |
| SPAG9 | 0.180282845487085 | 0.00398306115018343 | 0.058795379716606 |
| mar-05 | 0.180825636559733 | 0.000751718584841384 | 0.0289655267668279 |
| MGAT2 | 0.181585129918225 | 0.00254371866008846 | 0.0474694403764939 |
| TBC1D19 | 0.181865932218198 | 0.00180843449876843 | 0.0400841553482014 |
| KIAA0753 | 0.181937768184017 | 0.00404194861357289 | 0.0590195408960828 |
| RABL3 | 0.181942748016507 | 0.00783777771680222 | 0.0810038908588828 |
| GNAQ | 0.18224362084328 | 0.000983935775376929 | 0.0314972979171622 |
| FAM8A1 | 0.182520916276461 | 0.00476127753041823 | 0.0624120526276684 |
| ICK | 0.182774959070729 | 0.00191824395962275 | 0.0412682425059276 |
| C1RL | 0.182808703115448 | 0.007634342132195 | 0.0798750843070509 |
| TJP2 | 0.182955504924624 | 0.00535394640741627 | 0.0661140889449935 |
| MCFD2 | 0.183893426742693 | 0.00367557497095101 | 0.0566515008948615 |
| C3orf38 | 0.18446684477497 | 0.00529836916595047 | 0.0656273476573112 |
| LYST | 0.184616916972796 | 0.00137337805355457 | 0.0360397922668013 |
| OSBPL10 | 0.18506460462424 | 0.0103414530076004 | 0.094119096098697 |
| LRRC57 | 0.185564333598534 | 0.00275683006548986 | 0.0496110197515073 |
| RRM2B | 0.185573438427264 | 0.00960046717833005 | 0.0905864719066928 |
| SRI | 0.186181177412422 | 9,89E+14 | 0.0239757643173249 |
| HMGN2 | 0.186187850142093 | 0.00807788484086352 | 0.0823924455030724 |
| AMY2B | 0.186250101936729 | 0.00550835665400476 | 0.0671428233574088 |
| ATP1A2 | 0.186660386098093 | 0.000252241670268889 | 0.0239757643173249 |
| ZNF333 | 0.186707939482943 | 0.00137723009235616 | 0.0360397922668013 |
| SMC4 | 0.186899017917938 | 0.00855755745367972 | 0.0845897276567415 |
| MEX3B | 0.187550942510508 | 0.00174478024698317 | 0.0393544877930649 |
| RIC3 | 0.187834264336326 | 0.000674013825267072 | 0.0279546184311492 |
| STAMBP | 0.187986730939984 | 0.00702917041594711 | 0.0766842899409964 |
| PGBD2 | 0.188479352388464 | 0.00368039244212663 | 0.0566732771430526 |
| EPB41L5 | 0.188586431655615 | 0.00893268571634603 | 0.086891251737291 |
| CHODL | 0.188700527365335 | 0.00296455493857665 | 0.0517825774086197 |
| ATM | 0.189124023982229 | 0.00885439882859055 | 0.0866235952798865 |
| UHRF2 | 0.189209465145023 | 0.00238180561332519 | 0.0454750349991065 |
| YIPF4 | 0.190003997628085 | 0.00122994777730446 | 0.033840844133901 |
| TASP1 | 0.191018692565709 | 0.00436404072400475 | 0.0608597912300807 |
| C12orf26 | 0.19104400883334 | 0.00597573089001214 | 0.0698539440977121 |
| ZNF230 | 0.191070808605632 | 0.00917140579781936 | 0.0884525407786117 |
| SLC16A14 | 0.191189771420568 | 0.00312416455411569 | 0.0524771374044498 |
| RAF1 | 0.191251022600777 | 0.00607284036017075 | 0.070604197382115 |
| PSIP1 | 0.191891882950811 | 0.00151907439369856 | 0.0372715043388988 |
| FBXO11 | 0.192100338532251 | 0.00444564903099296 | 0.061098881984569 |
| EAF1 | 0.192217098678495 | 0.00306752323496946 | 0.0521465049409614 |
| TPM1 | 0.192460154280431 | 0.00111029752644756 | 0.0327204957769608 |
| C16orf87 | 0.192693592891569 | 0.00945930680849146 | 0.0900785910209195 |
| NHLRC3 | 0.192943641749765 | 0.00934509505510861 | 0.0893500587520609 |
| MRPL42 | 0.193001683969498 | 0.00353727732762775 | 0.0553508848719995 |
| SNORD21 | 0.193211443111267 | 0.000493475644311462 | 0.0263977209883983 |
| DOPEY1 | 0.193384425481913 | 0.00399018504375958 | 0.058795379716606 |
| FDX1 | 0.193744505744958 | 0.00623364932822022 | 0.0716298955379469 |
| VAMP4 | 0.194782531764989 | 0.000126709018598976 | 0.0239757643173249 |
| MRPS21 | 0.195142620439507 | 0.00201687215734985 | 0.0418844137451324 |
| CHCHD7 | 0.195380855753349 | 0.000258227563774654 | 0.0239757643173249 |
| COG3 | 0.19572867434024 | 0.00116401350385747 | 0.0334071875607094 |
| TANK | 0.19640957439242 | 0.00482553454833936 | 0.0628929549444057 |
| AGPAT5 | 0.196658254332686 | 0.00281282672774212 | 0.0500881522232834 |
| TMEM135 | 0.196748168977786 | 0.00515283778532667 | 0.064834872247295 |
| C5orf24 | 0.197169471737023 | 0.0106341772275189 | 0.095364576630716 |
| CLCN3 | 0.197540420801645 | 0.00115084792287111 | 0.0333327826408104 |
| TBCE | 0.197754463548954 | 0.00893655070132417 | 0.086891251737291 |
| ZBTB38 | 0.197808975548683 | 0.0044610761832234 | 0.061098881984569 |
| CDC7 | 0.197934892233132 | 0.00342253410652474 | 0.0545527466858994 |
| CSPP1 | 0.199454592434223 | 0.0044669794169903 | 0.061098881984569 |
| BHLHB9 | 0.199497650976881 | 0.0026785208254584 | 0.0486223093354204 |
| PAPPA | 0.199730236642879 | 0.00773625880744427 | 0.0804358301740895 |
| COX11 | 0.200290153742724 | 0.000181493555970332 | 0.0239757643173249 |
| BTN2A1 | 0.20035339723525 | 0.00144683831682015 | 0.0365131484210659 |
| ATXN1 | 0.200544281284411 | 0.00318597061277994 | 0.0528223773110904 |
| ZAK | 0.200640109881208 | 0.00138146010817175 | 0.0360397922668013 |
| KIF16B | 0.200883620951174 | 0.00309936612662285 | 0.0523765739733842 |
| GMNN | 0.201081427714775 | 0.0105207905510112 | 0.0950239172610595 |
| CGRRF1 | 0.201173254909354 | 0.010349767673173 | 0.0941432965506215 |
| RNF146 | 0.201714672088711 | 0.00328478910163608 | 0.053606469986112 |
| COPS8 | 0.201939998486142 | 0.00286274109183702 | 0.0505872486355829 |
| RPE | 0.202523736195446 | 0.00378239663222685 | 0.0573382465694033 |
| GOLGA7 | 0.202890864560725 | 0.000635670138407765 | 0.0278046472365304 |
| DLEU1 | 0.203465317227445 | 0.0102536497006672 | 0.0935610652120838 |
| DUSP11 | 0.203546054780421 | 0.00626978464572086 | 0.0717791163773517 |
| ZNF236 | 0.204618862181993 | 0.00765998802378511 | 0.0800427875981965 |
| CCDC126 | 0.205119652654121 | 0.00266815716971525 | 0.0485120114582663 |
| MPP5 | 0.205670180576583 | 0.00814122066554844 | 0.0827442748264649 |
| MUDENG | 0.205788619274022 | 0.00403397100795924 | 0.058954768567594 |
| CENPK | 0.206591689424708 | 0.009323986203632 | 0.089238238512631 |
| C17orf71 | 0.206694122558342 | 0.00216038783549744 | 0.0433150489997752 |
| C14orf43 | 0.206858082269685 | 0.00815418937222668 | 0.0827442748264649 |
| AKTIP | 0.207806304329523 | 0.00761482848514024 | 0.0798214325967535 |
| PHLDB2 | 0.208524546893011 | 0.000242572453023194 | 0.0239757643173249 |
| PCNA | 0.208732641360896 | 0.000478658689737979 | 0.026123778850421 |
| TMEM64 | 0.208778940453949 | 0.000225579113652097 | 0.0239757643173249 |
| SKAP2 | 0.209405187211237 | 0.0105824838706439 | 0.095270971612081 |
| ADH5 | 0.209424395818896 | 0.00171794328088891 | 0.0390667812208698 |
| PDE12 | 0.209497895086366 | 0.0113983465642199 | 0.0990074683178536 |
| FLJ20125 | 0.209575002740365 | 0.000900778339383582 | 0.0306331953292983 |
| NBN | 0.209707288964575 | 0.000507007086670418 | 0.0264565516135291 |
| ELMO2 | 0.210057614453835 | 0.00867819951287103 | 0.0854776976871309 |
| ZNF614 | 0.210435630233454 | 0.00716840983769339 | 0.0772955591829716 |
| NUMB | 0.210730766197137 | 0.00976000254377389 | 0.0911189020435559 |
| WSB2 | 0.211965401733223 | 0.00514220861463011 | 0.064834872247295 |
| C17orf95 | 0.212364400037602 | 0.000622665526333623 | 0.0278046472365304 |
| SNAP23 | 0.212407469214934 | 7,25E+14 | 0.0239757643173249 |
| CALCRL | 0.212973434946004 | 0.00490559168185681 | 0.0632933272718721 |
| STOM | 0.212988690080588 | 0.00449338384935507 | 0.0612085659217385 |
| RNASEL | 0.213231997923303 | 0.00634164664827183 | 0.0722593335060296 |
| NEK1 | 0.213462564363073 | 0.0110846781164672 | 0.0973696843940435 |
| FAM177A1 | 0.213465966993236 | 0.000251685388194863 | 0.0239757643173249 |
| ADNP | 0.213707053385259 | 0.000634671869281554 | 0.0278046472365304 |
| PDS5B | 0.213743273427269 | 0.0022978104467937 | 0.0444759915085209 |
| DIS3 | 0.214171337944239 | 0.00172231739078449 | 0.0391128175811713 |
| ZNF75D | 0.214215078138007 | 0.0113714905278904 | 0.0989492061303002 |
| HAT1 | 0.214865376308906 | 0.00438491767637981 | 0.0608597912300807 |
| ACYP1 | 0.214883892332938 | 0.00307628666658801 | 0.0521465049409614 |
| IGSF3 | 0.215115016628509 | 0.00458475014317832 | 0.0615268054694274 |
| C3orf57 | 0.215145129787867 | 0.000568592212806905 | 0.0276956297030149 |
| PRDM10 | 0.215488954789047 | 0.00194185701167219 | 0.0415477529772433 |
| ABI1 | 0.21570375283208 | 0.00671487784888233 | 0.0746665709235106 |
| MAPKAPK5 | 0.216842492514984 | 0.00438855590162741 | 0.0608597912300807 |
| IMPAD1 | 0.216925430535036 | 0.00529875977720932 | 0.0656273476573112 |
| PARP9 | 0.217799201352747 | 0.0115640413165864 | 0.0991220554870741 |
| ZNF22 | 0.218143408852024 | 0.011470843146227 | 0.0990453088884465 |
| PAIP2 | 0.218978738678549 | 0.00375710637473142 | 0.057231226636824 |
| ABT1 | 0.219213039975794 | 0.00010319621154603 | 0.0239757643173249 |
| APAF1 | 0.219297372246566 | 0.00927139774042988 | 0.0889637803952702 |
| PKD2 | 0.219738721676297 | 0.00484448305588569 | 0.0629814901556248 |
| CKAP2 | 0.21976853478686 | 0.000893163135276379 | 0.0306272188766164 |
| IGSF10 | 0.221040964872183 | 0.0018475461594725 | 0.0404787333017101 |
| ZNF30 | 0.221580106293923 | 0.00349049586300113 | 0.0551872990775131 |
| CCDC89 | 0.221814099175744 | 0.00755687980224148 | 0.0795394520114679 |
| CART1 | 0.221956148294723 | 0.00292751026334641 | 0.0512975380067001 |
| CCNG2 | 0.222776926678943 | 0.000766784903563932 | 0.029106626873633 |
| CWF19L2 | 0.223000610012225 | 0.000433103441005948 | 0.0256150411917438 |
| RCBTB2 | 0.22308569727197 | 0.00317236498085286 | 0.0527544330382385 |
| PCBD2 | 0.223419808829487 | 0.0107586236604026 | 0.0957690104016373 |
| TRMT5 | 0.223495692221355 | 0.0037322642864228 | 0.0570498359153295 |
| SEH1L | 0.223884614675785 | 0.00510743504067953 | 0.0646527480510657 |
| PDCD6IP | 0.223930066076035 | 0.00312413825435896 | 0.0524771374044498 |
| KCTD18 | 0.224135717043008 | 0.00161097463092374 | 0.0381274715578325 |
| QTRTD1 | 0.224154101914162 | 0.0013257823620263 | 0.0354589750767884 |
| ARMCX5 | 0.22490175064051 | 0.00420373338651179 | 0.0599103989314 |
| BCCIP | 0.226556055046727 | 0.0075768947664526 | 0.0796243625520012 |
| CYP2U1 | 0.226615354486483 | 0.00512491755216348 | 0.0647263866261861 |
| REV3L | 0.226658644585402 | 0.00961045953305415 | 0.0905864719066928 |
| C6orf115 | 0.22750863390207 | 0.011505147705243 | 0.0990772169654038 |
| GFPT1 | 0.227513979421533 | 0.00292758987783042 | 0.0512975380067001 |
| ACADL | 0.227971062529252 | 0.00818514854779517 | 0.0827442748264649 |
| UBE3B | 0.22892818716942 | 0.00642485496923599 | 0.0727815399630157 |
| KLHL12 | 0.229429361180412 | 0.00548515056694826 | 0.0671366296598682 |
| GLRB | 0.229641130144531 | 0.00541941866729207 | 0.0666101897377871 |
| BCHE | 0.229665369093917 | 0.00818650260392061 | 0.0827442748264649 |
| ASTE1 | 0.230242782336969 | 0.00225685302051486 | 0.0440954381510228 |
| PCDH20 | 0.23112706196581 | 0.00201793338071875 | 0.0418844137451324 |
| SHQ1 | 0.231844273786277 | 0.00198193740619034 | 0.0418281871627141 |
| C5orf44 | 0.23209256342716 | 0.0026213356056397 | 0.0480030280434306 |
| ME2 | 0.232301558084471 | 0.000800472565546589 | 0.029106626873633 |
| C8orf38 | 0.23342353776538 | 0.00474137893766635 | 0.062331226366849 |
| ENPP4 | 0.233424580282622 | 0.0105132569346121 | 0.0950074239595838 |
| JMJD2C | 0.233961155699546 | 0.0032589380179635 | 0.0532890788281143 |
| FILIP1L | 0.233993273587442 | 0.000903342692620439 | 0.0306331953292983 |
| PRKD1 | 0.234691738552823 | 0.00118805000422917 | 0.0335326583455989 |
| HNRNPH2 | 0.2349768738834 | 0.00263610705941945 | 0.0481675500670649 |
| ATMIN | 0.235044982566585 | 0.00567124371627763 | 0.0680631023079722 |
| NETO2 | 0.235718720438317 | 0.00214564531366904 | 0.043135763153786 |
| WRB | 0.235752073680351 | 0.00230153328380767 | 0.0444963101536149 |
| ANKRD17 | 0.235980667398741 | 0.00448951072294101 | 0.0612058931155414 |
| DPYD | 0.236565788955524 | 0.000164645931289321 | 0.0239757643173249 |
| FBXO3 | 0.236829483169775 | 0.000367037595393266 | 0.024952349438868 |
| NFU1 | 0.236890295056545 | 0.00472763271447819 | 0.0622596314598132 |
| PPIL1 | 0.236958278266256 | 0.00266953036740189 | 0.0485120114582663 |
| USPL1 | 0.237011831519402 | 0.0108263615374857 | 0.0960637602094817 |
| BTBD3 | 0.238075185384814 | 0.00168280581388915 | 0.0386171286585011 |
| SLBP | 0.238170766125704 | 0.00204020098027907 | 0.0420832534296474 |
| ARFGEF1 | 0.238199733914748 | 0.000947996577897656 | 0.0311864644974 |
| C3orf64 | 0.238453273083795 | 0.00181899222811969 | 0.0402110818449939 |
| TMCO1 | 0.238463470575648 | 0.00389916671982428 | 0.0581070091478916 |
| BRPF3 | 0.239424996538021 | 0.00415183969708502 | 0.0596303050885912 |
| POLR2G | 0.239503125030544 | 0.0101589704063005 | 0.0931201659599548 |
| N4BP2L1 | 0.239584214301939 | 0.00437009660739335 | 0.0608597912300807 |
| SNX3 | 0.239619172631209 | 0.00131655414181145 | 0.0353473552332152 |
| WNT5A | 0.239725271247737 | 0.000445270232587174 | 0.0256705006083636 |
| PRPSAP2 | 0.240414197293337 | 0.00140883590547447 | 0.0362130573107614 |
| ZNF318 | 0.240863313020701 | 0.00108341923103749 | 0.0324969469383994 |
| SPPL2A | 0.240899784937627 | 0.00444874011661375 | 0.061098881984569 |
| SAP30 | 0.241026421520904 | 0.00635312255647401 | 0.0722857676521301 |
| TMEM144 | 0.241095229274253 | 0.0106468022725958 | 0.095364576630716 |
| GBP1 | 0.241287176236136 | 0.00303393012412941 | 0.0520111234256005 |
| RNF138 | 0.241654626844305 | 0.0043907394939344 | 0.0608597912300807 |
| CTTNBP2NL | 0.242201217915091 | 0.00690083999304988 | 0.075822694735517 |
| INTS2 | 0.242331738748377 | 0.000459919422132413 | 0.025891456847287 |
| ZCRB1 | 0.243479779051912 | 0.00132710209496811 | 0.0354589750767884 |
| STAG2 | 0.24363986674006 | 0.00307276408542444 | 0.0521465049409614 |
| KIAA1199 | 0.244159452785059 | 0.00871138897220331 | 0.0857031801603406 |
| CHUK | 0.244163538031908 | 0.00833631104181297 | 0.0833430832444556 |
| TMEM199 | 0.244251258653193 | 0.00055948046909833 | 0.0275535854692627 |
| GXYLT1 | 0.244438118282027 | 0.00438451287387037 | 0.0608597912300807 |
| DEPDC6 | 0.244673835046568 | 0.000350313567046554 | 0.0248077604528682 |
| LRIG3 | 0.244815077454966 | 0.000344735991556405 | 0.0248077604528682 |
| C13orf23 | 0.246154394076113 | 0.00478214006367824 | 0.0625813706760912 |
| YWHAB | 0.246664824870843 | 0.00993386523324127 | 0.0917414239371758 |
| STRBP | 0.246762608156241 | 0.00310244514824924 | 0.0523765739733842 |
| PPP2R3C | 0.247313149999567 | 0.00321656541310647 | 0.0531188040513332 |
| HLTF | 0.247546092289307 | 0.00240582413618051 | 0.0456640234559415 |
| KITLG | 0.247582000323886 | 6,56E+14 | 0.0239757643173249 |
| CSTF3 | 0.24773187015237 | 0.00232056242137346 | 0.0448121601695855 |
| ARFGAP3 | 0.247926075737859 | 0.00300072698451075 | 0.051833729843994 |
| C13orf1 | 0.248508705395398 | 0.00885932031079122 | 0.0866235952798865 |
| NCOA6 | 0.249074918504675 | 0.00200074725284508 | 0.0418397471995718 |
| CNOT8 | 0.249270983507703 | 0.000435779276508324 | 0.0256150411917438 |
| SLC22A4 | 0.249323293530119 | 0.000779576571026468 | 0.029106626873633 |
| SEC62 | 0.249930677084156 | 0.00145051124283122 | 0.0365131484210659 |
| WDR51B | 0.250004094657864 | 0.000718885905528316 | 0.0285598443518481 |
| CCDC55 | 0.250497748154189 | 0.00641884904870712 | 0.0727815399630157 |
| CBL | 0.250971852628288 | 0.00194877944120942 | 0.0415988656406715 |
| B3GNT2 | 0.251127505590393 | 0.00516032478709645 | 0.064834872247295 |
| DYNC1I1 | 0.251232462695655 | 0.00290253080772216 | 0.0511275426723206 |
| FIGNL1 | 0.251580235815983 | 0.00127411365825353 | 0.034555767304929 |
| EHHADH | 0.252490047151072 | 0.000520175111674986 | 0.0267541840887701 |
| DNAJC19 | 0.252930900788696 | 0.010956458503541 | 0.0967129608577777 |
| MATN2 | 0.252989131355428 | 0.00622383685505215 | 0.0716298955379469 |
| SFRS3 | 0.253135395643884 | 0.00812629959096301 | 0.0827341791994925 |
| ABCD3 | 0.253317354809872 | 0.00103419728849644 | 0.0324144728863388 |
| FAM102B | 0.253351419675604 | 0.00323702312720211 | 0.0532172467740518 |
| WDR37 | 0.254133254558907 | 0.00166637954098288 | 0.0385993730251762 |
| CD2AP | 0.254446818769502 | 0.00408825690095499 | 0.0593492275292264 |
| NET1 | 0.254575368609919 | 0.00433440164614639 | 0.0607314473097 |
| PELI2 | 0.25498020182284 | 0.00447378930298647 | 0.0610991646546978 |
| PURA | 0.255215164490558 | 0.00560229872042357 | 0.0675291243042718 |
| C3orf70 | 0.255227577610589 | 0.00253532686015727 | 0.0474191583305371 |
| PPM1D | 0.255672391081873 | 0.000254052565529416 | 0.0239757643173249 |
| PAICS | 0.256255531769694 | 0.00153260297160289 | 0.0373382380162451 |
| PREI3 | 0.25672171758771 | 0.000317594505013456 | 0.0247792613550641 |
| WAC | 0.256943963655197 | 0.00396827093546178 | 0.0587163004370638 |
| FAM133B | 0.257050549474154 | 0.00300178514775983 | 0.051833729843994 |
| ACSL3 | 0.257062860690492 | 0.00299996451043569 | 0.051833729843994 |
| HNRPH3 | 0.257461952241854 | 0.0024434998414997 | 0.0462210208654591 |
| SNX13 | 0.257466532936798 | 0.000592065362672872 | 0.0276956297030149 |
| GOLT1B | 0.257867695326518 | 0.00400789668515406 | 0.0588217851090614 |
| DNAJC10 | 0.258172949758987 | 0.00281343399788357 | 0.0500881522232834 |
| RNF219 | 0.258932317685747 | 0.00594095990314776 | 0.0698398436072017 |
| TADA1L | 0.259242436614105 | 0.000327839666365293 | 0.0247792613550641 |
| PDGFC | 0.259712222131007 | 0.00595311790752748 | 0.0698450817725278 |
| TMEM20 | 0.25973948331321 | 0.00393494134068783 | 0.0583788177870674 |
| CDH13 | 0.259809771282174 | 0.00624376664656822 | 0.0716298955379469 |
| GPR115 | 0.260357321638691 | 0.000580591496408451 | 0.0276956297030149 |
| TMEM30A | 0.260462678713973 | 0.00745456306317121 | 0.0789955026850824 |
| OSGIN2 | 0.261307005511644 | 0.00675862488629298 | 0.0749028427811138 |
| CCDC104 | 0.261666920282577 | 0.0104175106499601 | 0.094513893498951 |
| IK | 0.262358857745335 | 0.00356385090307905 | 0.0553730541650761 |
| ISCA1 | 0.262643028553611 | 0.00270164760550405 | 0.0488822022187178 |
| C4orf31 | 0.263512539496229 | 0.000167733333265456 | 0.0239757643173249 |
| DONSON | 0.264186546045873 | 0.000132887905687202 | 0.0239757643173249 |
| C6orf173 | 0.264622517137527 | 0.000721318095931486 | 0.0285882405354179 |
| MRPL39 | 0.265028001577229 | 0.000762512694446582 | 0.0290452776012764 |
| USP16 | 0.266025293960453 | 0.00563430507840246 | 0.0677663600687048 |
| UBE3C | 0.266072490973969 | 0.0031687994843847 | 0.0527525711318772 |
| KIAA1671 | 0.26637865606555 | 0.00528616551204877 | 0.0656273476573112 |
| AMD1 | 0.266558490299306 | 0.0110266697990764 | 0.097011597643616 |
| MRPL51 | 0.266823161023556 | 0.00542613344253511 | 0.0666101897377871 |
| ARHGAP12 | 0.26685482662256 | 2,66E+14 | 0.0239757643173249 |
| ZNF45 | 0.267367414571043 | 0.00170443484577151 | 0.0388657841680993 |
| C4orf18 | 0.268077966473271 | 0.000469051597746495 | 0.025891456847287 |
| MTSS1 | 0.268082332321897 | 0.00636765607994022 | 0.0723728230693263 |
| ARMCX1 | 0.268103464661256 | 0.00060839773743156 | 0.0278046472365304 |
| PRMT6 | 0.268170458279781 | 0.00289059020798058 | 0.050971148942844 |
| PRPF4B | 0.268222272676845 | 0.000667901859888323 | 0.0279344079389473 |
| SLC40A1 | 0.268444234403789 | 0.0111852603640301 | 0.0978401702678116 |
| COMMD8 | 0.269422368538302 | 0.00334328180306151 | 0.0539816734387994 |
| KLHDC5 | 0.269828709200091 | 0.00972316172989841 | 0.0909443189567861 |
| SPTLC1 | 0.269828794744332 | 0.000186483128530612 | 0.0239757643173249 |
| CLNS1A | 0.269916813744901 | 0.005203538518635 | 0.0651263926174422 |
| LARS | 0.271030797312471 | 0.00213412897877985 | 0.0430602557342659 |
| ARPP19 | 0.271618366283076 | 0.00286839031041383 | 0.0506333246099138 |
| PPCS | 0.272640901997106 | 0.00825700784726515 | 0.0829487945839322 |
| HMGB2 | 0.273219092728445 | 0.00974737604746851 | 0.0911032126255815 |
| ZNF330 | 0.273374655784068 | 0.00138544597408406 | 0.0360397922668013 |
| HBXIP | 0.273488659314888 | 0.000549363890398493 | 0.0275377535242345 |
| SCO1 | 0.273729989326297 | 0.00518189471656159 | 0.0649839281489116 |
| RBM16 | 0.274550132398958 | 0.00334575001790062 | 0.0539816734387994 |
| LSM5 | 0.275029317721088 | 0.00408629854316274 | 0.0593492275292264 |
| UBR5 | 0.275183076583191 | 0.00208591009185124 | 0.0424475053654717 |
| FASTKD3 | 0.275520734222627 | 0.00353798759484606 | 0.0553508848719995 |
| RAB18 | 0.275898554705009 | 0.0043391697510353 | 0.0607314473097 |
| IARS | 0.276098801888674 | 4,64E+13 | 0.0239757643173249 |
| NUP35 | 0.276112264879165 | 0.000129319203756958 | 0.0239757643173249 |
| MRPL22 | 0.276119710706192 | 0.00654743634263929 | 0.0737406125572217 |
| STAMBPL1 | 0.276282670159807 | 0.00761320851345588 | 0.0798214325967535 |
| EID2 | 0.276346468593982 | 0.00205244092572902 | 0.0421789279625744 |
| BP75 | 0.277395071930994 | 0.000121623426475522 | 0.0239757643173249 |
| SRP54 | 0.278175833660369 | 0.000733503956787038 | 0.0287947341006642 |
| JAZF1 | 0.279278679463737 | 0.000571949297199988 | 0.0276956297030149 |
| HPS3 | 0.279952787603123 | 0.00343862432432762 | 0.0546176913194251 |
| RTN4 | 0.2801878721837 | 0.00426816285282917 | 0.0604662458282505 |
| PRPS2 | 0.280602088819852 | 0.0015919783097812 | 0.0379114033542458 |
| RB1 | 0.280873751063251 | 0.00199823738184272 | 0.0418397471995718 |
| TIMM9 | 0.281383605766699 | 0.00850503511239807 | 0.0844214755402375 |
| MTX2 | 0.28193906129452 | 0.000295760422234697 | 0.0247792613550641 |
| TLE4 | 0.282873886548189 | 0.00768169903221064 | 0.080219298676398 |
| TC2N | 0.283719389180799 | 0.00049713413079983 | 0.0263977209883983 |
| NME7 | 0.28405788665053 | 0.000242878900867752 | 0.0239757643173249 |
| CIR1 | 0.284139094025638 | 0.00773603167957015 | 0.0804358301740895 |
| GNG2 | 0.284455832913267 | 0.000443264671536648 | 0.0256705006083636 |
| ARID2 | 0.284605007286845 | 0.000208291035092928 | 0.0239757643173249 |
| UBE2V2 | 0.285532198550946 | 0.000957604873897186 | 0.0313240249062782 |
| THUMPD1 | 0.287227542910848 | 0.000136861068752659 | 0.0239757643173249 |
| AGGF1 | 0.287458897420527 | 0.00299966946390312 | 0.051833729843994 |
| RAB4A | 0.287550089867137 | 0.00700494779849489 | 0.0765622856557754 |
| MST4 | 0.288739297816251 | 0.00474430276383502 | 0.062331226366849 |
| SLTM | 0.288914093793676 | 0.00208478204006322 | 0.0424475053654717 |
| SPIN4 | 0.289016388154582 | 0.000889452271580597 | 0.0306272188766164 |
| BECN1 | 0.289103563198183 | 0.0104320067111522 | 0.0945297679443875 |
| C5orf35 | 0.289384777079531 | 0.00278017841505544 | 0.0499232469223439 |
| PPP4R1 | 0.290391005902264 | 0.00234955097418661 | 0.0451623851227603 |
| GOLGA5 | 0.290892679101939 | 0.00706265744629199 | 0.0769404423108485 |
| DNMT1 | 0.292917576168564 | 0.00779439088348238 | 0.0806874568696814 |
| UBE2T | 0.293145222504427 | 0.000295250724941333 | 0.0247792613550641 |
| SLFN11 | 0.293397748211247 | 0.0113080348068564 | 0.0985515954947286 |
| CROP | 0.293429063117567 | 0.0109435728176402 | 0.0967129608577777 |
| TMEM106B | 0.293783115776655 | 0.00175798743742543 | 0.0394644162156032 |
| ATP11B | 0.294018147315788 | 0.000188335639561515 | 0.0239757643173249 |
| C2orf30 | 0.295106909882527 | 0.00822937265347331 | 0.0827710798729406 |
| PIK3CB | 0.295704266151173 | 0.00325844580588235 | 0.0532890788281143 |
| MEX3C | 0.296683263644668 | 0.000600383736761683 | 0.0276956297030149 |
| CREBZF | 0.297007262040214 | 0.00202732517318391 | 0.0419215587985335 |
| HDAC4 | 0.297441874026225 | 0.00331202101851605 | 0.0538101935477045 |
| SAR1B | 0.297587619465075 | 0.00370761354504923 | 0.056934441947315 |
| OSTC | 0.298316278691885 | 0.00355516362976511 | 0.0553730541650761 |
| RTCD1 | 0.298520807359088 | 0.000957984447347673 | 0.0313240249062782 |
| PNPLA8 | 0.298802093695446 | 0.0011368818666434 | 0.0332592891953357 |
| LEO1 | 0.298890905675489 | 0.00503927971025276 | 0.064033473325853 |
| CBFB | 0.298931785481044 | 0.000390719246546107 | 0.0254059085078379 |
| C3orf26 | 0.299104703724607 | 0.000797358402368445 | 0.029106626873633 |
| NACAP1 | 0.299126973672959 | 0.00917397286641896 | 0.0884525407786117 |
| SENP7 | 0.30020261357527 | 0.00054793680339836 | 0.0275377535242345 |
| IQCB1 | 0.300399028030482 | 0.00046933940897341 | 0.025891456847287 |
| CRLF3 | 0.301518040378511 | 0.00445789039991288 | 0.061098881984569 |
| C2orf64 | 0.301747390189829 | 0.0102528112479766 | 0.0935610652120838 |
| FBXO21 | 0.301969404342193 | 0.000903574366615732 | 0.0306331953292983 |
| TSC22D2 | 0.302004535142813 | 0.00269662697873595 | 0.048844453414623 |
| ATF4 | 0.303211182929033 | 0.00986181122496934 | 0.0914538772428076 |
| ATAD1 | 0.303438725201011 | 0.00049938234139375 | 0.0263977209883983 |
| HERC3 | 0.30354710907614 | 0.00751181049659129 | 0.0792908037579319 |
| ARL2BP | 0.303578922063131 | 0.00166886661678591 | 0.0385993730251762 |
| ZMYM6 | 0.3038557475255 | 0.00275643662229055 | 0.0496110197515073 |
| DUSP12 | 0.303860096258577 | 6,68E+14 | 0.0239757643173249 |
| DYRK1A | 0.304638410276084 | 0.000774224611116776 | 0.029106626873633 |
| COPS5 | 0.304830246299518 | 0.000947868984442208 | 0.0311864644974 |
| UTP14C | 0.304839675082883 | 0.00228974636590919 | 0.0443714994259888 |
| MYO1B | 0.305128723458501 | 0.00199435472181568 | 0.0418397471995718 |
| JMJD1A | 0.305164496490822 | 0.00491080393176908 | 0.0632933272718721 |
| TBK1 | 0.305195669187463 | 0.00925658268446649 | 0.088935975594015 |
| ALDH3A2 | 0.306319722762212 | 0.000501515459456834 | 0.0263977209883983 |
| C2orf47 | 0.306587023516109 | 0.00498118529940053 | 0.0636109406478712 |
| ZBED5 | 0.306943824139998 | 0.010419772205145 | 0.094513893498951 |
| ARL4A | 0.307183368243139 | 0.00792364234591599 | 0.0815689242363127 |
| WDR35 | 0.307233017644402 | 0.00682056647808382 | 0.0751835271664935 |
| CRIM1 | 0.307565190177094 | 0.000587696688919122 | 0.0276956297030149 |
| EXOSC3 | 0.308066080591624 | 0.00445064636753943 | 0.061098881984569 |
| SHOC2 | 0.308072347574214 | 0.00342988308699806 | 0.0545527466858994 |
| BRIX1 | 0.308133302143442 | 0.00520301071475241 | 0.0651263926174422 |
| IMPA1 | 0.30818822730854 | 0.000148203598227258 | 0.0239757643173249 |
| TWSG1 | 0.30882912262218 | 0.00305761985719011 | 0.0521465049409614 |
| CPNE8 | 0.309053661115675 | 0.00276420296815573 | 0.0496899812180564 |
| ACVR2A | 0.310021890123531 | 0.00408948479971301 | 0.0593492275292264 |
| GOLGA4 | 0.310082157246358 | 0.00211290191616256 | 0.0427355592909381 |
| PTGR1 | 0.31011662385671 | 0.00709189830569751 | 0.0770070053467976 |
| DPY19L1 | 0.310177950890777 | 0.00322301995669322 | 0.0531450352404207 |
| GTF3C3 | 0.310441439201893 | 0.00410451443846274 | 0.0594317107781622 |
| MRPL45 | 0.311742717243565 | 0.00402195336461886 | 0.0588521882250363 |
| ZFX | 0.311832196263482 | 0.00120199723955304 | 0.0335326583455989 |
| FAM76B | 0.312169824865853 | 0.0109925961834755 | 0.0968939582678009 |
| DDX50 | 0.312361683950213 | 0.00101667403002623 | 0.0320521892117738 |
| REXO2 | 0.312766090305221 | 0.00552195873941444 | 0.0671428233574088 |
| MRS2 | 0.313220248120788 | 3,71E+14 | 0.0239757643173249 |
| RDH14 | 0.313320967156272 | 0.000931010547684571 | 0.0310573177890929 |
| HEATR5B | 0.314289133615411 | 0.00416133447105207 | 0.0596627298595866 |
| DIP2B | 0.314566324080879 | 0.00665181172139086 | 0.0743626983977651 |
| AASDH | 0.314613480140667 | 0.00818326889892193 | 0.0827442748264649 |
| DDX47 | 0.314656997204257 | 0.000330514423010187 | 0.0247792613550641 |
| SPRY2 | 0.31478169283224 | 0.00983320627547085 | 0.0913819472986147 |
| PDGFD | 0.314857995035826 | 0.00163627215311782 | 0.0383625158602805 |
| ANLN | 0.315249384023272 | 0.00297247450165857 | 0.051833729843994 |
| PNMA1 | 0.315300412038054 | 0.000107239514086468 | 0.0239757643173249 |
| STAU2 | 0.315714008198249 | 0.000924443019122964 | 0.0309763572525477 |
| SFRS7 | 0.315995948016055 | 0.0106501710895411 | 0.095364576630716 |
| MLLT10 | 0.316020660574027 | 0.000189623092591057 | 0.0239757643173249 |
| NUDT21 | 0.31660442010332 | 0.000654065860891844 | 0.0278046472365304 |
| DHX36 | 0.316699053745776 | 0.000208944271745197 | 0.0239757643173249 |
| CASK | 0.316748729543802 | 0.000188494430103078 | 0.0239757643173249 |
| GPBP1 | 0.317024973930345 | 0.00027412410368364 | 0.0244391352696618 |
| ABCC4 | 0.317288163731352 | 0.000892672339040148 | 0.0306272188766164 |
| C6orf72 | 0.317492252380928 | 0.000837390021572806 | 0.0297767990079848 |
| TARS | 0.318359275761416 | 0.00250300236279655 | 0.0469729169460105 |
| CYP39A1 | 0.31945620268818 | 0.00119509251535812 | 0.0335326583455989 |
| YTHDF3 | 0.319735688462046 | 0.000394149150069907 | 0.025430258728929 |
| AZIN1 | 0.32020355869182 | 0.00528405562467165 | 0.0656273476573112 |
| KIAA0895 | 0.320707121188357 | 0.0014898255523961 | 0.0370474414028692 |
| MAT2B | 0.321029202124096 | 0.000107485721642217 | 0.0239757643173249 |
| EDG1 | 0.321086776777744 | 6,16E+13 | 0.0239757643173249 |
| FBXO28 | 0.321200515907662 | 0.00410587933406744 | 0.0594317107781622 |
| PDIK1L | 0.321245744684524 | 0.0022163596623468 | 0.043728616421763 |
| CHD1 | 0.322031271120245 | 0.00967358176532968 | 0.0909325424619606 |
| SMNDC1 | 0.322687971752276 | 0.00444039889511895 | 0.061098881984569 |
| MAK16 | 0.323003219390884 | 0.00473666938058948 | 0.0623293268848162 |
| UTX | 0.323199166743873 | 0.00457090255874499 | 0.0614598093641915 |
| DDAH1 | 0.32349534064726 | 0.00301543933583819 | 0.0519099819851916 |
| YME1L1 | 0.32374270390326 | 0.00175913713997222 | 0.0394644162156032 |
| MGC16169 | 0.324429107351746 | 0.00120665999934001 | 0.0335326583455989 |
| AGTR1 | 0.324739976613968 | 0.00793356043073277 | 0.0816205481643867 |
| ZNF273 | 0.324839358794108 | 0.000326691493486917 | 0.0247792613550641 |
| TCF12 | 0.325551071340373 | 0.000459834927284698 | 0.025891456847287 |
| COPS4 | 0.325836849636402 | 0.000556464977221812 | 0.0275377535242345 |
| NRP1 | 0.325970725068392 | 0.0006464050484616 | 0.0278046472365304 |
| GALIG | 0.325976817997406 | 0.000458183799245321 | 0.025891456847287 |
| CDK6 | 0.327027154655016 | 0.0030003086585597 | 0.051833729843994 |
| RDH10 | 0.32748618712403 | 0.00168158875605956 | 0.0386171286585011 |
| RIOK2 | 0.327516906528728 | 0.00799132248724809 | 0.0819887850378902 |
| DHRS7 | 0.328083065862256 | 0.00634143727663458 | 0.0722593335060296 |
| JMJD1C | 0.3285014452396 | 0.000121601608419399 | 0.0239757643173249 |
| RHOT1 | 0.328819648102186 | 0.000357063341779057 | 0.0248077604528682 |
| RFC4 | 0.328975200652541 | 0.0034507581213343 | 0.0547060187502197 |
| TGDS | 0.329554455228866 | 0.000685461113879578 | 0.0280690958739751 |
| C1orf57 | 0.329627357089422 | 0.00412384813722651 | 0.0594849012931304 |
| NPTN | 0.329970128415831 | 0.0060865687398375 | 0.070604197382115 |
| C20orf72 | 0.330115488068878 | 0.00849314006823281 | 0.0844040654183901 |
| IRS1 | 0.330639332073165 | 0.00109910562968528 | 0.0325546482415323 |
| RIOK3 | 0.331526945006982 | 0.000470239398084645 | 0.025891456847287 |
| FAM134B | 0.332490745386296 | 0.00719816069244029 | 0.0775036111813461 |
| HSF2 | 0.33272914932413 | 0.000440161058554336 | 0.0256403684309919 |
| ANKRD46 | 0.333658072834367 | 0.00183654697280875 | 0.0404787333017101 |
| MTERFD1 | 0.333938948706547 | 0.0116705377516851 | 0.0996756138607232 |
| PDCD10 | 0.33405887277766 | 0.000211507434056542 | 0.0239757643173249 |
| IL18R1 | 0.334695910680543 | 0.0021118269518761 | 0.0427355592909381 |
| RP2 | 0.334765549199609 | 0.00133289031912868 | 0.0354996676035456 |
| TCHP | 0.334902252473095 | 0.000673961290694902 | 0.0279546184311492 |
| MOBKL2B | 0.335173691943866 | 0.00428378062441153 | 0.0605127884208247 |
| CRY1 | 0.335606175675264 | 0.00199649220073486 | 0.0418397471995718 |
| CCND2 | 0.335873311235521 | 0.00818484284978723 | 0.0827442748264649 |
| NCBP2 | 0.336255243020325 | 8,35E+14 | 0.0239757643173249 |
| KPNA3 | 0.336884492166907 | 0.00578266966288754 | 0.0688543055854264 |
| LRIG1 | 0.337389347506921 | 0.00022019780539573 | 0.0239757643173249 |
| CLPX | 0.338381328219785 | 0.00174375655159278 | 0.0393544877930649 |
| PGM2 | 0.338934946147226 | 0.00963752342298379 | 0.0907901612331569 |
| BTG3 | 0.339959120165159 | 0.000123476633896557 | 0.0239757643173249 |
| SNCA | 0.341298707889853 | 0.00516064865605242 | 0.064834872247295 |
| SP3 | 0.34152849684666 | 0.00129447985494429 | 0.0349803760801991 |
| NTN4 | 0.342316618982095 | 0.00370539652057872 | 0.056934441947315 |
| RABEP1 | 0.343409983956715 | 0.00108746214011439 | 0.0324969469383994 |
| TRIP12 | 0.344267633463555 | 0.0115846987330488 | 0.0991286993899792 |
| CLDN12 | 0.344616861033257 | 0.00443348965878666 | 0.061098881984569 |
| C4orf41 | 0.344762756695433 | 0.000208370181638703 | 0.0239757643173249 |
| SYNCRIP | 0.345573625127556 | 0.00313451086527136 | 0.0525105985735697 |
| C5orf53 | 0.346213339173011 | 0.00507903908157325 | 0.0644403083474606 |
| USP34 | 0.346640234853257 | 0.0062472711917278 | 0.0716298955379469 |
| EPRS | 0.34668216661261 | 0.00185376409348771 | 0.0404803351713351 |
| SNX1 | 0.347250863504503 | 0.00235963905504683 | 0.0453039812114296 |
| IVNS1ABP | 0.347526679194946 | 0.00603812595800287 | 0.0702871641237173 |
| NUP54 | 0.347565239864736 | 0.00115541364794831 | 0.0333327826408104 |
| ARL6IP6 | 0.347627137514554 | 0.00762637527321298 | 0.0798750843070509 |
| GGH | 0.347908487508501 | 0.00421693048082033 | 0.0600470699604237 |
| MTFR1 | 0.348236091786622 | 0.00313336912117726 | 0.0525105985735697 |
| SF3B14 | 0.348527933426893 | 0.00318217505997153 | 0.0528120498985903 |
| C14orf156 | 0.348881043776567 | 0.00371621628343075 | 0.0569477178767905 |
| KIF5B | 0.349227515092178 | 0.0012452639550914 | 0.0340931970336374 |
| SLC25A27 | 0.349513777979094 | 0.000760613881649548 | 0.0290452776012764 |
| SLC35A3 | 0.349813797049111 | 0.00537363595698254 | 0.0663080386508017 |
| C12orf31 | 0.350384775858637 | 0.00380119915985433 | 0.0573632542175886 |
| PSMC2 | 0.350584231622403 | 0.0044086444091872 | 0.061002738848986 |
| ZMIZ1 | 0.351168371550176 | 0.00592519096057237 | 0.0698398436072017 |
| KAL1 | 0.352458750813188 | 0.000190683021640453 | 0.0239757643173249 |
| SNX4 | 0.352915772070804 | 0.000384001223637178 | 0.0252651556073695 |
| EFR3A | 0.353432219464541 | 0.0098503149495033 | 0.091449159313682 |
| SLC39A6 | 0.35431326610281 | 0.000110153006582608 | 0.0239757643173249 |
| KLHL20 | 0.355332360227588 | 0.00393262117444756 | 0.0583788177870674 |
| RSBN1 | 0.355404527473847 | 0.00866613703144434 | 0.0854094239345308 |
| EHBP1 | 0.356470338655638 | 0.000603427314255833 | 0.0276956297030149 |
| DHX29 | 0.357278608124132 | 0.00164132070984497 | 0.0383727872697743 |
| HSPA2 | 0.357504280662931 | 0.00874303365006959 | 0.0859103615809321 |
| PIGF | 0.357703917811868 | 0.000294650688766497 | 0.0247792613550641 |
| KIF21A | 0.358019583105874 | 0.000245017175466615 | 0.0239757643173249 |
| XPR1 | 0.358569318726342 | 0.00323856007341791 | 0.0532172467740518 |
| SRGN | 0.359008887031141 | 0.00795635186473751 | 0.0818044676593086 |
| ZNF512 | 0.359201313818787 | 0.00160605391564904 | 0.0381274715578325 |
| AP4E1 | 0.359493174051471 | 0.00518434798640843 | 0.0649839281489116 |
| FAM135A | 0.359554069930755 | 0.00049522749502958 | 0.0263977209883983 |
| RBM35A | 0.360486799755324 | 0.00151825740817702 | 0.0372715043388988 |
| COPB1 | 0.360546856073107 | 0.0039314650286115 | 0.0583788177870674 |
| MFAP1 | 0.360901114577506 | 0.00387647987695723 | 0.0579765355182659 |
| EPS15 | 0.360977553325934 | 0.00332349629267447 | 0.0538160693461665 |
| STT3B | 0.361213499949553 | 0.000206147452448449 | 0.0239757643173249 |
| TSC22D1 | 0.361264509460631 | 1,28E+14 | 0.0239757643173249 |
| ZZZ3 | 0.361614374444433 | 0.000691597853064926 | 0.0280690958739751 |
| MBNL2 | 0.361763384538876 | 0.00891264271507835 | 0.0868109131861874 |
| EIF3J | 0.3626135457592 | 0.00683641305684169 | 0.0752140989717031 |
| CP110 | 0.362843256772809 | 0.0107024298515576 | 0.0955754545649295 |
| LACTB2 | 0.36303347780219 | 0.0037890226616811 | 0.0573382465694033 |
| GTF2H5 | 0.363439721612268 | 0.00168574089908736 | 0.0386171286585011 |
| CD46 | 0.363633704903783 | 0.00149115617805613 | 0.0370474414028692 |
| MRPL18 | 0.363708286240072 | 0.00334669512128085 | 0.0539816734387994 |
| TM2D3 | 0.364081259277498 | 1,62E+13 | 0.0239757643173249 |
| ATP5L | 0.364151722445071 | 0.00782946098144155 | 0.081000128960271 |
| ZEB2 | 0.364615523496548 | 0.000834306695844296 | 0.0297696230455384 |
| TSPAN13 | 0.365025829797348 | 0.000515342643577396 | 0.0266409740527619 |
| PRKAB2 | 0.370016825031833 | 0.000796169365256084 | 0.029106626873633 |
| ENOPH1 | 0.370310358727057 | 0.00105981159233009 | 0.0324144728863388 |
| DSC3 | 0.370341023499176 | 0.00673016076158474 | 0.0747865527619089 |
| SPATS2L | 0.370409573807387 | 0.00197361300841435 | 0.0417442975070714 |
| SYPL1 | 0.371228313341815 | 0.000317904101764133 | 0.0247792613550641 |
| PGAP1 | 0.371790160905392 | 0.00125412497892628 | 0.0342785854538313 |
| ALDH5A1 | 0.371978111053525 | 0.000435316293122967 | 0.0256150411917438 |
| METAP1 | 0.373649178914081 | 0.00109616138190221 | 0.0325253161553371 |
| CMAS | 0.373942397032804 | 0.00452407493461999 | 0.0613755104822203 |
| PPP1CB | 0.373986135849617 | 9,79E+14 | 0.0239757643173249 |
| CLASP2 | 0.374036823239816 | 0.00221813398005217 | 0.043728616421763 |
| GBAS | 0.374224742791212 | 0.00542262265892462 | 0.0666101897377871 |
| EFNB2 | 0.374338477805987 | 0.00879471881618869 | 0.0862680550467159 |
| AASDHPPT | 0.374376991133449 | 8,48E+13 | 0.0239757643173249 |
| HADHB | 0.374607516353968 | 0.00725090384516123 | 0.0779358676893957 |
| GNA13 | 0.375319241965185 | 0.00117194403700518 | 0.0334960839340747 |
| ZCCHC11 | 0.375695242763148 | 0.000535616270035772 | 0.0271825257043155 |
| C14orf142 | 0.375756228445598 | 0.000113134434927907 | 0.0239757643173249 |
| ACVR1 | 0.376744289545093 | 0.00398384393337135 | 0.058795379716606 |
| ERRFI1 | 0.377123732238047 | 0.000175466878061214 | 0.0239757643173249 |
| ARF4 | 0.377246997800024 | 0.0109308303941416 | 0.0966840894650842 |
| RSL24D1 | 0.377355997832143 | 0.0115886368504686 | 0.0991286993899792 |
| ARV1 | 0.377486223918854 | 0.00185336091679836 | 0.0404803351713351 |
| SLC25A46 | 0.378054785507153 | 0.0114607328290479 | 0.0990453088884465 |
| PAFAH1B1 | 0.379902052532405 | 0.000942895015904716 | 0.0311864644974 |
| SNRNP27 | 0.380388083856688 | 0.00361038887249859 | 0.0560098165625457 |
| C19orf2 | 0.380902998596706 | 0.00334554648057331 | 0.0539816734387994 |
| IL33 | 0.380984027485816 | 0.00066455157408718 | 0.0278642959754539 |
| MYC | 0.381649781656767 | 0.000971338549357864 | 0.031419702037191 |
| TGFBR2 | 0.381658481263469 | 3,91E+14 | 0.0239757643173249 |
| NDUFB5 | 0.381719383673008 | 0.00851915089082884 | 0.0844384553320816 |
| ALS2CR4 | 0.382189192440385 | 0.00675032053491864 | 0.0748606499828485 |
| NAE1 | 0.382208247319097 | 0.000354111621273716 | 0.0248077604528682 |
| PICALM | 0.382716804238857 | 0.00022016949771305 | 0.0239757643173249 |
| REV1 | 0.382905160179599 | 0.000694874423245087 | 0.0280690958739751 |
| LYSMD2 | 0.38315415952212 | 0.00707702938759408 | 0.0769820180111139 |
| C7orf23 | 0.383236262152983 | 0.00559193578475316 | 0.0675291243042718 |
| UPF3B | 0.383622639557411 | 0.000603959724990652 | 0.0276956297030149 |
| OXCT1 | 0.383760574923369 | 3,13E+14 | 0.0239757643173249 |
| MKL2 | 0.383838504489899 | 0.00122727577625962 | 0.0338245096530109 |
| MRPS35 | 0.384414731910489 | 0.000222130995578379 | 0.0239757643173249 |
| UFM1 | 0.38497476711741 | 0.000160594274391822 | 0.0239757643173249 |
| CRLS1 | 0.386049224424628 | 0.00142686474670732 | 0.0362611369418848 |
| CAV1 | 0.386059157087304 | 0.00696702947535102 | 0.0763483690893305 |
| PTPRK | 0.38616808365176 | 0.00569022328076687 | 0.0681925534425092 |
| C1QBP | 0.386744872309672 | 0.00553655052985609 | 0.067271109576631 |
| FAM122B | 0.387091870341931 | 0.00496499079293142 | 0.0636109406478712 |
| CTNNAL1 | 0.387237178382669 | 0.000966830224605173 | 0.031419702037191 |
| FAM83D | 0.38765002814045 | 0.00382534709594188 | 0.0574699709016684 |
| SH3BGRL | 0.38836915834531 | 0.00461542856325327 | 0.0616106045420321 |
| RECK | 0.388452306832011 | 0.00772083897856751 | 0.08037591346919 |
| CENTB2 | 0.388973697207687 | 0.000675102523688694 | 0.0279546184311492 |
| CCDC76 | 0.389136849960324 | 0.00888229681051956 | 0.0866235952798865 |
| RAB21 | 0.389416229301952 | 0.00845792273938568 | 0.0842502600690758 |
| ITM2B | 0.389537206037895 | 0.0051169209393495 | 0.0646744616221806 |
| GLCE | 0.39046645170137 | 0.000594478474967094 | 0.0276956297030149 |
| DDX60 | 0.391743510262959 | 0.00106518650455735 | 0.0324144728863388 |
| ITGAE | 0.391826767905051 | 0.00234728043190526 | 0.0451623851227603 |
| MRPL3 | 0.392953766267653 | 0.0102496709254496 | 0.0935610652120838 |
| NMI | 0.393182085478703 | 0.0015423536478183 | 0.0373382380162451 |
| C20orf30 | 0.393923807252507 | 0.00351555987587682 | 0.0552500330520554 |
| PPPDE1 | 0.394697030766706 | 0.000842537085952232 | 0.0297767990079848 |
| COMMD10 | 0.39529715199589 | 0.000248017445639519 | 0.0239757643173249 |
| ZDHHC17 | 0.39581795620851 | 0.00549090819821689 | 0.0671428233574088 |
| ZNF518B | 0.396332877359909 | 0.00175322187557615 | 0.0394380153254603 |
| TMEM126A | 0.396347055569115 | 0.00636946928971302 | 0.0723728230693263 |
| SERP1 | 0.396914489105597 | 0.00691062974644203 | 0.0758801733240594 |
| RIN2 | 0.39757327914022 | 0.00828657507523143 | 0.0830953787363268 |
| ACTR6 | 0.39963645072882 | 0.00432670859201008 | 0.0607271426834737 |
| PDHX | 0.399964479556543 | 0.00280841933190782 | 0.0500881522232834 |
| HNRPDL | 0.400254688686246 | 0.000906733314888854 | 0.0306778104870729 |
| ABHD3 | 0.400357939981571 | 0.000177763823379193 | 0.0239757643173249 |
| MRFAP1 | 0.400588316642387 | 0.00112121223853317 | 0.0329165765831096 |
| CAT | 0.400735307490784 | 3,64E+14 | 0.0239757643173249 |
| RAB11FIP2 | 0.400890765141175 | 0.00255383791999661 | 0.0475674471659167 |
| SNORD31 | 0.401222026195681 | 0.000307020995821389 | 0.0247792613550641 |
| SET | 0.40127131686048 | 0.0112074657163913 | 0.0979314825800781 |
| TFDP1 | 0.403572459447151 | 0.00667618670267984 | 0.074485123225743 |
| KIAA1370 | 0.403713751741177 | 0.00111256762043493 | 0.0327204957769608 |
| CDC42SE2 | 0.40381970278298 | 0.00589547699363143 | 0.0696989417869238 |
| PEX1 | 0.404003085249047 | 0.000189690511094389 | 0.0239757643173249 |
| SLMAP | 0.404038801081016 | 0.00294057296728164 | 0.0514708492254156 |
| LEPROTL1 | 0.404176716956127 | 0.00299111711372688 | 0.051833729843994 |
| YEATS4 | 0.404348623588548 | 0.00163925699624815 | 0.0383727872697743 |
| FLJ20718 | 0.404624129156294 | 0.000917964290644413 | 0.0309034273478687 |
| LTA4H | 0.405655764706969 | 0.00040652050587005 | 0.0256150411917438 |
| CHMP2B | 0.40691253599485 | 0.0115544188076445 | 0.0991043202782726 |
| FAM96A | 0.407816928710944 | 0.000620408197901532 | 0.0278046472365304 |
| EML4 | 0.408782398845521 | 0.000207107867743262 | 0.0239757643173249 |
| C14orf32 | 0.410373721611099 | 0.00108514039229129 | 0.0324969469383994 |
| BET1 | 0.410926249315725 | 0.000584769810049101 | 0.0276956297030149 |
| MTM1 | 0.411330668504297 | 0.00150945662664254 | 0.0372481964577041 |
| TMEM209 | 0.412711723512617 | 0.00282479775336963 | 0.0501831199600755 |
| TBC1D4 | 0.412962164860695 | 0.00356339812301053 | 0.0553730541650761 |
| RBBP7 | 0.41342327864271 | 0.00454130700497513 | 0.0614063625819381 |
| CDKN1B | 0.413500356646796 | 0.00451968242749921 | 0.0613699903293107 |
| NEBL | 0.41352243617311 | 0.000126368485498583 | 0.0239757643173249 |
| YTHDC2 | 0.415130436139306 | 0.00062267767974542 | 0.0278046472365304 |
| KIAA0494 | 0.415355746559054 | 0.00338960133758163 | 0.0543306492394007 |
| UQCRH | 0.415767566669008 | 0.00880335501896925 | 0.0863019126300131 |
| MRPL33 | 0.415785333060627 | 0.00418026645941857 | 0.0596910936534627 |
| SLC35A1 | 0.416079353648297 | 0.00354952010769465 | 0.0553730541650761 |
| ANKRD10 | 0.416873819457967 | 0.000651090761869787 | 0.0278046472365304 |
| ADSS | 0.418857326893775 | 0.0106794651615858 | 0.0954728126099659 |
| PDS5A | 0.419368850584037 | 0.000157294838407106 | 0.0239757643173249 |
| YPEL5 | 0.419556654897735 | 0.00322512920817745 | 0.0531450352404207 |
| ANKRD50 | 0.419655713738265 | 0.00888557002649508 | 0.0866235952798865 |
| PDGFRA | 0.420997256965626 | 0.00341582341811042 | 0.0545527466858994 |
| CBR4 | 0.421182598212914 | 0.0040234164484015 | 0.0588521882250363 |
| SERINC1 | 0.421278029603941 | 0.00417168339671428 | 0.0596627298595866 |
| MRPL50 | 0.421599262383095 | 0.00887835029945283 | 0.0866235952798865 |
| ZNF83 | 0.422611070902524 | 0.00643951793992857 | 0.0728122705472372 |
| ZMPSTE24 | 0.424248652893485 | 0.0098138970451413 | 0.0913147737358424 |
| TMED5 | 0.424405084654882 | 0.00969874627947283 | 0.0909443189567861 |
| CYCSL1 | 0.424853615573585 | 0.000694167407222879 | 0.0280690958739751 |
| TMX1 | 0.425722752344015 | 0.00350432386909356 | 0.0551872990775131 |
| IRS2 | 0.426510984023061 | 0.0115783029520901 | 0.0991286993899792 |
| BTF3 | 0.426685231621048 | 0.00702993070106695 | 0.0766842899409964 |
| CCNC | 0.42753595947134 | 5,20E+14 | 0.0239757643173249 |
| ARL5A | 0.428387602765327 | 0.00846995952537107 | 0.0842502600690758 |
| OR3A4 | 0.431089315247955 | 0.0108119143655096 | 0.0960580521779395 |
| PDCD5 | 0.432067318255251 | 0.000987306356741035 | 0.031544532848966 |
| APP | 0.433178366840897 | 0.00214407501410121 | 0.043135763153786 |
| PDZD2 | 0.434928920918342 | 0.00138564622436338 | 0.0360397922668013 |
| KIAA1598 | 0.435354659286222 | 0.00345069499733177 | 0.0547060187502197 |
| SLC39A8 | 0.436200043414571 | 0.000274274011947971 | 0.0244391352696618 |
| BCLAF1 | 0.436588064347499 | 0.002591113333647 | 0.0477015502496782 |
| FUNDC1 | 0.436626806288891 | 9,04E+14 | 0.0239757643173249 |
| PRMT3 | 0.436966186961519 | 0.00215852004112347 | 0.0433150489997752 |
| ARRDC4 | 0.437273469970768 | 0.00301622151653784 | 0.0519099819851916 |
| EHF | 0.437513180684112 | 0.000654203577906159 | 0.0278046472365304 |
| MBIP | 0.437821111693611 | 0.000298017664598159 | 0.0247792613550641 |
| TAF2 | 0.43783378464143 | 0.00917289035021653 | 0.0884525407786117 |
| ETS1 | 0.437948973823935 | 0.00514791357519281 | 0.064834872247295 |
| ATP5O | 0.43859152665224 | 0.0012565530186427 | 0.0342785854538313 |
| NCOA7 | 0.438759870598006 | 0.00348328496241878 | 0.0551691355703358 |
| SLC25A43 | 0.439060924964685 | 0.000600324226764359 | 0.0276956297030149 |
| MYLK | 0.440004713236145 | 0.00490211956209035 | 0.0632933272718721 |
| LIMA1 | 0.440492293034321 | 0.007148039589429 | 0.0772638097439189 |
| ACP1 | 0.440578617172908 | 0.00621302871093685 | 0.0715723708804532 |
| ATL2 | 0.440632691035151 | 0.00225865344093029 | 0.0440954381510228 |
| TMEM126B | 0.441296867178268 | 0.00284754427534055 | 0.0504257680928923 |
| MFF | 0.443761937658321 | 0.00012822309044476 | 0.0239757643173249 |
| ANKMY2 | 0.444648069319756 | 0.000587398909147021 | 0.0276956297030149 |
| CAV2 | 0.445129128298133 | 0.00052307786341429 | 0.0267912434289055 |
| KIAA1737 | 0.446020606097588 | 8,17E+13 | 0.0239757643173249 |
| BCL2L2 | 0.446239298521612 | 0.00216990172872236 | 0.0433541600152024 |
| EIF4E3 | 0.446778565745039 | 0.00104959889365591 | 0.0324144728863388 |
| G3BP2 | 0.447329027673351 | 0.000586173876179541 | 0.0276956297030149 |
| MRPL32 | 0.447525518629421 | 0.000340913439920762 | 0.0248077604528682 |
| ATP2C1 | 0.447618284978167 | 0.000230560748414679 | 0.0239757643173249 |
| FRMD4A | 0.447965948945826 | 0.00967475293603166 | 0.0909325424619606 |
| MFSD1 | 0.449543365469625 | 0.0108794831299065 | 0.096483684699213 |
| TMEM188 | 0.451555747212552 | 0.000831672567409389 | 0.0297696230455384 |
| ARMCX2 | 0.451713119003881 | 0.000314387173132473 | 0.0247792613550641 |
| DOCK9 | 0.4519766447987 | 0.0010119162241649 | 0.031962727642218 |
| MCEE | 0.453993575553098 | 0.00217906422430575 | 0.0433541600152024 |
| DMTF1 | 0.454283078343452 | 0.00451998126539319 | 0.0613699903293107 |
| ZNF800 | 0.454648410547567 | 0.00379985654925538 | 0.0573632542175886 |
| TFB2M | 0.455997311923305 | 0.00161995738317925 | 0.0381412615109841 |
| ZNF770 | 0.456680803001713 | 0.00593909223661878 | 0.0698398436072017 |
| EPHA4 | 0.45708068692706 | 0.0021728842336524 | 0.0433541600152024 |
| WEE1 | 0.457092666775435 | 7,23E+14 | 0.0239757643173249 |
| HPRT1 | 0.458410134828009 | 0.000973961687564012 | 0.031419702037191 |
| OGT | 0.459737327245607 | 9,40E+14 | 0.0239757643173249 |
| IGFBP7 | 0.460422072305926 | 0.0069756699003067 | 0.0763927639213851 |
| IARS2 | 0.460919240206123 | 0.000894198508592789 | 0.0306272188766164 |
| TSPAN3 | 0.461363920015631 | 0.000297749522537183 | 0.0247792613550641 |
| PTS | 0.463274968915239 | 0.00416992035076043 | 0.0596627298595866 |
| UBB | 0.46362103556449 | 0.00692874981648799 | 0.0760289844728142 |
| HSD17B4 | 0.463786694326874 | 0.00433362006483889 | 0.0607314473097 |
| TSPYL1 | 0.464049505954833 | 0.00752917229751204 | 0.0793734021940377 |
| sep-10 | 0.464099131105076 | 0.00157598016120669 | 0.0376555014846483 |
| GPSM2 | 0.464381023686599 | 0.00658059485206718 | 0.0739140228795615 |
| CAB39 | 0.46483395241557 | 0.00438209257715867 | 0.0608597912300807 |
| THEM2 | 0.46487425409846 | 0.00104723750559404 | 0.0324144728863388 |
| RERG | 0.465232323879158 | 0.000540165618622434 | 0.0273300817251946 |
| LXN | 0.465835199163353 | 0.00327591721820493 | 0.0535141491798226 |
| KIAA1468 | 0.466613585578447 | 0.00410258887669763 | 0.0594317107781622 |
| MAP3K8 | 0.466651036276097 | 0.00120204338480919 | 0.0335326583455989 |
| OTUD4 | 0.466947715037293 | 0.00511532001612441 | 0.0646744616221806 |
| RPL15 | 0.468594861919417 | 0.0010342806854654 | 0.0324144728863388 |
| LAP3 | 0.468671589325014 | 0.00670598382973634 | 0.0746175179343524 |
| PREPL | 0.468808847375719 | 0.000323820545270863 | 0.0247792613550641 |
| C14orf138 | 0.469594523549133 | 0.0019996633215299 | 0.0418397471995718 |
| SLC35F5 | 0.470775341810611 | 0.00102385574943897 | 0.0322175856430267 |
| UBE2N | 0.471197204105995 | 0.00799576399257885 | 0.0819887850378902 |
| MTDH | 0.472296433359602 | 0.00208541525270894 | 0.0424475053654717 |
| DLD | 0.472664102458843 | 0.000143748634195954 | 0.0239757643173249 |
| SLK | 0.47285010971161 | 0.000402600586687697 | 0.0256150411917438 |
| FTSJD1 | 0.473895378506772 | 0.000182391057149931 | 0.0239757643173249 |
| ISOC1 | 0.47413669941867 | 0.000419255452124394 | 0.0256150411917438 |
| FAM116A | 0.474537272140224 | 0.00194924397451182 | 0.0415988656406715 |
| GCA | 0.475632006725528 | 0.00031651080873441 | 0.0247792613550641 |
| TAF7 | 0.476089110216025 | 0.00228451373635184 | 0.0443714994259888 |
| GPM6B | 0.476635297410061 | 0.0006964157512887 | 0.0280690958739751 |
| FAM190B | 0.476688996402379 | 0.00355913284663793 | 0.0553730541650761 |
| LRP11 | 0.476719728761981 | 0.00108961957340515 | 0.0324969469383994 |
| HMGN4 | 0.478415895266553 | 0.000489964098109397 | 0.0263094915391259 |
| ACN9 | 0.479287629838125 | 5,20E+14 | 0.0239757643173249 |
| GNG11 | 0.47947101809436 | 0.00429222773770027 | 0.0605418162855909 |
| CDH1 | 0.479789748921564 | 0.00485557681790944 | 0.0629814901556248 |
| STRAP | 0.480195931744729 | 0.00822707744737716 | 0.0827710798729406 |
| DR1 | 0.482154215344881 | 0.000424843375089477 | 0.0256150411917438 |
| PAQR3 | 0.482457596055599 | 0.000691763947740548 | 0.0280690958739751 |
| TM2D1 | 0.483655097383736 | 4,38E+14 | 0.0239757643173249 |
| BEX4 | 0.484466379678041 | 5,80E+14 | 0.0239757643173249 |
| MFSD6 | 0.48662042937485 | 0.0109311219736446 | 0.0966840894650842 |
| SEC23B | 0.487220827908976 | 0.000336328954751378 | 0.0247792613550641 |
| C1orf63 | 0.487932589486642 | 0.000995501216517371 | 0.0316242619277637 |
| SAT1 | 0.488124534093532 | 1,00E+15 | 0.0239757643173249 |
| SGCE | 0.488357449739385 | 0.00817275223961528 | 0.0827442748264649 |
| ANKRD12 | 0.488649365610786 | 0.00204394154838542 | 0.0421082314534947 |
| CCNG1 | 0.48979516754819 | 0.00190053742787629 | 0.041193158885975 |
| ZCCHC9 | 0.490330022060277 | 0.00324806300526588 | 0.0532642362209362 |
| KLHL9 | 0.4904753389402 | 0.00133189878528434 | 0.0354996676035456 |
| SLC35B3 | 0.490824679829773 | 0.00141768726818514 | 0.0362130573107614 |
| SCRG1 | 0.491759110534492 | 0.00204679340364757 | 0.0421148615539152 |
| TMEM66 | 0.492210680349521 | 0.00219025033262835 | 0.0434551931310269 |
| NOL7 | 0.492222116087002 | 0.0013042542387753 | 0.0351391085865032 |
| TRMT112 | 0.493234941417314 | 0.00479985158322025 | 0.0627638094691942 |
| PSMA3 | 0.493596165497125 | 0.000118070178045764 | 0.0239757643173249 |
| RYK | 0.493875648519615 | 0.000257841781932588 | 0.0239757643173249 |
| GPR126 | 0.494463277086647 | 0.00186880859514883 | 0.0406454402437993 |
| TOP2B | 0.494742717372353 | 0.00123414945784703 | 0.0339004156358444 |
| KIAA1012 | 0.495104022533071 | 0.00223315760075219 | 0.0437950007361145 |
| CD44 | 0.497465649913232 | 0.000274195641693722 | 0.0244391352696618 |
| GSTA4 | 0.498721909544936 | 0.0033150248854331 | 0.0538101935477045 |
| CPE | 0.499421081422126 | 0.0100262973514583 | 0.0921577833861818 |
| WBP4 | 0.499836843704654 | 0.000655329251937792 | 0.0278046472365304 |
| PSMA6 | 0.502562262882745 | 0.00375812633093816 | 0.057231226636824 |
| INSIG2 | 0.502664675029361 | 0.000450476868106068 | 0.0257685152800468 |
| KLF4 | 0.503048821387508 | 0.00799122387560187 | 0.0819887850378902 |
| LAMP2 | 0.503440063605447 | 0.00150956996666223 | 0.0372481964577041 |
| BRWD2 | 0.504779917184337 | 0.00148003666984054 | 0.0369920276368853 |
| SNX2 | 0.506044005504523 | 0.00195429430649574 | 0.0416086425260897 |
| RAPGEF4 | 0.506970786013809 | 0.00130457582040484 | 0.0351391085865032 |
| RNF103 | 0.508417319714829 | 0.000160851982787768 | 0.0239757643173249 |
| WDR26 | 0.508543447754941 | 0.00109130081332244 | 0.0324969469383994 |
| RAB38 | 0.508601889052752 | 0.00982893799097593 | 0.0913819472986147 |
| DEK | 0.508990881194277 | 0.00445188340979955 | 0.061098881984569 |
| RCN2 | 0.509027842956032 | 0.000413900660820437 | 0.0256150411917438 |
| SEPP1 | 0.512547044899321 | 0.000468649277938763 | 0.025891456847287 |
| CNIH | 0.512576935504041 | 0.00321501911522439 | 0.0531188040513332 |
| ANO6 | 0.512948873340116 | 0.000336423949672263 | 0.0247792613550641 |
| TMEM131 | 0.513327176906505 | 0.000603731426283906 | 0.0276956297030149 |
| KCTD3 | 0.514482467649449 | 0.000946118488484462 | 0.0311864644974 |
| PTPN12 | 0.515871530762025 | 0.000725684413162715 | 0.0286249827997786 |
| RAB9A | 0.51699205906082 | 0.00106370851502819 | 0.0324144728863388 |
| OXR1 | 0.517079010965977 | 0.00216236968153389 | 0.0433150489997752 |
| FAM84B | 0.517295258645567 | 0.000525804147204019 | 0.0268482694305463 |
| CLIP4 | 0.51814120083138 | 0.0110238143477648 | 0.097011597643616 |
| CMPK1 | 0.519963163603933 | 0.00168242137676221 | 0.0386171286585011 |
| ZMYM2 | 0.520657404654164 | 5,99E+14 | 0.0239757643173249 |
| C9orf5 | 0.52153803909656 | 0.000325177794833835 | 0.0247792613550641 |
| ZBTB33 | 0.522268076222607 | 0.00739798929833227 | 0.078688906451495 |
| MON2 | 0.523541748615912 | 0.000309115547660187 | 0.0247792613550641 |
| GNG12 | 0.524487304085882 | 0.00119728525911514 | 0.0335326583455989 |
| MKI67IP | 0.525338163317634 | 0.000237540754385542 | 0.0239757643173249 |
| ZC3H15 | 0.525433244295248 | 0.000291482971044738 | 0.0247792613550641 |
| ARL8B | 0.526220447492028 | 0.00508773379280724 | 0.0644570463798931 |
| NSUN2 | 0.527795784103102 | 0.000249763602379022 | 0.0239757643173249 |
| MTX3 | 0.529316428390519 | 0.000199517414735901 | 0.0239757643173249 |
| HSPA8 | 0.529551529158153 | 0.000170891832784705 | 0.0239757643173249 |
| MTPN | 0.529688034848122 | 0.00717411150582755 | 0.0772955591829716 |
| C3orf58 | 0.53018212437063 | 0.00154469363538194 | 0.0373382380162451 |
| LYRM5 | 0.530356834983899 | 0.0066609886421308 | 0.074415313380476 |
| COL15A1 | 0.531659048555253 | 0.00186234030134693 | 0.0405765924819648 |
| BMPR2 | 0.534998375012724 | 0.000658515394022056 | 0.0278046472365304 |
| GBE1 | 0.539392464349975 | 0.000312774628759074 | 0.0247792613550641 |
| C14orf166 | 0.539637001723871 | 0.00909659606016161 | 0.0881384970997963 |
| SMPDL3A | 0.540872821513494 | 0.000511903068409093 | 0.0265727974869209 |
| SEC61G | 0.542356420954869 | 0.00104245250455921 | 0.0324144728863388 |
| VBP1 | 0.543557209207022 | 0.00122107243977173 | 0.0337640728113624 |
| ZNF217 | 0.543686038474284 | 0.000346036271226741 | 0.0248077604528682 |
| PQLC3 | 0.543848918575395 | 0.000172375517387468 | 0.0239757643173249 |
| SERPINB2 | 0.54404283799463 | 0.00166956317302216 | 0.0385993730251762 |
| PHIP | 0.544293276938011 | 0.00555492409809711 | 0.0673959668636476 |
| DEGS1 | 0.544508873222029 | 0.0115560186026749 | 0.0991043202782726 |
| CALM2 | 0.545407317815983 | 0.00501535124680047 | 0.0638756976696562 |
| AFF4 | 0.545428425020654 | 0.00257481525447204 | 0.0475688381311017 |
| KIAA0261 | 0.546781739247856 | 0.000429724806081798 | 0.0256150411917438 |
| SDCBP | 0.549005381019822 | 0.000455290720873107 | 0.025891456847287 |
| TOR1AIP1 | 0.549141747257733 | 0.000344023305664306 | 0.0248077604528682 |
| SFRS2 | 0.549460240636142 | 0.00094649064608579 | 0.0311864644974 |
| TMEM14A | 0.550029684468138 | 8,08E+13 | 0.0239757643173249 |
| RSU1 | 0.550092769009761 | 0.00609768131607906 | 0.0706694262214385 |
| VPS54 | 0.550609910873654 | 2,50E+14 | 0.0239757643173249 |
| LTV1 | 0.551451726823833 | 0.00394514013199515 | 0.0584505015447552 |
| TMED7 | 0.551753913194765 | 0.00171391152767657 | 0.039028414896996 |
| KIAA0907 | 0.552805924827824 | 0.00652951648653944 | 0.0736883602948715 |
| IFI16 | 0.553516119845149 | 0.00497663065563007 | 0.0636109406478712 |
| PON2 | 0.55364192219549 | 0.00105860281291639 | 0.0324144728863388 |
| RBL2 | 0.554176882905064 | 0.00242548544012392 | 0.0459324580617778 |
| C14orf135 | 0.555793600320094 | 0.00126435046141789 | 0.0343895061777161 |
| LHFP | 0.558434019196125 | 0.00485018510456432 | 0.0629814901556248 |
| RAP2A | 0.559908099086692 | 0.000878216402273737 | 0.0304962121691716 |
| VAMP7 | 0.560174111596692 | 0.00190562829378789 | 0.0412497900889378 |
| SMAD4 | 0.562424423378172 | 0.000482177876281283 | 0.0261444069334796 |
| CYP1B1 | 0.562758944284435 | 0.001487729623429 | 0.0370474414028692 |
| PDCD6 | 0.565827648491707 | 5,13E+14 | 0.0239757643173249 |
| ANAPC13 | 0.566262859520754 | 4,40E+14 | 0.0239757643173249 |
| PPP1CC | 0.567101145557674 | 0.00224615411255625 | 0.0439876251266015 |
| CPNE3 | 0.56790246030548 | 0.000362000673419949 | 0.0249002611973077 |
| PRRC1 | 0.570305874601451 | 0.000227820919922657 | 0.0239757643173249 |
| F2R | 0.57078211182859 | 0.00191788738889427 | 0.0412682425059276 |
| TJP1 | 0.572184207933899 | 0.000685234466687303 | 0.0280690958739751 |
| KDELC2 | 0.573998991863007 | 0.00307585196415041 | 0.0521465049409614 |
| ARID5B | 0.577292648346813 | 0.000356533250184411 | 0.0248077604528682 |
| ATP5F1 | 0.578243945432798 | 0.000593804523504389 | 0.0276956297030149 |
| VPS29 | 0.579550522062034 | 0.000214315200853 | 0.0239757643173249 |
| LONRF1 | 0.580300373285134 | 0.00078132896399947 | 0.029106626873633 |
| SOSTDC1 | 0.580340229084396 | 0.00218255353194399 | 0.0433541600152024 |
| CHMP5 | 0.581426354132412 | 0.000257646362397297 | 0.0239757643173249 |
| VPS35 | 0.58384810171146 | 0.000272218241817074 | 0.0244391352696618 |
| SCOC | 0.584941673350816 | 0.000169225359336453 | 0.0239757643173249 |
| NAB1 | 0.584963038102088 | 0.0038044594891396 | 0.0573632542175886 |
| GOLPH3 | 0.585578859674668 | 0.00502155005982957 | 0.0638892510791362 |
| PSMD6 | 0.587055461777849 | 0.000117197266350464 | 0.0239757643173249 |
| PLSCR4 | 0.587942881773014 | 0.0020140778555427 | 0.0418844137451324 |
| DDX5 | 0.589609552067578 | 0.000405670231370099 | 0.0256150411917438 |
| OCIAD1 | 0.593345638558757 | 0.000549794040804427 | 0.0275377535242345 |
| SC5DL | 0.593599817485719 | 0.0107699591592177 | 0.0958186745934465 |
| ZFP36L2 | 0.594218307078508 | 0.00437751998078528 | 0.0608597912300807 |
| PELI1 | 0.601644451510853 | 0.000952803808417251 | 0.0312827853943068 |
| RPL17 | 0.602636152323643 | 0.00405889978246973 | 0.0592151146178713 |
| PLS3 | 0.605387453412312 | 0.00162859071982395 | 0.0382902840708892 |
| HECTD1 | 0.607270912400248 | 0.000318062885971471 | 0.0247792613550641 |
| NCK1 | 0.610045576893879 | 0.000694137967994086 | 0.0280690958739751 |
| LMBRD1 | 0.610365653935298 | 0.000835180461508256 | 0.0297696230455384 |
| KHDRBS1 | 0.611134179528333 | 0.00193932184978622 | 0.0415477529772433 |
| SH3D19 | 0.611571513580207 | 0.00260001004097002 | 0.0477174940925986 |
| HSPH1 | 0.611777105644273 | 0.000461132239695112 | 0.025891456847287 |
| DHX15 | 0.617281110833021 | 0.00017700796596332 | 0.0239757643173249 |
| RNF145 | 0.617917515975703 | 5,90E+14 | 0.0239757643173249 |
| C1orf55 | 0.621553254507344 | 9,03E+14 | 0.0239757643173249 |
| NDFIP2 | 0.623030250181589 | 0.00406712287241772 | 0.0592312575102934 |
| TANC1 | 0.623202412786739 | 0.000213010928269961 | 0.0239757643173249 |
| C8orf59 | 0.623620595530638 | 0.00626685393368989 | 0.0717791163773517 |
| YAP1 | 0.624336309505917 | 0.00789275475541991 | 0.0814524461616366 |
| XPO1 | 0.631259990422453 | 0.00031598732073791 | 0.0247792613550641 |
| PTPRZ1 | 0.632103787516837 | 0.000104666662635743 | 0.0239757643173249 |
| SASH1 | 0.632122275170535 | 0.000379416776973273 | 0.0250625859900678 |
| JAK1 | 0.632850842786183 | 0.000744694633894792 | 0.028858331434898 |
| CCT8 | 0.632983463396967 | 0.000144430068015553 | 0.0239757643173249 |
| CRYZ | 0.633714162388176 | 0.00301205885604414 | 0.0519099819851916 |
| EIF3M | 0.633728690423403 | 0.0011446069423963 | 0.0333327826408104 |
| TMEM14C | 0.63521793437628 | 0.00239735725823408 | 0.0456073244806452 |
| TACSTD1 | 0.639248307244148 | 0.000341323333196074 | 0.0248077604528682 |
| RAB5A | 0.64144693713438 | 9,65E+13 | 0.0239757643173249 |
| OAT | 0.646228982653834 | 0.000841226369759836 | 0.0297767990079848 |
| LANCL1 | 0.646377498872774 | 0.000262272115801302 | 0.0241203405504336 |
| RND3 | 0.646470721659807 | 8,00E+14 | 0.0239757643173249 |
| B2M | 0.647430932595431 | 0.00135215127432712 | 0.0357836408782976 |
| SSFA2 | 0.647733361810411 | 0.000695266796012062 | 0.0280690958739751 |
| DPM1 | 0.648131947408989 | 0.000741903552143584 | 0.028858331434898 |
| VPS4B | 0.660189789672564 | 0.000500355064135871 | 0.0263977209883983 |
| mar-07 | 0.660230869869491 | 0.0026089652870836 | 0.047829114723341 |
| ELF1 | 0.661568043719796 | 0.00114825408613456 | 0.0333327826408104 |
| EIF4G2 | 0.668725273784684 | 2,13E+14 | 0.0239757643173249 |
| MAP2K1IP1 | 0.66969942147775 | 0.000596744934034037 | 0.0276956297030149 |
| PGRMC1 | 0.671354679535661 | 0.00607653671699056 | 0.070604197382115 |
| SPARCL1 | 0.67422913225325 | 0.00121379030982817 | 0.0336185582319464 |
| ZFAND5 | 0.67457478351027 | 0.000250976138063107 | 0.0239757643173249 |
| SNAI2 | 0.680537657339012 | 7,54E+14 | 0.0239757643173249 |
| ATP6AP2 | 0.680727765566989 | 0.00325101910371688 | 0.0532642362209362 |
| PRNP | 0.682230493080857 | 0.000239422654556566 | 0.0239757643173249 |
| TM9SF2 | 0.684779879945109 | 0.0004877002542396 | 0.0263094915391259 |
| NUP160 | 0.686840274235931 | 1,82E+14 | 0.0239757643173249 |
| FUBP3 | 0.688475287833696 | 0.00372216250450247 | 0.0569477178767905 |
| CREG1 | 0.688673682245549 | 0.00927262759736836 | 0.0889637803952702 |
| SLC12A2 | 0.695252691585753 | 0.00349754749785849 | 0.0551872990775131 |
| NCKAP1 | 0.698999775365653 | 0.00342604502437978 | 0.0545527466858994 |
| PRKAR1A | 0.703908732688591 | 0.00821459198781937 | 0.0827601498538441 |
| PTPLB | 0.704759538504164 | 0.00223368710951347 | 0.0437950007361145 |
| CLINT1 | 0.713549300014442 | 0.00179105834557331 | 0.0398050163156387 |
| DAAM1 | 0.717035553050796 | 0.000481740786048213 | 0.0261444069334796 |
| C2orf25 | 0.724617479415855 | 0.000319378024561496 | 0.0247792613550641 |
| CD9 | 0.734308115148511 | 0.000529636821653323 | 0.0269612676857529 |
| METTL5 | 0.734631451909224 | 9,74E+14 | 0.0239757643173249 |
| PTPN13 | 0.741612129512311 | 0.000126181431181357 | 0.0239757643173249 |
| DDX21 | 0.741768044406119 | 0.00156237128530871 | 0.0375827058023828 |
| HNRPK | 0.746575475154973 | 0.00202440053145033 | 0.04191314831657 |
| sep-15 | 0.758554181825742 | 0.000199798203799788 | 0.0239757643173249 |
| ITGAV | 0.777148492465938 | 0.00559466962738505 | 0.0675291243042718 |
| SCEL | 0.777728576263488 | 0.00401073687965786 | 0.0588217851090614 |
| HNRPA2B1 | 0.780536671985461 | 0.00417201835615516 | 0.0596627298595866 |
| SMG1 | 0.782253122339021 | 0.00147933988827779 | 0.0369920276368853 |
| TMEM123 | 0.78253726924307 | 0.00196607380643446 | 0.0416624177472815 |
| AADACL2 | 0.791823121374626 | 0.00426699410093733 | 0.0604662458282505 |
| PIGY | 0.796690753192992 | 5,53E+14 | 0.0239757643173249 |
| CNBP | 0.827807551743364 | 2,02E+14 | 0.0239757643173249 |
| KIT | 0.830652091841837 | 0.000237937854132976 | 0.0239757643173249 |
| ENPP2 | 0.834043567652253 | 0.000112384150972664 | 0.0239757643173249 |
| HSP90AA1 | 0.847629046769214 | 4,26E+14 | 0.0239757643173249 |
| HIGD1A | 0.875443078690766 | 0.000968045070133943 | 0.031419702037191 |
| CA2 | 0.885003184549468 | 0.00154547915374221 | 0.0373382380162451 |
| NFE2L2 | 0.88565128424735 | 0.0059317081657304 | 0.0698398436072017 |
| TOMM20 | 0.921564051580968 | 0.000152835567194917 | 0.0239757643173249 |
